# Strategies to improve recruitment to randomised trials

**DOI:** 10.1002/14651858.MR000013.pub7

**Published:** 2026-09-15

**Authors:** Adwoa Parker, Gloria Mongelli, Camila Piccolo-Lawrance, Elizabeth Coleman, Athanasios Gkekas, Han-I Wang, Shoba Dawson, Heidi Green, Rosalind Way, Arti Rai, Hanne Bruhn, Frank Sullivan, Cherish Boxall, Markus Chan, Paul Manson, Catherine Hewitt, Caroline Fairhurst, Mike Clarke, Shaun Treweek

**Affiliations:** York Trials Unit, Department of Health SciencesUniversity of YorkYorkUK; Centre for Ethics in Medicine, Bristol Medical SchoolUniversity of BristolBristolUK; Health Equity and Patient Involvement SpecialistSelf-employedAberdeenshireUK; Section of Vascular SurgeryImperial College LondonLondonUK; Royal Oldham HospitalNorthern Care Alliance NHS Foundation TrustOldhamUK; Aberdeen Centre for EvaluationUniversity of AberdeenAberdeenUK; School of Medicine, Population and Behavioural Science Research DivisionUniversity of St AndrewsSt AndrewsUK; School of PsychologyUniversity of SouthamptonSouthamptonUK; Centre for Public HealthQueen’s University BelfastBelfastUK; Department of Women's and Children’s HealthUppsala UniversityUppsalaSweden

## Abstract

**Background:**

Recruiting participants to randomised controlled trials (RCTs) is challenging. Identifying effective recruitment strategies would benefit health research: poor recruitment leads to underpowered trials, reducing the reliability of findings and increasing the risk of wasted resources, ethical concerns, and trial failure. Evidence to inform recruitment strategies is increasingly generated through Studies Within A Trial (SWATs), which are methodological studies embedded within host RCTs. This is an update of a review last published in 2018.

**Objectives:**

Primary: to quantify the effects of strategies to improve recruitment of participants to RCTs.

Secondary: to evaluate recruitment strategies' cost‐effectiveness and impact on retention, and the equity, diversity, and inclusion (EDI) characteristics of recruited participants.

**Search methods:**

We used MEDLINE, Embase, and six other databases to identify the studies included in the review. We also sought unpublished recruitment SWATs through social media and targeted email dissemination to trial methodology networks. The latest search date was 16 February 2023.

**Selection criteria:**

We included randomised SWATs evaluating trial recruitment strategies embedded in healthcare and non‐healthcare trials. We excluded quasi‐randomised, hypothetical, questionnaire‐only, retention‐only, or clinician incentive studies.

**Data collection and analysis:**

Primary outcome: proportion of eligible participants or centres recruited.

Secondary outcomes: cost‐effectiveness, retention rates, and EDI characteristics of included participants.

We conducted random‐effects meta‐analysis for strategies evaluated in at least two studies; otherwise, we synthesised results narratively. We reported effects as risk differences (RDs) with 95% confidence intervals (CIs), and assessed between‐trial heterogeneity. We used GRADE to assess the certainty of evidence for the primary outcome. We expressed cost‐effectiveness as the incremental cost per additional participant recruited in pounds sterling (GBP).

**Main results:**

We identified 91 eligible studies (53 new to this update), providing 94 comparisons and involving at least 176,747 participants. Eighty‐one studies involved strategies aimed at trial participants, while 10 evaluated strategies aimed at recruiters. All were healthcare studies.

We found 65 recruitment strategies; 49 were evaluated in a single study. Only five strategies were supported by high‐certainty evidence according to GRADE criteria, and we focus on these strategies in the summary below.

**Open‐label trials versus blinded, placebo trials**. Open‐label trials recruited more participants than blinded trials (RD 10%, 95% CI 8% to 12%; 3 studies, 9004 participants), corresponding to approximately 10 additional participants per 100 approached. The studies involved mostly women in the UK and Estonia. No cost or retention data were reported.

**Telephone reminder versus no telephone reminder.** Telephone reminders to people who did not respond to an initial postal invitation boosted recruitment by 6% (95% CI 3% to 9%; 2 studies, 1450 participants), in trials with low underlying recruitment (we are less certain for trials with over 10% recruitment). The studies involved people with a mean age of 58 years in Canada and Norway. No cost or retention data were reported.

**Recruitment primer letter versus no letter**. Pre‐recruitment letters and leaflets designed to encourage participation made little or no difference to recruitment (absolute improvement 1%, 95% CI −1% to 2%; 2 studies, 5376 participants), and were associated with increased costs compared to not sending a primer (incremental cost: GBP 2.08). The studies involved mostly older white people in the UK and Ireland.

**Multimedia information via a digital link/QR code plus paper participant information leaflet (PIL) versus paper PIL alone**. This made little or no difference to recruitment (absolute improvement 0%, 95% CI −1% to 1%; 7 studies, 11,612 participants) and retention (absolute improvement 0%, 95% CI −2% to 3%; 5 studies, 7403 participants), and increased costs compared to not including multimedia information (incremental cost: GBP 0.78). The studies involved people in the UK.

**Optimised, user‐tested PIL versus standard PIL**. Optimising participant information leaflets (e.g. through user‐testing the leaflet with the target population to shape its content, format, and appearance) made little or no difference to recruitment: absolute improvement was 0% (95% CI 0% to 1%; 6 studies, 27,805 participants). The studies involved people in the UK. Only one study reported EDI data; participants were mostly older women. No cost or retention data were reported.

We had moderate‐certainty evidence for 13 other strategies; confidence was often reduced because the results came from single studies. Seven strategies involved changes to how potential participants received information; four involved changes to trial conduct; one targeted the recruiter or recruitment site; and one tested non‐monetary incentives.

We had much less confidence in the other 47 comparisons because the studies had design flaws, were single studies, or had very uncertain results.

Costs were reported in only 17 of 91 studies. Strategy impact on retention was reported in 15 studies. All but one study (99%) were from high‐income countries. The most reported demographics were age (49 studies), sex (32 studies), gender (27 studies), and education level (16 studies).

**Authors' conclusions:**

The evidence on strategies to improve trial recruitment remains broad but lacks depth. Of 65 strategies evaluated, only five were supported by high‐certainty evidence. Open‐label trial designs and telephone reminders to non‐responders increased recruitment, while optimised participant information leaflets, recruitment primer letters, and multimedia information provided alongside a paper participant information leaflet had little or no effect. Reporting of participant characteristics was poor, limiting assessment of equity, diversity, and inclusion across most studies.

Evidence is heavily skewed toward high‐income countries. Future research must prioritise evaluations in low‐to‐middle‐income settings and consistently report cost, retention, and EDI outcomes.

We strongly urge the methodology research community to strengthen the evidence base by prioritising replications of existing strategies over the development and testing of new ones.

**Funding:**

National Institute for Health and Care Research (Advanced Fellowship, Adwoa Parker, reference:NIHR302256).

Health Research Board, Republic of Ireland, Evidence Synthesis Ireland (grant ESI‐2021‐001)

**Registration:**

This review updates an earlier Cochrane review, which was first published in 2002 and subsequently updated in 2007, 2010, and 2018. Previous versions of the review and their protocols are available at:

https://doi.org/10.1002/14651858.MR000013.pub2

https://doi.org/10.1002/14651858.MR000013.pub3

https://doi.org/10.1002/14651858.MR000013.pub4

https://doi.org/10.1002/14651858.MR000013.pub5

https://doi.org/10.1002/14651858.MR000013.pub6

## Plain language summary

**How can researchers get people to join their trial?**

**Key messages**

- Telling people what treatment they will receive – rather than them not being told – improves recruitment.
- Telephoning people who don't respond to an initial postal invitation to join a trial improves recruitment when the initial recruitment rate is low.
- Tailored, user‐tested information leaflets, sending letters about the importance of research, or providing a link to videos make little or no difference to recruitment.

**What are research trials?**

Research trials compare different approaches, such as treatments, programmes, or ways of delivering services. In health care, trials are essential to test new medicines and devices on human volunteers (‘trial participants’) to make sure they are safe and effective. However, finding and keeping trial participants is challenging. About half of all trials fail to recruit the number of people they need on time. Strategies to boost recruitment include changing trial methods and improving information for potential participants and how they are invited to take part.

**What did we want to find out?**

We wanted to find out which strategies encouraged more people to join research trials, and whether they:

- were good value for money (cost‐effective);
- made any difference to whether people stayed in the trial until the end (retention); and
- had any effect on the diversity of the people and places involved.

**What did we do?**

We searched for studies that tested different recruitment strategies embedded within research trials. Potential participants were chosen by chance to receive different recruitment approaches, and the studies measured how many people joined the trial.

We compared and summarised the results of the studies and rated our confidence in the evidence, based on factors such as methods and the number of people participating.

**What did we find?**

We found 91 studies, each involving between 37 and 30,000 participants, mostly conducted in the UK (46 studies) and USA (23 studies). All were in healthcare trials. Nearly all (99%) were carried out in high‐income countries. Most studies gave little information about the people they recruited.

The studies tested 65 different recruitment strategies ranging from changes to trial methods to how people were approached and informed about taking part. Participants had different health concerns (e.g. cancer, surgery).

We are confident in the evidence for only 5 of the 65 strategies investigated, and we focus on these below.

**Main results**

*Trial procedures*

- Potential participants are more likely to join an ‘open‐label’ trial, where they know their treatment, than a ‘blinded’ trial, where they do not know which treatment they will receive (3 trials, 9004 participants).
- For every 100 people invited, an open‐label trial recruits 10 more participants.
- The trials involved mostly women in the UK and Estonia.
- There was no information about cost‐effectiveness and retention.

*Telephone reminders*

- Telephoning people who have not responded to a postal invite improves recruitment (2 trials, 1450 people).
- Six more people take part if 100 people are called.
- Participants were aged 58 years on average and lived in Canada and Norway.
- There was no information on cost‐effectiveness or retention.

*Letters about the importance of research*

- Sending letters about the importance of research before the trial invitation does not significantly improve recruitment (2 trials, 5376 participants).
- One more person joins if 100 people are sent a letter.
- This small gain was not good value, costing 2.08 pounds sterling (GBP) per additional participant recruited.
- The trials involved mostly older white people in the UK and Ireland.

*QR code or link to videos alongside printed information leaflets*

- Providing a QR code or link to videos alongside a printed information leaflet makes no difference to recruitment (7 trials, 11,612 participants) or retention (5 trials, 7403 participants).
- There is zero change per 100 people.
- This strategy was not cost‐effective (GBP 0.78 more).
- The trials involved people in the UK.

*Information leaflets*

- Information leaflets developed with input from people similar to those being recruited make no difference compared to standard leaflets (6 trials, 27,805 participants).
- Both types of leaflet result in 7 in 100 people joining the trial.
- The trials involved people in the UK, mostly older women in one trial.
- There was no information on cost‐effectiveness or retention.

**What are the limitations of the evidence?**

- Our confidence in all but the five strategies above is limited because they were tested in only one trial, the trials testing them had problems with their methods, or the results were very uncertain.
- We found cost information for only 8 strategies, and retention information for only 15 trials.
- Few trials reported information about participants' age, sex, gender, education level, and income level, so we are not sure how effective the strategies would be for different people.
- Most trials (75%) were carried out in the UK or USA, so our findings may not apply to healthcare systems in other parts of the world. We screened all non‐English studies, none were eligible.

**How up to date is this evidence?**

This review updates our 2018 review. The evidence is current to February 2023.

## Summary of findings

**Summary of findings 1:** Open‐label trial versus blinded trial

**Open RCT versus blinded RCT**						
**Patient or population**: individuals eligible for a trial **Settings**: any **Intervention**: open‐label trial **Comparison**: blinded, placebo trial						
Outcomes	Illustrative effects* (95% CI)		Relative effect (95% CI)	No. of participants (studies)	Certainty of the evidence (GRADE)	**Cost‐effectiveness, retention, and EDI summary**
	**Effect with blinded trial**	Effect with open‐label trial				
**Number recruited**	1806 out of 4578	2162 out of 4426	**RD** 10% (8% to 12%)	9004 (3 studies)	⊕⊕⊕⊕ **High**	**Cost:** no data reported **Retention:** no data reported **EDI**: all studies were conducted in two high‐income countries. Participants were almost all women; their mean age, where reported, was 78 years. There was no information on sex, ethnicity, or socioeconomic status of participants.
**Proportion recruited**	**As measured**^a^		**RR 1.26** (1.19 to 1.32)			
	**39 per 100**	**50 per 100** (46 to 51)				
	**Low**^b^					
	**10 per 100**	**13 per 100** (12 to 13)				
	**Moderate**^b^					
	**30 per 100**	**38 per 100** (36 to 40)				
	**High**^b^					
	**50 per 100**	**63 per 100** (60 to 66)				
*The basis for the **assumed risk** (e.g. the median control group risk across studies) is provided in footnotes. The **effect for the open‐label trial** (and its 95% CI) is based on the assumed risk in the comparison group (blinded trial) and the **relative effect** of the intervention (and its 95% CI). **CI**: confidence interval; **EDI**: equity, diversity, and inclusion; **RD**: risk difference; **RR**: risk ratio						
**GRADE Working Group grades of evidence** **High certainty:** we are very confident that the true effect lies close to that of the estimate of the effect. **Moderate certainty:** we are moderately confident in the effect estimate; the true effect is likely to be close to the estimate of the effect, but there is a possibility that it is substantially different. **Low certainty:** our confidence in the effect estimate is limited; the true effect may be substantially different from the estimate of the effect. **Very low certainty:** we have very little confidence in the effect estimate; the true effect is likely to be substantially different from the estimate of effect.						

^a^This is the baseline recruitment measured in the included studies. ^b^We selected the low, moderate, and high illustrative recruitment levels of 10%, 30%, and 50% based on our prior experience with trial recruitment.

**Summary of findings 2:** Telephone reminder versus no telephone reminder

**Telephone reminder versus no telephone reminder**						
**Patient or population**: individuals eligible for a trial **Settings**: any **Intervention**: telephone reminder **Comparison**: no telephone reminder						
Outcomes	Illustrative comparative risks* (95% CI)		Relative effect (95% CI)	No. of participants (studies)	**Certainty** of the evidence (GRADE)	**Cost‐effectiveness, retention, and EDI summary**
	**Effect with no telephone reminder**	Effect with t**elephone reminder**				
**Number recruited**	46 out of 714	90 out of 736	**RD 6%** (3% to 9%)	1450 (2 studies)	⊕⊕⊕⊕ **High**^c^	**Cost**: no data reported **Retention**: no data reported **EDI**: both studies were conducted in high‐income countries. Participant mean age, where reported, was 58 years. No data reported on sex, gender, ethnicity, or socioeconomic status of participants. Both included studies had very low baseline recruitment of < 10%.
**Proportion recruited**	**As measured**^a^		**RR 1.94** (1.25 to 3.02)			
	6 per 100	12 per 100 (8 to 18)				
	**Low**^b^					
	**10 per 100**	**19 per 100** (13 to 30)				
	**Moderate**^b^					
	**30 per 100**	**58 per 100** (38 to 91)				
	**High**^b^					
	**50 per 100**	**97 per 100** (63 to 100)				
*The basis for the **assumed risk** (e.g. the median control group risk across studies) is provided in the footnotes. The **effect with thetelephone reminder** (and its 95% CI) is based on the assumed risk in the comparison group (no reminder) and the **relative effect** of the intervention (and its 95% CI). **CI**: confidence interval; **EDI**: equity, diversity, and inclusion; **RD**: risk difference; **RR**: risk ratio						
**GRADE Working Group grades of evidence** **High certainty:** we are very confident that the true effect lies close to that of the estimate of the effect. **Moderate certainty:** we are moderately confident in the effect estimate; the true effect is likely to be close to the estimate of the effect, but there is a possibility that it is substantially different. **Low certainty:** our confidence in the effect estimate is limited; the true effect may be substantially different from the estimate of the effect. **Very low certainty:** we have very little confidence in the effect estimate; the true effect is likely to be substantially different from the estimate of effect.						

^a^This is the baseline recruitment measured in the included studies. ^b^We selected the low, moderate, and high illustrative recruitment levels of 10%, 30%, and 50% based on our prior experience with trial recruitment. ^c^The evidence for this intervention comes entirely from trials with low (< 10%) underlying recruitment. When applied to trials with higher recruitment, we would downgrade the assessment of evidence certainty to moderate due to indirectness.

**Summary of findings 3:** Recruitment primer letter/leaflet versus no letter/leaflet

**Recruitment primer letter/leaflet versus no letter/leaflet**						
**Patient or population**: individuals eligible for a trial **Settings**: any **Intervention**: recruitment primer letter/leaflet **Comparison**: no letter/leaflet						
Outcomes	Illustrative effects* (95% CI)		Relative effect (95% CI)	No. of participants (studies)	Certainty of the evidence (GRADE)	Cost‐effectiveness, retention, and EDI summary
	**Effect without letter/leaflet**	Effect with primer letter/leaflet				
**Number recruited**	344 out of 3421	280 out of 1955	**RD 1%** (−1% to 2%)	5376 (2 studies)	⊕⊕⊕⊕ **High**	**Cost:** data from 1 study^c^ only: adding letter/primer, ICER = GBP 208.38; incremental costs = GBP 2.08; partial incremental effectiveness = 1% (RD 0.01, 95% CI −0.01 to 0.02) **Retention:** no data reported **EDI:** both studies were conducted in high‐income countries. Participant mean age, where reported, was 77 years; 43% male participants, 57% female; ethnicity > 99% White. No data on socioeconomic status of participants.
**Proportion recruited**	**As measured**^a^		**RR 1.30** (0.90 to 1.17)			
	**2 per 100**	**2 per 100** (1 to 4)				
	**Low**^b^					
	**10 per 100**	**10 per 100** (6 to 18)				
	**Moderate**^b^					
	**30 per 100**	**30 per 100** (17 to 54)				
	**High**^b^					
	**50 per 100**	**50 per 100** (28 to 90)				
*The basis for the **assumed risk** (e.g. the median control group risk across studies) is provided in the footnotes. The **effect for the recruitment primer letter/leaflet** (and its 95% CI) is based on the assumed risk in the comparison group (no letter/leaflet) and the **relative effect** of the intervention (and its 95% CI). **CI**: confidence interval; **EDI**: equity, diversity, and inclusion; **GBP:** pounds sterling; **ICER**: incremental cost‐effectiveness ratio; **RD**: risk difference; **RR**: risk ratio.						
**GRADE Working Group grades of evidence** **High certainty:** we are very confident that the true effect lies close to that of the estimate of the effect. **Moderate certainty:** we are moderately confident in the effect estimate; the true effect is likely to be close to the estimate of the effect, but there is a possibility that it is substantially different. **Low certainty:** our confidence in the effect estimate is limited; the true effect may be substantially different from the estimate of the effect. **Very low certainty:** we have very little confidence in the effect estimate; the true effect is likely to be substantially different from the estimate of effect.						

^a^This is the baseline recruitment measured in the included studies. ^b^We selected the low, moderate, and high illustrative recruitment levels of 10%, 30%, and 50% based on our prior experience with trial recruitment. ^c^Data from Arundel 2017 (falls prevention).

**Summary of findings 4:** Multimedia participant information leaflet (PIL) (provided as link/QR code) plus paper PIL versus paper PIL alone

**Multimedia information (provided as link/QR code) plus paper participant information leaflet (PIL) versus paper PIL alone**						
**Patient or population**: individuals eligible for a trial **Settings**: any **Intervention**: multimedia information PIL (provided as link/QR code) plus paper PIL **Comparison**: paper PIL alone						
Outcomes	Illustrative effects* (95% CI)		Relative effect (95% CI)	No. of participants (studies)	Certainty of the evidence (GRADE)	Cost‐effectiveness and EDI summary
	**Effect with paper PIL only**	Effect with multimedia information + paper PIL				
**Number recruited**	516 out of 6029	451 out of 5583	**RD 0%** (−1% to 1%)	11,612 (7 studies)	⊕⊕⊕⊕ **High**	**Cost:** data from 1 study^c^ only: multimedia information plus paper PIL was more costly (GBP 0.78 higher) and slightly less effective than paper PIL alone (RD −0.01, 95% CI −0.03 to 0.01); difference not statistically significant. **EDI**: two studies were conducted in one high‐income country (UK). No data reported on age, sex, gender, ethnicity or socioeconomic status of participants.
**Proportion recruited**	**As measured**^a^		**RR 0.97** (0.83 to 1.13)			
	**9 per 100**	**8 per 100** (7 to 10)				
	**Low**^b^					
	**10 per 100**	**10 per 100** (8 to 11)				
	**Moderate**^b^					
	**30 per 100**	**29 per 100** (25 to 34)				
	**High**^b^					
	**50 per 100**	**49 per 100** (42 to 57)				
**Number retained**	306 out of 3480	354 out of 3923	**RD 0%** (−2% to 3%)	7403 (5 studies)	⊕⊕⊕⊕ **High**	
*The basis for the **assumed risk** (e.g. the median control group risk across studies) is provided in the footnotes. The **effect for the multimedia information participant information (provided as link/QR code) alongside paper PIL** (and its 95% CI) is based on the assumed risk in the comparison group (paper PIL alone) and the **relative effect** of the intervention (and its 95% CI). **CI**: confidence interval; **EDI**: equity, diversity and inclusion; **GBP:** pounds sterling; **RD**: risk difference; **RR**: risk ratio						
**GRADE Working Group grades of evidence** **High certainty:** we are very confident that the true effect lies close to that of the estimate of the effect. **Moderate certainty:** we are moderately confident in the effect estimate; the true effect is likely to be close to the estimate of the effect, but there is a possibility that it is substantially different. **Low certainty:** our confidence in the effect estimate is limited; the true effect may be substantially different from the estimate of the effect. **Very low certainty:** we have very little confidence in the effect estimate; the true effect is likely to be substantially different from the estimate of effect.						

^a^This is the baseline recruitment measured in the included studies. ^b^We selected the low, moderate, and high illustrative recruitment levels of 10%, 30%, and 50% based on our prior experience with trial recruitment. ^c^Jolly 2019 (START MMI PSM‐COPD)

**Summary of findings 5:** Optimised participant information leaflet (PIL) (bespoke and user‐tested) versus usual PIL

**Optimised participant information leaflet (PIL) (bespoke and user‐tested) versus usual PIL**						
**Patient or population**: individuals eligible for trial **Settings**: any **Intervention**: optimised PIL (bespoke and user‐tested) **Comparison**: usual PIL						
Outcomes	Illustrative comparative risks* (95% CI)		Relative effect (95% CI)	No. of participants (studies)	Certainty of the evidence (GRADE)	Cost‐effectiveness, retention, and EDI summary
	Effect with usual PIL	Effect with optimised PIL (bespoke & user‐tested)				
**Willingness to participate/number recruited**	937 out of 13,745	1007 out of 14,060	**RD 0%** (0% to 1%)	27,805 (6 studies)	⊕⊕⊕⊕ **High**	**Cost:** no data reported **Retention:** no data reported **EDI:** all six studies were conducted in one high‐income country (UK). Only one study reported EDI data: participants were mostly women with a mean age of 78 years. Other studies provided no information on the sex, gender, ethnicity, or socioeconomic status of participants.
**Proportion willing to participate/recruited**	**As measured**^a^		**RR 1.01** (0.93 to 1.1)			
	**7 per 100**	**7 per 100** (7 to 8)				
	**Low**^b^					
	**10 per 100**	**10 per 100** (9 to 11)				
	**Moderate**^b^					
	**30 per 100**	**30 per 100** (28 to 33)				
	**High**^b^					
	**50 per 100**	**51 per 100** (47 to 55)				
*The basis for the **assumed risk** (e.g. the median control group risk across studies) is provided in the footnotes. The **effect with theoptimised PIL** (and its 95% CI) is based on the assumed risk in the comparison group (usual PIL) and the **relative effect** of the intervention (and its 95% CI). **CI**: confidence interval; **EDI**: equity, diversity, and inclusion; **RD**: risk difference; **RR**: risk ratio						
**GRADE Working Group grades of evidence** **High certainty:** we are very confident that the true effect lies close to that of the estimate of the effect. **Moderate certainty:** we are moderately confident in the effect estimate; the true effect is likely to be close to the estimate of the effect, but there is a possibility that it is substantially different. **Low certainty:** our confidence in the effect estimate is limited; the true effect may be substantially different from the estimate of the effect. **Very low certainty:** we have very little confidence in the effect estimate; the true effect is likely to be substantially different from the estimate of effect.						

^a^This is the baseline recruitment measured in the included studies. ^b^We selected the low, moderate, and high illustrative recruitment levels of 10%, 30%, and 50% based on our prior experience with trial recruitment.

**Summary of findings 6:** Brief participant information leaflet (PIL) versus usual PIL

**Brief participant information leaflet (PIL) versus usual PIL**						
**Patient or population**: individuals eligible for a trial **Settings**: any **Intervention**: brief PIL **Comparison**: usual PIL						
Outcomes	Illustrative comparative risks* (95% CI)		Relative effect (95% CI)	No. of participants (studies)	Certainty of the evidence (GRADE)	Cost‐effectiveness, retention, and EDI summary
	Effect with usual PIL	Effect with brief PIL				
**Number recruited**	749 out of 2287	783 out of 2346	**RD 0%** (−1% to 2%)	4633 (2 studies)	⊕⊕⊕⊝ **Moderate**^c^	**Cost:** no data reported **Retention:** no data reported **EDI:** both studies were conducted in one high‐income country (UK). Only one study reported demographic data: participant mean age was reported as about 43 years. About 75% of participants were female, 25% male. There was no information on ethnicity or socioeconomic status of participants.
**Proportion recruited**	**As measured**^a^		**RR 0.99** (0.93 to 1.06)			
	**33 per 100**	**32 per 100** (31 to 35)				
	**Low**^b^					
	**10 per 100**	**10 per 100** (9 to 11)				
	**Moderate**^b^					
	**30 per 100**	**30 per 100** (28 to 32)				
	**High**^b^					
	**50 per 100**	**50 per 100** (47 to 53)				
*The basis for the **assumed risk** (e.g. the median control group risk across studies) is provided in the footnotes. The **effect with thebrief PIL** (and its 95% CI) is based on the assumed risk in the comparison group (usual PIL) and the **relative effect** of the intervention (and its 95% CI). **CI**: confidence interval; **EDI**: equity, diversity and inclusion; **RR**: risk ratio						
**GRADE Working Group grades of evidence** **High certainty:** we are very confident that the true effect lies close to that of the estimate of the effect. **Moderate certainty:** we are moderately confident in the effect estimate; the true effect is likely to be close to the estimate of the effect, but there is a possibility that it is substantially different. **Low certainty:** our confidence in the effect estimate is limited; the true effect may be substantially different from the estimate of the effect. **Very low certainty:** we have very little confidence in the effect estimate; the true effect is likely to be substantially different from the estimate of effect.						

^a^This is the baseline recruitment measured in the included studies. ^b^We selected the low, moderate, and high illustrative recruitment levels of 10%, 30%, and 50% based on our prior experience with trial recruitment. ^c^We downgraded the certainty of the evidence by one level for indirectness: Chen 2011 measured entry to pre‐randomisation phase, not recruitment.

**Summary of findings 7:** Participant information leaflet (PIL) developed with feedback from users versus usual PIL

**Participant information leaflet (PIL) developed with feedback from users versus usual PIL**						
**Patient or population**: individuals eligible for a trial **Settings**: any **Intervention**: PIL developed with feedback from users **Comparison**: usual PIL						
Outcomes	Illustrative comparative risks* (95% CI)		Relative effect (95% CI)	No. of participants (studies)	Certainty of the evidence (GRADE)	Cost‐effectiveness, retention, and EDI summary
	Effect with usual PIL	Effect with PIL developed with feedback from users				
**Number recruited**	401 out of 8358	441 out of 8405	**RD 0%** (0% to 1%)	16,763 (2 studies)	⊕⊕⊕⊝ **Moderate**^c^	**Cost:** no data reported **Retention:** no data reported **EDI:** both studies were conducted in one high‐income country (UK). One study reported demographic data: participant mean age was reported as about 78 years. About 45% of participants were male. There was no information on ethnicity or socioeconomic status of participants.
**Proportion recruited**	**As measured**^a^		**RR 1.09** (0.96 to 1.25)			
	**5 per 100**	**5 per 100** (5 to 6)				
	**Low**^b^					
	**10 per 100**	**11 per 100** (10 to 13)				
	**Moderate**^b^					
	**30 per 100**	**33 per 100** (29 to 38)				
	**High**^b^					
	**50 per 100**	**55 per 100** (48 to 63)				
*The basis for the **assumed risk** (e.g. the median control group risk across studies) is provided in the footnotes. The **effect with aPIL developed with feedback from users** (and its 95% CI) is based on the assumed risk in the comparison group (usual PIL) and the **relative effect** of the intervention (and its 95% CI). **CI**: confidence interval; **EDI**: equity, diversity, and inclusion; **RD**: risk difference; **RR**: risk ratio						
**GRADE Working Group grades of evidence** **High certainty:** we are very confident that the true effect lies close to that of the estimate of the effect. **Moderate certainty:** we are moderately confident in the effect estimate; the true effect is likely to be close to the estimate of the effect, but there is a possibility that it is substantially different. **Low certainty:** our confidence in the effect estimate is limited; the true effect may be substantially different from the estimate of the effect. **Very low certainty:** we have very little confidence in the effect estimate; the true effect is likely to be substantially different from the estimate of effect.						

^a^This is the baseline recruitment measured in the included studies. ^b^We selected the low, moderate, and high illustrative recruitment levels of 10%, 30%, and 50% based on our prior experience with trial recruitment. ^c^We downgraded the certainty of the evidence by one level for indirectness: Chen 2011 measured entry to pre‐randomisation phase, not recruitment.

**Summary of findings 8:** Providing information by video versus by standard means alone

**Video information versus standard information alone**						
**Patient or population**: individuals eligible for trial **Settings**: any **Intervention**: video information **Comparison**: standard information (mixed but not including video)						
Outcomes	Illustrative comparative risks* (95% CI)		Relative effect (95% CI)	No. of participants (studies)	Certainty of the evidence (GRADE)	**Cost‐effectiveness, retention, and EDI summary**
	**Effect with standard information**	Effect with video information				
**Number recruited**	237 out of 830	212 out of 804	**RD −4%** (−23% to 15%)	1634 (5 studies)	⊕⊝⊝⊝ **Very low**^c,d,e^	**Cost:** data reported from one study indicated that providing information by video is both more costly (GBP 41.58 more) and less effective (−2% reduction). **Retention:** no data reported **EDI:** all studies were conducted in high‐income countries (UK and USA). Three studies reported participants' mean age to be 34 to 70 years, with 20% to 100% female participants. Two studies reported race, one reported ethnicity. Two studies included 30% to 40% African Americans. One study included 6% Asian participants, 8% Black/African/Caribbean participants, and 6% participants from other groups, alongside White participants. All socioeconomic levels were represented; 20% to 50% were from the most disadvantaged communities.
**Proportion recruited**	**As measured**^a^		**RR 0.92** (0.45 to 1.90)			
	**29 per 100**	**26 per 100** (13 to 55)				
	**Low**^b^					
	**10 per 100**	**9 per 100** (5 to 19)				
	**Moderate**^b^					
	**30 per 100**	**28 per 100** (14 to 57)				
	**High**^b^					
	**50 per 100**	**46 per 100** (23 to 95)				
*The basis for the **assumed risk** (e.g. the median control group risk across studies) is provided in the footnotes. The **effect with thevideo information** (and its 95% CI) is based on the assumed risk in the comparison group (standard information) and the **relative effect** of the intervention (and its 95% CI). **CI**: confidence interval; **EDI**: equity, diversity and inclusion; **GBP**: pounds sterling; **RD**: risk difference; **RR**: risk ratio						
**GRADE Working Group grades of evidence** **High certainty:** we are very confident that the true effect lies close to that of the estimate of the effect. **Moderate certainty:** we are moderately confident in the effect estimate; the true effect is likely to be close to the estimate of the effect, but there is a possibility that it is substantially different. **Low certainty:** our confidence in the effect estimate is limited; the true effect may be substantially different from the estimate of the effect. **Very low certainty:** we have very little confidence in the effect estimate; the true effect is likely to be substantially different from the estimate of effect.						

^a^This is the baseline recruitment measured in the included studies. ^b^We selected the low, moderate, and high illustrative recruitment levels of 10%, 30%, and 50% based on our prior experience with trial recruitment. ^c^We downgraded the certainty of the evidence by one level for study limitations: Du 2008, Du 2009, and Rodgers 2019 were at unclear risk of bias; Mattock 2020 was at high risk of bias. ^d^We downgraded the certainty of the evidence by one level for inconsistency. Most studies suggested little or no difference in recruitment due to the intervention. However, point estimates in Hutchison 2007, Mattock 2020, and Rodgers 2019 favoured control, while point estimates in Du 2008 and Du 2009 favoured the intervention. ^e^We downgraded the certainty of the evidence by one level for imprecision, with a wide confidence interval crossing the line of no effect.

**Summary of findings 9:** Monetary incentive versus no incentive

**Monetary incentive versus no incentive**						
**Patient or population**: individuals eligible for a trial **Settings**: any **Intervention**: monetary incentive **Comparison**: no incentive						
Outcomes	Illustrative comparative risks* (95% CI)		Relative effect (95% CI)	No. of participants (studies)	Certainty of the evidence (GRADE)	**Cost‐effectiveness, retention, and EDI summary**
	Effect with no incentive	Effect with monetary incentive				
**Number recruited**	235 out of 2351	522 out of 2731	**RD 5%** (1% to 9%)	5082 (12 studies)	⊕⊕⊝⊝ **Low**^c,d^	**Cost:** the estimated ICER for monetary incentives versus no incentive was GBP 3052.96, with an incremental cost of GBP 152.65 and an incremental effectiveness of 5%. **Retention:** no data reported **EDI:** all studies were conducted in high‐income countries (Canada, the UK, and USA). In nine studies, participants had a mean age of 47 to 66 years, with 40% to 58% male. Ethnicities were mixed: 34% Black/African American and 52% White. Participants spanned all income and deprivation levels, with 27% from the most deprived areas. Urban/rural status was not reported.
**Proportion recruited**	**As measured**^a^		**RR 1.48** (1.00 to 2.19)			
	**10 per 100**	**15 per 100** (10 to 22)				
	**Low**^b^					
	**10 per 100**	**15 per 100** (10 to 22)				
	**Moderate**^b^					
	**30 per 100**	**44 per 100** (30 to 66)				
	**High**^b^					
	**50 per 100**	**74 per 100** (50 to 100)				
*The basis for the **assumed risk** (e.g. the median control group risk across studies) is provided in the footnotes. The **effect with afinancial incentive** (and its 95% CI) is based on the assumed risk in the comparison group (no incentive) and the **relative effect** of the intervention (and its 95% CI). **CI**: confidence interval; **EDI**: equity, diversity and inclusion; **GBP:** pounds sterling; **ICER:** incremental cost‐effectiveness ratio; **RD**: risk difference; **RR**: risk ratio						
**GRADE Working Group grades of evidence** **High certainty:** we are very confident that the true effect lies close to that of the estimate of the effect. **Moderate certainty:** we are moderately confident in the effect estimate; the true effect is likely to be close to the estimate of the effect, but there is a possibility that it is substantially different. **Low certainty:** our confidence in the effect estimate is limited; the true effect may be substantially different from the estimate of the effect. **Very low certainty:** we have very little confidence in the effect estimate; the true effect is likely to be substantially different from the estimate of effect.						

^a^This is the baseline recruitment measured in the included studies. ^b^We selected the low, moderate, and high illustrative recruitment levels of 10%, 30%, and 50% based on our prior experience with trial recruitment. ^c^We downgraded the certainty of the evidence by one level for study limitations: Fairhurst 2022, Halpern 2021a, and Halpern 2021b were at high risk of bias. ^d^We downgraded the certainty of the evidence by one level for inconsistency (substantial heterogeneity; I^2^ = 73%).

**Summary of findings 10:** Information provided over telephone versus information provided face‐to‐face

**Information provided over telephone versus information provided face‐to‐face**						
**Patient or population**: individuals eligible for a trial **Settings**: any **Intervention**: information provided over the telephone **Comparison**: information provided face‐to‐face						
Outcomes	Illustrative effects* (95% CI)		Relative effect (95% CI)	No. of participants (studies)	Certainty of the evidence (GRADE)	**Cost‐effectiveness, retention, and EDI summary**
	**Effect with face‐to‐face information**	Effect with information provided over telephone				
**Number recruited**	100 out of 126	86 out of 123	**RD −9%** (−18% to 1%)	249 participants (2 studies)	⊕⊕⊕⊝ **Moderate**^c^	**Cost:** no data reported **Retention:** no data reported **EDI:** both studies were conducted in high‐income countries (Denmark and USA). Most participants were aged 30 to 60 years. One study reported an even male‐female split; another included only women. In one study, 55% had a master’s degree or higher, while 11% had only basic schooling. Urban/rural status was not reported.
**Proportion recruited**	**As measured**^a^		**RR 0.90** (0.80 to 1.02)			
	**79 per 100**	**71 per 100** (63 to 81)				
	**Low**^b^					
	**10 per 100**	**9 per 100** (8 to 10)				
	**Moderate**^b^					
	**30 per 100**	**27 per 100** (24 to 31)				
	**High**^b^					
	**50 per 100**	**45 per 100** (40 to 51)				
*The basis for the **assumed risk** (e.g. the median control group risk across studies) is provided in the footnotes. The **effect for information provided over the telephone** (and its 95% CI) is based on the assumed risk in the comparison group (information provided face‐to‐face) and the **relative effect** of the intervention (and its 95% CI). **CI**: confidence interval; **EDI**: equity, diversity and inclusion; **RCT**: randomised controlled trial; **RD**: risk difference; **RR**: risk ratio						
**GRADE Working Group grades of evidence** **High certainty:** we are very confident that the true effect lies close to that of the estimate of the effect. **Moderate certainty:** we are moderately confident in the effect estimate; the true effect is likely to be close to the estimate of the effect, but there is a possibility that it is substantially different. **Low certainty:** our confidence in the effect estimate is limited; the true effect may be substantially different from the estimate of the effect. **Very low certainty:** we have very little confidence in the effect estimate; the true effect is likely to be substantially different from the estimate of effect.						

^a^This is the baseline recruitment measured in the included studies. ^b^We selected the low, moderate, and high illustrative recruitment levels of 10%, 30%, and 50% based on our prior experience with trial recruitment. ^c^We downgraded the certainty of the evidence by one level for imprecision (wide CI crossing the line of no effect).

**Summary of findings 11:** Multimedia participant information leaflet (PIL) (viewed in person) plus paper PIL versus paper PIL alone

**Multimedia participant information leaflet (PIL) (viewed in person) plus paper PIL versus paper PIL alone**						
**Patient or population**: individuals eligible for a trial **Settings**: any **Intervention**: multimedia information (MMI) PIL (viewed in person) plus paper PIL **Comparison**: paper PIL only						
Outcomes	Illustrative effects* (95% CI)		Relative effect (95% CI)	No. of participants (studies)	Certainty of the evidence (GRADE)	Cost‐effectiveness and EDI summary
	**Effect with paper PIL only**	Effect with multimedia information + paper PIL				
**Number recruited**	7 out of 9	13 out of 14	**RD 17%** (−89% to 123%)	23 (3 studies)	⊕⊝⊝⊝ **Very low**^c,d^	**Cost:** no data reported **EDI:** all studies were conducted in a high‐income country (UK), and involved non‐white male and female children aged between six and 13 years, living mainly in disadvantaged areas.
**Proportion recruited**	**As measured**^a^		**RR 1.02** (0.72 to 1.44)			
	**78 per 100**	**79 per 100** (56 to 100)				
	**Low**^b^					
	**10 per 100**	**10 per 100** (7 to 14)				
	**Moderate**^b^					
	**30 per 100**	**31 per 100** (22 to 43)				
	**High**^b^					
	**50 per 100**	**51 per 100** (36 to 72)				
**Number retained**	5 out of 9	11 out of 14	**RD 28%** (−79% to 136%)	23 (3 studies)	⊕⊝⊝⊝**Very low**^c,e^	
*The basis for the **assumed risk** (e.g. the median control group risk across studies) is provided in the footnotes. The **effect for the multimedia information participant information leaflet (PIL) (viewed in person) plus paper PIL** (and its 95% CI) is based on the assumed risk in the comparison group (paper PIL only) and the **relative effect** of the intervention (and its 95% CI). **CI**: confidence interval; **EDI**: equity, diversity and inclusion; **RD**: risk difference; **RR**: risk ratio						
**GRADE Working Group grades of evidence** **High certainty:** we are very confident that the true effect lies close to that of the estimate of the effect. **Moderate certainty:** we are moderately confident in the effect estimate; the true effect is likely to be close to the estimate of the effect, but there is a possibility that it is substantially different. **Low certainty:** our confidence in the effect estimate is limited; the true effect may be substantially different from the estimate of the effect. **Very low certainty:** we have very little confidence in the effect estimate; the true effect is likely to be substantially different from the estimate of effect.						

^a^This is the baseline recruitment measured in the included studies. ^b^We selected the low, moderate, and high illustrative recruitment levels of 10%, 30%, and 50% based on our prior experience with trial recruitment. ^c^We downgraded the certainty of the evidence by two levels for imprecision (extremely wide CI crossing the line of no effect; RD = 0). ^d^We downgraded the certainty of the evidence by one level for inconsistency (substantial heterogeneity; I^2^ = 64%). ^e^We downgraded the certainty of the evidence by one level for inconsistency (substantial heterogeneity; I^2^ = 57%).

**Summary of findings 12:** Multimedia participant information leaflet (PIL) (viewed in person) versus paper PIL

**Multimedia information participant information leaflet (PIL) (viewed in person) versus paper PIL**						
**Patient or population**: individuals eligible for a trial **Settings**: any **Intervention**: multimedia information PIL (viewed in person) **Comparison**: paper PIL						
Outcomes	Illustrative effects* (95% CI)		Relative effect (95% CI)	No.of participants (studies)	Certainty of the evidence (GRADE)	**Cost‐effectiveness and EDI summary**
	**Effect with paper PIL**	Effect with multimedia information PIL				
**Number recruited**	489 out of 736	486 out of 692	**RD 4%** (−1% to 8%)	1428 (3 studies)	⊕⊕⊕⊝ **Moderate**^c^	**Cost:** no data reported **EDI:** all studies were conducted in a high‐income country (UK). Mean age range: 6 to 9 years. Female participants made up 39.3% to 76.2%. Two studies (BALANCE and FORCE) reported ethnicity. BALANCE: 42.9% White, 47.6% non‐White, 4.8% missing. FORCE: 80.2% White, 10.1% Asian, 5% Black, 2% mixed, 2.2% other, 0.5% not stated. Median deprivation scores (3.5 to 4.5) indicated moderate to substantial deprivation. Urban/rural status was not reported.
**Proportion recruited**	**As measured**^a^		**RR 1.05** (0.98 to 1.13)			
	**66 per 100**	**70 per 100** (65 to 75)				
	**Low**^b^					
	**10 per 100**	**11 per 100** (10 to 11)				
	**Moderate**^b^					
	**30 per 100**	**32 per 100** (29 to 34)				
	**High**^b^					
	**50 per 100**	**53 per 100** (49 to 57)				
**Number retained**	484 out of 736	483 out of 692	**RD 22%** (−51% to 96%)	1428 (3 studies)	⊕⊝⊝⊝ **Very low**^d,e^	
*The basis for the **assumed risk** (e.g. the median control group risk across studies) is provided in the footnotes. The **effect for the multimedia information participant information leaflet (viewed in person)** (and its 95% CI) is based on the assumed risk in the comparison group (paper PIL) and the **relative effect** of the intervention (and its 95% CI). **CI**: confidence interval; **EDI**: equity, diversity and inclusion; **RD**: risk difference; **RR**: risk ratio						
**GRADE Working Group grades of evidence** **High certainty:** we are very confident that the true effect lies close to that of the estimate of the effect. **Moderate certainty:** we are moderately confident in the effect estimate; the true effect is likely to be close to the estimate of the effect, but there is a possibility that it is substantially different. **Low certainty:** our confidence in the effect estimate is limited; the true effect may be substantially different from the estimate of the effect. **Very low certainty:** we have very little confidence in the effect estimate; the true effect is likely to be substantially different from the estimate of effect.						

^a^This is the baseline recruitment measured in the included studies. ^b^We selected the low, moderate, and high illustrative recruitment levels of 10%, 30%, and 50% based on our prior experience with trial recruitment. ^c^We downgraded the certainty of the evidence by one level for imprecision. ^d^We downgraded the certainty of the evidence by one level for inconsistency (substantial heterogeneity; I^2^ = 64%). ^e^We downgraded the certainty of the evidence by two levels for imprecision (extremely wide CI crossing the line of no effect; RD = 0).

**Summary of findings 13:** Multimedia information (provided as link/QR code) versus paper participant information leaflet (PIL)

**Multimedia information (provided as link/QR code) versus paper participant information leaflet (PIL)**						
**Patient or population**: individuals eligible for a trial **Settings**: any **Intervention**: multimedia information (provided as link/QR code) **Comparison**: paper PIL						
Outcomes	Illustrative effects* (95% CI)		Relative effect (95% CI)	No. of participants (studies)	Certainty of the evidence (GRADE)	**Cost‐effectiveness and EDI summary**
	**Effect with paper PIL**	Effect with multimedia information				
**Number recruited**	52 out of 112	70 out of 117	**RD 13%** (1% to 26%)	229 (2 studies)	⊕⊕⊕⊝ **Moderate**^c^	**Cost:** no data reported **EDI:** all studies were conducted in a high‐income country (UK). Participants’ mean age was 6 years and median age 12 years. Between 62.5% and 76.2% of participants were female. Median deprivation scores ranged from 3.5 to 4.5, indicating moderate to substantial deprivation. One study reported ethnicity: 42.9% White, 47.6% non‐White, and 4.8% missing. Urban/rural status was not reported.
**Proportion recruited**	**As measured**^a^		**RR 1.29** (1.01 to 1.65)			
	**46 per 100**	**60 per 100** (46 to 76)				
	**Low**^b^					
	**10 per 100**	**13 per 100** (10 to 17)				
	**Moderate**^b^					
	**30 per 100**	**39 per 100** (30 to 50)				
	**High**^b^					
	**50 per 100**	**65 per 100** (51 to 83)				
**Number retained**	47 out of 112	64 out of 117	**RD 13%** (0% to 26%)	229 (2 studies)	⊕⊕⊕⊝ **Moderate**^c^	
*The basis for the **assumed risk** (e.g. the median control group risk across studies) is provided in the footnotes. The **effect for the multimedia information (provided as link/QR code)** (and its 95% CI) is based on the assumed risk in the comparison group (paper participant information leaflet (PIL) and the **relative effect** of the intervention (and its 95% CI). **CI**: confidence interval; **EDI**: equity, diversity and inclusion; **RCT**: randomised controlled trial; **RD**: risk difference; **RR**: risk ratio						
**GRADE Working Group grades of evidence** **High certainty:** we are very confident that the true effect lies close to that of the estimate of the effect. **Moderate certainty:** we are moderately confident in the effect estimate; the true effect is likely to be close to the estimate of the effect, but there is a possibility that it is substantially different. **Low certainty:** our confidence in the effect estimate is limited; the true effect may be substantially different from the estimate of the effect. **Very low certainty:** we have very little confidence in the effect estimate; the true effect is likely to be substantially different from the estimate of effect.						

^a^This is the baseline recruitment measured in the included studies. ^b^We selected the low, moderate, and high illustrative recruitment levels of 10%, 30%, and 50% based on our prior experience with trial recruitment. ^c^We downgraded the certainty of the evidence by one level for imprecision (wide CIs).

**Summary of findings 14:** Monetary incentives (change in value)

**Monetary incentives (change in value)**						
**Patient or population**: individuals eligible for a trial **Settings**: any **Intervention**: higher‐value monetary incentives **Comparison**: lower‐value monetary incentives						
Outcomes	Illustrative effects* (95% CI)		Relative effect (95% CI)	No. of participants (studies)	Certainty of the evidence (GRADE)	**Cost‐effectiveness, retention, and EDI summary**
	**Effect with lower incentive value**	Effect with higher incentive value				
**Number recruited**	180 out of 429	196 out of 435	**RD 3%** (−13% to 19%)	864 (2 studies)	⊕⊝⊝⊝ **Very low**^c, d,e^	**Cost:** the estimated ICER of changing financial incentives was GBP 8214.83, with an incremental effectiveness of 3%. **Retention:** no data reported **EDI:** both studies were conducted in a high‐income country (USA). The result applies to middle‐aged males and females with a range of ethnicities and socioeconomic statuses living in the USA. Urban/rural status was not reported.
**Proportion recruited**	**As measured**^a^		**RR 1.08** (0.74 to 1.57)			
	**42 per 100**	**45 per 100** (31 to 66)				
	**Low**^b^					
	**10 per 100**	**11 per 100** (7 to 16)				
	**Moderate**^b^					
	**30 per 100**	**32 per 100** (22 to 47)				
	**High**^b^					
	**50 per 100**	**54 per 100** (37 to 79)				
*The basis for the **assumed risk** (e.g. the median control group risk across studies) is provided in the footnotes. The **effect for the higher‐value financial incentives** (and its 95% CI) is based on the assumed risk in the comparison group (lower‐value financial incentives) and the **relative effect** of the intervention (and its 95% CI). **CI**: confidence interval; **EDI**: equity, diversity and inclusion; **GBP:** pounds sterling; **RD**: risk difference; **RR**: risk ratio						
**GRADE Working Group grades of evidence** **High certainty:** we are very confident that the true effect lies close to that of the estimate of the effect. **Moderate certainty:** we are moderately confident in the effect estimate; the true effect is likely to be close to the estimate of the effect, but there is a possibility that it is substantially different. **Low certainty:** our confidence in the effect estimate is limited; the true effect may be substantially different from the estimate of the effect. **Very low certainty:** we have very little confidence in the effect estimate; the true effect is likely to be substantially different from the estimate of effect.						

^a^This is the baseline recruitment measured in the included studies. ^b^We selected the low, moderate, and high illustrative recruitment levels of 10%, 30%, and 50% based on our prior experience with trial recruitment. ^c^We downgraded the certainty of the evidence by one level for study limitations: both studies were at an overall high risk of bias. ^d^We downgraded the certainty of the evidence by one level for imprecision (very wide CI which crossed the line of no effect; RD = 0). ^e^We downgraded the certainty of the evidence by one level for inconsistency (substantial heterogeneity; I^2^ = 83%).

**Summary of findings 15:** Non‐monetary incentive (branded pen versus no pen)

**Non‐monetary incentive (branded pen versus no pen)**						
**Patient or population**: individuals eligible for a trial **Settings**: any **Intervention**: branded pen **Comparison**: no pen						
Outcomes	Illustrative effects* (95% CI)		Relative effect (95% CI)	No. of participants (studies)	Certainty of the evidence (GRADE)	Cost‐effectiveness and EDI summary
	**Effect with no pen**	Effect with pen				
**Number recruited**	238 out of 5140	188 out of 4500	**RD 0%** (−1% to 1%)	9640 (3 studies)	⊕⊕⊕⊝ **Moderate**^c^	**Cost:** trial branded pens resulted in a small increase in cost (incremental cost = GBP 0.45) with no difference in recruitment. **EDI:** one study reported age (mean 46.1, range 18 to 70) and sex (83% female). Most participants were British (87.1%), with small numbers from other White (5.3%), Asian (3.7%), mixed (1.2%), Black (0.6%), and Arab (0.6%) groups. No Gypsy/Traveller or African participants. Conducted in the UK, across urban and rural areas.
**Proportion recruited**	**As measured**^a^		**RR 0.91** (0.74 to 1.11)			
	**5 per 100**	**4 per 100** (4 to 6)				
	**Low**^b^					
	**10 per 100**	**9 per 100** (7 to 11)				
	**Moderate**^b^					
	**30 per 100**	**27 per 100** (22 to 33)				
	**High**^b^					
	**50 per 100**	**46 per 100** (37 to 56)				
**Number retained**	49 out of 1242	27 out of 620	**RD 0%** (−2% to 2%)	1862 (1 study)	⊕⊕⊕⊝ **Moderate**^d^	
*The basis for the **assumed risk** (e.g. the median control group risk across studies) is provided in the footnotes. The **effect for the branded pen** (and its 95% confidence interval) is based on the assumed risk in the comparison group (no pen) and the **relative effect** of the intervention (and its 95% CI). **CI**: confidence interval; **EDI**: equity, diversity and inclusion; **GBP:** pounds sterling; **RD**: risk difference; **RR**: risk ratio						
GRADE Working Group levels of evidence **High certainty:** we are very confident that the true effect lies close to that of the estimate of the effect. **Moderate certainty:** we are moderately confident in the effect estimate; the true effect is likely to be close to the estimate of the effect, but there is a possibility that it is substantially different. **Low certainty:** our confidence in the effect estimate is limited; the true effect may be substantially different from the estimate of the effect. **Very low certainty:** we have very little confidence in the effect estimate; the true effect is likely to be substantially different from the estimate of effect.						

^a^This is the baseline recruitment measured in the included studies. ^b^We selected the low, moderate, and high illustrative recruitment levels of 10%, 30%, and 50% based on our prior experience with trial recruitment. ^c^We downgraded the certainty of the evidence by one level for study limitations: the risk of bias was high for Fairhurst 2022 and was unclear for White 2023. ^d^We downgraded the certainty of the evidence by one level for imprecision (single study).

## Background

All trials need to recruit participants, but this is often a challenge. Poor recruitment can lead to an underpowered study, which may report clinically relevant effects to be statistically non‐significant. A non‐significant finding increases the risk that an effective intervention will be abandoned before its true value is established, or that there will be a delay in demonstrating this value while more trials or meta‐analyses are done. Underpowered trials also raise an ethical problem: researchers have exposed participants to an intervention with uncertain benefit but may still be unable to determine whether the intervention does more good than harm on completion of the trial. Poor recruitment can also lead to trial extension, higher costs, or trial abandonment.

Investigations of recruitment differ in their estimates of the proportion of studies that achieve their recruitment targets, but it is likely that around half meet their target [1, 2, 3, 4, 5]. For example, McDonald and colleagues found that only 38 (31%) of 114 trials achieved their original recruitment target and 65 (53%) were extended [3]. A more recent replication of this work found that the number of trials meeting recruitment targets had increased to around 63% (245/388), but 32% (79/245) of these trials required an extension to their recruitment period to meet the target, meaning approximately 43% of trials recruit to target without an extension [1]. An additional 22% (86/388) of trials recruited to within 80% of their final recruitment target, with 36% (31/86) of these requiring an extension to their recruitment period [1].

Poor recruitment can have significant public health implications [6]. Evidence from the dexamethasone arm of the COVID‐19 RECOVERY trial suggests that delays in trial completion – such as those caused by slow recruitment – led to avoidable deaths in the UK [7]. Faster recruitment could lead to earlier trial completion, and if the intervention proves effective, earlier access could benefit more people. Had RECOVERY recruited 50% of eligible participants rather than the typical 15%, nearly 3000 additional lives could have been saved through earlier access to dexamethasone, which was shown to be an effective treatment. The economic implications may also be substantial: increasing recruitment from 15% to 50% at all sites could have delivered an incremental net benefit of 13.9 million pounds sterling (GBP) to the UK from a healthcare system perspective [8]. These findings highlight the vital importance of efficient trial processes – not only for saving lives but also for maximising the health and economic value of research.

This systematic review updates our previous reviews [9, 10, 11]. In addition to updating the literature search, we adjusted the eligibility criteria by excluding quasi‐randomised and hypothetical trials (i.e. where people are asked to imagine whether they would take part in a trial that is not really happening, rather than being invited to a real trial). We also included three new outcomes: cost‐effectiveness of recruitment strategies; impact of recruitment strategies on participant retention; and equity, diversity, and inclusion (EDI) characteristics of the participants.

### Description of the methods being investigated

Strategies to improve participant recruitment aim to increase the number of people who consent and enrol in a trial. These interventions can be implemented at three levels: the participant, the recruiter, or the overall trial management. Methods include changes to how potential participants are contacted or invited to join, strategies directed at recruiters (such as training or prompts), modifications to trial design or management (e.g. altering consent procedures), and changes to the information or materials used to present trial details.

## Objectives

Primary: to quantify the effects of strategies to improve recruitment of participants to RCTs.

Secondary: to evaluate recruitment strategies' cost‐effectiveness and impact on retention, and the equity, diversity, and inclusion (EDI) characteristics of recruited participants.

## Methods

We followed guidance in the Methodological Expectations for Cochrane Intervention Reviews (MECIR) manual when undertaking the review [12], and PRISMA 2020 for reporting the review [13].

### Differences between protocol and review

Since the 2018 update, we amended the protocol in several ways to enhance methodological rigour and address emerging priorities in trial methodology research. We narrowed the inclusion criteria to include only randomised controlled trials. We excluded hypothetical and quasi‐randomised trials to ensure the meta‐analysis reflects high‐certainty, real‐world evidence. We also introduced three new secondary outcomes to provide a more comprehensive evaluation of recruitment strategies: retention rates – to assess downstream effects on trial completion; cost‐effectiveness – to provide practical resource implications; and equality, diversity, and inclusion (EDI) characteristics – to identify representation gaps.

### Criteria for considering studies for this review

#### Types of studies

We included randomised Studies Within A Trial (SWATs), including individually randomised and cluster‐randomised designs, that evaluated strategies to improve participant recruitment to randomised trials.

#### Types of data

We included any intervention that aimed to improve participant recruitment to a randomised trial, except incentives or disincentives for clinicians to recruit participants, which is covered by another Cochrane review [14]. We did not impose any restrictions on the number or types of participants included or the study setting. The interventions being studied could be directed at potential participants (e.g. patients being randomised to a trial), collaborators (e.g. clinicians recruiting patients for a trial), or others (e.g. research ethics committees). Examples of eligible interventions include: letters introducing the trial signed by influential people; alternative methods of providing information about the trial to potential participants; additional training for collaborators; monetary incentives for participants; telephone follow‐up after expressions of interest; and modifications to the trial design (e.g. preference designs).

#### Types of methods

We included randomised Studies Within A Trial (SWATs) of strategies to improve recruitment to randomised trials. These were not limited to healthcare trials since strategies that work in other fields (e.g. education, housing) could be applicable to healthcare settings.

We excluded quasi‐randomised SWATs and recruitment strategies evaluated within quasi‐randomised host trials because these designs are at high risk of bias and contribute low‐certainty evidence. We also excluded studies conducted in hypothetical trial settings because participant decisions in hypothetical scenarios may not reflect real‐world decisions about trial participation. Additionally, given the growing body of evidence from evaluations embedded in real trials, we no longer consider their inclusion justified. We excluded studies focused on questionnaire response rates, trial retention, or incentives and disincentives for clinicians to recruit participants because these topics are addressed in separate Cochrane methodology reviews [14, 15, 16, 17, 18].

#### Types of outcome measures

##### Primary outcomes

Proportion of eligible individuals or centres recruited (i.e. randomised to the trial).

##### Secondary outcomes

We added the following outcomes for this update. Previous versions of this review did not include any secondary outcomes.

- Cost‐effectiveness of recruitment strategies, measured using the incremental cost‐effectiveness ratio (ICER), defined as the incremental cost per additional participant recruited. We defined incremental cost as the additional cost of one strategy compared with another, and the ICER as the additional cost per additional participant recruited.
- Impact of the recruitment strategy on retention rates. We defined retention as the proportion of participants randomised to each SWAT arm who were retained in the host trial (e.g. attended follow‐up visits or completed questionnaires). Where available, we used retention at the host trial's primary outcome time point; otherwise, we used the first reported retention measure.
- Participant equity, diversity, and inclusion (EDI) characteristics, as reported by trialists, to inform equity‐related assessments of the populations to whom the evidence applies.

### Search methods for identification of studies

#### Electronic searches

To update the review, we re‐screened the 68 previously included studies and searched the following electronic databases, with no language restrictions, for eligible studies published from 1 January 2015 onwards.

We searched the following databases on 16 February 2023:

- MEDLINE and MEDLINE In‐Process (Ovid);
- Embase (Ovid);
- Science Citation Index and Social Sciences Citation Index (Web of Science).

We added the following databases for this update:

- Applied Social Sciences Index and Abstracts (ASSIA) (searched to 31 December 2023);
- CINAHL Plus (Cumulative Index to Nursing and Allied Health Literature; EBSCO) (searched to 31 December 2023);
- ERIC (EBSCO) (searched to 31 December 2023);
- Online Resource for Research in Clinical triAls (ORRCA) (searched to 31 December 2019, when it was last updated);
- PsycINFO (EBSCO) (searched to February Week 2 2023).

To ensure global coverage, we also consulted regional sources such as LILACS (Latin American and Caribbean Health Sciences Literature). We did not search the Cochrane Methodology Review (CMR) Group Specialised Register as it has not been updated since July 2012. However, we included eligible papers identified from the CMR Group Specialised Register for the previous review (last search date 11 February 2015) in this review update. Supplementary material 1 details the full search strategies for all databases.

#### Searching other resources

To identify unpublished and ongoing recruitment SWATs [19], we issued a call for information via social media and email. On 23 March 2023, we posted on X (formerly Twitter), inviting trial methodology researchers who had conducted randomised recruitment SWATs to share their findings. On 27 March 2023, we circulated a similar request through the Trial Forge SWAT Network, the UK Trial Managers’ Network, the UK Clinical Research Collaboration (CRC) Registered Trials Units Network, the Medical Research Council and National Institute for Health and Care Research (MRC‐NIHR) Trial Methodology Partnership, and the Health Research Board Trial Methodology Network in Ireland.

We implemented a study‐integrity audit to identify retractions, expressions of concern, or corrigenda. We checked the digital object identifiers of all eligible studies against the Crossref/CrossMark API and the Retraction Watch database, and used this information to ensure that the results reflected the most up‐to‐date, corrected versions of each included study.

### Data collection and analysis

We prepared a revised protocol for this updated review, including it as Supplementary material 6 to make it available alongside this review in the Cochrane Library.

#### Selection of studies

We downloaded the search results to Endnote reference management software for deduplication and then uploaded the records to Covidence to enable screening and data extraction [20]. Working independently, two review authors screened the titles and abstracts of all records retrieved from searches of the electronic databases. We resolved any disagreements by involving a third author. We retrieved the full texts of studies that appeared to meet the inclusion criteria. Two review authors independently assessed all potentially eligible studies to determine if they met the inclusion criteria. We resolved any disagreements through discussion or by involving a third author.

Authors Adwoa Parker, Shaun Treweek, Frank Sullivan, Elizabeth Colement, Mike Clarke, Caroline Fairhurst, and Catherine Hewitt were lead authors or co‐authors of a number of studies included in this review. They were not involved in study eligibility decisions, data extraction, risk of bias assessment, or GRADE assessments for any included studies they authored.

#### Data extraction and management

Working independently, two review authors extracted data from each included study using a data extraction form designed for the purpose. We resolved any differences in data extraction through discussion. We extracted data on:

- recruitment strategy evaluated;
- country in which the study was carried out;
- population;
- study setting;
- host trial;
- randomisation method;
- number and proportion of participants in the SWAT intervention and comparator groups;
- participant characteristics, including age, sex, gender, ethnicity, socioeconomic status, and geographic location, using the PRO EDI tool [21];
- costs;
- proportion of participants or clusters retained.

For the cost‐effectiveness analysis, we extracted cost data from newly included studies, where available. We also extracted cost data from previously included studies identified in a systematic review of economic evaluations conducted alongside SWATs [22]. For each study, we recorded the costs of the intervention and comparator strategies, and any reported incremental costs. Where incremental costs were not reported, we estimated them by attributing relevant unit costs to each strategy and calculating the difference.

#### Assessment of risk of bias in included studies

We assessed risk of bias in the included studies using the original Cochrane risk of bias tool [23], for consistency with previous versions of the review [11]. We adapted the assessment to reflect the methodological nature of the interventions evaluated. Working independently, two review authors performed a blinded risk of bias assessment of each study before comparing assessments. We resolved any discrepancies through discussion. We assessed random sequence generation, allocation concealment, blinding of participants and personnel, blinding of outcome assessment, incomplete outcome data, selective reporting, and other potential sources of bias. We judged each domain as low, high, or unclear risk of bias. We considered a study to be at low overall risk of bias if all domains were judged to be at low risk of bias, at high overall risk of bias if one or more domains were judged to be at high risk of bias, and otherwise at unclear risk of bias.

We did not exclude studies judged to be at high overall risk of bias from the meta‐analyses (Supplementary material 4). However, because we have lower confidence in these studies' evidence, we do not discuss them individually in the Results or Discussion, except where their data contribute to a meta‐analysis and can be considered alongside evidence from studies at lower risk. We suggest that readers should not draw conclusions about intervention effects solely from the results of studies at high overall risk of bias. Rather, these studies may help identify interventions that warrant further evaluation using more rigorous methods.

Methodological quality data for all included studies are presented in the risk of bias graph (Figure).

**1:**
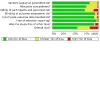
Risk of bias graph: review authors' judgements about each risk of bias item presented as percentages across all included studies

#### Measures of the effect of the methods

We calculated risk differences (RDs) with 95% confidence intervals (CIs) to estimate the effects of recruitment strategies on recruitment. In accordance with guidance in the *Cochrane Handbook for Systematic Reviews of Interventions*, we estimated RDs for single‐study comparisons using fixed‐effect models with Mantel‐Haenszel weighting [24]. For comparisons involving multiple studies, we conducted random‐effects meta‐analyses using inverse‐variance weighting [24]. In the summary of findings tables, we report the effect of recruitment strategies using both risk ratios (RRs) and risk differences (RDs) with 95% CIs, to provide a relative effect measure alongside the absolute impact of the strategies.

#### Unit of analysis issues

For recruitment SWATs that randomised individuals and clusters, the unit of analysis was the participant. We included cluster‐randomised trials in meta‐analyses only when sufficient data were available to adjust for clustering (e.g. intra‐cluster correlation coefficients or cluster‐adjusted estimates). When cluster‐adjusted risk differences (RDs) or risk ratios (RRs) were not available, we used odds ratios (ORs) as the summary effect measure.

#### Dealing with missing data

We contacted study authors to request missing data needed for the risk of bias assessment, the number of participants randomised to each group, and the number of participants recruited.

#### Assessment of heterogeneity

We sought statistical evidence of heterogeneity of results using the Chi^2^ test for heterogeneity, and we quantified the degree of heterogeneity using the I^2^ statistic [25]. The I² is independent of the number of studies and reflects variation based on the treatment effect. Where we detected substantial heterogeneity, we informally investigated possible explanations and summarised the data using a random‐effects meta‐analysis.

#### Assessment of reporting biases

We assessed the potential for reporting bias for the primary outcome using a funnel plot when at least 10 studies were available for a meta‐analysis.

#### Data synthesis

We used the Online Resource for Recruitment research in Clinical triAls (ORRCA) project's taxonomy of recruitment interventions to classify the interventions included in this review [26]. We split one ORRCA category ('Recruitment information needs') into two to distinguish interventions aimed at the consent process from those aimed at more general participant information. Our seven categories are as follows.

- **Category A: design:** changes to the general design of the trial made specifically to increase recruitment.
- **Category B: pre‐trial planning:** work done before the trial starts (possibly in a separate study) to explicitly make it more likely that recruitment will be successful.
- **Category C: trial conduct changes:** initiatives implemented once the trial has started, including better ways of identifying participants, changes to how data are collected, changes to the type of data collected, and tailoring recruitment to different types of participants.
- **Category D: modifications to the consent process:** changes to the personnel obtaining consent, the timing of consent, what sort of consent information is presented and how it is presented.
- **Category E: modifications to the information given to potential participants about the trial:** including who provides it, when, where, what sort of information is presented, and how the information is presented.
- **Category F: interventions aimed at the recruiter or recruitment site:** any interventions aimed at the recruiter or recruitment site staff, such as changes to training.
- **Category G: incentives:** monetary and other incentives for participants (but not staff, which is covered in a separate review [14]).

We conducted random‐effects meta‐analyses when at least two studies evaluated the same recruitment strategy. Unlike previous versions of this review, which used fixed‐effect models in the absence of substantial heterogeneity, we used random‐effects models throughout because we anticipated substantial heterogeneity across most comparisons. In accordance with updated guidance in the *Cochrane Handbook for Systematic Reviews of Interventions* [27], we estimated between‐study variance using the restricted maximum likelihood (REML) method. We calculated confidence intervals for the summary effect using the Wald‐type method by default. However, when the estimated between‐study variance was greater than zero and more than two studies contributed to a comparison, we used the Hartung‐Knapp‐Sidik‐Jonkman (HKSJ) method.

To synthesise cost‐effectiveness evidence across studies within each comparison, we reported the incremental cost‐effectiveness ratio (ICER), defined as the additional/incremental cost of the intervention strategy compared with the control strategy per additional participant recruited. We measured incremental effectiveness using the RD, defined as the number of additional participants recruited, and defined incremental cost as the difference in cost between the intervention and control strategies.

When all studies in a comparison reported cost data, the RD used in the cost‐effectiveness analysis matched the RD estimated from the pooled effectiveness results. When only a subset of studies reported cost data, we re‐estimated the RD using data from those studies only. For single‐study comparisons, we estimated RDs using fixed‐effect meta‐analysis with Mantel‐Haenszel weighting [27]. For comparisons involving multiple studies, we conducted random‐effects meta‐analyses using inverse‐variance weighting [27].

For comparisons with cost data from multiple studies, incremental costs were weighted according to the same weights applied in the estimation of the RD. This approach enabled us to estimate the overall incremental cost of each recruitment strategy. We were unable to synthesise incremental costs using single‐arm meta‐analysis methods because the included SWATs did not report cost variability data.

We calculated ICERs only when both incremental cost and incremental effectiveness data were available for a given strategy. All ICERs are reported in pounds sterling (GBP) at 2023 price levels. For studies reporting cost data in GBP at price levels other than 2023, we adjusted costs to 2023 values using the UK Consumer Price Index (CPI) [28]. For studies reporting cost data in other currencies and price years, we first adjusted costs to 2023 values using the CPI for the relevant country [29], and then converted the figures to GBP using the UK integrated tariff exchange rates [30].

#### Subgroup analysis and investigation of heterogeneity

We planned to explore the following factors in subgroup analyses for our primary outcome (proportion of eligible participants recruited). We assumed sufficient studies would be identified for these analyses and that these factors represented plausible explanations for any observed heterogeneity.

- Type of allocation concealment used to evaluate recruitment strategies (adequate versus inadequate or unclear).
- Setting of the study recruiting participants (e.g. primary versus secondary care; healthcare versus non‐healthcare settings).
- Type of health condition in healthcare studies (e.g. cancer versus lifestyle change).
- Design of the study recruiting participants (e.g. open‐label versus blinded studies, trials with placebo arms versus those without).
- Target group (e.g. ethics committees, clinicians, patients).

However, there were too few studies to permit subgroup analysis except for one strategy (monetary incentive versus no incentive – detailed in the Effects of methods).

#### Sensitivity analysis

We did not perform sensitivity analyses because the limited number of replications for most strategies precluded a meaningful assessment of the robustness of the results. If the evidence base for specific recruitment strategies expands in future review updates, we will consider the feasibility of conducting sensitivity analyses.

##### Certainty of the evidence assessment

We used the GRADE framework to assess the certainty of the evidence for our primary outcome (proportion of eligible individuals or centres recruited), and where data were available, our secondary outcomes (cost‐effectiveness, retention, participant EDI characteristics) [31]. We present GRADE assessments for meta‐analysed comparisons in the corresponding summary of findings tables [32]. These tables compare specific recruitment strategies with standard practice or alternative interventions and report the primary and secondary outcomes.

As all included studies were randomised SWATs, we started each GRADE assessment at high certainty and considered downgrading the evidence for risk of bias, inconsistency, indirectness, imprecision, and publication bias [31, 32]. For all comparisons, we assessed:

- risk of bias, downgrading by one level when studies were judged to have an overall high or unclear risk of bias. This differs from previous versions of the review, in which we automatically downgraded studies at high overall risk of bias by two levels;
- inconsistency, downgrading when there was substantial unexplained heterogeneity across studies;
- indirectness, downgrading when there were key differences across study interventions or methods;
- imprecision, downgrading when confidence intervals were wide or crossed the line of no effect (risk difference = 0);
- publication bias, downgrading when we detected evidence of reporting bias.

For single‐study comparisons, we:

- did not assess inconsistency as it is inapplicable;
- downgraded by one level for imprecision due to sparse data and by an additional level when confidence intervals were wide and crossed the line of no effect.

We used the GRADE assessments to inform the narrative summaries of intervention effects presented in the Results, in accordance with GRADE guidance [33].

## Results

### Description of studies

#### Results of the search

We conducted the literature search on 15 and 16 February 2023, identifying 21,127 records. Additionally, we screened the 68 studies included in the previous version of the review, as well as 148 studies identified via reference checking and handsearching (Supplementary material 7). After deduplication, we screened 21,072 titles and abstracts, excluded 20,876 records, and retrieved 196 full‐text articles for detailed screening. A total of 91 studies (53 new to this update), providing 94 comparisons and reported across 78 articles, were eligible for inclusion (see the study flow diagram in Figure).

**2:**
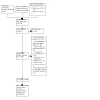
PRISMA study flow diagram *The number of included studies does not match the number of eligible records because the reporting differs across the 78 included records, ultimately yielding a total of 94 comparisons. Four records reported a total of 17 unique SWATs not published elsewhere, resulting in a sub‐total of 91 SWATs. Three of these were 3‐arm SWATs, each contributing one additional comparison. Adding these three 3‐arm comparisons results in a total of 94 comparisons.

#### Included studies

Studies came from 10 countries; there were also four multinational studies, one conducted in Australia and New Zealand, one conducted in the UK and Ireland, one involving the UK, Netherlands, Belgium, Poland, and Greece, and one involving 19 countries. The UK and USA dominated, with 46 and 23 studies, respectively. The next largest contributor was Australia with six studies. The full breakdown is given in Table.

**1:** Countries where the included studies took place

**Country**	**Number of studies**
UK	46
USA	26^a^
Australia	6
Canada	3
Multinational	3 (one involving the UK and Ireland, one involving Australia and New Zealand, and one involving 19 countries)
Estonia	2
France	2
Denmark	1
Norway	1
Sweden	1
Republic of Ireland	1

^a^There were 23 included studies from the USA; however, three of these studies are three‐arm SWATs (two of which were reported in a single published paper).

Eighty‐one studies involved strategy interventions aimed at trial participants, and 10 studies evaluated strategy interventions aimed at those recruiting participants (Supplementary material 8). At least 176,747 individuals were involved in 89 studies; it was not clear how many participants were recruited in two studies (Supplementary material 9). The figure of 176,747 includes individuals who were successfully recruited as well as those who were approached about recruitment but declined or did not respond. We describe the included studies in Supplementary material 2. The full data package for the review is available as Supplementary material 5.

##### Equity, diversity, and inclusion characteristics of included participants (PRO‐EDI)

Nearly all studies (99%) were conducted in Global North, high‐income countries [34, 35], with only one study (1%) including both high‐ and middle‐income settings. Just over half of the studies (53%) reported participants’ age. Fewer reported other characteristics: 35% reported sex, 29% reported gender, 14% reported ethnicity, and 13% reported race. Socioeconomic status was reported in 17% of studies using education, 15% using a deprivation index, and another 15% using other measures. Overall, few studies provided information on participant diversity beyond age.

Table outlines the EDI characteristics of the included participants. See Supplementary material 11 for a detailed report of the EDI data presented in the SWATs.

**2:** Reported EDI characteristics of participants in included studies

**Equity, diversity, and inclusion (EDI) characteristics**		**Number (%) of studies reporting this characteristic (of 91 studies)**
Location (country/countries of data collection and site co‐ordination)	High‐income country (Global North) only	90 (99)
	Mix of high‐ and middle‐income countries*	1 (1)
Age		49 (54)
Sex		32 (35)
Gender		27 (30)
Ethnicity		13 (14)
Race		12 (13)
Socioeconomic status: deprivation index		14 (15)
Socioeconomic status: education		16 (18)
Socioeconomic status: other		14 (15)

*Monaghan 2007 was undertaken in Australia, Canada, Czech Republic, Estonia, France, Germany, Hungary, India, Ireland, Italy, Lithuania, Malaysia, the Netherlands, New Zealand, the Philippines, Poland, Russia, Slovakia, and the United Kingdom.

##### Cost‐effectiveness

We identified and extracted costing data from 17 of 91 studies (19%). Of these, 11 studies (64%) explicitly reported incremental costs including costing data, and eight (47%) explicitly reported direct estimates of incremental cost‐effectiveness ratios (ICERs) including costing data. Overall, cost‐effectiveness results, presented in terms of the ICER, were reported for nine of the 65 strategies (14%). The corresponding ICERs are presented in Table 7a, as part of Supplementary material 12, and detailed cost‐effectiveness data, along with the underlying calculations, are also available in Supplementary material 12.

##### Impact of recruitment strategies on retention rates

Thirteen of the 91 studies (14%) reported the impact of recruitment strategies on participant retention rates. Of these, four (31%) reported data at the host trial’s primary outcome time point, and nine (69%) reported data at other time points. Analyses of the impact of recruitment strategies on retention rates are available in Supplementary material 13. We followed the approach used by Gillies and colleagues in their 2021 Cochrane review on strategies to improve retention rates [18]: specifically, we used the retention rate at the primary outcome time point for the host trial, where reported. If these data were not reported, we used the retention rate for the first time point reported.

#### Excluded studies

As depicted in the flow diagram (Figure), we excluded 118 studies in this review update. This included 31 previously included studies that no longer met the eligibility criteria (28 studies were hypothetical and three were quasi‐randomised). Reasons for exclusions are listed in the flow diagram (Figure), and all studies excluded at the full‐text screening stage are documented in Supplementary material 3, with the reasons for exclusion.

### Risk of bias in included studies

We rated 52 studies to have an overall low risk of bias, 25 to have an unclear risk, and 15 studies to have a high risk. For details, see the individual risk of bias tables in Supplementary material 2, as well as Figure and Figure for summaries of the risk of bias assessment.

**3:**
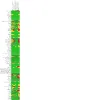
Risk of bias summary: review authors' judgements about each risk of bias item for each included study

### Effects of methods

In Table, we present an overview of the included studies organised according to the seven intervention categories described in the Data synthesis section. We assigned studies to these categories using the available information and the authors’ stated focus. We acknowledge that both the distinctions between categories and the intervention descriptions leave room for subjective interpretation. In Table, we present an overview of the synthesis, the included studies' characteristics, and outcomes.

**3:** Intervention categories

Study	Host trial intervention	Type of participants
**A–Design.** This includes changes to the general design of the trial made specifically to increase recruitment. N = 7		
Avenell 2004	Drug: vitamin D tablet	Patients (adults): attending a fracture clinic or orthopaedic ward
Cooper 1997	Drug/surgery: medical management or transcervical resection of the endometrium	Patients (adults): first‐time attendees at a gynaecological clinic
Fowell 2006	Drug: anti‐emetics only if symptomatic	Patients (adults): cancer inpatients receiving palliative care
Hemminki 2004	Drug: HRT	Patients (adults): postmenopausal women considering HRT
Litchfield 2005	Device: alternative delivery systems (NovoPen and Innovo) for insulin	Patients (probably adults): people with type 1 diabetes
Paul 2011	Drug: adjuvant treatment	Patients (probably adults): with colorectal cancer
Veerus 2016	Drug: postmenopausal hormone therapy	Patients (adults): postmenopausal women
**B–Pre‐trial planning.** This includes work done before the trial starts (possibly in a separate study) that explicitly aims to increase recruitment success. N = 1		
Garvelink 2015	Training: training interprofessional home‐care teams in shared decision‐making	Patients (adults): elderly patients receiving home care
**C–Trial conduct changes.** This includes initiatives implemented once the trial has started, such as better ways of identifying participants, changes to how data are collected, changes to the type of data collected and tailoring recruitment to different types of participant. N = 6		
Bracken 2019	Drug: testosterone for type 2 diabetes	Patients (adults): obese or overweight men with prediabetes or type 2 diabetes and low testosterone
Free 2010	Behaviour: mobile phone‐based smoking cessation	Healthy volunteers (adults): smokers
Free 2011	Behaviour: mobile phone‐based smoking cessation	Healthy volunteers (adults): smokers
Nystuen 2004	Therapy: psychologist intervention for issues linked to psychological problems or musculoskeletal pain	Patients (adults): on sick leave receiving benefits
Treweek 2012	Drug: antibiotic prescribing	Health professionals (adults): family doctors
Wong 2013	Screening: colorectal cancer screening	Healthy volunteers (adults): eligible for colorectal cancer screening
**D–Modification to the consent form or process.** This includes changes to personnel helping with consent, when consent is taken, what sort of consent information is presented and how it is presented. N = 5 (2 of which were excluded from the results)		
Abd‐Elsayed 2012 [136]*	Drug or blood storage trials	Patients (adults): eligible for 1 of 3 trials, all of whom had substantial illness requiring major surgery (cardiac)
Coyne 2003	Drug: various	Patients (adults): eligible for cancer trial
Lipstein 2021 [137]*	Drug: methotrexate for paediatric Crohn’s disease patients	Patients (children and young adults): paediatric Crohn’s disease patients.
Trevena 2006	Screening: colorectal cancer	Healthy volunteers (adults): eligible for colorectal screening
Wadland 1990	Lifestyle: smoking cessation	Healthy volunteers (adults): smokers
**E–Modification to the information given to potential participants about the trial.** This includes who provides it, when, where, what sort of information is presented, and how the information is presented. N = 52 (4 of which were excluded from the results)		
Arundel 2017	Therapy: podiatry intervention for falls prevention	Patients (adults): older patients who have attended routine podiatry services or with a fear of falling
Bergenmar 2014	Drug: various	Patients (probably adults): eligible for cancer trials
Bobb 2016	Drug: oral antiseptic for preventing hospital‐acquired pneumonia	Patients (adults): emergency department patients expected to be admitted to hospital
Brierley 2012	Therapy: cognitive behavioural therapy	Patients (adults): depression
Brown 2015	Lifestyle: lifestyle intervention for pregnant women with gestational diabetes or prediabetes	Patients (adults): pregnant women with gestational diabetes or pre‐diabetes
Chen 2011	Unclear	Patients (probably adults): unclear what type
Chow 2020	Lifestyle: self‐management intervention to treat heart disease	Patients (adults): childhood cancer survivors at risk of heart disease or heart failure
Cockayne 2017 (START PIL REFORM)^a^	Device: orthosis	Patients (adults): podiatry
Crane 2016	Lifestyle: weight gain prevention	Healthy volunteers (adults): normal and overweight young adults
Dear 2011 [138]*	Information: access to cancer trials site	Patients (adults): have cancer
Du 2008	Cancer trials (unspecified)	Patients (adults): lung cancer
Du 2009	Cancer trials (unspecified)	Patients (adults): women with breast cancer
Dwyer 2022	Therapy: cognitive occupation‐based programme	Patients (adults): people with multiple sclerosis and cognitive difficulty
Felicitas‐Perkins 2017 [139]*	Unspecified: cancer trials	Patients (adults): Filipino patients eligible for an ongoing oncology trial
Ford 2004	Screening: prostate, lung and colorectal cancer screening	Healthy volunteers (adults): men eligible for prostate, lung and colorectal cancer screening
Foss 2016	Vaccination	Healthy volunteers (adults): pregnant women
Fracasso 2013	Cancer trials (unspecified)	Patients (adults): cancer (various)
Hughes‐Morley 2016	Therapy: training for mental health professionals	Patients (adults): adults with severe mental illness
Hutchison 2007	Cancer trials (unspecified)	Patients (probably adults): cancer (various)
Ives 2001	Unclear but probably drug	Patients (adults): people with HIV
Jolly 2019 (START MMI PSM‐COPD)^b^	Therapy: telephone self‐management coaching	Patients (adults): primary care patients with chronic obstructive pulmonary disease
Kamen 2018	Cancer trials (unspecified)	Patients (adults): oncology patients eligible for an ongoing oncology trial
Kendrick 2001	Injury prevention trial	Healthy volunteers (adults and children): families
Kimmick 2005	Cancer trials (various)	Patients (adults): cancer (various)
Knapp 2020 (START PIS CASPER)^a^	Behaviour: behavioural activation for sub‐threshold depression	Healthy volunteers (adults): older adults with sub‐threshold depression
Knapp 2023e (Thermic‐3)^c^	Therapy: warm versus cold blood during open‐heart surgery	Patients (children): children undergoing open‐heart surgery
Knapp 2023a (BALANCE)^c^	Therapy: customised movies viewed on a console	Patients (children): children with lazy eye
Knapp 2023b (BAMP)^c^	Therapy: orthodontic treatment	Patients (children): children with an underbite
Knapp 2023c (CHAMP‐UK)^c^	Therapy: eye drop therapy	Patients (children): children with short‐sightedness
Knapp 2023d (FORCE)^c^	Device: splint versus soft bandage immobilisation	Patients (children): children with a torus fracture
Knapp 2023f (UKALL)^c^	Therapy: alternative chemotherapy dosing	Patients (children and young adults): patients with lymphocytic cancer
Larkey 2002	Various targeting cardiovascular disease, cancer and osteoporosis	Healthy volunteers: (adults) women
Man 2015 (START PIS Healthlines Depression)^a^	Therapy: telephone support and self‐management	Patients (adults): cardiovascular
Madurasinghe 2021 (START PIS HeLP Diabetes)^a^	Behaviour: web‐based self‐management programme	Patients (adults): people with type 2 diabetes
Madurasinghe 2023c (START MMI HI‐Light)^b^	Therapy: home interventions and light therapy for vitiligo	Patients (children and adults): people with vitiligo
Madurasinghe 2023d (START MMI seAFOod)^b^	Therapy/drug: fish oil and aspirin for polyp prevention	Healthy volunteers (adults): individuals at high risk of colon cancer
Madurasinghe 2023a (START MMI GHT2000a)^b^	Behaviour: web‐delivered exercise self‐referral tool	Patients (adults): inactive adults with high normal blood pressure or hypertension
Madurasinghe 2023b (START MMI GHT2000b)^b^	Behaviour: exercise programme referral	Patients (adults): inactive adults with high normal blood pressure or hypertension
Mattock 2020	Behaviour: positive parenting intervention	Healthy volunteers (children): parents of toddlers exhibiting behavioural problems
McCaffery 2019	Therapy: occupation therapy for falls prevention	Healthy volunteers (adults): elderly people who had fallen or had a fear of falling
Miller 2021a	Drug: vitamin D for falls prevention	Healthy volunteers (adults): older people at risk of falling, with low vitamin D
Paris 2015	Clinical trials (various)	Patients (adults): adults being screened for an ongoing trial
Parker 2018 (START PIS ECLS)^a^	Screening: a new cancer screening test	Healthy volunteers (adults): smokers undergoing lung cancer screening
Paul 2014	Screening: colorectal cancer	Healthy volunteers (adults): colorectal cancer screening
Rogers 2019	Drug: comparing different treatments for gout	Patients (adults): chronic gout patients with cardiovascular risk factors
Saif 2020	Drug: Alzheimer’s disease treatment for Alzheimer's prevention	Healthy volunteers (adults): adults at risk of developing Alzheimer’s disease
Sheridan 2020 (START PIS ISDR)^a^	Screening: personalised risk‐based screening for diabetic retinopathy	Patients (children and adults): diabetic patients attending diabetic retinopathy screening
Skinner 2019 [140]*	Behaviour: educational video	Patients (adults): oncology patients
Simes 1986	Unclear: cancer	Patients (adults): cancer
Sundaresan 2017	Therapy: alternative timings for radiotherapy after a radical prostatectomy	Patients (adults): high‐risk prostate cancer patients
White 2023	Therapy: medical appointments with a specially‐trained GP	Patients (adults): patients with persistent, medically unexplained, physical symptoms
Witham 2018 [141]*	Drug: vitamin K supplementation	Healthy volunteers (adults): older people at risk of falls
**F–Interventions aimed at the recruiter or recruitment site.** This includes anything aimed at the recruiter or recruitment site staff, such as changes to training. N = 9 (2 of which were excluded from the results)		
Agni 2022	Surgery: high‐dose dual‐antibiotic cement versus single‐antibiotic cement during partial hip replacement	Patients (adults): older adults with intracapsular hip fracture requiring acute surgery
Ethier 2017 [142]*	Drug: comparing on‐demand versus continuous use medication for gastric reflux	Patients (adults): people with gastric reflux requiring protein pump inhibitors
Isaksson 2025	Drug: antidepressant medication versus placebo for stroke recovery	Patients (adults): patients who have recently had a stroke
Jefferson 2018	Therapy: cast treatment versus surgical fixation	Patients (adults): patients with a wrist fracture
Lee 2017	Therapy: pain education	Staff at primary care clinics (sites are target, not patients)
Liénard 2006	Drug: breast cancer treatment	Staff at breast cancer treatment centres (sites are target, not patients)
Maxwell 2017	Drug: restarting versus avoiding antiplatelet drugs following a stroke	Patients (adults): patients who had experienced a stroke due to intracerebral haemorrhage
Monaghan 2007	Unclear: diabetes management	Staff at clinical sites recruiting to a diabetes and vascular disease treatment trial (sites are target, not patients)
Tilley 2012 [143]*	Drug: Parkinson's disease	Neurologists, primary care doctors and internists (adults)
Tilley 2021	Drug: pharmacological treatments for various conditions depending on host trial, namely atrial fibrillation, blood and marrow transplants, colon cancer prevention, and Parkinson’s	Patients (adults): patients eligible for various host trials covering atrial fibrillation, blood and marrow transplants, colon cancer prevention, and Parkinson’s
**G–Incentives.** Financial and other incentives for participants. N = 12		
Fairhurst 2022	Lifestyle: yoga programme	Patients (adults): patients with multiple chronic conditions
Free 2010	Lifestyle: mobile phone‐based smoking cessation	Healthy volunteers (adults): smokers
Giguere 2015	Behaviour: online education	Healthy volunteers (adults): family physicians
Halpern 2021a ($200)^d^	Behaviour: varenicline and behavioural activation for smoking cessation	Patients (adults): depressed smokers
Halpern 2021b ($100)^d^	Device: gamification intervention with a social support partner to increase patient mobility after hospital discharge	Patients (adults): hospital in‐patients
Jennings 2015a^e^	Drug: NSAID	Patients (adults): arthritis
Jennings 2015b^e^	Drug: hyperuricaemia	Patients (adults): symptomatic hyperuricaemia
Jennings 2015c^e^	Drug: hypertension	Patients (adults): hypertension
Jennings 2015d^e^	Drug: hypertension	Patients (adults): hypertension
Jennings 2015e^e^	Drug: diuretic therapy	Patients (adults): metabolic syndrome
White 2023	Therapy: medical appointments with a specially‐trained GP	Patients (adults): patients with persistent, medically unexplained, physical symptoms
Whiteside 2019	Therapy: occupation therapy for falls prevention	Healthy volunteers (adults): elderly people who had fallen or had a fear of falling

**GP**: general practice; **HIV**: human immunodeficiency virus; **HRT**: hormone replacement therapy; **MMI**: multimedia information; **NSAID**: non‐steroidal anti‐inflammatory drugs. ^*^Studies excluded from the results because we judged them to be at high overall risk of bias, and they tested a comparison not evaluated in another study. ^a^Cockayne 2017 (START PIL REFORM); Knapp 2020 (START PIS CASPER); Madurasinghe 2021 (START PIS HeLP Diabetes); Man 2015 (START PIS Healthlines Depression); Parker 2018 (START PIS ECLS); and Sheridan 2020 (START PIS ISDR) were all from the MRC START PIS programme, a series of embedded trials evaluating the impact of optimised participant information sheets within multiple host trials.  ^b^Jolly 2019 (START MMI PSM‐COPD); Madurasinghe 2023a (START MMI GHT2000a); Madurasinghe 2023b (START MMI GHT2000b); Madurasinghe 2023c (START MMI HI‐Light); and Madurasinghe 2023d (START MMI seAFOod) were all from the MRC START MMI programme, a series of embedded trials evaluating the impact of MMI on trial recruitment within multiple host trials.  ^c^Knapp 2023a (BALANCE); Knapp 2023b (BAMP); Knapp 2023c (CHAMP‐UK); Knapp 2023d (FORCE); Knapp 2023e (Thermic‐3); and Knapp 2023f (UKALL) were all from the TRECA (TRials Engagement in Children and Adolescents) study. This was a series of co‐ordinated trials evaluating the impact of modified participant information sheets on trial recruitment within multiple paediatric host trials.  ^d^Halpern 2021a ($200) and Halpern 2021b ($100) are from a single paper that describes two separate embedded 3‐arm recruitment trials. ^e^ Jennings 2015a, Jennings 2015b, Jennings 2015c, Jennings 2015d, and Jennings 2015e are from a single paper that describes five embedded recruitment trials.

**4:** Overview of synthesis and included studies

**Study**	**Country**	**Host trial area**	**Study design**	**Interventions**	**Outcomes**	**Analysis**
**A–Design.** This includes changes to the general design of the trial made specifically to increase recruitment (N = 7). Note: EDI data reporting was very limited for studies in this category; interpret results with caution regarding generalisability to under‐served groups.						
Avenell 2004	UK	Fracture prevention	Randomised trial	Investigated the effect of different trial designs	Proportion recruited to host trial	MA
Cooper 1997	UK	Management strategies for heavy menstrual bleeding	Randomised embedded trial	Investigated the effect of different trial designs	Proportion recruited to host trial	MA
Fowell 2006	UK	Palliative care	Cluster‐randomised cross‐over SWAT	Investigated the effect of different trial designs	Proportion recruited to host trial	Narrative summary
Hemminki 2004	Estonia	Postmenopausal hormone therapy	Randomised SWAT	Investigated the effect of different design methods	Proportion recruited to host trial	MA
Litchfield 2005	UK	Delivery systems for insulin	Cluster‐randomised SWAT	Investigated electronic data capture	Number of participants recruited to the trial	MA
Paul 2011	UK	Adjuvant treatment for colorectal cancer	Randomised SWAT	Investigated the effect of the randomisation time point	Proportion recruited to host trial	Narrative summary
Veerus 2016	Estonia	Postmenopausal hormone therapy	Randomised SWAT	Investigated the effect of participant being unblinded to the host trial intervention	Proportion recruited to trial	MA
**B–Pre‐trial planning.** This includes work done before the trial starts (possibly in a separate study) that explicitly aims to increase recruitment success (N = 1). Note: EDI data were not reported for the study in this category; interpret results with caution regarding generalisability to under‐served groups.						
Garvelink 2015	Canada	Frailty	Cluster‐randomised cross‐over trial	Investigated pre‐engagement with potential trial sites during proposal development	Proportion recruited to host trial	MA
**C–Trial conduct changes.** This includes initiatives implemented once the trial has started, such as better ways of identifying participants, changes to how data are collected, changes to the type of data collected and tailoring recruitment to different types of participant (N = 6). Note: EDI data reporting was very limited for studies in this category; interpret results with caution regarding generalisability to under‐served groups.						
Bracken 2019	Australia	Testosterone for type 2 diabetes	Randomised SWAT	Investigated telephone call reminder versus SMS reminder	Proportion recruited to host trial, cost‐effectiveness	MA
Free 2010	UK	Smoking cessation	Randomised SWAT	Investigated whether including GBP 5 with invitation or sending SMS messages to potential participants increased recruitment	Proportion recruited to host trial	MA
Free 2011		Smoking cessation	Randomised SWAT	Investigated effect of mentioning scarcity on recruitment	Proportion recruited to host trial	MA
Nystuen 2004	Norway	Getting people to return to work	Randomised SWAT	Investigated the effect of different telephone reminders	Proportion recruited to host trial	MA
Treweek 2012	UK	Antibiotic prescribing by GPs	Randomised SWAT	Investigated use of different modes of invitation to take part in trial	Proportion recruited to host trial	MA
Wong 2013	Canada	Colorectal cancer	Randomised SWAT	Investigated use of telephone reminders to non‐responders	Proportion recruited to host trial	MA
**D–Modification to the consent form or process.** This includes changes to personnel helping with consent, when consent is taken, what sort of consent information is presented, and how it is presented (N = 5, 2 of which were excluded from the results). Note: EDI data reporting was very limited for studies in this category; interpret results with caution regarding generalisability to under‐served groups.						
Abd‐Elsayed 2012*	USA	Cardiac surgery	Randomised SWAT	Investigated the use of different consent form presentations Intervention A: consent documents on heavy‐ weight cream‐coloured paper (20‐pound) and a blue folder Comparator: consent documents as photocopies stapled together	Proportion recruited to host trial	Not presented
Coyne 2003	USA	Cancer	Cluster‐randomised SWAT	Investigated the effect of different consent methods	Proportion recruited to host trial	Narrative summary
Lipstein 2021*	USA	Paediatric Crohn’s disease	Cluster‐randomised cross‐over trial	Investigated enhanced pre‐consent discussion with aid of poster	Proportion recruited to host trial	Not presented
Trevena 2006	Australia	Colorectal cancer	Randomised SWAT	Investigated the effect of different trial information methods	Proportion recruited to host trial	MA
Wadland 1990	USA	Smoking cessation	Randomised SWAT	Intervention: consent form read out by researcher	Proportion recruited to host trial	MA
**E–Modification to the information given to potential participants about the trial.** This includes who provides it, when, where, what sort of information is presented, and how the information is presented (N = 52, 4 of which were excluded from the results). Note: EDI data reporting was very limited for studies in this category; interpret results with caution regarding generalisability to under‐served groups.						
Arundel 2017	UK and Ireland	Falls prevention	Randomised SWAT	Investigated a recruitment primer leaflet	Proportion recruited to host trial, cost‐effectiveness	MA
Bergenmar 2014	Sweden	Cancer	Randomised SWAT	Investigated use of audio recording to improve communication about the trial	Proportion recruited to host trial	MA
Bobb 2016	USA	Emergency department trials	Randomised SWAT	Telemedicine‐enabled research informed consent versus standard face‐to‐face consent	Proportion recruited to host trial	MA
Brierley 2012	UK	Depression	Randomised SWAT	Investigated effect of length of the participant information leaflet on recruitment	Proportion recruited to host trial	MA
Brown 2015	USA	Lifestyle intervention for gestational diabetes or pre‐diabetes	Randomised SWAT	Investigated a targeted invitation letter for minority groups	Proportion recruited to host trial	MA
Chen 2011	UK	Clinical area unclear	Randomised SWAT	Investigated different versions of the participant information leaflet (PIL)	Proportion recruited to pre‐randomisation phase of host trial	MA
Chow 2020	USA	Self‐management for coronary heart disease following paediatric cancer treatment	3‐arm randomised SWAT	Investigated shortened introductory packets with simplified consent	Proportion recruited to host trial	MA
Cockayne 2017 (START PIL REFORM) ^a^	UK	Falls prevention	Randomised SWAT	Investigated two different versions of the participant information leaflet (PIL): optimised PIL (bespoke and user‐tested) or a template‐developed PIL	Proportion recruited to host trial	MA
Crane 2016	USA	Behavioural intervention for weight gain prevention	Randomised SWAT	Investigated gender‐targeted recruitment postcards	Proportion recruited to host trial	MA
Dear 2011*	Australia	Cancer (unspecified)	Cluster‐randomised cross‐over trial	Investigated whether information provided through a website improved recruitment	Self‐reported (by participant) recruitment to a trial	Not presented
Du 2008	USA	Lung cancer	Randomised SWAT	Investigated the effect of different methods of providing information about the trial	Proportion recruited to host trial	MA
Du 2009	USA	Breast cancer	Randomised SWAT	Intervention: 18‐minute video. The video presents an overview of phase I, II and III clinical trials and the importance of cancer clinical research to society. The video addresses common concerns regarding clinical trials and cancer treatment from the patient's perspective, such as side effects, expected risks and benefits, eligibility criteria, the enrolment process, and treatment costs.	Enrolment in therapeutic trials	MA
Dwyer 2022	Republic of Ireland	Cognitive rehabilitation intervention for multiple sclerosis	Randomised SWAT	Investigated patient‐designed‐and‐informed PIL	Proportion recruited to host trial, proportion retained at 6 months.	MA
Felicitas‐Perkins 2017*	USA	Cancer (unspecified)	Randomised SWAT	Investigated culturally‐tailored educational DVD about clinical trials	Proportion recruited to host trial	Not presented
Ford 2004	USA	Cancer	Randomised SWAT	Investigated the effect of different trial information and consent methods	Proportion recruited to host trial	MA
Foss 2016	Denmark	Vaccination	Randomised SWAT	Investigated the effect of different trial information and consent methods	Proportion recruited to host trial	MA
Fracasso 2013	USA	Cancer	Randomised SWAT	Investigated coaching as a way of improving recruitment	Proportion recruited to host trial	Narrative summary
Hughes‐Morley 2016	USA	Care planning training for community mental health teams	Cross‐factorial, cluster‐randomised SWAT	Investigated leaflet advertising patient and public involvement	Proportion recruited to host trial	MA
Hutchison 2007	UK	Cancer trials (unspecified)	Randomised SWAT	Investigated providing trial information by video	Proportion recruited to host trial	MA
Ives 2001	UK	HIV	Randomised SWAT	Investigated the effect of different trial information methods	Proportion recruited to host trial	MA
Jolly 2019 (START MMI PSM‐COPD)^b^	UK	COPD	Cluster‐randomised cross‐over trial	Investigated multimedia trial information	Proportion recruited to host trial, proportion retained at 6 and 12 months, intervention costs	MA
Kamen 2018	USA	Oncology	Randomised SWAT	Investigated multimedia trial information	Proportion recruited to host trial	MA
Kendrick 2001	UK	Injury prevention, recruiting family units	Cluster‐randomised cross‐over trial	Investigated the effect of different trial information methods	Proportion recruited to host trial, proportion retained at 6 and 12 months, intervention costs	MA
Kimmick 2005	USA	Cancer	Cluster‐randomised cross‐over trial	Investigated the effect of different trial information methods	Proportion recruited to host trial	Narrative summary
Knapp 2020 (START PIS CASPER)^b^	UK	Behavioural activation for depression	Randomised SWAT	Investigated optimised participant information leaflet (bespoke and user‐tested)	Proportion recruited to host trial, proportion retained.	MA
Knapp 2023e (Thermic‐3)^c^	UK	Paediatric congenital heart defects	3‐arm randomised SWAT	Investigated multimedia trial information	Proportion recruited to host trial, proportion retained at 3 months.	MA
Knapp 2023a (BALANCE)^c^	UK	Paediatric lazy eye	3‐arm randomised SWAT	Investigated multimedia trial information	Proportion recruited to host trial, proportion retained at 16 weeks.	MA
Knapp 2023b (BAMP)^c^	UK	Paediatric underbite	3‐arm randomised SWAT	Investigated multimedia trial information	Proportion recruited to host trial, proportion retained at 18 months	MA
Knapp 2023c (CHAMP‐UK)^c^	UK	Paediatric short‐sightedness	3‐arm randomised SWAT	Investigated multimedia trial information	Proportion recruited to host trial, proportion retained at 6 months	MA
Knapp 2023d (FORCE)^c^	UK	Paediatric wrist fracture	Cluster‐randomised cross‐over trial	Investigated multimedia trial information	Proportion recruited to host trial, proportion retained at 6 weeks	MA
Knapp 2023f (UKALL)^c^	UK	Paediatric and adolescent leukaemia or lymphoma	3‐arm randomised SWAT	Investigated multimedia trial information	Proportion recruited to host trial, proportion retained at 3 months	MA
Larkey 2002	USA	Unclear	Cluster‐randomised cross‐over trial	Investigated the effect of different methods of training lay advocates for trials	Proportion recruited to host trial	Narrative summary
Man 2015 (START PIS Healthlines Depression)^a^	UK	Extra support for depression	Randomised SWAT	Investigated an alternative way of presenting patient information materials	Proportion recruited to host trial	MA
Madurasinghe 2021 (START PIS HeLP Diabetes)^a^	UK	Type 2 diabetes self‐management	Randomised SWAT	Investigated optimised participant information leaflet (bespoke and user‐tested)	Proportion recruited to host trial	MA
Madurasinghe 2023c (START MMI HI‐Light)^b^	UK	Vitiligo	Randomised coordinated SWAT (across 5 host trials)	Investigated multimedia trial information	Proportion recruited to host trial, proportion retained at 9 months	MA
Madurasinghe 2023d (START MMI seAFOod)^b^	UK	Bowel cancer prevention	Randomised coordinated SWAT (across 5 host trials)	Investigated multimedia trial information	Proportion recruited to host trial, proportion retained at 12 months	MA
Madurasinghe 2023a (START MMI GHT2000a)^b^	UK	Exercise for high blood pressure	Randomised coordinated SWAT (across 5 host trials)	Investigated multimedia trial information	Proportion recruited to host trial	MA
Madurasinghe 2023b (START MMI GHT2000b)^b^	UK	Exercise for high blood pressure	Randomised coordinated SWAT (across 5 host trials)	Investigated multimedia trial information	Proportion recruited to host trial	MA
Mattock 2020	UK	Paediatric behavioural difficulties	Randomised SWAT	Investigated providing information by video	Proportion recruited to host trial	MA
McCaffery 2019	UK	Falls prevention	Randomised SWAT	Investigated a handwritten personalised invitation letter	Proportion recruited to host trial, proportion retained at 3 months	MA
Miller 2021a	USA	Falls prevention	3‐arm randomised SWAT	Investigated different wording of risk of underlying condition	Proportion recruited to host trial	MA
Paris 2015	France	Multiple host trials in different clinical areas, including heart surgery, dementia, diabetes, and sleep disorders	Randomised SWAT	Investigated simplified PILs	Proportion recruited to host trial	MA
Parker 2018 (START PIS ECLS)^a^	UK	Lung cancer diagnosis	Randomised SWAT	Investigated optimised participant information brochure (bespoke and user‐tested)	Proportion recruited to host trial	MA
Paul 2014	Australia	Colorectal cancer	Randomised SWAT	Investigated pre‐recruitment primer letter	Proportion recruited to host trial	MA
Rogers 2019	UK	Arthritis	Randomised SWAT	Investigated DVD with invitation pack	Proportion recruited to host trial, cost‐effectiveness.	MA
Saif 2020	USA	Alzheimer's disease	Randomised SWAT	Investigated web‐based course	Proportion recruited to host trial	MA
Sheridan 2020 (START PIS ISDR)^a^	UK	Diabetic retinopathy screening	Cluster‐randomised cross‐over trial	Investigated optimised participant information leaflet (bespoke and user‐tested)	Proportion recruited to host trial	MA
Skinner 2019*	Australia	Cancer	Randomised SWAT	Investigated the effect of different consent methods	Proportion recruited to host trial	MA
Simes 1986	USA	Cancer (unspecified)	Randomised SWAT	Investigated in‐office video viewing	Proportion recruited to host trial	Not presented
Sundaresan 2017	Australia and New Zealand	Radiotherapy for prostate cancer	Randomised SWAT	Investigated decision aid	Proportion recruited to host trial	MA
White 2023	UK	Community‐based clinic for patients with persistent, medically unexplained, physical symptoms	2x2 factorial randomised SWAT	Investigated brief PIL and trial‐branded pen	Proportion recruited to host trial, cost‐effectiveness.	MA
Witham 2018*	UK	Falls prevention	Randomised SWAT	Investigated information sheet with different wording	Proportion recruited to host trial	Not presented
**F–Interventions aimed at the recruiter or recruitment site.** This includes anything aimed at the recruiter or recruitment site staff, such as changes to training (N = 9, 2 of which were excluded from the results). **Note:** EDI data were not reported for studies in this category; interpret results with caution regarding generalisability to under‐served groups.						
Agni 2022	UK	Partial hip replacement following hip fracture	2x2 factorial cluster‐randomised SWAT	Investigated additional support and/or digital nudges for Trainee Principal Investigators (TPI)	Total number of patients randomised, from each site, in their first 6 months of trial recruitment	Narrative summary
Ethier 2017*	UK, Netherlands, Belgium, Poland and Greece	Gastric reflux	Cluster‐randomised cross‐over trial	Investigated software for automatically flagging eligible patients for a trial during GP appointments	Proportion recruited to host trial (only reported for 2 out of 5 countries)	Not presented
Isaksson 2025	Sweden	Stroke recovery	Crossover cluster‐randomised SWAT	Investigated teleconference with recruiting centres and commitment contracts	Number of included subjects before and after intervention	Narrative summary
Jefferson 2018	UK	Cast versus surgery for wrist fracture	Cluster‐randomised cross‐over trial	Investigated remote site initiation visits	Proportion recruited to host trial, proportion retained at each follow‐up time point including 12 months (host trial primary endpoint), intervention costs	MA
Lee 2017	Australia	Recruiting GP practices to low back pain trial	Cluster‐randomised cross‐over trial	Investigated the use of a teaser campaign to increase recruitment of clinical centres	Proportion of clinics recruited to host trial	MA
Liénard 2006	France	Breast cancer	Cluster‐randomised cross‐over trial	Investigated the effect of organising visits by the trial co‐ordination team to centres participating in a multicentre trial	Proportion recruited to host trial	Narrative summary
Maxwell 2017	UK	Antiplatelet drugs following stroke	Stepped‐wedge cluster randomised SWAT	Investigated a recruitment co‐ordinator intervention	Number of patients randomised into host trial per month per site	Narrative summary
Monaghan 2007	19 countries	Diabetes	Cluster‐randomised cross‐over trial	Investigated the effect of different levels of communication between the trial co‐ordination team and participating sites	Proportion recruited to host trial	Narrative summary
Tilley 2012*	USA	Parkinson’s Disease	Cluster‐randomised cross‐over trial	Investigated effect of a recruitment coordinator	Proportion recruited to host trial	Not presented
Tilley 2021	USA	Various: atrial fibrillation, blood and marrow transplants, colon cancer prevention, Parkinson’s	Cluster‐randomised cross‐over trial	Investigated trust‐based educational programme	Proportion recruited to host trial	MA
**G–Incentives.** Financial and other incentives for participants (N = 12). Note: EDI data reporting was very limited for studies in this category; interpret results with caution regarding generalisability to under‐served groups.						
Fairhurst 2022	UK	Yoga for older adults with multimorbidities	2x2 factorial cluster‐randomised SWAT	Investigated trial‐branded pen and unconditional GBP 5 incentive enclosed in trial recruitment pack	Proportion recruited to host trial, cost‐effectiveness	MA
Free 2010	UK	Smoking cessation	Randomised SWAT	Investigated whether including GBP 5 with invitation or sending SMS messages to potential participants increased recruitment	Proportion recruited to trial	MA
Giguere 2015	Canada	Recruiting family physicians to a medical education trial	Randomised SWAT	Investigated CAD 300 monetary incentive	Proportion recruited to host trial	MA
Halpern 2021a ($200)^d^	USA	Smoking cessation	3‐arm randomised SWAT	Investigated higher versus lower monetary incentive versus nothing	Proportion recruited to host trial	MA
Halpern 2021b ($100)^d^	USA	Ambulation	3‐arm randomised SWAT	Investigated higher versus lower monetary incentive versus nothing	Proportion recruited to host trial	MA
Jennings 2015a^e^	UK	Arthritis	Randomised coordinated SWAT (across 5 host trials)	Investigated effect of GBP 100 financial incentive on recruitment	Proportion recruited to host trial, cost‐effectiveness	MA
Jennings 2015b^e^	UK	Hyperuricemia	Randomised coordinated SWAT (across 5 host trials)	Investigated effect of GBP 100 financial incentive on recruitment	Proportion recruited to host trial, cost‐effectiveness	MA
Jennings 2015c^e^	UK	Hypertension	Randomised coordinated SWAT (across 5 host trials)	Investigated effect of GBP 100 financial incentive on recruitment	Proportion recruited to host trial, cost‐effectiveness	MA
Jennings 2015d^e^	UK	Hypertension	Randomised coordinated SWAT (across 5 host trials)	Investigated effect of GBP 100 financial incentive on recruitment	Proportion recruited to host trial, cost‐effectiveness	MA
Jennings 2015e^e^	UK	Hypertension	Randomised coordinated SWAT (across 5 host trials)	Investigated effect of GBP 100 financial incentive on recruitment	Proportion recruited to host trial, cost‐effectiveness	MA
White 2023	UK	Community‐based clinic for patients with persistent, medically unexplained, physical symptoms	2x2 factorial randomised SWAT	Investigated brief PIL and trial‐branded pen	Proportion recruited to host trial, cost‐effectiveness.	MA
Whiteside 2019	UK	Falls prevention	Randomised SWAT	Investigated branded pen with invitation pack	Proportion recruited to host trial, costs, proportion retained at 3 months.	MA

**CAD:** Canadian dollars; **EDI:** equity, diversity, and inclusion; **GBP:** pounds sterling; **GP**: general practice; **HIV**: human immunodeficiency virus; **HRT**: hormone replacement therapy; **MA**: meta‐analysis; **MMI**: multimedia information; **NSAID**: non‐steroidal anti‐inflammatory drugs; **PIL:** participant information leaflet; **SWAT:** Study Within A Trial ^*^Studies excluded from the results sections because we judged them to be at overall high risk of bias studies, and they tested a comparison not evaluated in another study. ^a^Cockayne 2017 (START PIL REFORM); Knapp 2020 (START PIS CASPER); Madurasinghe 2021 (START PIS HeLP Diabetes); Man 2015 (START PIS Healthlines Depression); Parker 2018 (START PIS ECLS); and Sheridan 2020 (START PIS ISDR) were all from the MRC START PIL programme, a series of embedded trials evaluating the impact of optimised participant information leaflets (bespoke & user‐tested) within multiple host trials.  ^b^Jolly 2019 (START MMI PSM‐COPD); Madurasinghe 2023a (START MMI GHT2000a); Madurasinghe 2023b (START MMI GHT2000b); Madurasinghe 2023c (START MMI HI‐Light); and Madurasinghe 2023d (START MMI seAFOod) were all from the MRC START MMI programme, a series of embedded trials evaluating the impact of MMI on trial recruitment within multiple host trials.  ^c^Knapp 2023a (BALANCE); Knapp 2023b (BAMP); Knapp 2023c (CHAMP‐UK); Knapp 2023d (FORCE); Knapp 2023e (Thermic‐3); and Knapp 2023f (UKALL) were all from the TRECA (TRials Engagement in Children and Adolescents) study. This was a series of co‐ordinated trials evaluating the impact of modified participant information sheets on trial recruitment within multiple paediatric host trials.  ^d^Halpern 2021a ($200) and Halpern 2021b ($100) are from a single paper that describes two separate embedded 3‐arm recruitment trials. ^e^ Jennings 2015a, Jennings 2015b, Jennings 2015c, Jennings 2015d, and Jennings 2015e are from a single paper that describes five embedded recruitment trials.

We report the results of studies rated as being at low or unclear risk of bias here. The full list of strategies (irrespective of risk of bias) is given in Supplementary material 8; participant numbers per study are presented in Supplementary material 9; and the data and analyses are in Supplementary material 10.

We produced summary of findings (SoF) tables for all strategies investigated by at least two studies, for a total of 15 SoF tables (Table; Table; Table; Table; Table; Table; Table; Table; Table; Table; Table; Table; Table; Table; Table). Category E strategies (i.e. studies investigating modifications to the information given to potential participants about the trial) dominated.

We detected substantial heterogeneity in four comparisons. Whilst we planned to undertake subgroup analyses to explore plausible explanations for heterogeneity, there were too few studies to allow subgroup analyses for three of these comparisons. We conducted a subgroup analysis for the comparison of monetary incentives to no incentives, exploring the effect of the type of allocation concealment used, which is presented as Analysis 63.2 and described below.

#### Category A: design

##### Overview

Seven studies (9625 participants) evaluated five trial design comparisons. High‐certainty evidence from three pooled studies shows that an open‐label trial design significantly increases recruitment compared to a blinded, placebo‐controlled design (RD 10%). Conversely, single‐study evidence for other strategies, including patient preference designs and internet‐based data collection, is of low certainty or remains insufficient to facilitate GRADE assessments due to small sample sizes and wide confidence intervals. None of the included studies reported cost or retention data for any of these strategies. We describe results for the individual comparisons below.

###### Open‐label versus blinded, placebo‐controlled design

An open‐label design compared to a blinded, placebo‐controlled design results in a large increase in recruitment: RD = 10% (95% CI 8% to 12%); GRADE: high; Analysis 1.1; Summary of findings table 1. This is based on three studies (9004 participants): Avenell 2004 [36] (fracture prevention), RoB: low; Hemminki 2004 [37] (postmenopausal hormone therapy), RoB: low; and Veerus 2016 [38] (postmenopausal hormone therapy), RoB: unclear. This result and its GRADE assessment apply mainly to women (although one study did involve some men) in the UK and Estonia. Applicability to other populations is unclear because the authors provided no further participant characteristics. For other populations, certainty would be downgraded for indirectness. None of the studies reported cost or retention data.

###### Patient preference randomisation versus conventional randomised allocation

A patient preference design increased total participation but may have resulted in little or no difference to recruitment to the randomised trial: RD = −4% (reduced recruitment) (95% CI −15% to 7%); GRADE: low (−2 levels: imprecision – single study; wide CI crossing the line of no effect); Analysis 2.1. This is based on single‐study evidence (273 participants): Cooper 1997 [39] (management strategies for heavy menstrual bleeding), RoB: low. This result and its GRADE assessment apply mainly to higher‐income women in the UK (Scotland); applicability to other populations is unclear because the authors provided no further participant characteristics. For other populations, certainty would be downgraded for indirectness. Cooper 1997 did not report cost or retention data.

###### Electronic data capture versus paper‐based data capture

Internet‐based, electronic data collection compared to paper‐based data capture may reduce recruitment: RD = −13% (95% CI −24% to −3%); GRADE: low (−1 level: study limitations – unclear RoB; −1 level: imprecision – single study); Analysis 3.1. This is based on single‐study evidence (28 practices and 80 participants involved): Litchfield 2005 [40] (delivery systems for insulin), RoB: unclear. This result and GRADE assessment apply only to people living in the UK. Because participant characteristics were insufficiently reported, applicability to other populations is unclear. For other populations, certainty would be downgraded for indirectness. Litchfield 2005 did not report cost or retention data.

###### Cluster‐randomised design versus Zelen design

Compared to a Zelen design, a cluster‐randomised design may increase recruitment (6/24 recruited in the cluster arm versus 0/29 in the Zelen arm). This is based on single‐study evidence involving mostly older people receiving end of life care in the UK (Wales; two cluster sites and 53 participants): Fowell 2006 [41] (palliative care), RoB: low; comparison 4A, no forest plot available, Supplementary material 10. We were unable to generate a forest plot or complete a GRADE assessment for this comparison due to lack of data. Fowell 2006 did not report cost or retention data.

###### Two‐stage randomisation to choose duration of treatment versus single randomisation

A single, upfront randomisation to three or six months of treatment may increase recruitment compared to delayed, two‐stage randomisation to decide whether a second 3‐month treatment should be given (5.21 versus 4.09 participants per year, per centre). Data on numbers recruited were not available for one arm. This is based on single‐study evidence involving people living in the UK (215 participants): Paul 2011 [42] (adjuvant treatment for colorectal cancer), RoB: low; comparison 5A, no forest plot available, Supplementary material 10. We were unable to generate a forest plot or complete a GRADE assessment for this comparison due to lack of data. Paul 2011 did not report cost or retention data.

#### Category B: pre‐trial planning

##### Overview

There was only one study evaluating a single comparison assessing the effect of pre‐trial planning on improving recruitment. The study involved 33 healthcare organisations.

###### Pre‐engagement with potential trial sites during proposal development versus no pre‐engagement

Pre‐engagement with potential trial sites during proposal development may increase recruitment to the randomised trial: RD = 50% (95% CI 18% to 81%); GRADE: low (−1 level: study limitations – unclear RoB; −1 level: imprecision – single study); Analysis 6.1. This is based on single‐study evidence: Garvelink 2015 [43] (frailty), RoB: unclear. This result and its GRADE assessment apply to directors of health and social services centres in Canada; applicability to other populations is unclear because the authors provided no further participant characteristics. For other populations, certainty would be downgraded for indirectness. Garvelink 2015 did not report cost or retention data.

#### Category C: trial conduct changes

##### Overview

Six studies (6592 participants) evaluated five conduct‐related strategies. High‐certainty evidence from two pooled studies shows that telephone reminders to non‐responders increase recruitment (RD 6%), although this specifically applies to trials with low underlying recruitment. Conversely, single‐study evidence of moderate certainty suggests that SMS messages mentioning trial scarcity (RD 3%) or including participant quotes (RD 4%) probably increase recruitment. Single‐study evidence also indicates that email and postal invitations perform similarly. While single‐study evidence suggests telephone reminders probably improve recruitment more than SMS, they involve higher costs (GBP 3.64 incremental cost per participant) with statistically non‐significant effectiveness. None of the studies reported retention data. We describe results for the individual comparisons below.

###### Telephone reminder versus no telephone reminder

Using a telephone reminder to contact non‐responders to a postal invitation increases recruitment: RD = 6% (95% CI 3% to 9%); GRADE: high; Analysis 7.1; Table. This is based on two studies (1450 participants): Nystuen 2004 [44] (getting people to return to work), RoB: low; Wong 2013 [45] (colorectal cancer), RoB: low. **Note:** the evidence for this intervention comes entirely from trials with low (< 10%) underlying recruitment. When applied to trials with higher recruitment, we would downgrade the evidence to moderate certainty for indirectness. In addition, this result and its GRADE assessment apply to people with a mean age of 58 years living in Canada and Norway; applicability to other populations is unclear because the authors provided no further participant characteristics. For other populations, certainty would be further downgraded for indirectness. Neither study reported cost or retention data.

###### SMS reminder mentioning scarcity versus SMS reminder with no mention

Mentioning the scarcity of trial places in SMS messages probably increased recruitment: RD = 3% (95% CI 1% to 6%); GRADE: moderate (−1 level: imprecision – single study); Analysis 8.1. This is based on single‐study evidence (1862 participants): Free 2011 [46] (smoking cessation), RoB: low. This result and its GRADE assessment apply to younger people living in the UK; applicability to other populations is unclear because the authors provided no further participant characteristics. For other populations, certainty would be downgraded for indirectness. Free 2011 did not report cost or retention data.

###### SMS messages containing quotes from existing participants versus no messages

Giving quotes from previous participants in SMS messages probably increased recruitment: RD = 4% (95% CI 2% to 6%); GRADE: moderate (−1 level: imprecision – single study); Analysis 9.1. This is based on single‐study evidence (811 participants): Free 2010 [47] (smoking cessation), RoB: low. This result and its GRADE assessment apply to younger people living in the UK; applicability to other populations is unclear because the authors provided no further participant characteristics. For other populations, certainty would be downgraded for indirectness. Free 2010 did not report cost or retention data.

###### Email invitation versus postal invitation

Using email invitations probably made little or no difference to recruitment compared to postal invitations: RD = 1% (95% CI −3% to 4%); GRADE: moderate (−1 level: imprecision – single study); Analysis 10.1. This is based on single‐study evidence (1760 participants): Treweek 2012 [48] (antibiotic prescribing by GPs), RoB: low. This result and its GRADE assessment apply to general practitioners living in the UK (Scotland); applicability to other populations is unclear because the authors provided no further participant characteristics. For other populations, certainty would be downgraded for indirectness. Treweek 2012 did not report cost or retention data.

###### Telephone reminders versus SMS reminders

Using telephone reminders probably increases recruitment compared to SMS reminders. RD = 5% (95% CI −1% to 11%); GRADE: moderate (−1 level: imprecision – single study); Analysis 11.1. This is based on single‐study evidence (709 participants): Bracken 2019 [49] (testosterone for type 2 diabetes), RoB: low. This result and its GRADE assessment apply to middle‐aged men living in Australia; applicability to other populations is unclear because the authors provided no further participant characteristics. For other populations, certainty would be downgraded for indirectness. Telephone reminders, versus SMS reminders, have an estimated ICER of GBP 72.72, with incremental costs of GBP 3.64 (consisting of inflation‐adjusted implementation, i.e. landline charges per call for telephone reminders versus bulk prices for SMS reminders and staff time costs) and an incremental effectiveness of 5%. However, the effectiveness of this strategy is not statistically significant. Bracken 2019 did not report retention data.

#### Category D: modification to the consent process

##### Overview

Five studies evaluated five comparisons assessing the effect of modifying the consent process. We present only three studies (482 participants) here as two were at high risk of bias. Single‐study evidence of low certainty suggests that opt‐out consent may improve recruitment (RD 19%), based on a study of older, affluent participants. Single‐study evidence for other strategies, such as having a researcher read consent details or using easy‐to‐read forms, remains very uncertain or insufficient to undertake GRADE assessments. None of the included studies reported cost or retention data. We describe results for the individual comparisons below.

###### Opt‐out consent versus opt‐in consent

Opt‐out consent may improve recruitment: RD = 19% (95% CI 3% to 35%); GRADE: low (−1 level: study limitations – unclear RoB; −1 level: imprecision – single study); Analysis 12.1. This is based on single‐study evidence (152 participants): Trevena 2006 [50] (colorectal cancer), RoB: unclear. This result and its GRADE apply mainly to better‐off males and females above the age of 50 living in Australia; applicability to other populations is unclear because the authors provided no further participant characteristics. For other populations, certainty would be downgraded for indirectness. Trevena 2006 did not report cost or retention data.

###### Researcher reading out consent versus participant reading consent

It is very uncertain whether a researcher reading out the consent details affects recruitment: RD = 6% (95% CI −13% to 25%); GRADE: very low (−1 level: study limitations – unclear RoB; −2 levels: imprecision – single study; wide CI crossing the line of no effect); Analysis 13.1. This is based on single‐study evidence (104 participants): Wadland 1990 [51] (smoking cessation), RoB: unclear. This result and its GRADE assessment apply to people living in the USA; applicability to other populations is unclear because the authors provided no further participant characteristics. For other specific groups, an additional GRADE downgrade for indirectness would be warranted, although the certainty of the evidence is already very low. Wadland 1990 did not report cost or retention data.

###### Easy‐to‐read versus standard consent form

An easy‐to‐read consent form may make little or no difference to recruitment compared to standard consent: RD = 3% (P = 0.32); Comparison 14D, Supplementary material 10 (no forest plot available). The authors of this cluster‐randomised trial did not present centre‐level recruitment data, or provide an intracluster correlation coefficient. However, they did consider intracluster correlation in their analysis and found that recruitment did not differ significantly between the two trial groups. This is based on single‐study evidence (226 participants): Coyne 2003 [52] (cancer), RoB: unclear. We were unable to complete a GRADE assessment for this comparison due to lack of data. This result applies mostly to well‐educated white women above the age of 50 years living in the USA. Coyne 2003 did not report cost or retention data.

#### Category E: modification to the information given to potential participants about the trial

##### Overview

Fifty‐two studies evaluated 37 comparisons, with 48 studies included, involving at least 109,968 participants. It was unclear how many participants were involved in two studies, although at least 126 centres were involved. We judged four studies (7.7%) to be at high risk of bias and do not present them here. Single‐study evidence of moderate certainty shows that enclosing a trial‐relevant questionnaire probably increases recruitment (RD 5%). Moderate‐certainty evidence from two pooled studies suggests that providing multimedia information via links or QR codes probably leads to a large increase in recruitment and retention (RD 13%). Conversely, high‐certainty evidence from pooled studies demonstrates that optimised participant information leaflets (PILs)(bespoke and user‐tested) and recruitment primer letters make little to no difference. Moderate‐certainty evidence from pooled studies also suggests that brief or user‐feedback‐optimised PILs do not significantly improve recruitment.

Some strategies may reduce recruitment: moderate‐certainty evidence from two pooled studies indicates that telephone‐based information is less effective than face‐to‐face information (RD −9%). Single‐study evidence of low certainty suggests that handwritten personalisation or ethnicity‐specific risk framing may slightly decrease participation. Evidence for other strategies, such as video‐based information or ethnic‐matched recruiters, remains very uncertain or insufficient to facilitate GRADE assessments. Cost data show that while additional leaflets are low‐cost, video‐based information costs more for lower effectiveness. Most studies lacked cost or retention data. The results for the 33 individual comparisons are described below.

###### Brief participant information leaflet (PIL) versus full PIL

Using a brief patient information leaflet (PIL) probably makes little or no difference to recruitment compared to a full PIL: RD = 0% (95% CI −1% to 2%); GRADE: moderate (−1 level: indirectness, Chen 2011 actually measured entry to pre‐randomisation phase); Analysis 17.1; Table. This is based on two studies (4633 participants): Chen 2011 [53] (clinical area unclear), RoB: low; Brierley 2012 [54] (depression), RoB: low. This result and its GRADE assessment apply to people, especially women, around 40 years old living in the UK. For other populations, certainty would be downgraded for indirectness. Neither study reported cost or retention data.

###### Study‐related questionnaire plus trial invitation versus trial invitation

Enclosing a questionnaire covering issues relevant to the trial with the invitation probably increases recruitment: RD = 5% (95% CI 2% to 8%); GRADE: moderate (−1 level: imprecision – single study); Analysis 18.1. This is based on single‐study evidence (2393 participants): Kendrick 2001 [55] (injury prevention, recruiting family units), RoB: low. This result and its GRADE assessment apply to people living in the UK; applicability to other populations is unclear because the authors provided no further participant characteristics. For other populations, certainty would be downgraded for indirectness. Kendrick 2001 did not report cost or retention data.

###### PIL developed with feedback from users versus usual PIL

Optimised PILs developed with feedback from potential participants or stakeholders, but without formal iterative user‐testing, probably make little or no difference to recruitment: RD = 0% (95% CI 0% to 1%); GRADE: moderate (−1 level: indirectness, Chen 2011 actually measured entry to pre‐randomisation phase); Analysis 19.1; Table. This is based on two studies (16,763 participants): Chen 2011 (clinical area unclear), RoB: low; Cockayne 2017 (START PIL REFORM) [56] (falls prevention), RoB: low. This result and its GRADE assessment apply to older people living in the UK; applicability to other populations is unclear because the authors provided no further participant characteristics. For other populations, certainty would be downgraded for indirectness. None of the studies reported cost or retention data.

###### Enhanced recruitment package plus recruitment at churches versus standard recruitment package

Recruitment at a church and other enhancements, such as having African American recruiters, may result in little to no difference in recruitment: RD = 1% (95% CI 0% to 2%); GRADE: low (−1 level: study limitations – unclear RoB; −1 level: imprecision – single study); Analysis 20.1. This is based on single‐study evidence (6246 participants): Ford 2004 [57] (cancer), RoB: unclear. This result and its GRADE assessment apply to African American men living in the USA; applicability to other populations is unclear because the authors provided no further participant characteristics. For other populations, certainty would be downgraded for indirectness. Ford 2004 did not report cost or retention data.

###### Enhanced versus standard recruitment package

An enhanced recruitment package including more contact may make little or no difference in recruitment: RD = 0% (95% CI −1% to 0%); GRADE: low (−1 level: study limitations – unclear RoB; −1 level: imprecision – single study); Analysis 21.1. This is based on single‐study evidence (6376 participants): Ford 2004 (cancer), RoB: unclear. This result and its GRADE assessment apply to African American men living in the USA; applicability to other populations is unclear because the authors provided no further participant characteristics. For other populations, certainty would be downgraded for indirectness. Ford 2004 did not report cost or retention data.

###### Enhanced recruitment package plus baseline data over telephone versus standard recruitment package

An enhanced recruitment package including more contact by telephone may make little or no difference in recruitment: RD = 0% (95% CI −1% to 1%); GRADE: low (−1 level: study limitations – unclear RoB; −1 level: imprecision – single study); Analysis 22.1. This is based on single‐study evidence (6372 participants): Ford 2004 (cancer), RoB: unclear. This result and its GRADE assessment apply to African American men living in the USA; applicability to other populations is unclear because the authors provided no further participant characteristics. For other populations, certainty would be downgraded for indirectness. Ford 2004 did not report cost or retention data.

###### Audio record of information given about trial versus no audio record

It is very uncertain whether providing an audio record of the discussion about the trial affects recruitment: RD = −3% (reduced recruitment) (95% CI −19% to 13%); GRADE: very low (−1 level: study limitations – unclear RoB; −2 levels: imprecision – single study; wide CI crossing the line of no effect); Analysis 23.1 This is based on single‐study evidence (130 participants): Bergenmar 2014 [58] (cancer), RoB: unclear. This result and its GRADE assessment apply to middle‐aged, mostly female participants with a broad range of educational backgrounds living in Sweden. For other specific groups, an additional GRADE downgrade for indirectness would be warranted, although the certainty of the evidence is already very low. Bergenmar 2014 did not report cost or retention data.

###### Clinical trial booklet plus standard information versus standard information

It is very uncertain whether providing a clinical trial booklet together with standard information affects recruitment: RD = 20% (95% CI −5% to 46%); GRADE: very low (−1 level: study limitations – unclear RoB; −2 levels: imprecision – single study; wide CI crossing the line of no effect); Analysis 24.1. This is based on single‐study evidence (31 participants): Ives 2001 [59] (HIV), RoB: unclear. This result and its GRADE assessment apply to males in their thirties with a good education living in an urban population in the UK. For other specific groups, an additional GRADE downgrade for indirectness would be warranted, although the certainty of the evidence is already very low. Ives 2001 did not report cost or retention data.

###### Total information disclosure versus standard disclosure

It is very uncertain whether providing total information disclosure rather than leaving it to recruiters as to what to reveal affects recruitment: RD = 11% (95% CI −6% to 28%); GRADE: very low (−1 level: study limitations – unclear RoB; −2 levels: imprecision – single study; wide CI crossing the line of no effect); Analysis 25.1. This is based on single‐study evidence (57 participants): Simes 1986 [60] (cancer), RoB: unclear. This result and its GRADE assessment apply to middle‐aged white women whose first language is English, with a high‐school or lower education, who live in Australia. For other specific groups, an additional GRADE downgrade for indirectness would be warranted, although the certainty of the evidence is already very low. Simes 1986 did not report cost or retention data.

###### Bespoke user‐tested PIL versus usual PIL

Optimised PILs (bespoke and user‐tested) tailored for a specific trial and refined through formal user testing make little or no difference to recruitment: RD = 0% (95% CI 0% to 1%); GRADE: high; Analysis 27.1; Table. This is based on six studies (27,805 participants): Cockayne 2017 (START PIL REFORM) (falls prevention), RoB: low; Knapp 2020 (START PIS CASPER) [61] (behavioural activation for depression), RoB: low; Madurasinghe 2021 (START PIS HeLP Diabetes) [62] (type 2 diabetes self‐management), RoB: low; Man 2015 (START PIS Healthlines Depression) [63] (extra support for depression), RoB: low; Parker 2018 (START PIS ECLS) [64] (lung cancer diagnosis), RoB: unclear; Sheridan 2020 (START PIS ISDR) [65] (diabetic retinopathy screening), RoB: high. This result and its GRADE assessment apply to people living in the UK; applicability to other populations is unclear because the authors of most of the studies provided no further participant characteristics. Only one out of the six studies reported EDI data, and participants were mostly older women. For other populations, certainty would be downgraded for indirectness. None of the studies reported cost or retention data for this comparison.

###### Recruitment primer letter versus no letter

Sending a recruitment primer letter/leaflet makes little or no difference to recruitment: RD = 1% (95% CI −1% to 2%); GRADE: high; Analysis 28.1; Table. This is based on two studies (5376 participants): Arundel 2017 [66] (falls prevention), RoB: low; Paul 2014 [67] (colorectal cancer), RoB: low. This result and its GRADE assessment apply mainly to older white people living in the UK and Ireland; applicability to other populations is unclear because the authors provided no further participant characteristics. For other populations, certainty would be downgraded for indirectness. We extracted cost‐effectiveness data from Arundel 2017. Adding a recruitment leaflet has an estimated ICER of GBP 208.38, with incremental costs of GBP 2.08 (consisting of inflation‐adjusted printing, packing, supply, and labelling costs) and a partial incremental effectiveness of 1%. However, the partial effectiveness of this strategy is not statistically significant (RD 0.01, 95% CI −0.01 to 0.02). Neither study reported retention data.

###### Information provided over telephone versus information provided face‐to‐face

Providing information over the telephone rather than face‐to‐face probably reduces recruitment: RD = −9% (reduced recruitment) (95% CI −18% to 1%); GRADE: moderate (−1 level: imprecision – wide CI crossing the line of no effect); Analysis 29.1; Table. This is based on two studies (249 participants): Bobb 2016 [68] (emergency department trials), RoB: low; Foss 2016 [69] (vaccination), RoB: low. This result and its GRADE assessment apply to people, especially women, between the ages of 30 and 60 who have a high degree of education and are living in the USA or Denmark. For other populations, certainty would be downgraded for indirectness. Neither study reported cost or retention data for this comparison.

###### Providing information by video versus standard information

It is very uncertain whether providing trial information by video affects recruitment compared to standard information: RD = −4% (reduced recruitment) (95% CI −23% to 15%); GRADE: very low (−1 level: study limitations – unclear and high RoB; −1 level: inconsistency; −1 level: imprecision – wide CI crossing the line of no effect); Analysis 30.1; Table. This is based on five studies (1634 participants): Du 2008 [70] (lung cancer), RoB: unclear; Du 2009 [71] (breast cancer), RoB: unclear; Hutchison 2007 [72] (cancer), RoB: low; Mattock 2020 [73] (paediatric behavioural difficulties), RoB: high; Rogers 2019 [74] (arthritis), RoB: unclear. This result and its GRADE assessment apply to older, mainly ethnic majority males and females from a broad range of socioeconomic backgrounds living in high‐income countries. For other specific groups, an additional GRADE downgrade for indirectness would be warranted, although the certainty of the evidence is already very low. We extracted cost‐effectiveness data from Rogers 2019. Providing information by video is dominated by standard information, as it is both more costly (with incremental costs of GBP 41.58 consisting of inflation‐adjusted video production, DVD manufacture, packaging, and postage costs) and less effective (with a partial incremental effectiveness of −2%). However, the partial effectiveness of this strategy is not statistically significant (RD: from −0.02, 95% CI −0.07 to 0.04). None of the studies reported retention data for this comparison.

###### Invitation letter emphasising ethnicity‐specific disease risk versus standard letter

An invitation letter that emphases ethnicity‐specific disease risk may reduce recruitment slightly compared to a standard invitation leaflet: RD = −3% (reduced recruitment) (95% CI −12% to 6%); GRADE: low (−2 levels: imprecision – single study; wide CI crossing the line of no effect); Analysis 31.1. This is based on single‐study evidence (445 participants): Brown 2015 [75] (lifestyle intervention for gestational diabetes or pre‐diabetes), RoB: low. This result and its GRADE assessment apply to women over the age of 30 from a range of ethnic groups who live in the USA. For other populations, certainty would be downgraded for indirectness. Brown 2015 did not report cost or retention data.

###### Handwritten personalised invitation letter versus standard letter

A handwritten, personalised invitation leaflet may reduce recruitment compared to a standard invitation leaflet: RD = −4% (reduced recruitment) (95% CI −8% to 0%); GRADE: low (−1 level: study limitations – unclear RoB; −1 level: imprecision – single study); Analysis 32.1. Additionally, a handwritten personalised invitation leaflet may reduce retention compared to a standard invitation leaflet: RD = −4% (reduced retention) (95% CI −8% to 0%); GRADE: low (−1 level: study limitations – unclear RoB; −1 level: imprecision – single study); Analysis 32.2. These findings are based on single‐study evidence (317 participants): McCaffery 2019 [76] (falls prevention), RoB: unclear. This result and its GRADE assessment apply to people living in the UK; applicability to other populations is unclear because the authors provided no further participant characteristics. For other populations, certainty would be downgraded for indirectness. McCaffery 2019 did not report cost data.

###### Different website wording, including infographic, about the risk of underlying condition versus standard wording

Different website wording, including an infographic, about the risk of the underlying condition may make little or no difference to recruitment, compared to standard wording, but the evidence is very uncertain: RD = 0% (95% CI −0.01 to 0.01); GRADE: very low (−2 levels: study limitations – both included studies are high RoB; −1 level: indirectness – ‘visitors’ to the website may not be unique); Analysis 33.1. These findings are based on single‐study evidence involving three arms, published in a single paper (2605 participants): Miller 2021a [77] (falls prevention), RoB: high; and Miller 2021b [78] (falls prevention), RoB: high. This result and its GRADE assessment apply to people aged 70 years and over living in the USA; applicability to other populations is unclear because the authors provided no further participant characteristics. For other specific groups, an additional GRADE downgrade for indirectness would be warranted, although the certainty of the evidence is already very low. Neither study reported cost or retention data.

###### Shorter recruitment package versus standard recruitment package

It is very uncertain whether a shorter recruitment package with standard consent affects recruitment: RD = 2% (95% CI −13% to 17%); GRADE: very low (−1 level: study limitations – unclear RoB; −2 levels: imprecision – single study; wide CI crossing the line of no effect); Analysis 34.1. This is based on single‐study evidence (170 participants): Chow 2020 [79] (self‐management for coronary heart disease following paediatric cancer treatment), RoB: unclear. This result and its GRADE assessment apply to people living in the USA; applicability to other populations is unclear because the authors provided no further participant characteristics. For other specific groups, an additional GRADE downgrade for indirectness would be warranted, although the certainty of the evidence is already very low. Chow 2020 did not report cost or retention data.

###### Shorter recruitment package with simplified consent versus standard recruitment package

It is very uncertain whether a shorter recruitment pack with simplified consent affects recruitment: RD = 2% (95% CI −13% to 17%); GRADE: very low (−1 level: study limitations – unclear RoB; −2 levels: imprecision – single study; wide CI crossing the line of no effect); Analysis 35.1. This is based on single‐study evidence (175 participants): Chow 2020 (self‐management for coronary heart disease following paediatric cancer treatment), RoB: unclear. This result and its GRADE assessment apply to people living in the USA; applicability to other populations is unclear because the authors provided no further participant characteristics. For other specific groups, an additional GRADE downgrade for indirectness would be warranted, although the certainty of the evidence is already very low. Chow 2020 did not report cost or retention data.

###### Leaflet advertising patient and public involvement (PPI) in trial plus standard trial invitation versus standard trial invitation only

Including a leaflet advertising patient and public involvement (PPI) in a trial alongside the standard trial invitation probably makes little or no difference to recruitment: RD = −1% (reduced recruitment) (95% CI −2% to 0%); GRADE: moderate (−1 level: imprecision – single study); Analysis 36.1. This is based on single‐study evidence (8182 participants): Hughes‐Morley 2016 [80] (care planning training for community mental health teams), RoB: low. This result and its GRADE assessment apply to middle‐aged adults, predominantly female and relatively socioeconomically disadvantaged, living in the UK. For other populations, certainty would be downgraded for indirectness. Hughes‐Morley 2016 did not report cost or retention data.

###### Gender‐targeted recruitment postcard versus generic recruitment postcard

Gender‐targeted recruitment postcards may have little or no impact on recruitment compared to generic recruitment postcards: RD = 0% (95% CI −0% to 0%); GRADE: low (−1 level: study limitations – unclear RoB; −1 level: imprecision – single study); Analysis 37.1. This is based on single‐study evidence (30,000 participants): Crane 2016 [81] (behavioural intervention for weight gain prevention), RoB: unclear. This result and its GRADE assessment apply mainly to females who live in the USA. For other populations, certainty would be downgraded for indirectness. Crane 2016 did not report cost or retention data.

###### Researcher‐optimised PIL versus standard PIL

An optimised PIL, modified by the research team to improve readability without direct user input or feedback, may reduce recruitment: RD = −9% (reduced recruitment) (95% CI −17% to 0%); GRADE: low (−2 levels: imprecision – single study; wide CI including the line of no effect); Analysis 38.1. This is based on single‐study evidence (480 participants): Paris 2015 [82] (multiple host trials in different clinical areas, including heart surgery, dementia, diabetes, and sleep disorders), RoB: low. This result and its GRADE assessment apply to middle‐aged people with a broad range of socioeconomic backgrounds who live in France. For other populations, certainty would be downgraded for indirectness. Paris 2015 did not report cost or retention data.

###### Brief PIL plus standard PIL versus standard PIL

Providing a brief PIL alongside the standard PIL may have little or no effect on recruitment: RD = 0% (95% CI −1% to 1%); GRADE: low (−1 level: study limitations – unclear RoB; −1 level: imprecision – single study); Analysis 40.1. This is based on single‐study evidence (6946 participants): White 2023 [83] (community‐based clinic for people with persistent, medically unexplained, physical symptoms), RoB: unclear. This result and its GRADE assessment apply mainly to white British females around the age of 46 with a good education living in the UK. For other populations, certainty would be downgraded for indirectness. We also extracted cost‐effectiveness data from White 2023. Providing a brief PIL alongside the standard PIL, versus standard PIL alone, has an undefined ICER, as the incremental effectiveness (the denominator) is zero. This strategy presents an incremental cost of GBP 0.57 consisting of the inflation‐adjusted cost of adding a brief PIL. Overall, adding a brief PIL alongside the standard PIL results in a small increase in cost with no difference in recruitment. White 2023 did not report retention data.

###### Decision aid about RCTs versus blank notebook

A decision aid about RCTs may result in a large increase in trial recruitment compared to a blank notebook: RD = 12% (95% CI −1% to 24%); GRADE: low (−2 levels: imprecision – single study; wide CI crossing the line of no effect); Analysis 41.1. This is based on single‐study evidence (129 participants): Sundaresan 2017 [84] (radiotherapy for prostate cancer), RoB: low. This result and its GRADE assessment apply to older males with a broad range of countries of birth and levels of education living in Australia and New Zealand. For other populations, certainty would be downgraded for indirectness. Sundaresan 2017 did not report cost or retention data.

###### Interactive online digital educational tool versus time‐matched webinar

An interactive online digital educational tool may make little or no difference to recruitment compared to a time‐matched webinar: RD = 1% (95% CI −1% to 3%); GRADE: low (−1 level: study limitations – unclear RoB; −1 level: imprecision – single study); Analysis 42.1. This is based on single‐study evidence (351 participants): Saif 2020 [85] (Alzheimer's disease), RoB: unclear. This result and its GRADE assessment apply to older people, especially females, from a range of ethnicities (though predominantly white) with varying amounts of education living in the USA. For other populations, certainty would be downgraded for indirectness. Saif 2020 did not report cost or retention data.

###### PPI‐designed and ‐informed PIL versus standard researcher‐designed PIL

It is very uncertain whether a PPI‐designed and ‐informed PIL affects recruitment compared to a standard researcher‐designed PIL: RD = −2% (reduced recruitment) (95% CI −15% to 12%); GRADE: very low (−1 level: study limitations – unclear RoB; −2 levels: imprecision – single study; wide CI crossing the line of no effect); Analysis 43.1. These findings are based on single‐study evidence (207 participants): Dwyer 2022 [86] (cognitive rehabilitation intervention for multiple sclerosis), RoB: unclear. This result and its GRADE assessment apply to people living in Ireland; applicability to other populations is unclear because the authors provided no further participant characteristics. For other groups, an additional GRADE downgrade for indirectness would be warranted, although the certainty of the evidence is already very low. Additionally, it is very uncertain whether a PPI‐designed and ‐informed PIL affects retention compared to a standard researcher‐designed PIL: RD = 4% (reduced retention) (95% CI −9% to 18%); GRADE: very low (−1 level: study limitations – unclear RoB; −2 levels: imprecision – single study; wide CI crossing the line of no effect); Analysis 43.2. Dwyer 2022 did not report cost data.

###### Multimedia education about clinical trials (DVD plus booklet) versus standard educational booklet only

Providing multimedia education about clinical trials via a DVD and booklet may increase recruitment: RD = 4% (95% CI −5% to 14%); GRADE: low (−2 levels: imprecision – single study; wide CI crossing the line of no effect); Analysis 45.1. This is based on single‐study evidence (418 participants): Kamen 2018 [87] (oncology), RoB: low. This result and its GRADE assessment apply to older people, especially females, who are predominantly white, with a range of incomes and levels of education, living in the USA. For other populations, certainty would be downgraded for indirectness. Kamen 2018 did not report cost or retention data.

###### Multimedia information (viewed in person) plus paper PIL versus paper PIL alone

It is very uncertain whether providing multimedia information (viewed in person) alongside a paper PIL increases recruitment: RD = 17% (95% CI −89% to 123%); GRADE: very low (−1 level: inconsistency; −2 levels: imprecision – extremely wide CI crossing the line of no effect); Analysis 47.1; Table. Additionally, it is very uncertain whether providing multimedia information (viewed in person) alongside a paper PIL affects retention compared to a paper PIL only: RD = 28% (95% CI −79% to 136%); GRADE: very low (−1 level: inconsistency; −2 levels: imprecision – extremely wide CI crossing the line of no effect); Analysis 47.2. These findings are based on three studies (23 participants): Knapp 2023a (BALANCE) [88] (paediatric lazy eye), RoB: low; Knapp 2023b (BAMP) [89] paediatric underbite), RoB: low; Knapp 2023f (UKALL) [90] paediatric and adolescent leukaemia or lymphoma), RoB: low. This result and its GRADE assessment apply to non‐white male and female children aged between six and 13 years, living in the UK, mainly in disadvantaged areas. For other specific groups, an additional GRADE downgrade for indirectness would be warranted, although the certainty of the evidence is already very low. None of the studies reported cost data for this comparison.

###### Multimedia information (viewed in person) PIL versus paper PIL

Providing a multimedia information PIL (viewed in person) probably increases recruitment slightly compared to a paper PIL: RD = 4% (95% CI −1% to 8%); GRADE: moderate (−1 level: imprecision); Analysis 48.1; Table. These findings are based on three studies (1428 participants): Knapp 2023a (BALANCE) paediatric lazy eye), RoB: low; Knapp 2023b (BAMP) paediatric underbite), RoB: low; Knapp 2023d (FORCE) [91] paediatric wrist fracture), RoB: low. This result and its GRADE assessment apply to male and female children aged between six and 12 years, of mixed ethnicities, living in the UK, mainly in disadvantaged areas. For other populations, certainty would be downgraded for indirectness. Additionally, it is very uncertain whether providing multimedia information (viewed in person) affects retention compared to a paper PIL: RD = 22% (95% CI −51% to 96%); GRADE: very low (−2 levels: imprecision – extremely wide CI crossing the line of no effect; −1 level: inconsistency); Analysis 48.2. None of the studies reported cost data for this comparison.

###### Multimedia information (provided as link/QR code) plus paper PIL versus paper PIL

Providing multimedia information in the form of a link or QR code alongside a paper PIL makes little or no difference to recruitment: RD = 0% (95% CI −1% to 1%); GRADE: high; Analysis 49.1; Table. This is based on seven studies (11,612 participants): Jolly 2019 (START MMI PSM‐COPD) [92] (COPD), RoB: low; Knapp 2023e (Thermic‐3) [93] (paediatric congenital heart defects), RoB: low; Knapp 2023c (CHAMP‐UK) [94] (paediatric short‐sightedness), RoB: low; Madurasinghe 2023a (START MMI GHT2000a) [95] (exercise for high blood pressure), RoB: low; Madurasinghe 2023b (START MMI GHT2000b) [96] (exercise for high blood pressure), RoB: low; Madurasinghe 2023c (START MMI HI‐Light) [97] (vitiligo), RoB: low; Madurasinghe 2023d (START MMI seAFOod) [98] (bowel cancer prevention), RoB: low. This result and its GRADE assessment apply to people living in the UK, some of whom were male and female children between the ages of nine months and nine years with mixed ethnicities; however, reporting in most studies was very limited. For other populations, certainty would be downgraded for indirectness. We also extracted cost‐effectiveness data from Jolly 2019 (START MMI PSM‐COPD). Multimedia information plus paper PIL is dominated by paper PIL only, as it is both more costly (with incremental costs of GBP 0.78 consisting of the intervention inflation‐adjusted unit costs) and less effective (with a partial incremental effectiveness of −1%). However, the partial effectiveness of this strategy is not statistically significant (RD −0.01, 95% CI −0.03 to 0.01). Additionally, providing multimedia information in the form of a link or QR code alongside a paper PIL makes little or no difference to the likelihood of being retained compared to a paper PIL only: RD = 0% (95% CI −2% to 3%); GRADE: high; Analysis 49.2. This is based on five studies (7403 participants): Jolly 2019 (START MMI PSM‐COPD) (COPD), RoB: low; Knapp 2023e (Thermic‐3) (paediatric congenital heart defects), RoB: low; Knapp 2023c (CHAMP‐UK) (paediatric short‐sightedness), RoB: low; Madurasinghe 2023c (START MMI HI‐Light) (vitiligo), RoB: low; Madurasinghe 2023d (START MMI seAFOod) (bowel cancer prevention), RoB: low.

###### Multimedia information (provided as link/QR code) versus paper PIL

Providing multimedia information in the form of a link or QR code probably results in a large increase in recruitment compared to a paper PIL: RD = 13% (95% CI 1% to 26%); GRADE: moderate (−1 level: imprecision – wide CI); Analysis 50.1; Table. This is based on two studies (229 participants): Knapp 2023e (Thermic‐3) (paediatric congenital heart defects), RoB: low; Knapp 2023c (CHAMP‐UK) (paediatric short‐sightedness), RoB: low. This result and its GRADE assessment apply to studies involving children with a range of ethnicities, of both sexes, aged between approximately eight months and nine years, living in the UK, mainly in disadvantaged areas. In one study (Knapp 2023c (CHAMP‐UK), parents were predominantly female, highly educated, and employed. For other populations, certainty would be downgraded for indirectness. Additionally, providing multimedia information in the form of a link or QR code probably results in a large increase in retention (assessed at three months) compared to a paper PIL only. RD = 13% (95% CI 0% to 26%); GRADE: moderate (−1 level: imprecision – wide CI); Analysis 50.2. This is based on two studies: Knapp 2023e (Thermic‐3) (paediatric congenital heart defects), RoB: low; Knapp 2023c (CHAMP‐UK) (paediatric short‐sightedness), RoB: low. None of the studies reported cost data for this comparison.

###### Educational material providing additional information about a trial versus standard information alone

An educational package providing additional information about a trial did not significantly increase recruitment compared to standard information alone (31% of participants aged over 65 in both intervention and control groups in year 2, P = 0.83), comparison 51E, no forest plot available, Supplementary material 10. Although the authors of this cluster‐randomised trial did not present centre‐level recruitment data, or provide an intracluster correlation coefficient, they did consider intracluster correlation in their analysis. This is based on single‐study evidence (126 centres): Kimmick 2005 [99] (cancer), RoB: unclear. We were unable to complete a GRADE assessment for this comparison due to lack of data. This study provided minimal information, and we have therefore not presented the characteristics of included participants. Kimmick 2005 did not report cost or retention data.

###### Trained recruiters from a similar ethnic background to study population already taking part in a trial as lay advocates

Trained recruiters from a similar ethnic background to the trial population acting as lay advocates may increase recruitment compared to untrained recruiters (8/28 trained Hispanic recruiters recruited one or more women versus 0/26 untrained Hispanic women and 2/42 untrained Anglo controls). The authors of this cluster‐randomised trial did not report an analysis that corrected for the clustering or provide an intracluster correlation coefficient. Larkey 2002 [100] reported data at the recruiter aggregate level on whether a recruiter did or did not recruit anyone to the trial. This is based on single‐study evidence (number of participants unclear): Larkey 2002 (unclear), RoB: unclear, comparison 52E, no forest plot available, Supplementary material 10. We were unable to complete a GRADE assessment for this comparison due to lack of data. This study provided minimal information, and we have therefore not presented the characteristics of included participants. Larkey 2002 did not report cost or retention data.

###### Coach to support recruitment of minority participants versus no coach

Using a coach to promote trust among minority participants did not significantly increase trial recruitment compared to usual care. The authors report the number of participants in each arm who went on to be recruited in a trial (16 participants in the intervention group and 13 participants in the control group, P = 0.35). However, we were unable to determine the proportion of participants who were recruited in a trial in each study arm as they did not report the numbers of participants who were randomised to receive coaching versus usual care. This is based on single‐study evidence (75 participants): Fracasso 2013 [101] (cancer), RoB unclear, comparison 53E, no forest plot available, Supplementary material 10. We were unable to complete a GRADE assessment for this comparison due to lack of data. This study provided limited information, and we have therefore not presented the characteristics of included participants. Fracasso 2013 did not report cost or retention data.

#### Category F: interventions aimed at the recruiter or recruitment site

##### Overview

Ten studies evaluated 10 comparisons targeting recruiters or sites. We judged two studies to be at high risk of bias and do not present them here. The remaining eight studies involved at least 5045 participants. It was unclear how many participants were involved in four studies, although 316 clinical sites were involved between them. Single‐study evidence of low certainty shows that remote site initiation visits may increase recruitment (RD 2%) and are more cost‐effective than face‐to‐face visits, saving approximately GBP 42.29 per site. Conversely, single‐study evidence suggests that postcard teaser campaigns (moderate certainty) and educational programmes for ethnic minority recruitment (low certainty) appear to have little or no impact. Other strategies, including recruiter training and site teleconferences, showed mixed results in individual studies but lacked sufficient data to undertake GRADE assessments. Data on cost and retention were rarely reported. We describe the results for the eight individual comparisons below.

###### Teaser campaign using postcards versus no teaser

Using a postcard teaser campaign probably makes little or no difference to recruitment: RD = 0% (95% CI −4% to 5%); GRADE: moderate (−1 level: imprecision – single study); Analysis 54.1 This is based on single‐study evidence (670 participants): Lee 2017 [102] (recruiting GP practices to low back pain trial), RoB: low. This result and its GRADE assessment apply to practices and clinics in Australia; applicability to other populations is unclear because the authors provided no further participant characteristics. For other populations, certainty would be downgraded for indirectness. Lee 2017 did not report cost or retention data.

###### Educational programme to tailor recruitment strategies for minority recruitment versus usual recruitment

Using an educational programme to tailor recruitment for ethnic minority recruitment may have little or no impact on recruitment: RD = 0% (95% CI −4% to 3%); GRADE: low (−1 level: study limitations – unclear RoB; −1 level: imprecision – single study); Analysis 55.1. This is based on single‐study evidence (2107 participants): Tilley 2021 [103] (various host trials: atrial fibrillation, blood and marrow transplants, colon cancer prevention, Parkinson’s), RoB: unclear. This result and its GRADE assessment apply to Latinx white people, especially those who speak English, who live in the USA. For other populations, certainty would be downgraded for indirectness. Tilley 2021 did not report cost or retention data.

###### Remote versus face‐to‐face site initiation visits

Using remote site initiation visits probably increases recruitment compared to face‐to‐face site initiation visits: RD = 2% (95% CI −5% to 10%); GRADE: moderate (−1 level: imprecision – single study); Analysis 56.1. This is based on single‐study evidence (688 participants): Jefferson 2018 [104] (cast versus surgery for wrist fracture), RoB: low. This result and its GRADE apply to the UK; applicability to other populations is unclear because the authors provided no further participant characteristics. For other populations, certainty would be downgraded for indirectness. We also extracted cost‐effectiveness data from Jefferson 2018. Remote visits dominate face‐to‐face site initiation visits, as they are both less costly (with inflation‐adjusted cost savings of GBP 42.29, taking into consideration travel costs and the average cost of setting up a site) and more effective (with an incremental effectiveness of 6%). However, the effectiveness of this strategy is not statistically significant. Additionally, remote site initiation visits may make little or no difference to retention compared to face‐to‐face site initiation visits: RD = 1% (95% CI −6% to 9%); GRADE: low (−2 levels: imprecision – single study; wide CI crossing the line of no effect); Analysis 56.2.

###### Recruitment coordinator plus training versus usual recruitment

Maxwell 2017 [105] compared having a recruitment coordinator conducting teleconferences with site teams to enhance trial recruitment using software for identifying eligible participants (with a second teleconference after six months) to standard recruitment. A stepped wedge approach was used to evaluate this intervention; raw figures at each step were not provided, so it was not possible to calculate a risk difference. The authors found an adjusted rate ratio of 1.06 (95% CI 0.55 to 2.03, P = 0.87), suggesting that the intervention may make little to no difference to recruitment. This is based on single‐study evidence (72 hospital sites): Maxwell 2017 (antiplatelet drugs following stroke), RoB: low, comparison 57F, no forest plot available, Supplementary material 10. We were unable to complete a GRADE assessment for this comparison due to lack of data. This study provided limited information, and we have therefore not presented the characteristics of included participants. Maxwell 2017 did not report cost or retention data.

###### Teleconference and commitment contracts with site teams versus no teleconference

Isaksson 2025 [106] compared using teleconference and commitment contracts with site teams to enhance recruitment versus standard recruitment. A stepped wedge approach was used to evaluate this intervention; raw figures at each step were not provided, so it was not possible to calculate a risk difference. The authors found that the intervention increased recruitment from 1.9 participants/centre/60 days to 2.1 participants/centre/60 days – a 10% increase. This is based on single‐study evidence (20 tertiary care centres): Isaksson 2025 (stroke recovery), RoB: unclear, comparison 58F, no forest plot available, Supplementary material 10. We were unable to complete a GRADE assessment for this comparison due to lack of data. This study provided limited information, and we have therefore not presented the characteristics of included participants. Isaksson 2025 did not report cost or retention data.

###### On‐site initiation visit versus no initiation visit

For this on‐site initiation visit strategy, the authors did not present the proportion of eligible participants recruited, only the number recruited: visited sites recruited 302 participants while those not receiving visits recruited 271. This is based on single‐study evidence (573 participants): Liénard 2006 [107] (breast cancer), RoB: low, comparison 59F, no forest plot available, Supplementary material 10. We were unable to complete a GRADE assessment for this comparison due to lack of data. This study provided limited information, and we have therefore not presented the characteristics of included participants. Liénard 2006 did not report cost or retention data.

###### Additional communication from central trial coordinator to sites versus standard communication

Monaghan 2007 [108] investigated different levels of communication between the trial coordination team and participating sites, comparing the effect of additional communication strategies such as tailored feedback on recruitment. The median total number of participants in the additional communication group was 37.5, compared to 37.0 in the standard communication group. Intervention centres achieved half their recruitment targets in 4.4 months, compared to 5.8 months for control centres. This is based on single‐study evidence (167 clinical sites): Monaghan 2007 (diabetes), RoB: low, comparison 60F, no forest plot available, Supplementary material 10. We were unable to complete a GRADE assessment for this comparison due to lack of data. This study provided limited information, and we have therefore not presented the characteristics of included participants. Monaghan 2007 did not report cost or retention data.

###### Recruiter training with ongoing support and/or personalised nudge versus standard training and support

Agni 2022 [109] investigated the effect of recruiter training with ongoing support and/or theory‐informed personalised nudge to the recruiter compared to standard recruiter support and training. The authors did not collect the number of eligible participants approached in the study. Thus, it was not possible to calculate a risk difference for this intervention. The total number of participants recruited in the enhanced Trainee Principal Investigator (TPI)‐only arm was 279; 147 in the digital nudge‐only arm; 410 for the arm which received both interventions; and 379 in the control arm. The authors concluded that the enhanced TPI education intervention improved recruitment (incident rate ratio (IRR) 1.23, 95% CI 1.09 to 1.40, P = 0.001) – but that the digital nudge did not improve recruitment (IRR 0.89, 95% CI 0.79 to 1.01, P = 0.07). This is based on single‐study evidence (1215 participants): Agni 2022 (partial hip replacement following hip fracture), RoB: low, comparison 61F, no forest plot available, Supplementary material 10. We were unable to complete a GRADE assessment for this comparison due to lack of data. This study provided limited information, and we have therefore not presented the characteristics of included participants. Agni 2022 did not report cost or retention data.

#### Category G: incentives

##### Overview

Twelve studies involving 4934 participants evaluated three incentive types. This included one publication involving five Studies Within A Trial (SWATs) of the same strategy, and another involving two SWATs testing three different comparisons each. Pooled evidence of low certainty indicates that monetary incentives increase recruitment (RD = 4%), particularly at higher values. The estimated cost‐effectiveness ratio is GBP 3052.96 per additional recruit. It remains very uncertain whether higher monetary amounts are more effective than lower ones, as this single‐study evidence (across two trials) is statistically non‐significant. Moderate‐certainty evidence from three pooled studies shows that trial‐branded pens make little or no difference to recruitment or retention, representing a small cost (GBP 0.45) with no gain in effectiveness. Retention data were rarely reported. We describe the results for the 12 studies below.

###### Monetary incentive versus no incentive

Monetary incentives offered to potential participants may improve recruitment compared to no incentive: RD = 5% (95% CI 1% to 9%); GRADE: low (−1 level: study limitations high RoB; −1 level: inconsistency); Analysis 63.1; Table. This is based on 12 studies (5082 participants), including five trials within a single published paper, and two 3‐arm trials within a single published paper: Fairhurst 2022 [110] (yoga for older adults with multimorbidity), RoB: high; Free 2010 (smoking cessation), RoB: low; Giguere 2015 [111] (recruiting family physicians to a medical education trial), RoB: low; Jennings 2015a [112]; Jennings 2015b [113]; Jennings 2015c [114]; Jennings 2015d [115]; Jennings 2015e [116] (primary care, older people, mainly hypertension), RoB: low; Halpern 2021a ($200) [117] and Halpern 2021a ($500) [118] (smoking cessation), RoB: high; Halpern 2021b ($100) [119] and Halpern 2021b ($300) [120] (ambulation), RoB: high. **Note:** the evidence mainly comes from high incentive amounts. The comparisons are shown split by incentive value, grouped into incentives up to GBP 5 (two studies), around GBP 100 (six studies), and over GBP 100 (three studies, one providing two comparisons). This result and its GRADE assessment apply to people of both sexes aged from their thirties to mid‐sixties, probably mostly white ethnicity (but good Black/African American involvement in two studies), and a reasonable spread of socioeconomic statuses living in Canada, the UK, and the USA. For other populations, certainty would be downgraded for indirectness.

As this was the only comparison with 10 or more studies available, we created a funnel plot, which showed a fairly symmetrical distribution, suggesting that publication bias is unlikely (Figure). The symmetry indicates that small and large studies report similar effects, and studies with negative or non‐significant results do not appear to be systematically missing.

**4:**
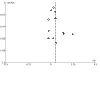
Funnel plot for participant recruitment for the comparison of monetary incentives versus no monetary incentives (category G strategy)

A subgroup analysis to investigate variations in allocation concealment as a potential source of heterogeneity separated the studies into two groups: those with adequate allocation concealment (eight studies) and those with inadequate or unclear allocation concealment (two 3‐arm studies); Analysis 63.2. There was no statistical evidence of a difference in the recruitment effect between studies with adequate allocation concealment (RD = 3%, 95% CI 0% to 5%) and those with inadequate or unclear concealment (RD = 10%, 95% CI −2% to 22%). Although the point estimate was larger in studies with inadequate or unclear concealment, the test for subgroup differences was not statistically significant (Chi² = 1.41, df = 1, P = 0.24, I² = 29%).

We extracted cost‐effectiveness data from all 10 studies included in this comparison. Monetary incentives, versus no incentives, have an estimated ICER of GBP 3052.96, with incremental costs of GBP 152.65 (consisting of the weighted‐average and inflation‐adjusted amount of the monetary incentive which varied from GBP 5 to USD 500 (approximately GBP 340) across the included studies) and an incremental effectiveness of 5%. The effectiveness of this strategy is also statistically significant. Nevertheless, due to the substantial heterogeneity in the size of the monetary incentives offered, we conducted sensitivity analyses by different levels of monetary incentive (low: up to GBP 5; medium: around GBP 100; high: above GBP 100) to assess the robustness of these findings; the results can be found in Table 7a in Supplementary material 12.

None of the studies reported retention data for this comparison.

###### Higher versus lower monetary incentive

It is very uncertain whether offering a higher value of monetary incentive affects recruitment compared to a lower monetary incentive: RD = 3% (95% CI −13% to 19%); GRADE: very low (−2 levels: study limitations – high RoB; −1 level: inconsistency; −1 level: imprecision – wide CI crossing the line of no effect); Analysis 64.1; Table. This is based on two studies, which are both trials within a single published article (864 participants): Halpern 2021a ($200) versus Halpern 2021a ($500) (smoking cessation), RoB: high; Halpern 2021b ($100) versus Halpern 2021b ($300) (ambulation), RoB: high. This result and its GRADE assessment apply to middle‐aged males and females, with a range of ethnicities and socioeconomic statuses, living in the USA. For other specific groups, an additional GRADE downgrade for indirectness would be warranted, although the certainty of the evidence is already very low. We extracted cost‐effectiveness data from both studies included in this comparison. Changing monetary incentives has an estimated ICER of GBP 8214.83, with incremental costs of GBP 246.44 (consisting of the weighted‐average and inflation‐adjusted differences in monetary incentives between the intervention and the comparison groups evaluated from the two included studies) and an incremental effectiveness of 3%. However, the effectiveness of this strategy is not statistically significant. None of the studies reported retention data for this comparison.

###### Non‐monetary incentive (branded pen versus no pen)

Offering a non‐monetary incentive in the form of a trial‐branded pen alongside the trial invitation probably has little or no effect on recruitment: RD = 0% (95% CI −1% to 1%); GRADE: moderate (−1 level: study limitations – high and unclear RoB); Analysis 65.1; Table. This is based on three studies (9640 participants): Fairhurst 2022 (yoga for elderly patients with multimorbidity), RoB: high; White 2023 (community‐based clinic for patients with persistent, medically unexplained, physical symptoms), RoB: unclear; Whiteside 2019 [121] (falls prevention), RoB: low. This result and its GRADE assessment apply to people in the UK, probably in both urban and rural settings; applicability to other populations is unclear because the authors provided no further participant characteristics. For other populations, certainty would be downgraded for indirectness. We extracted cost‐effectiveness data from all three studies. Trial branded pens, versus no pen, have an undefined ICER, as the difference in effectiveness (the denominator) is zero. This strategy presents an incremental cost of GBP 0.45, consisting of the inflation‐adjusted branded pen costs. Overall, trial‐branded pens result in a small increase in cost with no difference in recruitment. Only one of the three included studies reported retention results (Whiteside 2019), which showed that offering a non‐monetary incentive in the form of a trial‐branded pen alongside the trial invitation probably has little or no effect on retention (at three months): RD = 0% (95% CI −2% to 2%); GRADE: moderate (−1 level: imprecision – single study); Analysis 65.2. This is based on one study: Whiteside 2019 (falls prevention), RoB: low.

## Discussion

### Summary of main results

Researchers looking to the literature for evidence‐informed recruitment strategies will find an expanding body of studies that continue to offer plenty of variety but little depth. Despite the large number of SWATs, the evidence remains fragmented and methodologically inconsistent; much of it relies on single‐study evidence, limiting opportunities for meta‐analysis and preventing the generation of high‐certainty findings. As a result, the literature continues to offer more questions than answers. We assessed five strategies as having high‐certainty evidence using the GRADE framework, only two of which improve recruitment (using an open‐label trial design compared to a blinded design and using a telephone reminder to contact non‐responders to postal invitations). Both have challenges associated with their use. Blinding is used to support internal validity, which means switching to an open‐label trial as a recruitment intervention is an unlikely proposition in most cases. Contacting non‐responders is widely considered to be cold‐calling, and ethics committees often rule against it. The three other strategies with high‐certainty evidence – optimised participant information leaflet (PIL; bespoke and user‐tested), sending a recruitment primer letter to participants before they are formally invited to a trial, and providing multimedia information via a link or QR code alongside a paper PIL – all make little or no difference to recruitment.

We assessed 13 strategies as having moderate‐certainty evidence according to GRADE criteria, mostly embedded within single, well‐conducted studies. We assessed the evidence for all other comparisons as being of low or very low certainty, or as at high risk of bias if insufficient data were available to apply the GRADE criteria. There is only one evaluation of an intervention used pre‐trial to support recruitment (category B), rated as low‐certainty evidence, and no evaluations of a consent‐related intervention (category D) with a GRADE rating above low‐certainty evidence.

#### Little or no effect for optimised PILs as a recruitment strategy

Our review provides high‐certainty evidence that optimised PILs (bespoke and user‐tested) do not improve trial recruitment rates. While enhancing clarity is valuable, the effect on the initial decision to enrol is minimal, suggesting several underlying mechanisms.

The lack of recruitment efficacy may stem from potential participants not fully engaging with the PIL. The leaflet may not be the critical component in their decision: Dwyer 2022, for instance, found that 89.3% of participants in their SWAT reported deciding to consent before receiving the PIL, despite acknowledging its formal role. This suggests factors like rapport with the clinical team or general attitudes toward research often dominate the enrolment decision, making the content of PILs largely inconsequential to recruitment success.

Alternatively, the benefits of PIL optimisation may lie elsewhere, primarily in improved participant understanding, and the quality of informed consent. Evidence supports the idea that user‐testing enhances comprehension, though this effect does not translate into better recruitment [122]. Therefore, while PIL optimisation is an ethical and regulatory necessity, trial teams should not rely on it as a strategy to boost participant recruitment. This is consistent with principles of shared decision‐making and the function of decision aids [123, 124]: these tools improve knowledge and reduce post‐trial regret, but their core purpose is simply to clarify the decision for individuals, ensuring participation is the right choice for them.

#### Cost‐effectiveness

Our findings highlight a persistent and significant gap in the evidence on the cost‐effectiveness of recruitment strategies, primarily due to limited reporting of cost data. Of the 91 SWATs included in this review, only 17 reported cost outcomes, which allowed for cost‐effectiveness estimates in just nine out of the 65 comparisons. In six of these nine comparisons, the analysis was based on a single study – either because only one study was available for the comparison, or because other studies in the same comparison did not report cost data. Such evidence gaps result in uncertain and limited cost‐effectiveness findings.

The cost‐effectiveness analysis showed wide variation across recruitment strategies, with ICERs ranging from GBP 72.72 (telephone versus SMS reminders) to GBP 8214.83 (monetary incentive value change) per additional participant recruited. These estimates are uncertain due to few contributing studies and wide confidence intervals of incremental effectiveness.

Monetary incentives were the only strategy with a statistically significant incremental effect (ICER: GBP 3052.96). However, the cost‐effectiveness results remain uncertain, as the sensitivity analyses showed large variation in ICER values (from GBP 229.33 for an incentive of GBP 5 to GBP 4279.82 for an incentive of GBP 100). Additionally, none of the subgroup incremental effects were statistically significant, which limits the generalisability of these findings. The ICER uncertainty is further compounded by inconsistent cost data reporting across studies; for example, some omitted detailed breakdowns, and others did not account for staff time, which collectively hindered accurate comparisons. To improve this, clear guidance is needed for costing SWAT recruitment strategies. We support the PROMETHEUS programme’s call for streamlined economic evaluations alongside all SWATs to strengthen the evidence base and inform policy [125]. Finally, the absence of variability measures (e.g. standard deviations) surrounding the reported costs, and consequently, the estimated incremental costs, introduces persistent uncertainty into the cost‐effectiveness results presented in our review.

#### Impact of recruitment on retention rates

Retention outcomes were reported in 15 SWATs. Only four reported retention at the host trial’s primary outcome time point, while the remaining 11 reported retention at another time point. Overall, the evidence base is limited and highly uncertain, with most comparisons relying on single studies and wide confidence intervals around the estimated effects. Future recruitment SWATs should aim to report retention at the primary outcome time point of the host trial to align with core retention outcome standards and improve comparability. We recognise that waiting until the primary time point may in some cases lead to an unacceptable delay in publishing the recruitment results, and that retention at other time points may be the best that can be included. Regardless, even when the primary aim is recruitment, researchers should routinely aim to collect and report retention data, given the potential for unintended effects on participant follow‐up and engagement. Multimedia information provided to potential participants, particularly when delivered via link or QR code, shows some potential for improving retention and warrants further investigation through replication.

#### Equity, diversity, and inclusion (EDI)

The evidence identified in this review was heavily concentrated in high‐income countries in the Global North. Overall, 99% of included studies were conducted in these settings, with 76% coming from the UK or USA. Only one study included participants from middle‐income countries (India, Indonesia, Malaysia, and the Philippines). Consequently, none of the five recruitment strategies identified with high‐certainty evidence have been robustly tested in the Global South or low‐income settings. This stark geographic imbalance significantly limits the transferability of our findings to these contexts, where recruitment barriers, healthcare infrastructures, and cultural norms may differ substantially.

A key limitation of the included trials is the unclear and inconsistent reporting of participant sex and gender. While we recorded whether trial teams explicitly used the terms 'sex', 'gender', or both, it is difficult to determine if these terms were used accurately or interchangeably in some articles. Only six of the included studies explicitly reported both sex and gender, which suggests that these specific teams at least recognised the need to distinguish between the two. However, most studies were ambiguous on this point. Our data extraction process sometimes involved changing the recorded variable (e.g. correcting 'sex' to 'gender' or vice versa) depending on whether the primary authors referred to social/cultural roles (men and women) or biological characteristics (males and females), based on the definitions provided in our review guidance from PRO‐EDI [21]. This inconsistency limits the applicability of the evidence and prevents meaningful subgroup analyses based on biological or social differences, ultimately hindering conclusions about whether the recruitment strategies tested are effective for specific population groups.

Future recruitment SWATs should say more about who was included in the evaluation and, as a minimum, report the six PRO‐EDI mandatory characteristics of age, sex, gender, ethnicity, socioeconomic status, and location [21]. Trial teams may also wish to utilise the more comprehensive PROGRESS‐Plus framework to guide a deeper consideration of equity issues in their SWATs [126]. Where collection of these data is not possible or permitted (for example, ethnicity data in some jurisdictions), this should be clearly reported. Future research must prioritise evaluating recruitment strategies in the Global South and low‐ and middle‐income countries (LMICs). Addressing this stark geographic imbalance is essential to determine the transferability of these findings across diverse healthcare systems, cultural, and socio‐economic contexts, ensuring the recruitment evidence base is globally representative and applicable. Researchers should aim to understand how contextual factors – such as healthcare infrastructure, digital access, literacy levels, and cultural norms – affect the feasibility and effectiveness of recruitment strategies. Funding bodies and trial networks should actively support and incentivise recruitment methodology research in under‐represented settings to reduce the current Global North bias and to support greater involvement in trials of currently under‐served groups. Collaborative, international SWATs embedded within multinational trials should be encouraged, as they would enable comparative analyses of recruitment strategies across diverse settings and enhance the external validity of findings.

### Methodological evolution and impact

This update introduced several changes to enhance rigour, directly impacting on our findings. Focusing exclusively on randomised trials ensured our meta‐analyses reflected high‐certainty evidence, but this refined scope also highlighted that many previously identified strategies lack the data required to reach robust conclusions. Similarly, including retention, cost‐effectiveness, and EDI provided deeper insights into the long‐term sustainability of recruitment strategies. However, this mostly revealed a significant reporting deficit for these outcomes. Ultimately, these shifts replaced over‐optimistic assessments with a more cautious stance, highlighting a thin evidence base and an urgent need for standardised reporting of retention, costs, and EDI data.

### Limitations of the evidence included in the review

While over half of all included studies were new to this review update, we still have surprisingly little robust evidence supporting strategies that actually improve recruitment to trials. The overall picture of effective strategies to improve trial recruitment remains largely unchanged from the 2018 review [11], and is similar to that reported in 2010 [9, 127]. In other words, almost two decades of research into the effect of strategies to improve trial recruitment has not substantially reduced our uncertainty with regard to which strategies make recruitment more likely. Five recruitment strategies with high‐certainty evidence – three of which do not improve recruitment and two of which present substantial challenges – is thin gruel after 20 years of recruitment research. We should all be concerned.

The chief reasons behind this state of affairs include methodology researchers' inclination to evaluate new strategies rather than replicate evaluations of existing strategies. This means the evidence base is heavily fragmented; many strategies, including several with moderate‐certainty evidence, rely on single studies. Consequently, quantitative estimates such as risk differences (RDs) must be interpreted with caution, as they may reflect specific trial contexts rather than a universal effect. This reliance on single studies introduces significant uncertainty regarding the transferability of findings across different clinical areas, trial contexts, and demographics. A strategy’s apparent effectiveness is not guaranteed in new settings, which underscores the urgent need to move beyond single evaluations in single contexts toward co‐ordinated replication to achieve the stability and precision required for high‐certainty evidence.

Although we used random‐effects models to provide conservative effect estimates, substantial heterogeneity remains a challenge. We planned subgroup analyses including study setting, disease area, and trial design. However, these were not feasible for most comparisons due to the scarcity of trials per strategy. One exception was monetary incentives, where data allowed us to investigate allocation concealment. In this instance, the effect remained consistent (RD 4%, 95% CI 2% to 7%) across studies with adequate concealment. For most other strategies, the lack of granular data is a limitation, as effectiveness likely depends on the host trial context. Consequently, our pooled estimates represent average effects that may vary in practice. This inability to fully account for heterogeneity reinforces the need for SWAT replications to identify the conditions under which these strategies succeed.

Poor reporting also leads to uncertain risk of bias assessments, and we recommend that researchers start to use the reporting standard for SWATs [128]. We strongly urge the trial methodology research community to strengthen the evidence base by prioritising replication of existing strategies over the development and testing of new ones. We urgently need less recruitment strategy innovation and more replication to reach conclusive answers. Future research should replicate promising strategies with moderate‐certainty evidence to strengthen the evidence base, including: SMS messages mentioning trial place scarcity or including participant quotes, telephone reminders, enclosing questionnaires with invitations, multimedia information via digital links or QR codes, and remote site initiation visits. While currently supported by low‐certainty evidence, monetary incentives are also a priority because they are frequently used by trial teams and may improve recruitment.

The literature search was conducted in February 2023, and some relevant studies may have been published since then. However, recruitment research continues to be characterised by isolated evaluations rather than systematic replication. In the absence of major ongoing replication studies, it is unlikely that individual studies published since 2023 would materially change the overall conclusions of this review.

SWAT initiatives such as MRC START [122, 129], TRECA [130], PROMETHEUS [125], and Implement SWATs have played, or are playing, a central role in the co‐ordinated evaluation of recruitment strategies across multiple trials. Notably, two recruitment strategies rated as having high‐certainty evidence and supported by the largest number of studies – multimedia information provided via link or QR code (Analysis 46.1; Table) and user‐tested participant information leaflets (Analysis 24.1; Table) – were developed through MRC START and TRECA, respectively. PROMETHEUS and Implement SWATs are expected to contribute additional studies in future updates of this review. Reducing uncertainty around recruitment strategies in a timely manner requires sustained focus and co‐ordination across diverse trial contexts.

All recruitment strategies have a cost, and generally authors do not tell us what this is. We strongly encourage researchers to report cost data to support formal cost‐effectiveness analyses (see guidance from Gkekas and colleagues [131]), equity, diversity, and inclusion data to inform applicability, as well as retention data to assess potential impacts on longer‐term trial engagement. Collectively, these elements are essential for enabling researchers to make evidence‐based decisions about which recruitment strategies are most appropriate for their contexts.

### Potential biases in the review process

Despite conducting a comprehensive search and screening nearly 21,000 records in this update, we identified three relevant studies outside the formal search process. Two were published after we completed the search, so our search strategy missed only one eligible study. We welcome feedback on any studies we may have overlooked, and on newly published research, to help ensure future updates remain complete and up to date.

## Authors' conclusions

### Implications for systematic reviews and evaluations of healthcare

Researchers should prioritise well‐designed evaluations of strategies to improve recruitment in randomised trials. We strongly urge the methodology research community to strengthen the evidence base by prioritising replication of existing strategies over the development and testing of new ones. What is needed urgently now is less innovation and more replication.

Conclusions about cost‐effectiveness, participant retention, as well as equity, diversity, and inclusion (EDI) were severely limited by sparse, low‐quality data. Most strategies lacked the reporting necessary to assess their cost‐effectiveness or impact on retention. Similarly, the evidence for recruiting under‐served groups is currently too weak to support clear recommendations. To avoid overinterpretation and build a robust evidence base, future Studies Within A Trial (SWATs) must move beyond simple recruitment rates to consistently report cost, retention, and participant characteristics data that support EDI judgements.

### Implications for methodological research

The implications for methodological research are clear.

**Replication, replication, replication.** We need many more replications of SWATs and less innovation. After 20 years of research, only five of 65 comparisons are supported by GRADE high‐certainty evidence. The research community should therefore focus on testing the strategies already identified in this review to strengthen the evidence base, rather than developing new strategies. There are resources available to support trial teams to undertake replications of SWATs, which we outline below.

**The research community should focus on testing priority recruitment strategies.** To accelerate the growth of the evidence base, the research community should replicate strategies identified as priorities for evaluation. The UK's National Institute for Health and Care Research (NIHR)‐funded Implement SWATs project, working together with the SWAT Network, has produced a list of 11 priority recruitment and retention SWATs for trial teams to undertake, which can be found here: https://www.trialforge.org/2024/02/a-list-of-11-priority-recruitment-and-retention-swats/[132]. These SWATs were prioritised based on the frequency with which recruitment and retention strategies were used (assessed using a technique developed by Way and colleagues [133]), evidence from Cochrane systematic reviews [11, 18], and findings from two James Lind Alliance priority‐setting exercises on recruiting and retaining trial participants [134, 135]. Evaluations of these SWATs would be highly valuable.

**There are promising strategies that need further replications.** Future research should prioritise replicating promising strategies with moderate GRADE certainty that probably increase recruitment, such as SMS messages mentioning trial place scarcity or including participant quotes, telephone reminders versus SMS reminders, and enclosing questionnaires with invitations. Multimedia information via digital links or QR codes and remote site initiation visits also warrant testing as potentially cost‐effective methods. Although evidence is currently of low certainty, monetary incentives are a key priority because they are widely used and may improve recruitment. These strategies are the focus of the NIHR‐funded Implement SWATs project, which is currently undertaking coordinated replication SWATs to build a more robust evidence base.

**The research community should make use of available template SWAT protocols and resources.** To support trial teams to undertake replications of SWATs, template protocols, ethics guidance, and other resources for testing priority SWATs have been developed as part of the PRESS project, which are available here: https://www.trialforge.org/trial-diversity/press/. The PRESS project developed protocols and resources to assist and encourage trial teams to evaluate recruitment and retention strategies in their trials, especially those that have been prioritised for evaluation.

**We need better reporting of the cost‐effectiveness of recruitment strategies.** We strongly encourage researchers to report cost data to support cost‐effectiveness analyses. Our findings highlight a significant gap in the evidence on the cost‐effectiveness of recruitment strategies. Of the 91 studies included in this review, we were only able to estimate cost‐effectiveness in nine comparisons. Health economic guidance for undertaking and reporting recruitment and retention SWATs has been developed as part of the PRESS project, and can be found here: https://www.trialforge.org/wp-content/uploads/2025/03/PRESS-Health-Economics-Guidance-for-randomised-recruitment-and-retention-SWATs-V1.0-24.03.2025.pdf.

**Evidence from the Global South and low‐ and middle‐income countries (LMICs) is needed.** Current evidence is heavily skewed towards populations in high‐income, Global North countries (and the UK and USA in particular), with only one study including participants from middle‐income‐country settings. Future research must prioritise evaluating recruitment strategies in LMICs to ensure recruitment science is globally representative and applicable.

**Applicability and generalisability of findings.** Current evidence is concentrated in healthcare settings, leaving strategy effectiveness in environments like social care or education uncertain. Transferability is also limited by the interplay between study context and participant characteristics. Recruitment effectiveness is not universal; it is influenced by a range of factors including disease severity, socioeconomic status, and perceived trial burden. Since most strategies have only been tested in specific clinical contexts, trial teams should exercise caution when applying these methods to different populations or settings.

**Better reporting of participant populations and contexts is needed.** In many cases, limited or no demographic information (e.g. age, gender, ethnicity) is reported for SWAT participants, which restricts the ability to assess how applicable the findings are to specific population groups. Future recruitment SWATs should provide clearer information about who was included in the evaluation, and at a minimum, report the six mandatory PRO‐EDI characteristics, which are: age; sex; gender; race, ethnicity, and ancestry; socioeconomic status; and location (country/countries of data collection and site co‐ordination). More information is available at https://www.trialforge.org/trial-diversity/pro-edi/.

**We also need better reporting of the impact of recruitment strategies on participant retention.** Retention outcomes were reported in only 13 comparisons. The evidence base is limited and highly uncertain, with most strategies supported by single studies and imprecise estimates. As it is entirely possible for recruitment interventions to also affect retention, future SWATs should report retention at the host trial’s primary outcome time point to align with core outcome standards and enhance comparability across studies.

**Register your recruitment SWAT.** Researchers evaluating recruitment strategies should register their protocol on the SWAT Repository (go.qub.ac.uk/SWAT‐SWAR), in addition to registering the SWAT as part of the main trial on trial registries (such as ClinicalTrials.gov, EU‐CTR, and ISRCTN).

**Consider informing the Trial Forge SWATs Centre.** Researchers should consider notifying the York Trial Forge SWATs Centre about their planned recruitment (and other trial process) evaluations to favour better co‐ordination and wider dissemination of evaluation efforts.

**Consider including a SWAT in all your trial funding applications.** Researchers should aim to include SWAT evaluations – especially those identified as high‐priority – in all their trial funding applications. Guidance for researchers applying for funding to conduct high‐priority SWATs of recruitment and retention strategies has been developed as part of the PRESS project, and is available here: https://osf.io/w6zym/files/osfstorage. Funders should support this to avoid another decade with little progress regarding which interventions are effective in improving trial recruitment. Indeed, some funders are increasingly supporting SWATs (e.g. the NIHR and the Wellcome Trust in the UK; the Health Research Board Trial Methodology Network in Ireland), and we strongly encourage more funders to routinely support the embedding of SWATs in the trials they fund.

## Supporting Information

Supplementary materials are available with the online version of this article: 10.1002/14651858.MR000013.pub7.

Supplementary materials are published alongside the article and contain additional data and information that support or enhance the article. Supplementary materials may not be subject to the same editorial scrutiny as the content of the article and Cochrane has not copyedited, typeset or proofread these materials. The material in these sections has been supplied by the author(s) for publication under a Licence for Publication and the author(s) are solely responsible for the material. Cochrane accordingly gives no representations or warranties of any kind in relation to, and accepts no liability for any reliance on or use of, such material.

**Supplementary material 1** Search strategies

**Supplementary material 2** Characteristics of included studies

**Supplementary material 3** Characteristics of excluded studies

**Supplementary material 4** Analyses

**Supplementary material 5** Data package

**Supplementary material 6** Protocol

**Supplementary material 7** Reference list of reviews and other publications searched

**Supplementary material 8** Full list of strategies

**Supplementary material 9** Participant numbers per study

**Supplementary material 10** Data and analyses

**Supplementary material 11** Equity, diversity and inclusion characteristics of included participants (PRO‐EDI)

**Supplementary material 12** Cost‐effectiveness of recruitment strategies

**Supplementary material 13** Impact of recruitment strategies on retention rates

## Figures and Tables

**Table:** 

**Date**	**Event**	**Description**
15 September 2026	New search has been performed	Search updated to 15 February 2023. We made significant changes to the protocol to strengthen methodological rigour, such as excluding hypothetical and quasi‐randomised trials, and adding three new secondary outcomes: 1) retention rates, 2) cost‐effectiveness, and 3) EDI characteristics of included participants. The review has changed substantially. We added 53 new studies to the review, and identified two additional strategies supported by high‐certainty evidence according to GRADE criteria. For the new outcomes, we identified costs were reported in 17 of the 91 included studies; the impact of the recruitment strategies on participant retention was reported in 15 studies; and that 99% of included studies were from high‐income countries. We also identified the reported participant demographics to be age (49 studies), sex (32 studies), gender (27 studies), and education level (16 studies). We have updated the 'Authors' conclusions' section to make recommendations for methodological researchers to prioritise replications, testing of strategies that have been identified as high‐priority, along with signposting to significant new resources that the trial methodology community can use to robustly evaluate recruitment strategies.
15 September 2026	New citation required and conclusions have changed	Review updated

**Table:** 

**Date**	**Event**	**Description**
20 February 2018	New citation required and conclusions have changed	Review updated
9 June 2017	New search has been performed	Review updated: search extended to February 2015; 24 additional included studies, including 6 recent studies identified outside the search (two from 2017) and 1 study missed in earlier searches. One previously included study excluded (it was included in error). Changes to protocol for next update introduced, chiefly linked to hypothetical trials, which will be excluded in future updates. While we added new studies to the review, the overall picture with regard to interventions for improving recruitment to trials remains similar to the previous version of the review. We have updated the 'Implications for methodological research' section to suggest interventions that methodological researchers should prioritise for enhanced evaluation, along with protocols for Studies Within A Trial (SWATs) to support these areas.
10 June 2011	New search has been performed	Review updated: search extended to April 2010, 18 additional included studies. While new studies were added to the review, the overall picture with regard to interventions to improve recruitment to trials remains similar to the previous version of the review.
16 April 2010	Feedback has been incorporated	Feedback from Margo Michaels added with reply from authors.
10 November 2009	New citation required but conclusions have not changed	The title of this review has changed, as have the authors.
10 November 2009	New search has been performed	New search conducted September 2007. Twelve new studies identified.
27 December 2007	Amended	Converted to new review format.
20 February 2007	New citation required and conclusions have changed	Substantive amendment.

## Additional information

### Registration and protocol

Protocol: Strategies to improve recruitment to research studies (2002). https://doi.org/10.1002/14651858.MR000013

Original review: Strategies to improve recruitment to research studies (2002). https://doi.org/10.1002/14651858.MR000013.pub2

Review updates:

Strategies to improve recruitment to research studies (2007). https://doi.org/10.1002/14651858.MR000013.pub3

Strategies to improve recruitment to randomised controlled trials (2010). https://doi.org/10.1002/14651858.MR000013.pub5

Strategies to improve recruitment to randomised trials (2018). https://doi.org/10.1002/14651858.MR000013.pub6

### Data, code and other materials

As part of the published Cochrane review, the following is made available for download for users of the Cochrane Library (see Data package, Supplementary material 5): full search strategies for each database; full citations of each unique report for all studies (included, ongoing, or awaiting classification, or excluded at the full‐text screen) in the final review; study data, including study information, study arms, and study results or test data; consensus risk of bias assessments; and analysis data, including overall estimates and settings, subgroup estimates, and individual data rows. Appropriate permissions have been obtained for such use. Analyses and data management were conducted within Cochrane’s authoring tool, RevMan, using the inbuilt computation methods. Template data extraction forms are available from the authors on reasonable request.

### History

Protocol first published: Issue 3, 2002 Review first published: Issue 1, 2004
