# Supplementary material for: Strategies to improve recruitment to randomised trials
Source: Cochrane Database Syst Rev. 2026 Sep 15;2026(9):MR000013. doi: 10.1002/14651858.MR000013.pub7 (PMC13576036; doi:10.1002/14651858.MR000013.pub7)
Supplement: Supplementary file 1 [file MR000013-SUP-01-searchStrategy.html]

Search strategies


# Supplementary material 1 to: Strategies to improve recruitment to randomised trials

Parker A, Mongelli G, Piccolo-Lawrance C, Coleman E, Gkekas A, Wang HI, Dawson S, Green H, Way R, Rai A, Bruhn H, Sullivan F, Boxall C, Chan M, Manson P, Hewitt C, Fairhurst C, Clarke M, Treweek S
  
https://doi.org/10.1002/14651858.MR000013.pub7

The material in this section has been supplied by the author(s) for publication under a Licence for Publication and the author(s) are solely responsible for the material. Cochrane has peer reviewed this material in accordance with its editorial policies, but Cochrane has not copyedited, formatted or proofread. Cochrane accordingly gives no representations or warranties of any kind in relation to, and accepts no liability for any reliance on or use of, such material.

Back to top

# Search strategies

Search strategies undertaken on 15th and 16th February 2023.

### Ovid MEDLINE(R) and Epub Ahead of Print, In-Process, In-Data-Review & Other Non-Indexed Citations, Daily and Versions

<1946 to February 15, 2023>

1 Patient Selection/ 69271

2 ((participat$ or recruit$ or enrol$) adj4 trial?).tw. 35516

3 1 or 2 102118

4 Informed Consent/ 38582

5 informed consent.tw. 43089

6 4 or 5 70143

7 exp Clinical Trials as Topic/ 380558

8 Research Subjects/ 6673

9 (trial? or study or studies or research).tw. 13061511

10 7 or 8 or 9 13167732

11 3 or (6 and 10) 139927

12 (research support nih extramural or research support nih intramural or research support non us govt or research support us govt non phs or research support us govt phs).pt. 10133718

13 recruitment.ab. /freq=2 32756

14 participation.ab. /freq=2 38366

15 12 or 13 or 14 10167972

16 11 and 15 45241

17 randomized controlled trial.pt. 587215

18 controlled clinical trial.pt. 95195

19 random$.ab. 1351586

20 ((nest\* or embed\*) adj4 (RCT or trial? or stud\*)).tw. 20524

21 (substud\* or sub-stud\*).tw. 8692

22 ("stud\* within a trial" or "trial? within a trial").tw. 91

23 ("stud\* in a trial" or "trial? in a trial").tw. 41

24 ("stud\* within a study" or "trial? within a study").tw. 3

25 ("stud\* in a study" or "trial? in a study").tw. 51

26 20 or 21 or 22 or 23 or 24 or 25 29183

27 16 and (17 or 18 or 19 or 26) 17540

28 exp animals/ not humans/ 5095551

29 27 not 28 17508

30 29 not (comment or editorial).pt. 17468

31 limit 30 to yr="2015 -Current" 7559

## Embase

<1974 to 2023 February 15>

1 ((participat$ or recruit$ or enrol$ or enter$ or entry) and (trial? or study)).ti. 25739

2 (select$ adj3 (participants or patients or controls)).tw. 243226

3 recruit$.ab. /freq=2 113842

4 participat$.ab. /freq=2 145388

5 research.tw. 2554243

6 2 and (3 or 4 or 5) 19891

7 Informed Consent/ 129517

8 (informed consent or consent process$ or consent procedure?).tw. 97282

9 exp "controlled clinical trial (topic)"/ 263565

10 (trial? or study or studies or research).tw. 17050394

11 (7 or 8) and (9 or 10) 101915

12 1 or 6 or 11 145719

13 Randomized Controlled Trial/ 771832

14 Cross-over Procedure/ 74276

15 random$.tw. 1933105

16 (factorial or crossover or cross-over or assign$ or allocat$).tw. 821652

17 ((nest\* or embed\*) adj4 (RCT or trial? or stud\*)).tw. 27399

18 (substud\* or sub-stud\*).tw. 16004

19 ("stud\* within a trial" or "trial? within a trial").tw. 151

20 ("stud\* in a trial" or "trial? in a trial").tw. 58

21 ("stud\* within a study" or "trial? within a study").tw. 10

22 ("stud\* in a study" or "trial? in a study").tw. 73

23 17 or 18 or 19 or 20 or 21 or 22 43344

24 13 or 14 or 15 or 16 or 23 2521979

25 nonhuman/ 7379552

26 editorial.pt. 765238

27 conference abstract.pt. 4685222

28 24 not (25 or 26 or 27) 1724672

29 12 and 28 21211

30 limit 29 to yr="2015 -Current" 11089

## APA PsycInfo

<2002 to February Week 2 2023>

1 exp Patient Selection/ 240

2 ((participat$ or recruit$ or enrol$) adj4 trial?).tw. 4463

3 1 or 2 4694

4 Informed Consent/ 3746

5 informed consent.tw. 7552

6 4 or 5 8505

7 exp Experimental Subjects/ 3168

8 (trial? or study or studies or research).tw. 2306087

9 7 or 8 2306196

10 3 or (6 and 9) 10749

11 exp Randomized Controlled Trials/ 1385

12 clinical trials/ 11756

13 random.ab. 49433

14 ((nest\* or embed\*) adj4 (RCT or trial? or stud\*)).tw. 4259

15 (substud\* or sub-stud\*).tw. 935

16 ("stud\* within a trial" or "trial? within a trial").tw. 6

17 ("stud\* in a trial" or "trial? in a trial").tw. 1

18 ("stud\* within a study" or "trial? within a study").tw. 2

19 ("stud\* in a study" or "trial? in a study").tw. 28

20 14 or 15 or 16 or 17 or 18 or 19 5212

21 11 or 12 or 13 or 20 67010

22 10 and 21 1578

23 limit 22 to yr="2015 -Current" 624

## CINAHL

<Search date, 16th February>

S1 (MH "Patient Selection") 25,265

S2 (participat$ OR recruit$ OR enrol$) N4 trial? 552

S3 S1 OR S2 25,760

S4 (MH "Consent (Research)") 4,847

S5 TX informed consent. 18,289

S6 TX s4 OR s5 22,027

S7 (MH "Research Subject Recruitment") 12,499

S8 TX trial? OR study OR studies OR research 4,745,419

S9 s7 OR s8 4,745,419

S10 S6 AND S9 19,261

S11 S3 OR S10 44,736

S12 (MH "Clinical Trials+") 347,336

S13 AB random 71,249

S14 TX (nest\* OR embed\*) N4 (RCT OR trial$ OR stud\*) 7,605

S15 TX "study within" 1,575

S16 TX "trial within" 220

S17 TX sub stud\* 4,292

S18 S12 OR S13 OR S14 OR S15 OR S16 OR S17 420,515

S19 S11 AND S18 8,419

S20 S11 AND S18 Limiters - Published Date: 20150101-20231231 3,463

## Web of Science Core Collection: SCI, SSCI <search date, 16th February>

1: recruitment NEAR/8 controlled NEAR/3 trial$ (Topic) 662

2: recruitment NEAR/8 randomi$ed NEAR/3 trial$ (Topic) 1022

3: recruitment NEAR/8 clinical NEAR/3 trial$ (Topic) 1942

4: recruitment NEAR/8 study NEAR/2 trial (Topic) 347

5: (nest\* or embed\*) NEAR/4 (RCT or trial$ or stud\*) (Topic) 33025

6: recruitment (Topic) 214536

7: #5 AND #6 732

8: #1 OR #2 OR #3 OR #4 OR #7 3641

9: #1 OR #2 OR #3 OR #4 OR #7 and Article (Document Types) and 2023 or 2022 or 2021 or 2020 or 2019 or 2018 or 2017 or 2016 or 2015 (Publication Years) 1558

## ASSIA (Applied Social Sciences Index & Abstracts) <Search date: 16th February>

S1 recruitment NEAR/8 controlled NEAR/3 trial? 332

S2 recruitment NEAR/8 randomi?ed NEAR/3 trial? 452

S3 recruitment NEAR/8 clinical NEAR/3 trial? 503

S4 (nest\* or embed\*) NEAR/4 (RCT or trial? or stud\*) 3749

S5 sub-stud\* or "sub stud\*" 487

S6 "study within" or "trial within" 1909

S7 s4 or s5 or s6 5989

S8 recruitment 43508

S9 s7 and s8 1480

S10 s1 or s2 or s3 or s9 2287

S11 (s1 or s2 or s3 or s9) AND pd(20150101-20231231) 1268

## ERIC (EBSCOhost)

<Searched ERIC from 1st Jan 2015 to 31 Dec 2023, search date - 16th February >

S1 recruit\* 34,102

S2 participat\* 191,827

S3 S1 OR S2 220,432

S4 clinical trial\* OR controlled trial\* OR randomi\* 9,372

S5 (nest\* OR embed\*) N4 (RCT OR trial$ OR stud\*) 1,167

S6 "study within" OR "trial within" 206

S7 sub stud\* 676

S8 S4 OR S5 OR S6 OR S7 11,341

S9 S3 AND S8 2,476

S10 S3 AND S8 Limiters - Date Published: 20150101-20231231 418

## Online Resource for Research in Clinical triAls (ORRCA)

Date of Search: 07/02/2023 18:41:20

Search Options:

Year from: 1 Jan 2015

Year to: 31 Dec 2019

Recruitment research

- Recruitment evidence type
- Randomised evaluation

=115 papers identified
