# Supplementary material for: Strategies to improve recruitment to randomised trials
Source: Cochrane Database Syst Rev. 2026 Sep 15;2026(9):MR000013. doi: 10.1002/14651858.MR000013.pub7 (PMC13576036; doi:10.1002/14651858.MR000013.pub7)
Supplement: Supplementary file 2 [file MR000013-SUP-02-characteristicsOfIncludedStudies.html]

Characteristics of included studies


# Supplementary material 2 to: Strategies to improve recruitment to randomised trials

Parker A, Mongelli G, Piccolo-Lawrance C, Coleman E, Gkekas A, Wang HI, Dawson S, Green H, Way R, Rai A, Bruhn H, Sullivan F, Boxall C, Chan M, Manson P, Hewitt C, Fairhurst C, Clarke M, Treweek S
  
https://doi.org/10.1002/14651858.MR000013.pub7

The material in this section has been supplied by the author(s) for publication under a Licence for Publication and the author(s) are solely responsible for the material. Cochrane has reviewed this material, but Cochrane has not copyedited, formatted or proofread. Cochrane accordingly gives no representations or warranties of any kind in relation to, and accepts no liability for any reliance on or use of, such material.

Back to top

# Characteristics of included studies

## Table of contents

- Studies ordered by Study ID
  - Abd-Elsayed 2012
  - Agni 2022
  - Arundel 2017
  - Avenell 2004
  - Bergenmar 2014
  - Bobb 2016
  - Bracken 2019
  - Brierley 2012
  - Brown 2015
  - Chen 2011
  - Chow 2020
  - Cockayne 2017 (START PIL REFORM)
  - Cooper 1997
  - Coyne 2003
  - Crane 2016
  - Dear 2011
  - Du 2008
  - Du 2009
  - Dwyer 2022
  - Ethier 2017
  - Fairhurst 2022
  - Felicitas-Perkins 2017
  - Ford 2004
  - Foss 2016
  - Fowell 2006
  - Fracasso 2013
  - Free 2010
  - Free 2011
  - Garvelink 2015
  - Giguere 2015
  - Halpern 2021a ($200)
  - Halpern 2021a ($500)
  - Halpern 2021b ($100)
  - Halpern 2021b ($300)
  - Hemminki 2004
  - Hughes-Morley 2016
  - Hutchison 2007
  - Isaksson 2025
  - Ives 2001
  - Jefferson 2018
  - Jennings 2015a
  - Jennings 2015b
  - Jennings 2015c
  - Jennings 2015d
  - Jennings 2015e
  - Jolly 2019 (START MMI PSM-COPD)
  - Kamen 2018
  - Kendrick 2001
  - Kimmick 2005
  - Knapp 2020 (START PIS CASPER)
  - Knapp 2023a (BALANCE)
  - Knapp 2023b (BAMP)
  - Knapp 2023c (CHAMP-UK)
  - Knapp 2023d (FORCE)
  - Knapp 2023e (Thermic-3)
  - Knapp 2023f (UKALL)
  - Larkey 2002
  - Lee 2017
  - Liénard 2006
  - Lipstein 2021
  - Litchfield 2005
  - Madurasinghe 2021 (START PIS HeLP Diabetes)
  - Madurasinghe 2023a (START MMI GHT2000a)
  - Madurasinghe 2023b (START MMI GHT2000b)
  - Madurasinghe 2023c (START MMI HI-Light)
  - Madurasinghe 2023d (START MMI seAFOod)
  - Man 2015 (START PIS Healthlines Depression)
  - Mattock 2020
  - Maxwell 2017
  - McCaffery 2019
  - Miller 2021a
  - Miller 2021b
  - Monaghan 2007
  - Nystuen 2004
  - Paris 2015
  - Parker 2018 (START PIS ECLS)
  - Paul 2011
  - Paul 2014
  - Rogers 2019
  - Saif 2020
  - Sheridan 2020 (START PIS ISDR)
  - Simes 1986
  - Skinner 2019
  - Sundaresan 2017
  - Tilley 2012
  - Tilley 2021
  - Trevena 2006
  - Treweek 2012
  - Veerus 2016
  - Wadland 1990
  - White 2023
  - Whiteside 2019
  - Witham 2018
  - Wong 2013
- Footnotes
- References to studies

## Studies ordered by Study ID

Abd-Elsayed 2012

|  |  |  |
| --- | --- | --- |
| ***Study characteristics*** | | |
| Methods | Randomised controlled trial | |
| Data | Setting: secondary care in USA. 499 participants were eligible for 1 of 3 trials; all had substantial illness requiring major surgery (cardiac) at least 24 hours after being asked about consent | |
| Comparisons | Investigated the use of different consent form presentations  Intervention A: consent documents on heavy weight cream-coloured paper (20-pound) and a blue folder  Comparator: consent documents as photocopies stapled together. | |
| Outcomes | Proportion recruited to trial | |
| ***Risk of bias*** | | |
| **Bias** | **Authors' judgement** | **Support for judgement** |
| Random sequence generation ok? | Low risk | The authors used a computer-generated random number sequence to allocate participants. |
| Allocation concealment? | Low risk | Allocation was concealed via a centralised, automated telephone system. This ensured personnel could not foresee or influence assignments, maintaining randomisation integrity |
| Blinding of participants and personnel ok? | Unclear risk | Participants did not know there was a study. Personnel knew, and there was possibility that this could influence consent conversation, but there was substantial training so the effect is less clear. |
| Blinding of outcome assessment ok? | Low risk | Participants were blind and data entered by someone who was blinded |
| Incomplete outcome data handled ok? | Low risk | All randomised participants were accounted for in the final analysis. The use of objective administrative records to measure recruitment ensured no missing outcome data or loss to follow-up |
| Free of selective reporting? | Low risk | Review only interested in recruitment, which is reported |
| Was the study free of other bias? | High risk | Trial stopped early because of host trials stopping early and consent responsibility for the third trial site moving to a different department |
| Overall bias? | High risk | Selection and attrition bias risks are low due to robust randomisation and objective data. Risks for blinding and other bias are unclear because staff were not masked and the trial terminated early. |

Agni 2022

|  |  |  |
| --- | --- | --- |
| ***Study characteristics*** | | |
| Methods | 2x2 factorial cluster-randomised study within a trial | |
| Data | Setting: Hospitals, UK. 1215 participants (recruited from 20 clusters) were patients aged 60 years or older with intracapsular hip fracture requiring acute surgical treatment. | |
| Comparisons | Invesitgating additional support and/or digital nudges for Trainee Principal Investigators (TPI).  Intervention 1: Enhanced Trainee Principal Investigator intervention involving education, support, supplementary information, 1:1 telephone training, and monthly communication  Intervention 2: a personalised email nudge expressing appreciation and encouragement each time a participant is randomised  Intervention 3: Both interventions combined  Comparator: Standard practice for TPIs involving a TPI manual, automated email, monthly updates, acknowledgment as collaborator. | |
| Outcomes | Total number of patients randomised, from each site, in their first 6 months of trial recruitment | |
| ***Risk of bias*** | | |
| **Bias** | **Authors' judgement** | **Support for judgement** |
| Random sequence generation ok? | Low risk | Randomisation was done via minimisation, on the day the site recruited their first participant; no selection bias. |
| Allocation concealment? | Low risk | As above, minimisation would prevent the possibility of selection bias. |
| Blinding of participants and personnel ok? | Low risk | ‘Participating sites, the data analyst and trial team were not blind to allocation’ - blinding not feasible due to the nature of the intervention, however is not deemed to have caused bias. |
| Blinding of outcome assessment ok? | Low risk | Outcomes were data collected for the trial (i.e. number randomised), therefore no blinding was required as not possible to influence these outcomes due to bias. |
| Incomplete outcome data handled ok? | Low risk | Screening data was unable to be obtained – so the PO is only comparing raw numbers, but does not introduce bias |
| Free of selective reporting? | Low risk | All results were reported in the manuscriptive; no evidence of bias. |
| Was the study free of other bias? | Low risk | No evidence of any other sources of bias. Site of the CI for the SWAT excluded to avoid bias. |
| Overall bias? | Low risk | No concerns about potential sources of bias raised. |

Arundel 2017

|  |  |  |
| --- | --- | --- |
| ***Study characteristics*** | | |
| Methods | 2-arm individually-randomised study within a trial | |
| Data | Setting: Hospital, United Kingdom and Ireland. 4314 participants were patients aged 65 years and over who attended routine podiatry services within the past 6 months, have had one fall in the past 12 months; or one fall in the past 24 months requiring hospital attention | |
| Comparisons | Investigating a recruitment primer leaflet  Intervention: A two-page pre-notification leaflet, sent 2–3 weeks prior to the trial recruitment pack, which used easy-to-read language, local research context, FAQs, and gain framing to encourage patient participation in the RCT.  Comparator: Trial recruitment pack only | |
| Outcomes | Proportion recruited to host trial, cost-effectiveness | |
| ***Risk of bias*** | | |
| **Bias** | **Authors' judgement** | **Support for judgement** |
| Random sequence generation ok? | Low risk | Simple parallel-arm randomisation, using a 1:2 ratio. |
| Allocation concealment? | Low risk | The participants did not know the alternative allocation. “The allocation was independent and concealed”, administered by a data manager at York Trials Unit. |
| Blinding of participants and personnel ok? | Low risk | The participants were randomised properly in this study. The podiatry clinical staff was not blinded, but the authors provided clear explanation about blinding of participants and trial staff. |
| Blinding of outcome assessment ok? | Low risk | The participants were unaware of receiving a specific recruitment strategy, for the purpose of improving patient recruitment in randomised trials. |
| Incomplete outcome data handled ok? | Low risk | There is no evidence of missing data in the study. |
| Free of selective reporting? | Low risk | There is no evidence of reporting bias in the study. |
| Was the study free of other bias? | Low risk | No other source of bias has been found in the study. |
| Overall bias? | Low risk | Low risk of bias as described in individual items above. |

Avenell 2004

|  |  |  |
| --- | --- | --- |
| ***Study characteristics*** | | |
| Methods | Randomised controlled trial | |
| Data | Setting: secondary care, UK. 538 participants aged 70 years or over, attending a fracture clinic or orthopaedic ward | |
| Comparisons | Investigated the effect of different trial designs  Open trial design comparing vitamin D versus calcium versus vitamin D plus calcium versus no tablets. Compared to conventional trial comparing vitamin D versus calcium versus vitamin D plus calcium versus placebo. | |
| Outcomes | Proportion recruited to trial | |
| ***Risk of bias*** | | |
| **Bias** | **Authors' judgement** | **Support for judgement** |
| Random sequence generation ok? | Low risk | Pre-programmed laptop computer-generated sequence |
| Allocation concealment? | Low risk | Adequate |
| Blinding of participants and personnel ok? | Low risk | Not all participants were blinded, but this was the point of the evaluation so the trial has not been penalised on this risk of bias item. Those in comparison group were blinded. Tablets were sent out centrally by trial staff, not handed out by clinical staff. |
| Blinding of outcome assessment ok? | Low risk | Objective outcome recorded by trial team |
| Incomplete outcome data handled ok? | Low risk | Adequate |
| Free of selective reporting? | Low risk | Recruitment outcome presented, which is all the review needs |
| Was the study free of other bias? | Low risk | No other biases apparent |
| Overall bias? | Low risk | Low risk of bias |

Bergenmar 2014

|  |  |  |
| --- | --- | --- |
| ***Study characteristics*** | | |
| Methods | Randomised controlled trial | |
| Data | Setting: secondary care, Sweden. Participants were 130 patients eligible for a phase II or III cancer drug trial involving 1 of 13 oncologists consenting to be recorded during study period | |
| Comparisons | Investigated use of audio recording to improve communication about the trial  Intervention: an audio recording (CD), using a portable voice recorder, of the information given at the medical consultation in which the patients were informed about a clinical drug trial  Comparator: no CD | |
| Outcomes | Proportion recruited to trial | |
| ***Risk of bias*** | | |
| **Bias** | **Authors' judgement** | **Support for judgement** |
| Random sequence generation ok? | Unclear risk | Nurse did randomisation but does not say how |
| Allocation concealment? | Unclear risk | As above |
| Blinding of participants and personnel ok? | Low risk | Adequate |
| Blinding of outcome assessment ok? | Low risk | Adequate |
| Incomplete outcome data handled ok? | Low risk | Adequate |
| Free of selective reporting? | Low risk | Recruitment reported and this is only outcome needed for review |
| Was the study free of other bias? | Low risk | No other biases apparent |
| Overall bias? | Unclear risk | Unclear risk of bias |

Bobb 2016

|  |  |  |
| --- | --- | --- |
| ***Study characteristics*** | | |
| Methods | 2-arm individually-randomised study within a trial | |
| Data | Setting: Hospital, USA. 131 participants were adult emergency department patients expected to be admitted to the hospital. | |
| Comparisons | Telemedicine-enabled research informed consent versus standard face-to-face consent  Intervention: telephone call reminder, made within 4 days of randomization by two staff members, confirmed participants' interest in the T4DM study, provided information on upcoming lab screenings, offered to resend screening forms if needed, and addressed any questions participants had about joining the study.  Comparator: SMS reminders, sent within 2 days of randomization via an online bulk SMS service, providing key enrollment information and included a social proof cue to motivate participants to take action. | |
| Outcomes | Proportion recruited to host trial | |
| ***Risk of bias*** | | |
| **Bias** | **Authors' judgement** | **Support for judgement** |
| Random sequence generation ok? | Low risk | “After patients were initially evaluated by a senior resident or physician assistant, they were randomized to telemedicineenabled or F2F research consent using a 1:1 allocation ratio. Randomization was conducted using block randomization with block sizes of 4." |
| Allocation concealment? | Low risk | “Randomization was conducted using block randomization with block sizes of 4, and the code was concealed in sequentially numbered opaque sealed envelopes.” |
| Blinding of participants and personnel ok? | Low risk | Participants were blinded to their SWAT allocation until after host trial completion. Blinding of personnel recruiting participants was not possible. |
| Blinding of outcome assessment ok? | Low risk | Enrollment rate is not subjective, and participants were unaware of the SWAT until after this had been collected. |
| Incomplete outcome data handled ok? | Low risk | Incomplete outcome data not relevant for the proportion of participants enrolled in each group, although they did not report the numbers of eligible participants approached in each group. |
| Free of selective reporting? | Low risk | No protocol available, however give parent trial accrual rates as specified in the methods section |
| Was the study free of other bias? | Low risk | No other sources of bias identified. |
| Overall bias? | Low risk | No concerns about bias were identified. |

Bracken 2019

|  |  |  |
| --- | --- | --- |
| ***Study characteristics*** | | |
| Methods | 2-arm individually-randomised study within a trial | |
| Data | Setting: Hospital, Australia. 709 participants were men aged 50-74 years, obese or overweight, with prediabetes or newly diagnosed type 2 diabetes, and a low serum testosterone. | |
| Comparisons | Telephone call reminder versus SMS reminder  Intervention: telephone call reminder, made within 4 days of randomization by two staff members, confirmed participants' interest in the T4DM study, provided information on upcoming lab screenings, offered to resend screening forms if needed, and addressed any questions participants had about joining the study   Comparator: SMS reminders, sent within 2 days of randomization via an online bulk SMS service, providing key enrollment information and included a social proof cue to motivate participants to take action. | |
| Outcomes | Proportion recruited to host trial, cost-effectiveness. | |
| ***Risk of bias*** | | |
| **Bias** | **Authors' judgement** | **Support for judgement** |
| Random sequence generation ok? | Low risk | Simple parallel-arm randomisation, where participants were stratified by centre and age group, using a 1:1 ratio. |
| Allocation concealment? | Low risk | The participants did not know the alternative allocation. |
| Blinding of participants and personnel ok? | Low risk | The participants were randomised properly in this study. |
| Blinding of outcome assessment ok? | Low risk | The participants were unaware of receiving a specific recruitment strategy, for the purpose of improving patient recruitment in randomised trials. |
| Incomplete outcome data handled ok? | Low risk | The approach stated by the authors is appropriate. “All participants were followed for response and included in the analysis, regardless of whether the allocated intervention was delivered or not.” |
| Free of selective reporting? | Low risk | There is no evidence of reporting bias in the study. |
| Was the study free of other bias? | Low risk | No other source of bias has been found in the study. |
| Overall bias? | Low risk | Low risk of bias as described in individual items above. |

Brierley 2012

|  |  |  |
| --- | --- | --- |
| ***Study characteristics*** | | |
| Methods | Randomised controlled trial | |
| Data | Setting: primary care, UK. 2330 participants were people eligible for a trial about computerised CBT in depression | |
| Comparisons | Investigated effect of length of the participant information leaflet on recruitment.  Intervention: short participant information leaflet (not clear how short) as initial info about trial  Comparator: full length participant information leaflet (8-pages) as initial info about trial | |
| Outcomes | Proportion recruited to trial | |
| ***Risk of bias*** | | |
| **Bias** | **Authors' judgement** | **Support for judgement** |
| Random sequence generation ok? | Low risk | Adequate |
| Allocation concealment? | Low risk | Adequate |
| Blinding of participants and personnel ok? | Low risk | People sending out packs blind, as well as potential participants |
| Blinding of outcome assessment ok? | Low risk | Adequate |
| Incomplete outcome data handled ok? | Low risk | Adequate |
| Free of selective reporting? | Low risk | Recruitment reported and this is only outcome needed for review. |
| Was the study free of other bias? | Low risk | No other biases apparent |
| Overall bias? | Low risk | Low risk of bias |

Brown 2015

|  |  |  |
| --- | --- | --- |
| ***Study characteristics*** | | |
| Methods | 2-arm individually-randomised study within a trial | |
| Data | Setting: Not-for-profit health insurance provider, USA. 445 participants were women with pregnancies complicated by gestational diabetes or impaired glucose tolerance. | |
| Comparisons | Investigating a targeted invitation letter for minority groups.  Intervention: Targeted invitation letter, personalized and bilingual in English and Spanish (double-sided), describing the APPLES trial, general diabetes risk, and including specific diabetes risk information based on each recipient's race/ethnicity, as well as pro-diversity statements to encourage participation from diverse backgrounds.  Comparator: The standard letter, bilingual in English and Spanish and personalized with the recipient's name, introduced the APPLES trial, outlined its lifestyle intervention, and provided general diabetes risk information relevant to all populations. | |
| Outcomes | Proportion recruited to host trial | |
| ***Risk of bias*** | | |
| **Bias** | **Authors' judgement** | **Support for judgement** |
| Random sequence generation ok? | Low risk | Following identification in the EHR, women were randomly assigned to receive targeted or standard  recruitment letters in a 1:1 allocation. Block randomization 26 was stratified on medical facility (five groups) and ethnicity (White vs non-White). Randomly selected  assignment sequences were generated in blocks of four for the two ethnic groupings within each facility, and  applied as potentially eligible women were identified over time. Random assignments were automatically date- and time-stamped in a database. |
| Allocation concealment? | Low risk | The authors used pre-generated blocked sequences stratified by centre and ethnicity, with assignments applied automatically through a database. |
| Blinding of participants and personnel ok? | Low risk | “Both women and staff were blind to allocation.” |
| Blinding of outcome assessment ok? | Low risk | Objective - screening rate |
| Incomplete outcome data handled ok? | Low risk | The study had no missing data for the recruitment outcome |
| Free of selective reporting? | Low risk | Recruitment results reported in full |
| Was the study free of other bias? | Low risk | No other suggestion of bias |
| Overall bias? | Low risk | No concerns raised regarding potential sources of bias |

Chen 2011

|  |  |  |
| --- | --- | --- |
| ***Study characteristics*** | | |
| Methods | Randomised controlled trial | |
| Data | Setting: unclear but probably secondary, UK. Participants were eligible for 3 host trials but unclear what the trials were. 2 comparisons against original PIL: 2302 participants in analysis for first, 12,164 participants in analysis for second | |
| Comparisons | Investigated different version of the participant information leaflet (PIL)  Intervention 1: invitation letter with brief summary of PIL  Intervention 2: PIL modified after focus group discussions; enclosed with letter  Comparator: invitation letter with full original PIL | |
| Outcomes | Proportion recruited to pre-randomisation phase of trial | |
| ***Risk of bias*** | | |
| **Bias** | **Authors' judgement** | **Support for judgement** |
| Random sequence generation ok? | Low risk | Conference abstract and limited details. Additional information from co-author R Haynes: randomisation by computer (Haynes 2016). |
| Allocation concealment? | Low risk | As above. R Haynes provided datasets from hospitals with typically thousands of potentially eligible participants and (under section 251 support) we mailed these patients from Cancer Trials Support Unit. The invitations were generated by a computer programme with an incorporated randomisation element (so the different invitations were produced automatically according to the random allocation); this is how allocation was kept concealed so the investigator had no way of knowing what their patients were going to receive. |
| Blinding of participants and personnel ok? | Low risk | Participants definitely blinded. Staff blinding unclear but effect of knowing on recruitment probably minimal |
| Blinding of outcome assessment ok? | Low risk | Objective outcome |
| Incomplete outcome data handled ok? | Low risk | Adequate |
| Free of selective reporting? | Low risk | Recruitment reported, and this is only outcome needed for review |
| Was the study free of other bias? | Low risk | No other biases apparent |
| Overall bias? | Low risk | Low risk of bias |

Chow 2020

|  |  |  |
| --- | --- | --- |
| ***Study characteristics*** | | |
| Methods | 3-arm individually-randomised study within a trial | |
| Data | Setting: Community, USA. 259 participants were adult survivors of childhood cancer identified as high risk for ischemic heart disease or heart failure. | |
| Comparisons | Investigating shortened introductory packets with simplified consent   Intervention 1: A shorter introductory packet included a simplified consent and medical release form (omitting the baseline questionnaire), provided a $25 incentive upfront, and described only the baseline home visit, with further study details and another $25 offered after completion of the home visit.  Intervention 2: An even shorter introductory packet featured a 7-page simplified consent and medical release form, focused solely on the baseline home visit, with a $25 incentive upfront and another $25 offered upon completing the visit, while questionnaires were provided after enrollment by mail, phone, or online.  Comparator: Standard packet containing a cover letter, study brochure, 9-page consent with medical release form, 14-page baseline questionnaire, and an upfront $25 check, with an additional $25 offered after completing the baseline home visit. | |
| Outcomes | Proportion recruited to host trial | |
| ***Risk of bias*** | | |
| **Bias** | **Authors' judgement** | **Support for judgement** |
| Random sequence generation ok? | Unclear risk | “We randomized (1:1:1) the content  and size of materials sent: […]”  Not described for the SWAT |
| Allocation concealment? | Unclear risk | Not described |
| Blinding of participants and personnel ok? | Unclear risk | Participants blinded. Not clear if study staff were blinded and the option of contact by telephone mentioned. It may be possible that unblinded staff are asked to support participants over the ‘phone with decisions linked to the trial but this is unclear. |
| Blinding of outcome assessment ok? | Low risk | Objective outcome |
| Incomplete outcome data handled ok? | Low risk | There were exclusions due to ineligibility to the host trial and not clear if this was done pre- or post-randomisation. I’ve given the team the benefit of the doubt. |
| Free of selective reporting? | Low risk | Recruitment results reported in full |
| Was the study free of other bias? | Low risk | No other suggestion of bias |
| Overall bias? | Unclear risk | Uncertainties raised about randomisation, allocation concealments, and blinding of participants and personnel as not described in the text |

Cockayne 2017 (START PIL REFORM)

|  |  |  |
| --- | --- | --- |
| ***Study characteristics*** | | |
| Methods | Randomised controlled trial | |
| Data | Setting: community NHS clinics, UK. 6900 patients eligible for the REFORM study (over 64 years, routine podiatry appointment in past 6 months) and offered an appointment at NHS podiatry clinics across 5 centres. Ineligible if report neuropathy, dementia or other neurological condition, unable to walk unaided, lower limb amputation, unwilling to attend local podiatry clinic. 3-arm trial of a bespoke user-tested PIL and a template-developed PIL against the usual PIL | |
| Comparisons | Investigated different version of the participant information leaflet (PIL)  Intervention 1: bespoke, user-tested PIL and letter, with graphic design input  Intervention 2: template developed PIL and original study letter with public and patient involvement (PPI) feedback but no user-testing or design input  Comparator: PIL developed for REFORM trial using NRES (ethics) template with study invitation letter | |
| Outcomes | Proportion recruited to trial | |
| ***Risk of bias*** | | |
| **Bias** | **Authors' judgement** | **Support for judgement** |
| Random sequence generation ok? | Low risk | Generated electronically, stratified by centre |
| Allocation concealment? | Low risk | Independent data manager, IDs used, invitation packs sent centrally |
| Blinding of participants and personnel ok? | Low risk | Participants and research staff blinded; not admin staff but unlikely to have affected the allocation |
| Blinding of outcome assessment ok? | Low risk | Objective assessment |
| Incomplete outcome data handled ok? | Low risk | No missing data |
| Free of selective reporting? | Low risk | Recruitment outcome presented, which is all the review needs |
| Was the study free of other bias? | Low risk | No other biases apparent. Sensitivity analysis showed negligible effect of newsletter in pack. May be underpowered |
| Overall bias? | Low risk | Low risk of bias |

Cooper 1997

|  |  |  |
| --- | --- | --- |
| ***Study characteristics*** | | |
| Methods | Randomised controlled trial | |
| Data | Setting: secondary care, UK. 273 first-time attendees at a gynaecological clinic | |
| Comparisons | Investigated the effect of different trial designs  Partially randomised patient preference design allocating to medical management or transcervical resection of the endometrium or preferred option. Comparator was a conventional trial design allocating to medical management or transcervical resection of the endometrium. | |
| Outcomes | Proportion recruited to trial | |
| ***Risk of bias*** | | |
| **Bias** | **Authors' judgement** | **Support for judgement** |
| Random sequence generation ok? | Low risk | Computer-generated list |
| Allocation concealment? | Low risk | Series of sealed, opaque envelopes |
| Blinding of participants and personnel ok? | Low risk | Participants were blinded but not investigators. All participants (intervention and control) were seen by the same trial investigator. Impossible not to unblind investigator since he/she had to know allocation to deliver information to participant |
| Blinding of outcome assessment ok? | Low risk | Objective outcome |
| Incomplete outcome data handled ok? | Low risk | Adequate |
| Free of selective reporting? | Low risk | Recruitment outcome presented, which is all the review needs |
| Was the study free of other bias? | Low risk | No other biases apparent |
| Overall bias? | Low risk | Low risk of bias |

Coyne 2003

|  |  |  |
| --- | --- | --- |
| ***Study characteristics*** | | |
| Methods | Cluster-randomised controlled trial | |
| Data | Setting: secondary care, USA. 226 patients eligible for participation in a cancer treatment trial | |
| Comparisons | Investigated the effect of different consent methods  Easy to read consent statements (altered text style, layout, font size, vocabulary; reading level 7th to 8th grade) were compared to standard consent statements | |
| Outcomes | Proportion recruited to trial | |
| ***Risk of bias*** | | |
| **Bias** | **Authors' judgement** | **Support for judgement** |
| Random sequence generation ok? | Unclear risk | Definitely randomised but unclear how this was done |
| Allocation concealment? | Unclear risk | As above |
| Blinding of participants and personnel ok? | Unclear risk | Nurse clearly knew that the participant had intervention or control consent statement; not clear how much participant was told about the intervention. Not clear if telephone interviewers knew the allocation |
| Blinding of outcome assessment ok? | Low risk | Objective outcome |
| Incomplete outcome data handled ok? | Low risk | Adequate |
| Free of selective reporting? | Low risk | Recruitment outcome presented, which is all the review needs |
| Was the study free of other bias? | Low risk | No other biases apparent |
| Overall bias? | Unclear risk | Unclear risk of bias |

Crane 2016

|  |  |  |
| --- | --- | --- |
| ***Study characteristics*** | | |
| Methods | 2-arm individually-randomised study within a trial | |
| Data | Setting: Community, USA. Participants were 30,000 young adults (18-35 years) with BMI 21.0–30.0 kg/m². | |
| Comparisons | Investigating gender-targeted recruitment postcards.  Intervention 1: Targeted postcard with message “Men: Don’t Settle for Average. The average American gains 30 pounds between the ages of 18 and 35”.    Comparator: Generic postcard with message “Don’t Settle for Average. The average American gains 30 pounds between the ages of 18 and 35”. | |
| Outcomes | Proportion recruited to host trial | |
| ***Risk of bias*** | | |
| **Bias** | **Authors' judgement** | **Support for judgement** |
| Random sequence generation ok? | Low risk | Randomisation was completed using a random number generator and did not stratify on sex of the recipient. |
| Allocation concealment? | Unclear risk | The authors do not provide details on how the allocation sequence was concealed from the staff generating and implementing the strategies. It was also unclear whether staff processing the mailing list could foresee group assignment prior to mailing. |
| Blinding of participants and personnel ok? | Unclear risk | There was no description or reporting of blinding of participants or those administering the intervention. |
| Blinding of outcome assessment ok? | Unclear risk | It is not reported whether outcome assessment was performed by staff blinded to allocation status. Information on blinding or independent assessment is absent. |
| Incomplete outcome data handled ok? | Unclear risk | It is not reported whether all randomised addresses actually received the intervention or whether any were excluded post-randomisation due to undeliverable mail; therefore, completeness of outcome data at the randomised unit level cannot be determined. |
| Free of selective reporting? | Low risk | Data presented in a flow chart |
| Was the study free of other bias? | Unclear risk | Self-reported mailing type was used to classify respondents.   Overall, there is a likely higher risk of contamination for the primary outcome of increasing male response as it was unclear which gender received which postcard. E.g., it was not known the number of women who received the targeted and generic postcard. |
| Overall bias? | Unclear risk | No additional comment |

Dear 2011

|  |  |  |
| --- | --- | --- |
| ***Study characteristics*** | | |
| Methods | Cluster-randomised controlled trial | |
| Data | Setting: secondary care, Australia. 340 participants with cancer who had Internet access | |
| Comparisons | Investigated whether information provided through a website improved recruitment  Intervention: access to a consumer-friendly cancer clinical trials site, which enables people to search for trials  Comparator: usual care (no access to site) | |
| Outcomes | Self-reported (by participant) recruitment to a trial | |
| ***Risk of bias*** | | |
| **Bias** | **Authors' judgement** | **Support for judgement** |
| Random sequence generation ok? | Low risk | Adequate |
| Allocation concealment? | Low risk | Adequate |
| Blinding of participants and personnel ok? | Low risk | Participants were blind to purpose of study. Doctors knew purpose but only intervention group got link to website. |
| Blinding of outcome assessment ok? | Low risk | Assessors were blinded |
| Incomplete outcome data handled ok? | High risk | More than double amount of missing data in intervention group because consultations not recorded and participants not completing follow-up questionnaires. |
| Free of selective reporting? | Low risk | Recruitment reported and this is only outcome needed for review |
| Was the study free of other bias? | Low risk | No other biases apparent |
| Overall bias? | High risk | High risk of bias |

Du 2008

|  |  |  |
| --- | --- | --- |
| ***Study characteristics*** | | |
| Methods | Randomised controlled trial | |
| Data | Setting: secondary care, USA. 126 patients aged 21 to 80 attending multidisciplinary lung clinic at a cancer centre | |
| Comparisons | Investigated the effect of different methods of providing information about the trial  18-minute educational video giving an overview of clinical trials and the importance of cancer clinical research to society. This was compared to standard care (i.e. normal first visit to oncologist). | |
| Outcomes | Proportion recruited to trial | |
| ***Risk of bias*** | | |
| **Bias** | **Authors' judgement** | **Support for judgement** |
| Random sequence generation ok? | Unclear risk | Randomised but no more details |
| Allocation concealment? | Unclear risk | As above |
| Blinding of participants and personnel ok? | Low risk | Oncologist was blinded but the participant was not (not clear if they were told that intervention was a video versus standard care). Outcome objective so probably not a problem |
| Blinding of outcome assessment ok? | Low risk | Objective outcome |
| Incomplete outcome data handled ok? | Low risk | Adequate |
| Free of selective reporting? | Low risk | Recruitment outcome presented, which is all the review needs |
| Was the study free of other bias? | Low risk | No other biases apparent |
| Overall bias? | Unclear risk | Unclear risk of bias |

Du 2009

|  |  |  |
| --- | --- | --- |
| ***Study characteristics*** | | |
| Methods | Randomised controlled trial | |
| Data | Setting: secondary care, USA. 196 women scheduled for treatment evaluation by medical oncology specialist at Karmanos Cancer Institute (KCI) breast clinic. Aged 21 to 80, new female patient at clinic, with diagnosis of histologically confirmed invasive breast cancer, and self-determined as white or African American. Plus: the ability to read and understand English at least at the 6th grade level, the capability to make their own treatment decisions, not having previously participated in a cancer clinical trial, and performance status (PS) B 2 (Southwest Oncology Group (SWOG) scale) | |
| Comparisons | Intervention: 18-minute video. The video presents an overview of phase I, II and III clinical trials and the importance of cancer clinical research to society. The video addresses common concerns regarding clinical trials and cancer treatment from the patient's perspective such as side effects, expected risks and benefits, eligibility criteria, the enrolment process, and treatment costs.    Comparator: usual practice - return to waiting room but not clear what 'standard care' actually is | |
| Outcomes | Enrolment in therapeutic trials | |
| ***Risk of bias*** | | |
| **Bias** | **Authors' judgement** | **Support for judgement** |
| Random sequence generation ok? | Unclear risk | Randomised but no more details |
| Allocation concealment? | Unclear risk | As above |
| Blinding of participants and personnel ok? | Unclear risk | Not clear if staff were blinded, and for participants it depended on what they had been told about study. Participants completed questionnaires themselves so may not have been influenced by staff if staff were unblinded. |
| Blinding of outcome assessment ok? | Low risk | Objective outcome |
| Incomplete outcome data handled ok? | Low risk | Adequate |
| Free of selective reporting? | Low risk | Recruitment outcome presented, which is all the review needs |
| Was the study free of other bias? | Low risk | No other biases apparent |
| Overall bias? | Unclear risk | Unclear risk of bias |

Dwyer 2022

|  |  |  |
| --- | --- | --- |
| ***Study characteristics*** | | |
| Methods | 2-arm individually-randomised study within a trial | |
| Data | Setting: Unclear (possibly community), Republic of Ireland. 207 participants were people with multiple sclerosis, 18 years or older, and had congitive difficulties. | |
| Comparisons | Investigating PPI-designed-and-informed PIL.  Intervention: PPI-designed-and-informed PIL.  Comparator: Standard researcher-designed PIL. | |
| Outcomes | Proportion recruited to host trial, proportion retained at 6 months. | |
| ***Risk of bias*** | | |
| **Bias** | **Authors' judgement** | **Support for judgement** |
| Random sequence generation ok? | Low risk | Individuals interested in further information were randomly allocated to either the SR-PIL or PPIPIL conditions, using 1:1 allocation, via randomised block permutation (i.e. two randomised blocks of four and six per block). Participants were also sent a consent form for the host trial and the outcome measures relevant to the SWAT. SWAT data and formal, written consent were  either posted back to the research team (stamped-addressed envelope provided) or collected by a researcher upon visiting the participant on baseline assessment for the host trial. The researcher who created the randomisation schedule was not involved in posting out material or consenting participants. |
| Allocation concealment? | Low risk | “The researcher who created the randomisation schedule was not involved in posting out material or consenting participants.”  “Both patients and those collecting outcome data were blinded to allocation.” |
| Blinding of participants and personnel ok? | Low risk | “The PIL developers were blinded to each other’s PIL and did not liaise or discuss the PILs during their development, both of which were submitted separately to the principal investigator for subsequent submission for ethical approval. No changes were made by the principal investigator. Both PILs were accompanied by a principal investigator developed addendum regarding GDPR guidelines in order to ensure consistency in this context, for ethical purposes. Both PILs passed ethical approval stage with no changes required.” |
| Blinding of outcome assessment ok? | Low risk | “Both patients and those collecting outcome data were blinded to allocation.” |
| Incomplete outcome data handled ok? | Unclear risk | We wonder whether some of the 64 ineligible had actually consented but were then excluded. Or maybe there was pre-screening. There were 3 exclusions due to COVID but I think that affects secondary outcomes rather than our primary of consent (i.e. recruitment).   Results mention for patient PIL that 62 consented, 11 did not; for researcher PIL data were 63 yes, 7 no. Totalling that gives 143, which is 207 – 64 excluded. |
| Free of selective reporting? | Low risk | No selective reporting. |
| Was the study free of other bias? | Low risk | No other sources of bias were identified. |
| Overall bias? | Unclear risk | Unclear RoB, as some concerns raised about potential incomplete outcome bias. |

Ethier 2017

|  |  |  |
| --- | --- | --- |
| ***Study characteristics*** | | |
| Methods | 2-arm cluster-randomised study within a trial | |
| Data | Setting: General practice in the UK, Netherlands, Belgium, Poland and Greece. 600 participants were adults aged 18-65 years who required proton proton pump inhibitors to treat gastric reflux and had previously had symptoms improved by this medication. | |
| Comparisons | Investigating a software for automatically flagging eligible patients for a trial during GP appointments.  Intervention 1: Practices were equipped with and received training for the TRANSFoRm software, which flagged eligible patients to clinicians, and prepopulated case report forms with electronic health record data.    Comparator: eligible patients for the trial were manually identified via electronic health records. Additionally, case report forms did not have any prepopulated fields. | |
| Outcomes | Proportion recruited to host trial (ony reported for 2 out of 5 countries) | |
| ***Risk of bias*** | | |
| **Bias** | **Authors' judgement** | **Support for judgement** |
| Random sequence generation ok? | Unclear risk | Did not describe randomisation process. |
| Allocation concealment? | Unclear risk | Unclear as authors do not describe the randomisation process. It is therefore unclear if allocation could have been foreseen. Authors did not mention any efforts to conceal allocation. |
| Blinding of participants and personnel ok? | High risk | Authors did not mention any efforts to blind patients, however, clinicians would not have been blind as a new system was installed in the intervention group, which they received training on. |
| Blinding of outcome assessment ok? | Low risk | Enrollment is an objective outcome, therefore it is unlikely that blinding status would have affected SWAT outcomes. |
| Incomplete outcome data handled ok? | High risk | Authors state in the results section that “Eight pairs of practices were available for analysis,” which implies that outcome data was not available for all sites. |
| Free of selective reporting? | High risk | No, the proportion recruited to the SWAT was only reported for Greece and Poland combined (“Greece and Poland were used to compare recruitment rates between the TRANSFoRm arm and the control arm”). |
| Was the study free of other bias? | Low risk | No other sources. |
| Overall bias? | High risk | No additional comment |

Fairhurst 2022

|  |  |  |
| --- | --- | --- |
| ***Study characteristics*** | | |
| Methods | 2x2 factorial individually-randomised study within a trial | |
| Data | Setting: General practice, UK. 818 participants were older adults (65+ years), community-dwelling, and with multimorbidity, defined as having two or more chronic conditions from a predefined list. | |
| Comparisons | Investigating trial-branded pen and unconditional £5 incentive enclosed in trial recruitment pack.  Intervention 1: Trial-branded pen enclosed within the trial recruitment pack and no £5 incentive.  Intervention 2: unconditional £5 incentive enclosed within the trial recruitment pack and no trial-branded pen.  Intervention 3: Trial-branded pen and an unconditional £5 incentive enclosed within the trial recruitment pack.  Comparator: No trial-branded pen and no incentive enclosed within the trial recruitment pack. | |
| Outcomes | Proportion recruited to host trial, cost-effectiveness | |
| ***Risk of bias*** | | |
| **Bias** | **Authors' judgement** | **Support for judgement** |
| Random sequence generation ok? | Low risk | A random sample of invitation packs additionally included a financial incentive and/or a pen as part of this SWAT. |
| Allocation concealment? | Low risk | The participants did not know the alternative allocation. They were hence not informed in advance about the SWAT being embedded in the host trial, i.e. that they may receive a pen or some money in the recruitment pack, and that the incentive they received (if allocated to receive a pack containing one) had been chosen through a process of randomisation. |
| Blinding of participants and personnel ok? | High risk | Neither the participants nor statisticians analysing the data were blinded to allocation. |
| Blinding of outcome assessment ok? | Low risk | The participants were unaware of receiving a specific recruitment strategy, for the purpose of improving patient recruitment in randomised trials. They were hence not informed in advance about the SWAT being embedded in the host trial. |
| Incomplete outcome data handled ok? | Low risk | There is no evidence of missing data in the study. |
| Free of selective reporting? | Low risk | There is no evidence of reporting bias in the study. |
| Was the study free of other bias? | Low risk | No other source of bias has been found in the study. |
| Overall bias? | High risk | High risk of bias as described in individual items above. |

Felicitas-Perkins 2017

|  |  |  |
| --- | --- | --- |
| ***Study characteristics*** | | |
| Methods | 2-arm individually-randomised study within a trial | |
| Data | Setting: Community, USA. Participants were 37 Filipino adults (18+ years) with a clinical trial available to them. | |
| Comparisons | Investigating culturally-tailored educational DVD about clinical trials.  Intervention: Culturally-tailored educational DVD + usual education from a nurse practitioner. The DVD was developed with input from the Filipino community, features bilingual content (Tagalog and Ilokano) on clinical trials and related topics.  Comparator: Usual education from a nurse practitioner about cancer treatments. | |
| Outcomes | Proportion recruited to host trial | |
| ***Risk of bias*** | | |
| **Bias** | **Authors' judgement** | **Support for judgement** |
| Random sequence generation ok? | Unclear risk | Not reported how randomisation was undertaken, whether any stratification, and by which method. |
| Allocation concealment? | Unclear risk | Not reported whether and/or how allocation concealment was attempted. |
| Blinding of participants and personnel ok? | High risk | It would not have been possible to blind participants to the intervention. Nurse practitioners involved in recruiting participants into the study were not blinded, which may have impacted on their delivery of the control intervention. The authors state: ‘experimenter bias may have been introduced into study results if nurse practitioners did not deliver usual education equally between control and intervention participants’. |
| Blinding of outcome assessment ok? | Low risk | For the recruitment outcome, not blinding outcome assessment was unlikely to be a risk. |
| Incomplete outcome data handled ok? | Low risk | The authors report where there are missing data. |
| Free of selective reporting? | Low risk | All outcomes are reported. |
| Was the study free of other bias? | Unclear risk | The authors note the following in the discussion: ‘The Hawthorne effect may further affect results if active engagement from nurse practitioners unduly influenced participant behaviour. Selection bias could have also affected the results of the study, because participants' previous knowledge of clinical trials was not evaluated. Those with a previous understanding of clinical trials may have been more likely to participate in this study’. |
| Overall bias? | High risk | Lack of clarity around random allocation and allocation concealment, and lack of blinding for the nurses delivering the education training to intervention and control participants. |

Ford 2004

|  |  |  |
| --- | --- | --- |
| ***Study characteristics*** | | |
| Methods | Randomised controlled trial | |
| Data | Setting: community, USA. 12,400 African American men aged 55 to 74 eligible for a prostate, lung and colorectal cancer screening trial | |
| Comparisons | Investigated the effect of different trial information and consent methods  Intervention A: enhanced recruitment letter, telephone call by African American interviewer, baseline information by mail, reminder calls/mailings for baseline information/consent  Intervention B: enhanced recruitment letter, telephone call by African American interviewer, baseline information over telephone, reminder calls/mailings for consent form  Intervention C: enhanced recruitment letter, telephone call by African American interviewer, church session, baseline information at church session  Compared to standard recruitment letter, telephone assessment by African American or white interviewer, baseline information by mail, reminder calls/mailings for baseline information/consent | |
| Outcomes | Proportion recruited to trial | |
| ***Risk of bias*** | | |
| **Bias** | **Authors' judgement** | **Support for judgement** |
| Random sequence generation ok? | Unclear risk | Randomised but no more details |
| Allocation concealment? | Unclear risk | As above |
| Blinding of participants and personnel ok? | Unclear risk | Potential participants were blinded but the researchers probably were not blinded |
| Blinding of outcome assessment ok? | Low risk | Objective outcome |
| Incomplete outcome data handled ok? | Low risk | Adequate |
| Free of selective reporting? | Low risk | Recruitment outcome presented, which is all the review needs |
| Was the study free of other bias? | Low risk | No other biases apparent |
| Overall bias? | Unclear risk | Unclear risk of bias |

Foss 2016

|  |  |  |
| --- | --- | --- |
| ***Study characteristics*** | | |
| Methods | Randomised controlled trial | |
| Data | Setting: secondary care, Denmark. 118 women giving birth at 1 of 3 hospitals and eligible for the Danish Calmette Study | |
| Comparisons | Investigated the effect of different trial information and consent methods | |
| Outcomes | Proportion recruited to trial | |
| ***Risk of bias*** | | |
| **Bias** | **Authors' judgement** | **Support for judgement** |
| Random sequence generation ok? | Low risk | Central, web-based block-randomisation with variable block sizes of 2, 4, and 6 in random order |
| Allocation concealment? | Low risk | See above |
| Blinding of participants and personnel ok? | Low risk | Participants blinded although staff giving information were not , though they followed an SOP regarding what to say. Probably didn't affect outcome |
| Blinding of outcome assessment ok? | Low risk | Outcome objective |
| Incomplete outcome data handled ok? | Low risk | Adequate |
| Free of selective reporting? | Low risk | Recruitment outcome presented, which is all the review needs |
| Was the study free of other bias? | Low risk | No other biases apparent |
| Overall bias? | Low risk | Low risk of bias |

Fowell 2006

|  |  |  |
| --- | --- | --- |
| ***Study characteristics*** | | |
| Methods | Cluster-randomised cross-over trial | |
| Data | Setting: secondary care, UK. 53 Cancer inpatients receiving palliative care and starting on a syringe driver | |
| Comparisons | Investigated the effect of different trial designs  Cluster-randomisation compared to Zelen's design (in which only those randomised to the intervention group were asked for consent) | |
| Outcomes | Proportion recruited to trial | |
| ***Risk of bias*** | | |
| **Bias** | **Authors' judgement** | **Support for judgement** |
| Random sequence generation ok? | Low risk | Coin-tossing for initial allocation to cluster or Zelen (2 sites only) |
| Allocation concealment? | Low risk | Only 2 sites and allocation to intervention (Zelen or cluster) by coin toss almost certainly done centrally |
| Blinding of participants and personnel ok? | Low risk | Blinding only partial, but looking at the effect of open study design was the purpose of the study |
| Blinding of outcome assessment ok? | Low risk | Objective outcome |
| Incomplete outcome data handled ok? | Low risk | Adequate |
| Free of selective reporting? | Low risk | Recruitment outcome presented, which is all the review needs |
| Was the study free of other bias? | Low risk | No other biases apparent |
| Overall bias? | Low risk | Low risk of bias |

Fracasso 2013

|  |  |  |
| --- | --- | --- |
| ***Study characteristics*** | | |
| Methods | Randomised controlled trial | |
| Data | Setting: secondary care, USA. Participants were 60 patients with cancer recruited through the Siteman Cancer Center (SCC). Patients were identified by their medical, radiation, or surgical oncologist at the time of evaluation for treatment. Patients were ≥ 18 years of age; English speaking; self-reported as a member of a racial or ethnic minority; diagnosed with advanced breast, colorectal, lung, or prostate carcinoma with an Eastern Cooperative Oncology Group (ECOG) performance status of 0 to 2 | |
| Comparisons | Investigated coaching as a way of improving recruitment  Intervention: African American coach providing individualised, flexible education and support to create context of trust promoting trial enrollment  Comparator: no coach (usual care) | |
| Outcomes | Proportion recruited to trial | |
| ***Risk of bias*** | | |
| **Bias** | **Authors' judgement** | **Support for judgement** |
| Random sequence generation ok? | Unclear risk | Says randomly allocated but nothing more |
| Allocation concealment? | Unclear risk | As above |
| Blinding of participants and personnel ok? | Unclear risk | Not clear what participants knew about the intervention prior to being randomised; all provided consent so they were told something |
| Blinding of outcome assessment ok? | Low risk | Objective outcome (recruitment) |
| Incomplete outcome data handled ok? | Low risk | 6 died or were lost to follow-up, but not clear which groups they were in. But unlikely due to intervention. |
| Free of selective reporting? | Low risk | Recruitment reported, and this is only outcome needed for review |
| Was the study free of other bias? | Low risk | No other biases apparent |
| Overall bias? | Unclear risk | Unclear risk of bias |

Free 2010

|  |  |  |
| --- | --- | --- |
| ***Study characteristics*** | | |
| Methods | Randomised controlled trial | |
| Data | Setting: community, UK. Participants were 1302 daily smokers, 16 or over, wanting to stop smoking in next month | |
| Comparisons | Investigated whether including GBP 5 with invitation or sending SMS messages to potential participants increased recruitment  Intervention A: GBP 5 with participant info sheet and consent form    Intervention B: series of 4 text messages with quotes from existing participants    Comparator: normal trial procedures - letter with participant information sheet and consent form | |
| Outcomes | Proportion recruited to trial | |
| ***Risk of bias*** | | |
| **Bias** | **Authors' judgement** | **Support for judgement** |
| Random sequence generation ok? | Low risk | For the 2 trials covered in this review the data manager placed registration ID numbers of participants in ascending numerical order and alternate participants were allocated systematically to the intervention or control group. The ID numbers were not linked to any names or other personally identifying information, so allocation was concealed.    Additional information from the study author: all the data manager had was a list of numbers with no other linked information. The order of numbers were generated by the timing of recruitment to the txt2stop randomisation. The allocation could be checked, i.e. there was no way of manipulating it. |
| Allocation concealment? | Low risk | Central (web-based)/data manager |
| Blinding of participants and personnel ok? | Low risk | Participants blind but not research staff, unlikely to affect outcome measurement (assessment was blinded) |
| Blinding of outcome assessment ok? | Low risk | Objective outcome and assessors were blind |
| Incomplete outcome data handled ok? | Low risk | Adequate |
| Free of selective reporting? | Low risk | Registration to trial outcome presented, which is all the review needs |
| Was the study free of other bias? | Low risk | No other biases apparent |
| Overall bias? | Low risk | Low risk of bias |

Free 2011

|  |  |  |
| --- | --- | --- |
| ***Study characteristics*** | | |
| Methods | Randomised controlled trial | |
| Data | Setting: primary care, UK. Participants were 1592 smokers eligible for a smoking cessation trial | |
| Comparisons | Investigated effect of mentioning scarcity on recruitment  Intervention: SMS reminder message including scarcity message 'only 300 places left'  Comparator: SMS reminder without mention of scarcity | |
| Outcomes | Proportion recruited to trial | |
| ***Risk of bias*** | | |
| **Bias** | **Authors' judgement** | **Support for judgement** |
| Random sequence generation ok? | Low risk | Adequate |
| Allocation concealment? | Low risk | Adequate |
| Blinding of participants and personnel ok? | Low risk | Adequate |
| Blinding of outcome assessment ok? | Low risk | Adequate |
| Incomplete outcome data handled ok? | Low risk | Adequate |
| Free of selective reporting? | Low risk | Recruitment reported and this is only outcome needed for review |
| Was the study free of other bias? | Low risk | No other biases apparent |
| Overall bias? | Low risk | Low risk of bias |

Garvelink 2015

|  |  |  |
| --- | --- | --- |
| ***Study characteristics*** | | |
| Methods | 2-arm cluster-randomised study within a trial | |
| Data | Setting: Mixed (community, long-term care facilities, hospitals), Canada. Participants were elderly patients (65+ years) receiving home care, and have faced the decision to stay at home or move to a care facility within the past 3 to 6 months. 33 healthcare organitations were included as clusters. | |
| Comparisons | Investigating pre-engagement with potential trial sites during proposal development.  Intervention: Pre-engagement of healthcare organizations. This involved contacting directors of homecare organizations with a draft abstract of the study proposal, discussing study details by phone, requesting a letter describing their support for the study and their intention to participate should it be funded, and, once funded, re-contacting directors to confirm their participation in the randomized IP-SDM intervention study.  Comparator: Engagement with sites after funding approval and asked to confirm their participation in the study. | |
| Outcomes | Proportion recruited to host trial | |
| ***Risk of bias*** | | |
| **Bias** | **Authors' judgement** | **Support for judgement** |
| Random sequence generation ok? | Low risk | A computer generated random list of the order in which to contact directors of eligible Health and Social Services Centres (HSSCs) was created. |
| Allocation concealment? | Low risk | No data reported on any procedure for concealing this sequence from the staff implementing the intervention. There is no mention of central allocation or other concealment mechanisms |
| Blinding of participants and personnel ok? | Unclear risk | The authors do not report celarly on this. . However, blinding of personnel was not possible due to the nature of the intervention. While agreement to participate is an objective outcome, it is unclear if non-standardised contact procedures introduced bias. |
| Blinding of outcome assessment ok? | Low risk | No blinding of outcome assessment, but this is unlikely to have had an impact on the outcome. |
| Incomplete outcome data handled ok? | Low risk | No incomplete outcome data - all outcome data reported. |
| Free of selective reporting? | Low risk | Reporting addressed the main study aim. |
| Was the study free of other bias? | Low risk | No other biases identified. |
| Overall bias? | Unclear risk | This study has unclear risk of bias due to unclear allocation concealment and unclear blinding |

Giguere 2015

|  |  |  |
| --- | --- | --- |
| ***Study characteristics*** | | |
| Methods | 2-arm individually-randomised study within a trial | |
| Data | Setting: General practice, Canada. Participants were 1314 family physicians listed in the 2004–5 directory of the College of Physicians of the Province of Quebec (Canada) with a valid email address. | |
| Comparisons | Investigating $300 monetary incentive.  Intervention: $300 monetary incentive provided to physicians being recruited as participants in a trial.  Comparator: No incentive. | |
| Outcomes | Proportion recruited to host trial, | |
| ***Risk of bias*** | | |
| **Bias** | **Authors' judgement** | **Support for judgement** |
| Random sequence generation ok? | Low risk | All physicians were randomly allocated to either group simultaneously using free online software (http://www.randomizer.org/), ensuring allocation concealment. |
| Allocation concealment? | Low risk | All physicians were randomly allocated to either group simultaneously using free online software (http://www.randomizer.org/), ensuring allocation concealment. |
| Blinding of participants and personnel ok? | Low risk | “The Office of Continuing Medical Education at Laval University in Quebec  City, Canada, emailed all of these physicians, inviting them to participate in a study to evaluate two formats of  an online CME program on rheumatology.”  Not clear what site emailing out invitations were told about the study or who sent the emails (office staff or study staff). Participants are blind but personnel are unlikely to influence the outcome. |
| Blinding of outcome assessment ok? | Low risk | Objective outcome |
| Incomplete outcome data handled ok? | Low risk | The study had no missing data for the recruitment outcome |
| Free of selective reporting? | Low risk | Recruitment results reported in full. |
| Was the study free of other bias? | Low risk | No other suggestion of bias |
| Overall bias? | Low risk | No concerns raised about potential sources of bias. |

Halpern 2021a ($200)

|  |  |  |
| --- | --- | --- |
| ***Study characteristics*** | | |
| Methods | 3-arm individually-randomised study within a trial | |
| Data | Setting: Smoking cessation clinics in 2 health systems, USA. Participants were 654 adult daily cigarette smokers with major depressive disorder. | |
| Comparisons | Investigating higher versus lower monetary incentive versus nothing.  Intervention 1: $200 monetary incentive.  Intervention 2: $500 monetary incentive.  Comparator: No monetary incentive ($0). | |
| Outcomes | Proportion recruited to host trial | |
| ***Risk of bias*** | | |
| **Bias** | **Authors' judgement** | **Support for judgement** |
| Random sequence generation ok? | Unclear risk | Not enough information to make judgement.  “Within strata, variable block sizes of 3 and 6 were used.” |
| Allocation concealment? | Unclear risk | Not enough information to make judgement as above. |
| Blinding of participants and personnel ok? | High risk | Recruiting staff were not blinded.  “Research coordinators administering the consent processes in both parent trials were unmasked to the incentive information but adhered to IRB-approved communication scripts.”   “Coordinators used scripted probes to assess whether patients had heard about the use of incentives in the trial. Patients who indicated any prior familiarity were deemed ineligible for RETAIN.” |
| Blinding of outcome assessment ok? | Low risk | “proportion of people assigned to each recruitment strategy that consented to participate.” – objective outcome |
| Incomplete outcome data handled ok? | Low risk | The study had no missing data for the recruitment outcome. |
| Free of selective reporting? | Low risk | Recruitment results reported in full. |
| Was the study free of other bias? | Low risk | No other suggestion of bias. |
| Overall bias? | High risk | Concerns raised about lack of blinding of recruiting staff. Insufficient data regarding random sequence generation and allocation concealment. |

Halpern 2021a ($500)

|  |  |  |
| --- | --- | --- |
| ***Study characteristics*** | | |
| Methods | Published data only (unpublished not sought) | |
| Data | Setting: Smoking cessation clinics in 2 health systems, USA. Participants were 654 adult daily cigarette smokers with major depressive disorder. | |
| Comparisons | Investigating higher versus lower monetary incentive versus nothing.  Intervention 1: $200 monetary incentive.  Intervention 2: $500 monetary incentive.  Comparator: No monetary incentive ($0). | |
| Outcomes | Proportion recruited to host trial | |
| ***Risk of bias*** | | |
| **Bias** | **Authors' judgement** | **Support for judgement** |
| Random sequence generation ok? | Unclear risk | Not enough information to make judgement.  “Within strata, variable block sizes of 3 and 6 were used.” |
| Allocation concealment? | Unclear risk | Not enough information to make judgement as above. |
| Blinding of participants and personnel ok? | High risk | Recruiting staff were not blinded.  “Research coordinators administering the consent processes in both parent trials were unmasked to the incentive information but adhered to IRB-approved communication scripts.”  “Coordinators used scripted probes to assess whether patients had heard about the use of incentives in the trial. Patients who indicated any prior familiarity were  deemed ineligible for RETAIN.” |
| Blinding of outcome assessment ok? | Low risk | “proportion of people assigned to each recruitment strategy that consented to participate.” – objective outcome |
| Incomplete outcome data handled ok? | Low risk | The study had no missing data for the recruitment outcome. |
| Free of selective reporting? | Low risk | Recruitment results reported in full. |
| Was the study free of other bias? | Low risk | No other suggestion of bias. |
| Overall bias? | High risk | Concerns raised about lack of blinding of recruiting staff. Insufficient data regarding random sequence generation and allocation concealment. |

Halpern 2021b ($100)

|  |  |  |
| --- | --- | --- |
| ***Study characteristics*** | | |
| Methods | 3-arm individually-randomised study within a trial | |
| Data | Setting: Hospital, USA. Participants were 642 adults (18+ years) admitted to medicine or oncology floors. | |
| Comparisons | Investigating higher versus lower monetary incentive versus nothing.  Intervention 1: $100 monetary incentive.  Intervention 2: $300 monetary incentive.  Comparator: No monetary incentive ($0). | |
| Outcomes | Proportion recruited to host trial | |
| ***Risk of bias*** | | |
| **Bias** | **Authors' judgement** | **Support for judgement** |
| Random sequence generation ok? | Unclear risk | Not enough information to make judgement.  “Within strata, variable block sizes of 3 and 6 were used. |
| Allocation concealment? | Unclear risk | Not enough information to make judgement as above. |
| Blinding of participants and personnel ok? | High risk | Recruiting staff were not blinded.  “Research coordinators administering the consent processes in both parent trials were unmasked to the incentive information but adhered to IRB-approved communication scripts.”  “Coordinators used scripted probes to assess whether patients had heard about the use of incentives in the trial. Patients who indicated any prior familiarity were deemed ineligible for RETAIN.” |
| Blinding of outcome assessment ok? | Low risk | “proportion of people assigned to each recruitment strategy that consented to participate.” – objective outcome |
| Incomplete outcome data handled ok? | Low risk | The study had no missing data for the recruitment outcome. |
| Free of selective reporting? | Low risk | Recruitment results reported in full. |
| Was the study free of other bias? | Low risk | No other suggestion of bias. |
| Overall bias? | High risk | Concerns raised about lack of blinding of recruiting staff. Insufficient data regarding random sequence generation and allocation concealment. |

Halpern 2021b ($300)

|  |  |  |
| --- | --- | --- |
| ***Study characteristics*** | | |
| Methods | 3-arm individually-randomised study within a trial | |
| Data | Setting: Hospital, USA. Participants were 642 adults (18+ years) admitted to medicine or oncology floors. | |
| Comparisons | Investigating higher versus lower monetary incentive versus nothing.  Intervention 1: $100 monetary incentive.  Intervention 2: $300 monetary incentive.  Comparator: No monetary incentive ($0). | |
| Outcomes | Proportion recruited to host trial | |
| ***Risk of bias*** | | |
| **Bias** | **Authors' judgement** | **Support for judgement** |
| Random sequence generation ok? | Unclear risk | Not enough information to make judgement.  “Within strata, variable block sizes of 3 and 6 were used.” |
| Allocation concealment? | Unclear risk | Not enough information to make judgement as above. |
| Blinding of participants and personnel ok? | High risk | Recruiting staff were not blinded.  “Research coordinators administering the consent processes in both parent trials were unmasked to the incentive information but adhered to IRB-approved communication scripts.”  “Coordinators used scripted probes to assess whether patients had heard about the use of incentives in the trial. Patients who indicated any prior familiarity were deemed ineligible for RETAIN.” |
| Blinding of outcome assessment ok? | Low risk | “proportion of people assigned to each recruitment strategy that consented to participate.” – objective outcome |
| Incomplete outcome data handled ok? | Low risk | The study had no missing data for the recruitment outcome. |
| Free of selective reporting? | Low risk | Recruitment results reported in full. |
| Was the study free of other bias? | Low risk | No other suggestion of bias. |
| Overall bias? | High risk | Concerns raised about lack of blinding of recruiting staff. Insufficient data regarding random sequence generation and allocation concealment. |

Hemminki 2004

|  |  |  |
| --- | --- | --- |
| ***Study characteristics*** | | |
| Methods | Randomised controlled trial | |
| Data | Setting: 'local clinics', Estonia. 4295 postmenopausal women aged 50 to 64 | |
| Comparisons | Investigated the effect of different design methods  Non-blinded allocation comparing active HRT treatment versus no treatment. This was compared to traditional blinded allocation comparing active HRT treatment versus placebo. | |
| Outcomes | Proportion recruited to trial | |
| ***Risk of bias*** | | |
| **Bias** | **Authors' judgement** | **Support for judgement** |
| Random sequence generation ok? | Low risk | Computer-based random number sequence |
| Allocation concealment? | Low risk | Sealed opaque envelope with ID on it |
| Blinding of participants and personnel ok? | Low risk | Blinding only partial but looking at the effect of open study design was the purpose of the study |
| Blinding of outcome assessment ok? | Low risk | Partial (see above) but objective outcome |
| Incomplete outcome data handled ok? | Low risk | Adequate |
| Free of selective reporting? | Low risk | Recruitment outcome presented, which is all the review needs |
| Was the study free of other bias? | Low risk | No other biases apparent |
| Overall bias? | Low risk | Low risk of bias |

Hughes-Morley 2016

|  |  |  |
| --- | --- | --- |
| ***Study characteristics*** | | |
| Methods | Cross-factorial, cluster-randomised study within a trial | |
| Data | Setting: Community Mental Health Teams, UK. Participants were 8,182 adults (18+ years) under the care of community mental health teams. | |
| Comparisons | Investigating leaflet advertising patient and public involvement.  Intervention: Booklet style leaflet advertising patient and public involvement in research (PPIR).  Comparator: Standard trial invitation. | |
| Outcomes | Proportion recruited to host trial | |
| ***Risk of bias*** | | |
| **Bias** | **Authors' judgement** | **Support for judgement** |
| Random sequence generation ok? | Low risk | “Randomisation was undertaken by the host trial statisticians (NO’L and CR), who were independent from the delivery of the trial interventions for both the host and embedded recruitment trials. Randomisation in the EQUIP host trial was stratified by cluster pairing, the site/region of each cluster and the caseload size of each cluster. For the recruitment trial, we used the same cluster pairing as the host trial and allocated each cluster pair by block-randomisation with permuted block sizes of 2, 4 and 6, using a computerised randomisation programme.” |
| Allocation concealment? | Low risk | “Service users did not know that they were part of a trial of a recruitment intervention so were blind to the study hypothesis.” |
| Blinding of participants and personnel ok? | Low risk | “CSOs and research nurses undertaking trial recruitment and mental health team clusters were also blind to the group to which clusters were allocated.” |
| Blinding of outcome assessment ok? | Low risk | Outcomes pre-determined and analysing group distinct from assessment group |
| Incomplete outcome data handled ok? | Low risk | Patient satisfaction with clinical care data not included in model due to incomplete data |
| Free of selective reporting? | Low risk | Outcomes pre-determined and all reported |
| Was the study free of other bias? | Low risk | No other source of bias identified. |
| Overall bias? | Low risk | Although there was no information on how incomplete data regarding patient satisfaction were managed, the authors excluded patient satisfaction with clinical care in the model due to incompleteness. The study was otherwise well randomised and blinded. Patient satisfaction was not a primary outcome and the attrition bias related to this was therefore not felt to be significant enough to affect the overall risk of bias. |

Hutchison 2007

|  |  |  |
| --- | --- | --- |
| ***Study characteristics*** | | |
| Methods | Randomised controlled trial | |
| Data | Setting: secondary care, UK. 173 patients with colorectal, breast, lung cancer and clinically eligible to enter 1 of centre's trials; access to a video recorder, CD-ROM or DVD player; can understand English | |
| Comparisons | Investigating providing trial information by video.  Intervention: video covering general trial info, randomisation, pictures of patients receiving care + voiceover discussing uncertainty + standard practice (clinician discussing treatment options and possibility of taking part in a trial) + standard practice   Comparator: standard practice (clinician discussing treatment options and possibility of taking part in a trial) | |
| Outcomes | Proportion recruited to trial | |
| ***Risk of bias*** | | |
| **Bias** | **Authors' judgement** | **Support for judgement** |
| Random sequence generation ok? | Low risk | Minimisation in Oracle database done by clinical trials unit |
| Allocation concealment? | Low risk | Centrally by CTU |
| Blinding of participants and personnel ok? | Low risk | Not clear if patients know about video versus normal info when consenting. Staff may also be unblinded although materials are sent to them at home and all participants receive standard care so probably small chance of introducing bias. |
| Blinding of outcome assessment ok? | Low risk | Partial (see above) but objective outcome |
| Incomplete outcome data handled ok? | Low risk | Adequate |
| Free of selective reporting? | Low risk | Recruitment outcome presented, which is all the review needs |
| Was the study free of other bias? | Low risk | No other biases apparent |
| Overall bias? | Low risk | Low risk of bias |

Isaksson 2025

|  |  |  |
| --- | --- | --- |
| ***Study characteristics*** | | |
| Methods | Crossover cluster-randomised study within a trial | |
| Data | Setting: Tertiary care (acute stroke units, rehabilitation centres, and geriatric rehabilitation centres), Sweden. Participants were stroke patients. 20 centres | |
| Comparisons | Investigating teleconference with recruiting centres and commitment contracts.  Intervention: Centres were invited to a structured teleconference, which included a presentation on the host trial and discussions addressing local recruitment barriers and strategies for improvement. This was accompanied by a commitment contract.   Comparator: No teleconference (control rates before intervention). | |
| Outcomes | Number of included subjects before and after intervention | |
| ***Risk of bias*** | | |
| **Bias** | **Authors' judgement** | **Support for judgement** |
| Random sequence generation ok? | Low risk | “The statistician used stratified block randomisation in each group to allocate the 20 centres into 10 groups of two or three centres per step, leading to (at least) one medium and one low recruiting centre in each step. Because two centres were closed between randomisation and the intervention, two groups finally consisted of one centre. The time at which the centres started the intervention was randomly allocated.” |
| Allocation concealment? | Unclear risk | “The participants were not informed about the aim of ERUTECC or that the timing of the intervention was randomised or that we measured numbers of randomised patients before and after intervention, but they were fully aware of the fact that we wanted to enhance recruitment.” |
| Blinding of participants and personnel ok? | Low risk | “The participants were not informed about the aim of ERUTECC or that the timing of the intervention was randomised or that we measured numbers of randomised patients before and after intervention, but they were fully aware of the fact that we wanted to enhance recruitment. The exact numbers of recruitment per centre were available at the public domain through a link that was updated in real time since the start of the EFFECTS study.” |
| Blinding of outcome assessment ok? | Low risk | “The participants were not informed about the aim of ERUTECC or that the timing of the intervention was randomised or that we measured numbers of randomised patients before and after intervention, but they were fully aware of the fact that we wanted to enhance recruitment. The exact numbers of recruitment per centre were available at the public domain through a link that was updated in real time since the start of the EFFECTS study.” |
| Incomplete outcome data handled ok? | Low risk | Recruitment outcomes were available for all randomised clusters. There was no indication of missing data or exclusion of sites following allocation, and all centres were included in the final analysis. |
| Free of selective reporting? | Low risk | Reported outcomes same as protocol |
| Was the study free of other bias? | Low risk | No additional sources of bias identified. |
| Overall bias? | Unclear risk | Study participants were aware of the outcome being studied but were otherwise adequately blinded and randomised to the different study arms. It is unclear if, and to what extent, this may have influenced the relevant findings of the study. All other RoB domains were rated as low risk of bias. |

Ives 2001

|  |  |  |
| --- | --- | --- |
| ***Study characteristics*** | | |
| Methods | Randomised controlled trial | |
| Data | Setting: secondary care, UK. 50 patients attending an HIV hospital clinic | |
| Comparisons | Investigated the effect of different trial information methods  Standard trial information plus booklet entitled, 'Clinical Trials in HIV and AIDS: Information for people who are thinking about joining a trial'. This was compared to standard trial information (information sheet specific to proposed trial, plus discussion with trial doctor and research nurse) | |
| Outcomes | Proportion recruited to trial | |
| ***Risk of bias*** | | |
| **Bias** | **Authors' judgement** | **Support for judgement** |
| Random sequence generation ok? | Low risk | Randomisation done sequence of numbered envelopes |
| Allocation concealment? | Low risk | See above |
| Blinding of participants and personnel ok? | Low risk | Patients and investigators not blinded. Not clear if interviewers were the investigators and therefore blind or unblinded. Unlikely to have affected outcome |
| Blinding of outcome assessment ok? | Low risk | Partial (see above) but objective outcome |
| Incomplete outcome data handled ok? | Unclear risk | 50 were randomised but outcome data available for only 31, most of whom had joined a trial. There were some difference between those who provide only baseline data and those who provided follow-up data. Not clear if there were differences between groups |
| Free of selective reporting? | Low risk | Recruitment outcome presented, which is all the review needs |
| Was the study free of other bias? | Low risk | No other biases apparent |
| Overall bias? | Unclear risk | Unclear risk of bias |

Jefferson 2018

|  |  |  |
| --- | --- | --- |
| ***Study characteristics*** | | |
| Methods | 2-arm cluster-randomised study within a trial | |
| Data | Setting: Hospital (outpatient clinic), UK. Participants were recruited from 37 sites and were patients aged 16 years or above with a wrist fracture. | |
| Comparisons | Investigating remote site initiation visits.  Intervention : Remote site initiation visit (SIV), which involved standardized initial meetings via telephone or video conference with key hospital site staff, using a uniform presentation and checklist to prepare for study approval.    Comparator: On-site SIV. | |
| Outcomes | Proportion recruited to host trial, proportion retained at each followup time point including 12 months (host trial primary endpoint), intervention costs. | |
| ***Risk of bias*** | | |
| **Bias** | **Authors' judgement** | **Support for judgement** |
| Random sequence generation ok? | Low risk | Randomisation was done via minimisation; selection bias not possible. |
| Allocation concealment? | Low risk | As above, randomisation was done via minimisation, selection bias not possible. |
| Blinding of participants and personnel ok? | Low risk | Not feasible – but sites weren’t aware of their involvement in the SWAT, so no risk of bias. |
| Blinding of outcome assessment ok? | Low risk | Outcome data is routinely collected data, so not possible to have been influenced. Sites were not aware of their involvement. |
| Incomplete outcome data handled ok? | Low risk | Missing data due only to sites not participating; no evidence of bias. |
| Free of selective reporting? | Low risk | All data reported with the manuscript; no evidence of bias. |
| Was the study free of other bias? | Low risk | No other sources of bias noted. |
| Overall bias? | Low risk | No concerns about potential sources of bias raised. |

Jennings 2015a

|  |  |  |
| --- | --- | --- |
| ***Study characteristics*** | | |
| Methods | Randomised controlled trial | |
| Data | Setting: primary care, UK. Participants were 181 people who were over 60 taking long-term NSAIDS for arthritis. | |
| Comparisons | Investigated effect of financial incentive on recruitment  Intervention: offer of GBP 100  Comparison: no offer | |
| Outcomes | Proportion recruited to trial, cost-effectiveness | |
| ***Risk of bias*** | | |
| **Bias** | **Authors' judgement** | **Support for judgement** |
| Random sequence generation ok? | Low risk | Done centrally using a computer algorithm. There was a slight imbalance in favour of control because of algorithm used but allocation still random |
| Allocation concealment? | Low risk | Done centrally |
| Blinding of participants and personnel ok? | Low risk | Research nurses and staff not blinded but interventions sent out to patients on GP list so staff could not influence response. Patients blinded |
| Blinding of outcome assessment ok? | Low risk | Objective outcome |
| Incomplete outcome data handled ok? | Low risk | Adequate |
| Free of selective reporting? | Low risk | Recruitment data reported, which is all the review needs |
| Was the study free of other bias? | Low risk | No other biases apparent |
| Overall bias? | Low risk | Low risk of bias |

Jennings 2015b

|  |  |  |
| --- | --- | --- |
| ***Study characteristics*** | | |
| Methods | Randomised controlled trial | |
| Data | Setting: primary care, UK. Participants were 332 people who were aged over 60 with symptomatic hyperuricaemia. | |
| Comparisons | Investigated effect of financial incentive on recruitment  Intervention: offer of GBP 100  Comparison: no offer | |
| Outcomes | Proportion recruited to trial, cost-effectiveness | |
| ***Risk of bias*** | | |
| **Bias** | **Authors' judgement** | **Support for judgement** |
| Random sequence generation ok? | Low risk | Done centrally using the computer algorithm. There was a slight imbalance in favour of control because of algorithm used but allocation still random |
| Allocation concealment? | Low risk | Done centrally |
| Blinding of participants and personnel ok? | Low risk | Research nurses and staff not blinded but invitations sent out to patients on GP list so staff could not influence response. Participants blinded |
| Blinding of outcome assessment ok? | Low risk | Objective outcome |
| Incomplete outcome data handled ok? | Low risk | Adequate |
| Free of selective reporting? | Low risk | Recruitment data reported, which is all the review needs |
| Was the study free of other bias? | Low risk | No other biases apparent |
| Overall bias? | Low risk | Low risk of bias |

Jennings 2015c

|  |  |  |
| --- | --- | --- |
| ***Study characteristics*** | | |
| Methods | Randomised controlled trial | |
| Data | Setting: primary care, UK. Participants were 93 people who were aged 18 to 79 years comparing monotherapy with dual therapy as initial hypertension treatment. | |
| Comparisons | Investigated effect of financial incentive on recruitment.  Intervention: offer of GBP 100  Comparison: no offer | |
| Outcomes | Proportion recruited to trial, cost-effectiveness | |
| ***Risk of bias*** | | |
| **Bias** | **Authors' judgement** | **Support for judgement** |
| Random sequence generation ok? | Low risk | Done centrally using computer algorithm. There was a slight imbalance in favour of control because of algorithm used but allocation still random |
| Allocation concealment? | Low risk | Done centrally |
| Blinding of participants and personnel ok? | Low risk | Research nurses and staff not blinded but invitations sent out to patients on GP list so staff could not influence response. Participants blinded |
| Blinding of outcome assessment ok? | Low risk | Objective outcome |
| Incomplete outcome data handled ok? | Low risk | Adequate |
| Free of selective reporting? | Low risk | Recruitment data reported, which is all the review needs |
| Was the study free of other bias? | Low risk | No other biases apparent |
| Overall bias? | Low risk | Low risk of bias |

Jennings 2015d

|  |  |  |
| --- | --- | --- |
| ***Study characteristics*** | | |
| Methods | Randomised controlled trial | |
| Data | Setting: primary care, UK. Participants were 210 people who were aged 18 to 79 years with uncontrolled blood pressure on 3 antihypertensive agents. | |
| Comparisons | Investigated effect of financial incentive on recruitment  Intervention: offer of GBP 100  Comparison: no offer | |
| Outcomes | Proportion recruited to trial, cost-effectiveness | |
| ***Risk of bias*** | | |
| **Bias** | **Authors' judgement** | **Support for judgement** |
| Random sequence generation ok? | Low risk | Done centrally using computer algorithm. There was a slight imbalance in favour of control because of algorithm used but allocation still random |
| Allocation concealment? | Low risk | Done centrally |
| Blinding of participants and personnel ok? | Low risk | Research nurses and staff not blinded but invitations sent out to patients on GP list so staff could not influence response. Participants blinded |
| Blinding of outcome assessment ok? | Low risk | Objective outcome |
| Incomplete outcome data handled ok? | Low risk | Adequate |
| Free of selective reporting? | Low risk | Recruitment data reported, which is all the review needs |
| Was the study free of other bias? | Low risk | No other biases apparent |
| Overall bias? | Low risk | Low risk of bias |

Jennings 2015e

|  |  |  |
| --- | --- | --- |
| ***Study characteristics*** | | |
| Methods | Randomised controlled trial | |
| Data | Setting: primary care, UK. Participants were 199 people who were 18 to 80 years with at least 1 component of the metabolic syndrome. | |
| Comparisons | Investigated effect of financial incentive on recruitment  Intervention: offer of GBP 100  Comparison: no offer | |
| Outcomes | Proportion recruited to trial, cost-effectiveness | |
| ***Risk of bias*** | | |
| **Bias** | **Authors' judgement** | **Support for judgement** |
| Random sequence generation ok? | Low risk | Done centrally using computer algorithm. There was a slight imbalance in favour of control because of algorithm used but allocation still random |
| Allocation concealment? | Low risk | Done centrally |
| Blinding of participants and personnel ok? | Low risk | Research nurses and staff not blinded but invitations sent out to patients on GP list so staff can not influence response. Participants blinded |
| Blinding of outcome assessment ok? | Low risk | Objective outcome |
| Incomplete outcome data handled ok? | Low risk | Adequate |
| Free of selective reporting? | Low risk | Recruitment data reported, which is all the review needs |
| Was the study free of other bias? | Low risk | No other biases apparent |
| Overall bias? | Low risk | Low risk of bias |

Jolly 2019 (START MMI PSM-COPD)

|  |  |  |
| --- | --- | --- |
| ***Study characteristics*** | | |
| Methods | 2-arm cluster-randomised study within a trial | |
| Data | Setting: General practice (for recruitment), Hospital (for COPD treatment), UK. Participants were 4,214 adults (18+ years) with COPD and mild dyspnoea. | |
| Comparisons | Investigating multimedia trial information.  Intervention: Multimedia/PPI intervention which provided standard printed materials and an online resource with trial-specific and general information, accessible via URL and QR code.    Comparator: Standard invitation, with no extra multimedia information resource. | |
| Outcomes | Proportion recruited to host trial, proportion retained at 6 and 12 months, intervention costs | |
| ***Risk of bias*** | | |
| **Bias** | **Authors' judgement** | **Support for judgement** |
| Random sequence generation ok? | Low risk | Parallel-arm randomisation by practices (ratio 1:1, with three varying block sizes selected randomly by the computer). |
| Allocation concealment? | Low risk | The allocation sequence was generated centrally by a statistician (who had no other involvement in the running of the host trial) using the ‘ralloc’ command in Stata. |
| Blinding of participants and personnel ok? | Low risk | None of the SWATs blinded participants, although participants did not know they were in a SWAT. The review authors judge that the outcome and the outcome measurement are not likely to be influenced by lack of blinding. |
| Blinding of outcome assessment ok? | Low risk | Most of the outcome assessments were blinded. However, where blinding did not occur, the review authors judge that the outcome and the outcome measurement are not likely to be influenced by lack of blinding. |
| Incomplete outcome data handled ok? | Low risk | They report approaching 4223 participants but only 4214 are accounted for in the flow diagram. However, as this only represents 0.2% of participants, it is unlikely to affect the results, meaning the risk of bias is deemed low. |
| Free of selective reporting? | Low risk | There is no evidence of reporting bias in the study. |
| Was the study free of other bias? | Low risk | No other source of bias has been found in the study. |
| Overall bias? | Low risk | Low risk of bias as described in individual items above |

Kamen 2018

|  |  |  |
| --- | --- | --- |
| ***Study characteristics*** | | |
| Methods | 2-arm individually-randomised study within a trial | |
| Data | Setting: Community-based oncology clinics, USA. Participants were 418 adults (18+ years) with cancer eligible for ongoing phase 2 or 3 oncology trials at the participating site. | |
| Comparisons | Investigating multimedia trial information.  Intervention: Multimedia psychoeducation programme, which included a DVD and booklet aimed to prepare and motivate patients to consider clinical trial participation through educational content and peer examples.   Comparator: Standard print education booklet provided information on cancer clinical trials primarily relating to the nature and conduct of cancer trials (including trial phases and regulatory oversight). | |
| Outcomes | Proportion recruited to host trial | |
| ***Risk of bias*** | | |
| **Bias** | **Authors' judgement** | **Support for judgement** |
| Random sequence generation ok? | Low risk | “Patients were randomly assigned 1:1 by computer generated, random blocks of 4 to 1 of 2 intervention conditions” |
| Allocation concealment? | Low risk | “The study biostatistician maintained the randomization table” |
| Blinding of participants and personnel ok? | Low risk | “The [site study] coordinator stayed with participants in both arms to ensure that they read or watched the study materials and to answer questions.” |
| Blinding of outcome assessment ok? | Low risk | Objective outcome assessment (recruited or not) |
| Incomplete outcome data handled ok? | Low risk | “Because no bias was detected in the pattern of missing data, we applied the Jamshidian and Jalal nonparametric test for data “missing completely at random” and used complete-case analyses for all outcomes” |
| Free of selective reporting? | Low risk | Objective outcomes, all reported as stated. |
| Was the study free of other bias? | Low risk | No other source of bias was identified. |
| Overall bias? | Low risk | Study personnel were not blinded to the arms of the study; however, the site coordinator was not clearly involved in the relevant outcome (study participation in clinical trial) as the other trials were not organised by the authors of the current study. It was felt this would therefore not be a significant source of performance bias, and the overall risk of bias of the study was low. |

Kendrick 2001

|  |  |  |
| --- | --- | --- |
| ***Study characteristics*** | | |
| Methods | Cluster-randomised controlled trial | |
| Data | Setting: primary care, UK. Families with children aged under 5 years, living in deprived areas; 2393 participants | |
| Comparisons | Investigated the effect of different trial information methods  Mailed invitation to participate in an injury prevention trial, including a home safety questionnaire. This was compared to mailed invitation to participate excluding the home safety questionnaire. | |
| Outcomes | Proportion recruited to trial | |
| ***Risk of bias*** | | |
| **Bias** | **Authors' judgement** | **Support for judgement** |
| Random sequence generation ok? | Low risk | Randomised using ACCESS software by neutral researcher |
| Allocation concealment? | Low risk | See above |
| Blinding of participants and personnel ok? | Low risk | Participants blinded, but researchers know (probably). However, because questionnaire was mailed, there was no way researchers could influence result. |
| Blinding of outcome assessment ok? | Low risk | Objective outcome |
| Incomplete outcome data handled ok? | Low risk | Adequate |
| Free of selective reporting? | Low risk | Recruitment outcome presented, which is all the review needs |
| Was the study free of other bias? | Low risk | No other biases apparent |
| Overall bias? | Low risk | Low risk of bias |

Kimmick 2005

|  |  |  |
| --- | --- | --- |
| ***Study characteristics*** | | |
| Methods | Cluster-randomised controlled trial | |
| Data | Setting: secondary care and academic institutions, USA. Practitioners and researchers from 126 Cancer and Leukaemia Group B (CALGB) institutions | |
| Comparisons | Investigated the effect of different trial information methods  Educational intervention of standard information plus an educational symposium, geriatric oncology educational materials, monthly mailings and emails for 1 year, lists of available protocols for use on patient charts, case discussion seminar. This was compared to standard information of periodic notification of all existing CALGB trials by the CALGB Central Office, and CALGB website access. | |
| Outcomes | Proportion recruited to trial | |
| Notes | Clustering was accounted for in the analysis. | |
| ***Risk of bias*** | | |
| **Bias** | **Authors' judgement** | **Support for judgement** |
| Random sequence generation ok? | Unclear risk | Randomisation mentioned but no more details |
| Allocation concealment? | Unclear risk | As above |
| Blinding of participants and personnel ok? | Unclear risk | Not clear what details were given to the participants about the study before it started |
| Blinding of outcome assessment ok? | Low risk | Objective outcome |
| Incomplete outcome data handled ok? | Low risk | Adequate |
| Free of selective reporting? | Low risk | Recruitment outcome presented, which is all the review needs |
| Was the study free of other bias? | Low risk | No other biases apparent |
| Overall bias? | Unclear risk | Unclear risk of bias |

Knapp 2020 (START PIS CASPER)

|  |  |  |
| --- | --- | --- |
| ***Study characteristics*** | | |
| Methods | 2-arm individually-randomised study within a trial | |
| Data | Setting: General practice, England, UK. Participants were 11,531 adults (65+ years) with sub-threshold depression. | |
| Comparisons | Investigating optimised, user-tested PIS.  Intervention: Optimised PIS developed through three rounds of user testing and revision.   Comparator: Standard PIS. | |
| Outcomes | Proportion recruited to host trial, proportion retained. | |
| ***Risk of bias*** | | |
| **Bias** | **Authors' judgement** | **Support for judgement** |
| Random sequence generation ok? | Low risk | Unclear - but not an issue. “Individual patients were allocated randomly (1:1) to receive either the standard or optimised PIS by an independent statistician” No information provided, but due to the nature of intervention it is not anticipated that selection bias would be possible. |
| Allocation concealment? | Low risk | No mention of allocation concealment, but it is not believed that there is a risk of bias. |
| Blinding of participants and personnel ok? | Low risk | No mentioned; due to the nature of the intervention and the SWAT, it would not be possible to introduce performance bias, as outcome was routinely collected data. |
| Blinding of outcome assessment ok? | Low risk | Outcome was routinely collected data, therefore blinding was not required as it was not possible to subvert. Blinding not mentioned - but not possible to introduce bias. |
| Incomplete outcome data handled ok? | Low risk | No evidence that attrition bias occurred; all outcome data reported. |
| Free of selective reporting? | Low risk | No evidence of reporting bias. |
| Was the study free of other bias? | Low risk | No evidence of other sources of bias. |
| Overall bias? | Low risk | No concerns raised about potential sources of bias. |

Knapp 2023a (BALANCE)

|  |  |  |
| --- | --- | --- |
| ***Study characteristics*** | | |
| Methods | 3-arm individually-randomised study within a trial | |
| Data | Setting: Hospitals (expect for BAMP which was in a University setting), UK. Participants were 1,792 children and young people aged 0-18 years being recruited to one of 6 host trials. | |
| Comparisons | Investigating multimedia trial information.  Intervention: Two versions of multimedia intervention(CYP; parents/carers). The MMIs included 5 short animation videos, each lasting 45–60 s. The parents’/carers’ MMI was organised on six main pages: ‘Home page’, ‘About the study’, ‘Taking part’, ‘After the trial’, ‘Questions’, and ‘Contacts’. The MMI for younger CYP had similar contents as the parents/carers. Both MMIs included 16 ‘talking head’ videos: study investigator (13), plus a young adult (1), and parent/carer (2) from a previous similar study (each lasting 20 s to 4 min)  Comparartor: Three versions of participant information leaflet (<6 years, 6–8 years, parent/carers) and ranged from 1 to 9 pages according to age group | |
| Outcomes | Proportion recruited to host trial, proportion retained at 16 weeks. | |
| ***Risk of bias*** | | |
| **Bias** | **Authors' judgement** | **Support for judgement** |
| Random sequence generation ok? | Low risk | *From* NIHR report*:* All SWATs used a random number generator to assign participants to SWAT arms (see. table 7), either at the cluster or individual-level |
| Allocation concealment? | Low risk | Allocation could not have been foreseen as a random number generator was used |
| Blinding of participants and personnel ok? | Low risk | *From* NIHR report*:* ‘Masking of the allocation at outcome measurement was not possible but was also irrelevant: the patient could not be masked to the information format they received but, as they were unaware of the SWAT (the information trial), a lack of masking did not affect their responses on the self-completion measures or have any biasing effect on their decisions on trial participation or continuation.’ |
| Blinding of outcome assessment ok? | Low risk | Participant enrollment is an objective outcome, therefore not a concern. |
| Incomplete outcome data handled ok? | Low risk | Missing data was part of the outcomes of interest (i.e., loss to follow-up), therefore not an issue. |
| Free of selective reporting? | Low risk | No evidence of selective reporting, as all outcomes reported. |
| Was the study free of other bias? | Low risk | No other sources of bias were identified. |
| Overall bias? | Low risk | No concerns raised about potential sources of bias. |

Knapp 2023b (BAMP)

|  |  |  |
| --- | --- | --- |
| ***Study characteristics*** | | |
| Methods | 3-arm individually-randomised study within a trial | |
| Data | Setting: University setting, UK. Participants were 1,792 children and young people aged 0-18 years being recruited to one of 6 host trials. | |
| Comparisons | Investigating multimedia trial information.  Intervention 1: Multimedia information (MMI) alone. This consisted of animations and videos tailored for children and young people.   Intervention 2: Combined PIS & MMI.   Comparator: Participant Information Sheet (PIS) alone. There were two version; one for children and young people, and one for parents/carers. | |
| Outcomes | Proportion recruited to host trial, proportion retained at 18 months | |
| ***Risk of bias*** | | |
| **Bias** | **Authors' judgement** | **Support for judgement** |
| Random sequence generation ok? | Low risk | *From* NIHR report*:* All SWATs used a random number generator to assign participants to SWAT arms (see. table 7), either at the cluster or individual-level |
| Allocation concealment? | Low risk | Allocation could not have been foreseen as a random number generator was used |
| Blinding of participants and personnel ok? | Low risk | *From* NIHR report*:* ‘Masking of the allocation at outcome measurement was not possible but was also irrelevant: the patient could not be masked to the information format they received but, as they were unaware of the SWAT (the information trial), a lack of masking did not affect their responses on the self-completion measures or have any biasing effect on their decisions on trial participation or continuation.’ |
| Blinding of outcome assessment ok? | Low risk | Participant enrollment is an objective outcome |
| Incomplete outcome data handled ok? | Low risk | Missing data was part of the outcomes of interest (i.e., loss to follow-up), therefore not an issue. |
| Free of selective reporting? | Low risk | No evidence of selective reporting, as all outcomes reported. |
| Was the study free of other bias? | Low risk | No other sources of bias were identified. |
| Overall bias? | Low risk | No concerns raised about potential sources of bias. |

Knapp 2023c (CHAMP-UK)

|  |  |  |
| --- | --- | --- |
| ***Study characteristics*** | | |
| Methods | 3-arm individually-randomised study within a trial | |
| Data | Setting: Hospitals (expect for BAMP which was in a University setting), UK. Participants were 1,792 children and young people aged 0-18 years being recruited to one of 6 host trials. | |
| Comparisons | Investigating multimedia trial information.  Intervention 1: Multimedia information (MMI) alone emailed prior to screening appointment. This consisted of animations and videos tailored for children and young people, with a separate version for parents and carers.   Intervention 2: Combined PIS & MMI.   Comparator: Participant Information Sheet (PIS) alone emailed prior to screening appointment. This standard PIS was customised for different age groups, with a version for parents/carers. | |
| Outcomes | Proportion recruited to host trial, proportion retained at 6 months. | |
| ***Risk of bias*** | | |
| **Bias** | **Authors' judgement** | **Support for judgement** |
| Random sequence generation ok? | Low risk | *From* NIHR report*:* All SWATs used a random number generator to assign participants to SWAT arms (see. table 7), either at the cluster or individual-level |
| Allocation concealment? | Low risk | Allocation could not have been foreseen as a random number generator was used |
| Blinding of participants and personnel ok? | Low risk | *From* NIHR report*:* ‘Masking of the allocation at outcome measurement was not possible but was also irrelevant: the patient could not be masked to the information format they received but, as they were unaware of the SWAT (the information trial), a lack of masking did not affect their responses on the self-completion measures or have any biasing effect on their decisions on trial participation or continuation.’ |
| Blinding of outcome assessment ok? | Low risk | Participant enrollment is an objective outcome |
| Incomplete outcome data handled ok? | Low risk | Missing data was part of the outcomes of interest (i.e., loss to follow-up), therefore not an issue. |
| Free of selective reporting? | Low risk | No evidence of selective reporting, as all outcomes reported. |
| Was the study free of other bias? | Low risk | No other sources of bias were identified. |
| Overall bias? | Low risk | No concerns raised about potential sources of bias. |

Knapp 2023d (FORCE)

|  |  |  |
| --- | --- | --- |
| ***Study characteristics*** | | |
| Methods | 2-arm cluster-randomised study within a trial | |
| Data | Setting: Hospital, UK. Participants were 1,409 children aged 4-15 years with a wrist fracture | |
| Comparisons | Investigating multimedia trial information.  Intervention: Multimedia intervention (MMI). This consisted of a webpage with five short video animations and 12 short ‘talking head’ video clips viewed on a tablet computer at the hospital. There was one version for children and young people and one for parents/carers.   Comparator: Standard Printed Participant Information Sheet (PIS) used in the trial. There were 3 versions tailored for different age groups, covering trial details and procedures to support understanding. | |
| Outcomes | Proportion recruited to host trial, proportion retained at 6 weeks | |
| ***Risk of bias*** | | |
| **Bias** | **Authors' judgement** | **Support for judgement** |
| Random sequence generation ok? | Low risk | The 23 hospital sites were allocated at the University of York, using a random number generator (Sealed envelope, 2019. Available: https://www.sealedenvelope.com/) |
| Allocation concealment? | Low risk | Allocations were sent to sites by email via the Clinical Trial Unit running the FORCE trial. |
| Blinding of participants and personnel ok? | Low risk | The recruitment centres or participants could not be masked to allocation due to the nature of the intervention.   Review authors judge that the outcome and the outcome measurement are not likely to be influenced by lack of blinding; |
| Blinding of outcome assessment ok? | Low risk | Participants were not aware that they were being randomised within the TRECA SWAT, as approved by the National Health Service (NHS) Research Ethics Committee, and they were not aware that participants in other hospitals were being given a different format of information.  Review authors judge that the outcome and the outcome measurement are not likely to be influenced by lack of blinding; |
| Incomplete outcome data handled ok? | Low risk | Clearly labelled in results section where data were missing. Up to three missing values were allowed, with the total score calculated by replacing the missing values with the mean score from the completed responses. |
| Free of selective reporting? | Low risk | All outcomes that were pre-specified, including recruitment rates and retention are reported |
| Was the study free of other bias? | Low risk | No other bias was identified. The SWAT was embedded within routine trial processes using standard procedures. There were no reports of baseline imbalances, contamination, or protocol deviations likely to introduce bias beyond the domains already assessed |
| Overall bias? | Low risk | Randomisation was conducted centrally using a validated system, and although blinding of personnel was not possible, the risk of performance bias was mitigated by the fact that participants were unaware of their involvement in the SWAT. Outcome assessment was objective, and the trial demonstrated high transparency in reporting both missing data and pre-specified outcomes |

Knapp 2023e (Thermic-3)

|  |  |  |
| --- | --- | --- |
| ***Study characteristics*** | | |
| Methods | 3-arm individually-randomised study within a trial | |
| Data | Setting: Hospital, England, UK. Participants were 153 children (0-17 years) awaiting surgery for congenital heart defects. | |
| Comparisons | Investigating multimedia trial information.  Intervention 1: Multimedia information (MMI) alone. This consisted of animations and videos tailored for different age groups, covering trial details and procedures to support understanding.   Intervention 2: Combined PIS & MMI.   Comparator: Participant Information Sheet (PIS) alone. This standard PIS customised for different age groups, provided essential trial details reviewed by ethics and advisory bodies | |
| Outcomes | Proportion recruited to host trial, proportion retained at 3 months. | |
| ***Risk of bias*** | | |
| **Bias** | **Authors' judgement** | **Support for judgement** |
| Random sequence generation ok? | Low risk | “The study used the computer generated randomisation service provided by sealed envelope” |
| Allocation concealment? | Low risk | Allocation was randomised. |
| Blinding of participants and personnel ok? | Low risk | “Recruiting nurses were not masked to allocation, although there was concealment of allocation. Participants were not aware of the information SWAT (and so did not consent to it), as approved by the Research Ethics Committee, so their masking was maintained.” |
| Blinding of outcome assessment ok? | Low risk | Outcome of interest was subjective. |
| Incomplete outcome data handled ok? | Low risk | “For the primary outcome analysis recruitment rates were compared between the PIS-only and MMI-only arms using logistic regression following modified intention-to-treat principles, where all patients eligible for TRECA were included in the group they were randomised to and excluding participants who were not eligible to join Thermic-3, to assess the effect of replacing printed information with multimedia.” |
| Free of selective reporting? | Low risk | All expected outcomes were reported. |
| Was the study free of other bias? | Low risk | No other source of bias was identified. |
| Overall bias? | Low risk | No concerns about potential sources of bias identified. |

Knapp 2023f (UKALL)

|  |  |  |
| --- | --- | --- |
| ***Study characteristics*** | | |
| Methods | 3-arm individually-randomised study within a trial | |
| Data | Setting: Hospitals (expect for BAMP which was in a University setting), UK. Participants were 1,792 children and young people aged 0-18 years being recruited to one of 6 host trials. | |
| Comparisons | Investigating multimedia trial information.  Intervention 1: Multimedia information (MMI) alone. This consisted of different webpage versions containing animations and videos tailored for different age groups and parents/carers.   Intervention 2: Combined PIS & MMI.   Comparator: Participant Information Sheet (PIS) alone. This standard PIS customised for different age groups, as well as parents/carers. | |
| Outcomes | Proportion recruited to host trial, proportion retained at 3 months. | |
| ***Risk of bias*** | | |
| **Bias** | **Authors' judgement** | **Support for judgement** |
| Random sequence generation ok? | Low risk | *From* NIHR report*: ‘*All SWATs used a random number generator to assign participants to SWAT arms (see. table 7), either at the cluster or individual-level.’ |
| Allocation concealment? | Low risk | Allocation could not have been foreseen as a random number generator was used |
| Blinding of participants and personnel ok? | Low risk | *From* NIHR report*:* ‘Masking of the allocation at outcome measurement was not possible but was also irrelevant: the patient could not be masked to the information format they received but, as they were unaware of the SWAT (the information trial), a lack of masking did not affect their responses on the self-completion measures or have any biasing effect on their decisions on trial participation or continuation.’ |
| Blinding of outcome assessment ok? | Low risk | Participant enrollment is an objective outcome |
| Incomplete outcome data handled ok? | Low risk | Missing data was part of the outcomes of interest (i.e., loss to follow-up), therefore not an issue. |
| Free of selective reporting? | Low risk | No evidence of selective reporting, as all outcomes reported. |
| Was the study free of other bias? | Low risk | No other sources of bias were identified. |
| Overall bias? | Low risk | No concerns raised about potential sources of bias. |

Larkey 2002

|  |  |  |
| --- | --- | --- |
| ***Study characteristics*** | | |
| Methods | Cluster-randomised controlled trial | |
| Data | Setting: various existing trial sites, USA. 96 participants in the Women's Health Initiative trial | |
| Comparisons | Investigated the effect of different methods of training lay advocates for trials  Intervention A: Hispanic lay advocates; attended 6 hour-long training sessions, 5 quarterly meetings and received brochures with interest cards to distribute to other women    Intervention B: Hispanic women controls, received quarterly telephone calls and brochures with interest cards to distribute to other women    Compared to Anglo women controls, received quarterly telephone calls and brochures with interest cards to distribute to other women | |
| Outcomes | Proportion recruited to trial | |
| ***Risk of bias*** | | |
| **Bias** | **Authors' judgement** | **Support for judgement** |
| Random sequence generation ok? | Unclear risk | Randomisation mentioned but no more details |
| Allocation concealment? | Unclear risk | See above |
| Blinding of participants and personnel ok? | Unclear risk | Not clear if the participants were blinded |
| Blinding of outcome assessment ok? | Low risk | Objective outcome |
| Incomplete outcome data handled ok? | Low risk | Adequate |
| Free of selective reporting? | Low risk | Recruitment outcome presented, which is all the review needs |
| Was the study free of other bias? | Low risk | No other biases apparent |
| Overall bias? | Unclear risk | Unclear risk of bias |

Lee 2017

|  |  |  |
| --- | --- | --- |
| ***Study characteristics*** | | |
| Methods | Cluster-randomised controlled trial | |
| Data | Setting: primary care, Australia. 744 primary care clinics (372 general practice and 372 physiotherapy clinics) in the Sydney metropolitan area. Recruiting clinics for a trial of an intervention to reduce low back pain | |
| Comparisons | Investigated the use of a teaser campaign to increase recruitment of clinical centres  Mailed 3 postcards out as a part of a staged teaser campaign to raise awareness of trial prior to invitation letter. This was compared to no teaser postcards. | |
| Outcomes | Proportion of clinics recruited | |
| ***Risk of bias*** | | |
| **Bias** | **Authors' judgement** | **Support for judgement** |
| Random sequence generation ok? | Low risk | An investigator not involved in outcome assessment generated a 1:1 randomisation schedule using a random number generator and assigned clinics to the groups. |
| Allocation concealment? | Low risk | See above |
| Blinding of participants and personnel ok? | Low risk | The clinicians and support staff were blind to the different recruitment strategies that were being tested in this study. |
| Blinding of outcome assessment ok? | Low risk | Objective outcome |
| Incomplete outcome data handled ok? | Low risk | Adequate |
| Free of selective reporting? | Low risk | Recruitment outcome available, which is all the review needs |
| Was the study free of other bias? | Low risk | No other biases apparent |
| Overall bias? | Low risk | Low risk of bias |

Liénard 2006

|  |  |  |
| --- | --- | --- |
| ***Study characteristics*** | | |
| Methods | Cluster-randomised controlled trial | |
| Data | Setting: secondary care, France. Centres recruiting to a randomised controlled trial for breast cancer; 573 participants | |
| Comparisons | Investigated the effect of organising visits by the trial co-ordination team to centres participating in a multicentre trial  Site visits including an initiation visit to review trial protocol, inclusion/exclusion criteria, safety, randomisation etc. plus ongoing review visits. This was compared to no site visits (unless requested). | |
| Outcomes | Proportion recruited to trial | |
| Notes | Clustering was not accounted for in the analysis. | |
| ***Risk of bias*** | | |
| **Bias** | **Authors' judgement** | **Support for judgement** |
| Random sequence generation ok? | Low risk | Minimisation |
| Allocation concealment? | Low risk | Done centrally by the coordinating office |
| Blinding of participants and personnel ok? | Low risk | Centres blind. Somewhat unclear if monitors were blind but probably were not |
| Blinding of outcome assessment ok? | Low risk | Partial (see above) but objective outcome |
| Incomplete outcome data handled ok? | Low risk | Adequate |
| Free of selective reporting? | Low risk | Recruitment outcome presented, which is all the review needs |
| Was the study free of other bias? | Low risk | No other biases apparent |
| Overall bias? | Low risk | Low risk of bias |

Lipstein 2021

|  |  |  |
| --- | --- | --- |
| ***Study characteristics*** | | |
| Methods | 2-arm cluster-randomised study within a trial | |
| Data | Setting: Hospital, USA. Participants were 265 individuals from 43 sites under age 21 with pediatric Crohn’s disease starting anti-TNF therapy. | |
| Comparisons | Investigating enhanced pre-consent discussion with aid of poster.  Intervention: Enhanced pre-consent discussion using a handheld poster.  The poster had English and Spanish versions, and guided clinicians and families through trial information on equipoise, logistics, and decision readiness, with compact versions available for families to take home.   Comparator: Standard consent materials, which included an FAQ handout. | |
| Outcomes | Proportion recruited to host trial | |
| ***Risk of bias*** | | |
| **Bias** | **Authors' judgement** | **Support for judgement** |
| Random sequence generation ok? | Low risk | "One of the authors (N.O.) generated the random allocation sequence using covariate-constrained randomization [15] based on the number of patients each site expected to recruit for COMBINE” |
| Allocation concealment? | Low risk | No concerns, as site randomisation was done centrally: “We informed sites of their allocation at the beginning of the study.” |
| Blinding of participants and personnel ok? | Low risk | “We concealed site allocation from individual participants, until after assessing the outcome for this trial (see Procedures section below for details).” |
| Blinding of outcome assessment ok? | Low risk | Yes (for enrollment outcome), as enrollment rate is not subjective, and participants were unaware of the SWAT until after this had been collected.   NOTE:Unclear for primary and secondary outcomes.  “Participants then completed the 10 question survey. We gave research coordinators a script to provide the correct answer to any incorrectly answered knowledge question and address any decisional uncertainty, prior to the participant signing the COMBINE written consent form.”  Unclear if participants may have had their answers influenced by the research coordinators, although it is reassuring that they were given a script. Primary and secondary outcomes were also self-reported by participants. |
| Incomplete outcome data handled ok? | Low risk | There is missing outcome data- 15 in intervention, 11 in control. No explanation for why it is missing. Note- this does not affect the outcome of patients enrolled. Similar numbers between groups. |
| Free of selective reporting? | Unclear risk | Unclear how the proportion of participants enrolled was calculated. Authors state: “We used the screening and enrollment log from each site to ascertain the number of eligible patients and the number enrolled,” suggesting that the number of eligible participants was the denominator (as opposed to the number of participants approached). Nevertheless, details of the numbers of participants approached in each arm and enrolled in the host trial are not given. |
| Was the study free of other bias? | High risk | “11 intervention subjects reported not having seen the intervention. 12 control participants reported exposure to the intervention. Indeed, early in the study, 8 control sites were inappropriately given access to the intervention and one used it, without training, with participants. We withdrew this access, thereby preventing further contamination of trial arms. A “perprotocol” analysis did not change our findings.” |
| Overall bias? | High risk | Downgraded due to concerns about contamination bias. |

Litchfield 2005

|  |  |  |
| --- | --- | --- |
| ***Study characteristics*** | | |
| Methods | Cluster-randomised controlled trial | |
| Data | Setting: primary care, UK. Participants were general practices participating in a trial of 2 delivery systems for insulin, NovoPen and Innovo. 28 practices were involved and 73 participants recruited | |
| Comparisons | Intervention: electronic data capture  Comparator: paper data capture | |
| Outcomes | Number of participants recruited to the trial. Improving recruitment was not the main aim (improving efficiency was the main aim) of the study though this information is provided. | |
| Notes | Clustering was not accounted for in analysis. | |
| ***Risk of bias*** | | |
| **Bias** | **Authors' judgement** | **Support for judgement** |
| Random sequence generation ok? | Low risk | Computer-generated randomisation code in compliance with FDA and EU regulations |
| Allocation concealment? | Low risk | Done centrally (inferred rather than explicit but seems reasonable to assume for this cluster trial) |
| Blinding of participants and personnel ok? | Unclear risk | Investigators knew that both paper and electronic data collection were to be used so study was not blinded. Unlikely that patient decisions to join study would be affected by this. Not clear how much influence knowledge of data collection method might have had on practices. |
| Blinding of outcome assessment ok? | Low risk | Objective outcome. Improving recruitment was not the main aim (improving efficiency was the main aim) of the study, though this information is provided |
| Incomplete outcome data handled ok? | Low risk | Adequate |
| Free of selective reporting? | Low risk | Recruitment outcome presented, which is all the review needs |
| Was the study free of other bias? | Low risk | No other biases apparent |
| Overall bias? | Unclear risk | Unclear risk of bias |

Madurasinghe 2021 (START PIS HeLP Diabetes)

|  |  |  |
| --- | --- | --- |
| ***Study characteristics*** | | |
| Methods | 2-arm individually-randomised study within a trial | |
| Data | Setting: General practices, England, UK. Participants were 4,880 adults (18+ years) with type 2 diabetes. | |
| Comparisons | Investigating optimised, user-tested PIS.  Intervention: Optimised participant information sheet optimised through user-testing.   Comparator: Standard participant information sheet. | |
| Outcomes | Proportion recruited to host trial | |
| ***Risk of bias*** | | |
| **Bias** | **Authors' judgement** | **Support for judgement** |
| Random sequence generation ok? | Low risk | Computer-generated randomised sequence. The statistician involved in the HeLP-Diabetes trial. Randomisation was conducted in a 1:1 ratio without stratification, at the general practice level |
| Allocation concealment? | Low risk | Computer-generated randomised sequence.  Both practices and patients were blinded to their allocation |
| Blinding of participants and personnel ok? | Low risk | Both practices and patients were blinded to their allocation |
| Blinding of outcome assessment ok? | Low risk | Both practices and patients were blinded to their allocation |
| Incomplete outcome data handled ok? | Low risk | There does not appear to be any missing data. |
| Free of selective reporting? | Low risk | Expected outcomes reported. |
| Was the study free of other bias? | Low risk | No other bias was identified. |
| Overall bias? | Low risk | All criteria were marked as “yes,” so overall this indicates a low risk of bias. |

Madurasinghe 2023a (START MMI GHT2000a)

|  |  |  |
| --- | --- | --- |
| ***Study characteristics*** | | |
| Methods | 2-arm individually-randomised coordinated study within a trial (across 5 host trials) | |
| Data | Settings: Various (mix of general practice, hospital, and community, UK. Participants were 11,381 (mostly adults, some children) being recruited to various host trials with different conditions. | |
| Comparisons | Investigating multimedia trial information.  Intervention: Multimedia intervention (MMI), developed with patient and public involvement partners, which included study-specific and generic information, with access provided via a URL link and QR code in the patient information sheet.   Comparator: Standard participant information sheet (PIS). | |
| Outcomes | Proportion recruited to host trial | |
| ***Risk of bias*** | | |
| **Bias** | **Authors' judgement** | **Support for judgement** |
| Random sequence generation ok? | Low risk | Random allocation using a method which is robust and ensures concealment of allocation, such as a computerised randomisation programme. |
| Allocation concealment? | Low risk | The type of intervention received will be determined by random allocation using a method which is robust and ensures concealment of allocation. |
| Blinding of participants and personnel ok? | Low risk | None of the SWATs blinded participants, although participants did not know they were in a SWAT. The review authors judge that the outcome and the outcome measurement are not likely to be influenced by lack of blinding. |
| Blinding of outcome assessment ok? | Low risk | Most of the outcome assessments were blinded. However, where blinding did not occur, the review authors judge that the outcome and the outcome measurement are not likely to be influenced by lack of blinding. |
| Incomplete outcome data handled ok? | Low risk | One host trial (STOP ACEi) was only able to report accurate data on 11/37 sites randomised and was excluded from all the analyses (available data by arm are reported in Additional file 3).  “One study was unable to provide the necessary data for our main analysis, which reduced the sample size available. In our experience, problems with delivering SWATs are fairly rare but these difficulties do highlight that SWATs can stretch the resources of already busy trial teams.” |
| Free of selective reporting? | Low risk | No selective reporting. |
| Was the study free of other bias? | Low risk | No other sources of bias identified. |
| Overall bias? | Low risk | Low risk of bias, as no concerns about potential sources of bias identified. |

Madurasinghe 2023b (START MMI GHT2000b)

|  |  |  |
| --- | --- | --- |
| ***Study characteristics*** | | |
| Methods | 2-arm individually-randomised coordinated study within a trial (across 5 host trials) | |
| Data | Settings: Various (mix of general practice, hospital, and community, UK. Participants were 11,381 (mostly adults, some children) being recruited to various host trials with different conditions. | |
| Comparisons | Investigating multimedia trial information.  Intervention: Multimedia intervention (MMI), developed with patient and public involvement partners, which included study-specific and generic information, with access provided via a URL link and QR code in the patient information sheet.   Comparator: Standard participant information sheet (PIS). | |
| Outcomes | Proportion recruited to host trial | |
| ***Risk of bias*** | | |
| **Bias** | **Authors' judgement** | **Support for judgement** |
| Random sequence generation ok? | Low risk | Random allocation using a method which is robust and ensures concealment of allocation, such as a computerised randomisation programme. |
| Allocation concealment? | Low risk | The type of intervention received will be determined by random allocation using a method which is robust and ensures concealment of allocation. |
| Blinding of participants and personnel ok? | Low risk | None of the SWATs blinded participants, although participants did not know they were in a SWAT. The review authors judge that the outcome and the outcome measurement are not likely to be influenced by lack of blinding. |
| Blinding of outcome assessment ok? | Low risk | Most of the outcome assessments were blinded. However, where blinding did not occur, the review authors judge that the outcome and the outcome measurement are not likely to be influenced by lack of blinding. |
| Incomplete outcome data handled ok? | Low risk | One host trial (STOP ACEi) was only able to report accurate data on 11/37 sites randomised and was excluded from all the analyses (available data by arm are reported in Additional file 3).  “One study was unable to provide the necessary data for our main analysis, which reduced the sample size available. In our experience, problems with delivering SWATs are fairly rare but these difficulties do highlight that SWATs can stretch the resources of already busy trial teams.” |
| Free of selective reporting? | Low risk | No selective reporting. |
| Was the study free of other bias? | Low risk | No other sources of bias identified. |
| Overall bias? | Low risk | Low risk of bias, as no concerns about potential sources of bias identified. |

Madurasinghe 2023c (START MMI HI-Light)

|  |  |  |
| --- | --- | --- |
| ***Study characteristics*** | | |
| Methods | 2-arm individually-randomised coordinated study within a trial (across 5 host trials) | |
| Data | Settings: Various (mix of general practice, hospital, and community, UK. Participants were 11,381 (mostly adults, some children) being recruited to various host trials with different conditions. | |
| Comparisons | Investigating multimedia trial information.  Intervention: Multimedia intervention (MMI), developed with patient and public involvement partners, which included study-specific and generic information, with access provided via a URL link and QR code in the patient information sheet.   Comparator: Standard participant information sheet (PIS). | |
| Outcomes | Proportion recruited to host trial, proportion retained at 9 months. | |
| ***Risk of bias*** | | |
| **Bias** | **Authors' judgement** | **Support for judgement** |
| Random sequence generation ok? | Low risk | Allocation of practices to the embedded trial will be minimised by recruitment centre and practice size retaining a random element, and implemented using a secure, online randomisation system created and maintained by Nottingham Clinical Trials Unit, thereby ensuring allocation concealment. |
| Allocation concealment? | Low risk | Allocation of practices to the embedded trial will be minimised by recruitment centre and practice size retaining a random element, and implemented using a secure, online randomisation system created and maintained by Nottingham Clinical Trials Unit, thereby ensuring allocation concealment. |
| Blinding of participants and personnel ok? | Low risk | None of the SWATs blinded participants, although participants did not know they were in a SWAT. The review authors judge that the outcome and the outcome measurement are not likely to be influenced by lack of blinding. |
| Blinding of outcome assessment ok? | Low risk | The online randomisation system was created and maintained by Nottingham Clinical Trials Unit, thereby ensuring allocation concealment. |
| Incomplete outcome data handled ok? | Low risk | One host trial (STOP ACEi) was only able to report accurate data on 11/37 sites randomised and was excluded from all the analyses (available data by arm are reported in Additional file 3).  “One study was unable to provide the necessary data for our main analysis, which reduced the sample size available. In our experience, problems with delivering SWATs are fairly rare but these difficulties do highlight that SWATs can stretch the resources of already busy trial teams.” |
| Free of selective reporting? | Low risk | No selective reporting. |
| Was the study free of other bias? | Low risk | No other sources of bias identified. |
| Overall bias? | Low risk | Low risk of bias, as no concerns about potential sources of bias identified. |

Madurasinghe 2023d (START MMI seAFOod)

|  |  |  |
| --- | --- | --- |
| ***Study characteristics*** | | |
| Methods | 2-arm cluster-randomised coordinated study within a trial (across 5 host trials) | |
| Data | Settings: Various (mix of general practice, hospital, and community, UK. Participants were 11,381 (mostly adults, some children) being recruited to various host trials with different conditions. | |
| Comparisons | Investigating multimedia trial information.  Intervention: Multimedia intervention (MMI), developed with patient and public involvement partners, which included study-specific and generic information, with access provided via a URL link and QR code in the patient information sheet.   Comparator: Standard participant information sheet (PIS). | |
| Outcomes | Proportion recruited to host trial, proportion retained at 12 months. | |
| ***Risk of bias*** | | |
| **Bias** | **Authors' judgement** | **Support for judgement** |
| Random sequence generation ok? | Low risk | The randomisation procedure will use a computer-generated random number sequence, and be conducted by a statistician at the University of Nottingham Clinical Trials Unit who has no other role in the trial. |
| Allocation concealment? | Low risk | The randomisation procedure will use a computer-generated random number sequence, and be conducted by a statistician at the University of Nottingham Clinical Trials Unit who has no other role in the trial. |
| Blinding of participants and personnel ok? | Low risk | None of the SWATs blinded participants, although participants did not know they were in a SWAT. The review authors judge that the outcome and the outcome measurement are not likely to be influenced by lack of blinding. |
| Blinding of outcome assessment ok? | Low risk | Most of the outcome assessments were blinded. However, where blinding did not occur, the review authors judge that the outcome and the outcome measurement are not likely to be influenced by lack of blinding. |
| Incomplete outcome data handled ok? | Low risk | One host trial (STOP ACEi) was only able to report accurate data on 11/37 sites randomised and was excluded from all the analyses (available data by arm are reported in Additional file 3).  “One study was unable to provide the necessary data for our main analysis, which reduced the sample size available. In our experience, problems with delivering SWATs are fairly rare but these difficulties do highlight that SWATs can stretch the resources of already busy trial teams.” |
| Free of selective reporting? | Low risk | No selective reporting. |
| Was the study free of other bias? | Low risk | No other sources of bias identified. |
| Overall bias? | Low risk | Low risk of bias, as no concerns about potential sources of bias identified. |

Man 2015 (START PIS Healthlines Depression)

|  |  |  |
| --- | --- | --- |
| ***Study characteristics*** | | |
| Methods | Randomised controlled trial | |
| Data | Setting: primary care, UK. 1364 participants who were identified as potentially eligible for the Healthlines CVD study | |
| Comparisons | Investigated an alternative way of presenting patient information materials  Intervention: participant information that developed in collaboration with patients together with a graphic designer  Comparator: standard participant information materials | |
| Outcomes | Proportion recruited to trial | |
| ***Risk of bias*** | | |
| **Bias** | **Authors' judgement** | **Support for judgement** |
| Random sequence generation ok? | Low risk | Computer-generated random numbers to split those to be invited |
| Allocation concealment? | Low risk | Use of IDs, sorted by random number |
| Blinding of participants and personnel ok? | Low risk | Patients unaware of recruitment study. Researchers blind to patient allocation |
| Blinding of outcome assessment ok? | Low risk | Objective outcomes |
| Incomplete outcome data handled ok? | Low risk | Adequate |
| Free of selective reporting? | Low risk | Recruitment reported and this is only outcome needed for review |
| Was the study free of other bias? | Low risk | No other biases apparent |
| Overall bias? | Low risk | Low risk of bias |

Mattock 2020

|  |  |  |
| --- | --- | --- |
| ***Study characteristics*** | | |
| Methods | 2-arm individually-randomised study within a trial | |
| Data | Setting: Unspecified, UK. Participants were 107 parents aged ≥18 years with children aged 12-36 months being recruited to a parenting trial. | |
| Comparisons | Investigating providing information by video.  Intervention: Video + PIS. The video was a 5-minute video emailed to participants, featuring a PPI group member and family explaining research visit details, a welcome from the chief investigator, trial arm overviews, and introductions to the research assistants conducting home visits.   Comparator: PIS only. | |
| Outcomes | Proportion recruited to host trial | |
| ***Risk of bias*** | | |
| **Bias** | **Authors' judgement** | **Support for judgement** |
| Random sequence generation ok? | High risk | Used quasi-randomisation, as participants were randomised according to the week they returned their screening questionnaire: “A randomisation list for the initial contact was prepared by the trial statistician, and allocation was released weekly to the research team. Each participant was allocated to their condition based on the week they returned their screening questionnaire.” |
| Allocation concealment? | High risk | Allocation could have been foreseen as participants were randomised according to the week they returned their screening questionnaire: “Each participant was allocated to their condition based on the week they returned their screening questionnaire.” |
| Blinding of participants and personnel ok? | Unclear risk | “The research team were not blinded to group allocation.”  Insufficient information to know if participants were blinded. |
| Blinding of outcome assessment ok? | Low risk | Outcomes of interest for this review (odds of recruitment) were objective and therefore unlikely to have been affected. |
| Incomplete outcome data handled ok? | Low risk | No missing outcome data for proportion recruited. |
| Free of selective reporting? | Low risk | Outcome of interest (proportion consenting to trial) reported in full, including those that received the incorrect initial contact. |
| Was the study free of other bias? | High risk | 6 of the 56 individuals assigned to the video clip condition received the incorrect initial contact, compared to none in the PIS condition. These participants were however excluded from formal analyses (as such the authors did not perform an intention to treat analysis). Additionally, "Despite randomisation, more participants in the PIS group (63%) were screened in person in comparison to other methods whereas only 40% of participants in the video clip condition were screened in person." |
| Overall bias? | High risk | Trial was quasi-ranodmised, as participants were randomised according to the week they returned their screening questionnaire meaning there was a high risk of bias. Other concerns were raised regarding some participants not receiving the type of initial contact they were randomised to, and more participants in the PIS group being screened in person than those in the video clip condition. |

Maxwell 2017

|  |  |  |
| --- | --- | --- |
| ***Study characteristics*** | | |
| Methods | Stepped-wedge cluster randomised study within a trial | |
| Data | Setting: Hospitals, UK. Participants were patients (aged 18+) with intracerebral haemorrhage following a stroke, who were uncertain about restaring antiplatelet drugs. | |
| Comparisons | Investigating a recruitment co-ordinator intervention.  Intervention: a recruitment coordinator conducting teleconferences with site teams to enhance host trial recruitment using software for identifying eligible patients, with a second teleconference after 6 months.   Comparator: Control condition (before sites receive recruitment review). | |
| Outcomes | Number of patients randomised into host trial per month per site | |
| ***Risk of bias*** | | |
| **Bias** | **Authors' judgement** | **Support for judgement** |
| Random sequence generation ok? | Low risk | All sites began in the control state and were allocated using block randomisation stratified by hospital location (Scotland versus England/Wales) to start the complex intervention in one of 12 different months.   ‘stratified block randomisation to randomly allocate the 72 sites into 12 groups of six sites each, with stratification by hospital location’ |
| Allocation concealment? | Low risk | We used stratified block randomisation…A programmer independent of the trial created the computer-generated random allocation sequence and sent this list to the trial data manager who then informed the recruitment co-ordinator at the required times for them to arrange the review. |
| Blinding of participants and personnel ok? | Low risk | From published statistical analysis plan (https://link.springer.com/article/10.1186/s13063-017-1840-8) :  The investigators, recruitment coordinator, and sites were all blinded to the timing of the recruitment review until this had to be revealed in order to organise the recruitment review.   No blinding, but the judged that the outcome and the outcome measurement are not likely to be influenced by lack of blinding. |
| Blinding of outcome assessment ok? | Low risk | Outcome assessment was not blinded, however, this was unlikely to have biased the outcome assessment |
| Incomplete outcome data handled ok? | Low risk | The primary analysis used intention-to-treat principle. In addition, for the primary analysis, all 72 sites were included regardless of any site withdrawals or site suspensions or compliance with the PRIME trial procedures. For sites that were suspended (i.e. not permitted to recruit patients due to the principal investigator leaving or another reason), their missing observations were imputed as ‘0’ patients randomised. This was believed to be an appropriate imputation since it is likely, based on the reasons for suspension (e.g. due to the principal investigator being off sick), that many of these sites who were suspended would have failed to randomise any patients if they remained in the trial, and also ‘0’ was by far the most frequently observed number of patients randomised per month. However, to check the robustness of this assumption and the impact of any deviations from the protocol, a per-protocol secondary analysis was performed after excluding any sites experiencing permanent suspensions or sites failing to receive either the initial recruitment review or the 6-month follow-up review. |
| Free of selective reporting? | Low risk | Reported all outcomes |
| Was the study free of other bias? | Low risk | No other biases identified |
| Overall bias? | Low risk | This study was assessed as low risk of bias across all domains. |

McCaffery 2019

|  |  |  |
| --- | --- | --- |
| ***Study characteristics*** | | |
| Methods | 2-arm individually-randomised study within a trial | |
| Data | Setting: General Practice, UK. 317 Participants were aged 65 years or over, living in the community, and at high risk of falls and were willing to receive a home visit from an occupational therapist. | |
| Comparisons | Investigating a handwritten personalised invitation letter.  Intervention: Handwritten personalised invitation letter, comprised as a handwritten personalised invitation letter with their name in the salutation, included in the standard recruitment pack.   Comparator: Standard invitation letter. | |
| Outcomes | Proportion recruited to host trial, proportion retained at 3 months | |
| ***Risk of bias*** | | |
| **Bias** | **Authors' judgement** | **Support for judgement** |
| Random sequence generation ok? | Low risk | Generation of the allocation sequence was undertaken by the OTIS trial statistician, who was not involved with the production of the recruitment packs, using Stata version 13. |
| Allocation concealment? | Low risk | Block randomisation was used to allocate the recruitment packs in a 1:1 ratio to either the control group or the intervention group, using one large block the size of the mail out (n=317). |
| Blinding of participants and personnel ok? | Unclear risk | Participants see the letter they receive, so they cannot be blinded.The paper does not report whether staff were blinded. |
| Blinding of outcome assessment ok? | Unclear risk | Not enough information to determine if detection bias was possible e.g whether the person recording outcomes was blinded or whether the process was automated. |
| Incomplete outcome data handled ok? | Low risk | Doesn’t look like there were any missing data. |
| Free of selective reporting? | Low risk | Expected outcomes reported. |
| Was the study free of other bias? | Low risk | No other bias was identified. |
| Overall bias? | Unclear risk | Overall marked as unclear as 2 criteria were ticked’ unclear. |

Miller 2021a

|  |  |  |
| --- | --- | --- |
| ***Study characteristics*** | | |
| Methods | 3-arm individually-randomised study within a trial | |
| Data | Setting: Community-based research clinics, USA. Participants were 2605 participants accessing the study website during the substudy period. The host trial was recruiting patients (aged 70+) at elevated risk of falling with low serum Vitamin D. | |
| Comparisons | Investigating different wording of risk of underlying condition.  Intervention: Version A infographic on the website landing page stating that “Every year 1 in 3 adults over the age of 65 fall”.   Comparator: Original landing page | |
| Outcomes | Proportion recruited to host trial | |
| ***Risk of bias*** | | |
| **Bias** | **Authors' judgement** | **Support for judgement** |
| Random sequence generation ok? | Low risk | “*In this experiment, web-users were randomized to one of four landing pages, one of which was the original landing page and three of which included a video.*” Participants were randomised using Optimizely web portal.  From Fig. 1: “*Digital randomization completed by A/B testing platform.*”  Randomised using Optimizely web portal. |
| Allocation concealment? | Low risk | Could not have been foreseen, as was done via a web portal. |
| Blinding of participants and personnel ok? | Low risk | Done automatically, and participants unlikely to have been aware of the SWAT. |
| Blinding of outcome assessment ok? | Low risk | Outcome of interest was objective (proportion of participants recruited to host trial). |
| Incomplete outcome data handled ok? | Low risk | No obvious missing data. |
| Free of selective reporting? | Low risk | Outcomes are reported as stated. |
| Was the study free of other bias? | High risk | Queried allocation method with authors, and although cookies were used to track individual IP addresses, SWAT participants may have viewed different landing pages if they accessed the website on a different landing device or were in incognito mode. This meant that there was the potential for contamination bias. |
| Overall bias? | High risk | High risk of bias, as participants were randomised according to their IP address, meaning they could have seen a different landing page if they accessed the website on incognito mode or from a different digital device, which would constitute contamination bias. |

Miller 2021b

|  |  |  |
| --- | --- | --- |
| ***Study characteristics*** | | |
| Methods | 3-arm individually-randomised study within a trial | |
| Data | Setting: Community-based research clinics, USA. Participants were 2605 participants accessing the study website during the substudy period. The host trial was recruiting patients (aged 70+) at elevated risk of falling with low serum Vitamin D. | |
| Comparisons | Investigating different wording of risk of underlying condition.  Intervention: Version B infographic on the website landing page stating that “According to the CDC, an older adult dies every 20 minutes from an unintended fall. Many more are injured.”    Comparator: Original landing page | |
| Outcomes | Proportion recruited to host trial | |
| ***Risk of bias*** | | |
| **Bias** | **Authors' judgement** | **Support for judgement** |
| Random sequence generation ok? | Low risk | “*In this experiment, web-users were randomized to one of four landing pages, one of which was the original landing page and three of which included a video.*” Participants were randomised using Optimizely web portal.  From Fig. 1: “*Digital randomization completed by A/B testing platform.*”  Randomised using Optimizely web portal. |
| Allocation concealment? | Low risk | Could not have been foreseen, as was done via a web portal. |
| Blinding of participants and personnel ok? | Low risk | Done automatically, and participants unlikely to have been aware of the SWAT. |
| Blinding of outcome assessment ok? | Low risk | Outcome of interest was objective (proportion of participants recruited to host trial). |
| Incomplete outcome data handled ok? | Low risk | No obvious missing data. |
| Free of selective reporting? | Low risk | Outcomes are reported as stated. |
| Was the study free of other bias? | High risk | Queried allocation method with authors, and although cookies were used to track individual IP addresses, SWAT participants may have viewed different landing pages if they accessed the website on a different landing device or were in incognito mode. This meant that there was the potential for contamination bias. |
| Overall bias? | High risk | High risk of bias, as participants were randomised according to their IP address, meaning they could have seen a different landing page if they accessed the website on incognito mode or from a different digital device, which would constitute contamination bias. |

Monaghan 2007

|  |  |  |
| --- | --- | --- |
| ***Study characteristics*** | | |
| Methods | Cluster-randomised controlled trial | |
| Data | Setting: existing, multicentre, international trial. 167 clinical sites in 19 countries recruiting to a diabetes and vascular disease treatment trial | |
| Comparisons | Investigated the effect of different levels of communication between the trial co-ordination team and participating sites  Additional communication – usual plus frequent emails, regular personalised mail-outs of league tables/graphs of performance against other sites, certificates of achievement for recruitment/other study items (1 per month). This was compared to usual communication (provided via the regional centre) plus occasional direct communications from the co-ordinating centre in the form of generic newsletters, emails and faxes. | |
| Outcomes | Proportion recruited to trial | |
| Notes | Clustering was not accounted for in analysis. | |
| ***Risk of bias*** | | |
| **Bias** | **Authors' judgement** | **Support for judgement** |
| Random sequence generation ok? | Low risk | Computer-generated randomisation |
| Allocation concealment? | Low risk | Central randomisation |
| Blinding of participants and personnel ok? | Low risk | Centres were blinded, but the central office was not blind |
| Blinding of outcome assessment ok? | Low risk | Objective outcome |
| Incomplete outcome data handled ok? | Low risk | Adequate |
| Free of selective reporting? | Low risk | Recruitment outcome (per site) presented, which is what review needs |
| Was the study free of other bias? | Low risk | No other biases apparent |
| Overall bias? | Low risk | Low risk of bias |

Nystuen 2004

|  |  |  |
| --- | --- | --- |
| ***Study characteristics*** | | |
| Methods | Randomised controlled trial | |
| Data | Setting: community, Norway. 498 sick-listed employees attending a participating social security office | |
| Comparisons | Investigated the effect of different telephone reminders  Written invitation to participate in a community-based trial followed by a telephone reminder if no response within 2 weeks; guide used for discussion. This was compared to written invitation to participate in a community-based trial followed by no reminder if no response within 2 weeks. | |
| Outcomes | Proportion recruited to trial | |
| ***Risk of bias*** | | |
| **Bias** | **Authors' judgement** | **Support for judgement** |
| Random sequence generation ok? | Low risk | Computer-generated list |
| Allocation concealment? | Low risk | Central allocation |
| Blinding of participants and personnel ok? | Low risk | Participants were blinded but not the research team who makes the phone calls. The team do not contact the control group. |
| Blinding of outcome assessment ok? | Low risk | Objective outcome |
| Incomplete outcome data handled ok? | Low risk | Adequate |
| Free of selective reporting? | Low risk | Recruitment outcome presented, which is all the review needs |
| Was the study free of other bias? | Low risk | No other biases apparent |
| Overall bias? | Low risk | Low risk of bias |

Paris 2015

|  |  |  |
| --- | --- | --- |
| ***Study characteristics*** | | |
| Methods | 2-arm individually-randomised study within a trial | |
| Data | Setting: Hospital, France. Participants were 480 adults (18+ years) across six French clinical research centres who were considered suitable for an ongoing trial. | |
| Comparisons | Investigating simplified PILs.  Intervention: Modified PILs revised for better readability using simpler language, a glossary, a summary, and a study diagram.   Comparator: Original PIL. | |
| Outcomes | Proportion recruited to host trial | |
| ***Risk of bias*** | | |
| **Bias** | **Authors' judgement** | **Support for judgement** |
| Random sequence generation ok? | Low risk | Envelopes were randomized electronically (Clininfo, Lyon, France) using variable block sizes and stratified by study and centre. |
| Allocation concealment? | Low risk | During the information/screening visit for a participating clinical study the investigator opened the consecutive randomized envelope and gave the patient the ICD and the questionnaire inside. In order to avoid randomization bias, the consecutive allocation of sealed envelopes was checked in each centre by a clinical research assistant. |
| Blinding of participants and personnel ok? | Low risk | Participants were not blinded, however this is unlikely to have biased the study |
| Blinding of outcome assessment ok? | Low risk | The study design did not permit blinding of consenters and physicians, and we cannot rule out a bias, but this seems unlikely given that both physicians and IRB members preferred the modified consent documents and expected no effect on consent rates. |
| Incomplete outcome data handled ok? | Low risk | Data presented in a flowchart, including potential participants that dropped out and multiple imputation in intention to treat analysis. |
| Free of selective reporting? | Low risk | All outcome measures reported. |
| Was the study free of other bias? | Low risk | No other biases were identified. |
| Overall bias? | Low risk | This study was well conducted with low risk of bias in most domains. Selection bias was minimised through electronic randomisation and verified concealment. Although the risk for blinding of participants and personnel is unclear because physicians and consenters were not masked, the objective primary outcome and rigorous intention to treat analysis support the findings. |

Parker 2018 (START PIS ECLS)

|  |  |  |
| --- | --- | --- |
| ***Study characteristics*** | | |
| Methods | 2-arm individually-randomised study within a trial | |
| Data | Setting: General Practice, UK. Participants were 2262 patients identified from GP lists who were aged 50-75 years who were current or ex-smokers that had smoked at least 20 cigarretes per day for 20 years. | |
| Comparisons | Investigating optimised participant information brochure.  Intervention: Optimised participant information brochure accompanying GP letter developed using performance-based user-testing process to improve readability, layout, and navigation, involving iterative feedback from volunteers with similar demographics to the target study sample.  Comparator: Standard participant information brochure developed by the host trial team, formatted by a professional design company | |
| Outcomes | Proportion recruited to host trial | |
| ***Risk of bias*** | | |
| **Bias** | **Authors' judgement** | **Support for judgement** |
| Random sequence generation ok? | Low risk | Potential participants identified from GP lists as eligible were randomly allocated to receive the control PIB or intervention (user-tested PIB) and GP covering invita- tion letter at a 1:1 ratio using the recruitment-tracking software developed by the Health Informatics Centre, University of Dundee, and the TCTU. |
| Allocation concealment? | Low risk | An independent statistician (VM) who conducted analyses remained blind to allocation until the analyses were complete. |
| Blinding of participants and personnel ok? | Unclear risk | Paper does not provide enough information to determine whether participants or study staff were blinded to group allocation. |
| Blinding of outcome assessment ok? | Unclear risk | An independent statistician (VM) who conducted analyses remained blind to allocation until the analyses were complete. No mention of others. |
| Incomplete outcome data handled ok? | Low risk | Doesn’t look like there were any missing data. |
| Free of selective reporting? | Low risk | Expected outcomes reported. |
| Was the study free of other bias? | Low risk | No other sources of bias was identified |
| Overall bias? | Unclear risk | Unclear due to Blinding of participants and personnel and detection bias being unclear |

Paul 2011

|  |  |  |
| --- | --- | --- |
| ***Study characteristics*** | | |
| Methods | Randomised controlled trial | |
| Data | Setting: secondaty care, UK. Participants were patients with colorectal cancer receiving adjuvant treatment. 215 were allocated to the comparator; it was unclear how many received the intervention. | |
| Comparisons | Investigated the effect of the randomisation time point  Intervention: randomise prior to treatment to get 3 or 6 months treatment  Comparator: randomise after 3 months of treatment to see if participant gets another 3 months of treatment | |
| Outcomes | Proportion recruited to trial | |
| ***Risk of bias*** | | |
| **Bias** | **Authors' judgement** | **Support for judgement** |
| Random sequence generation ok? | Low risk | Received additional information from Jim Paul by email (Paul 2016). Minimisation programmed in PL/SQL in Oracle |
| Allocation concealment? | Low risk | Central allocation |
| Blinding of participants and personnel ok? | Low risk | Participants blinded |
| Blinding of outcome assessment ok? | Low risk | Objective outcome (recruitment) |
| Incomplete outcome data handled ok? | Low risk | Adequate |
| Free of selective reporting? | Low risk | Recruitment outcome available, which is all the review needs |
| Was the study free of other bias? | Low risk | No other biases apparent |
| Overall bias? | Low risk | Low risk of bias |

Paul 2014

|  |  |  |
| --- | --- | --- |
| ***Study characteristics*** | | |
| Methods | Randomised controlled trial | |
| Data | Setting: community (via cancer registry), Australia. 1062 participants were 18 years or older, primary colorectal cancer diagnosis and within 3 months of diagnosis and on registry | |
| Comparisons | Investigated pre-recruitment primer letter  Intervention: pre-recruitment primer letter designed to encourage participation  Comparison: no primer letter | |
| Outcomes | Proportion recruited to trial | |
| ***Risk of bias*** | | |
| **Bias** | **Authors' judgement** | **Support for judgement** |
| Random sequence generation ok? | Low risk | Adequate |
| Allocation concealment? | Low risk | Done centrally from register |
| Blinding of participants and personnel ok? | Low risk | Adequate |
| Blinding of outcome assessment ok? | Low risk | Adequate |
| Incomplete outcome data handled ok? | Low risk | Adequate |
| Free of selective reporting? | Low risk | Recruitment reported ,and this is only outcome needed for review |
| Was the study free of other bias? | Low risk | No other biases apparent |
| Overall bias? | Low risk | Low risk of bias |

Rogers 2019

|  |  |  |
| --- | --- | --- |
| ***Study characteristics*** | | |
| Methods | 2-arm individually-randomised study within a trial | |
| Data | Setting: General Practice, UK. Participants were 1050 patients aged 60 or over, taking allopurinol for chronic gout, with additional cardiovascular risk factors | |
| Comparisons | Investigating DVD with invitation pack.  Intervention: DVD presentation and standard invitation pack. The DVD explained the background and operation of the host trial.  Comparator: Standard invitation pack only. | |
| Outcomes | Proportion recruited to host trial, cost-effectiveness. | |
| ***Risk of bias*** | | |
| **Bias** | **Authors' judgement** | **Support for judgement** |
| Random sequence generation ok? | Low risk | Parallel-arm 1:1 randomisation at a trial coordinating centre. |
| Allocation concealment? | Unclear risk | Whereas allocation concealment occurred properly by the trial coordinating centre, there is a risk of patients allocated to the control group who could access the patient information video as it is openly accessible on Youtube, which would undermine allocation concealment. |
| Blinding of participants and personnel ok? | Low risk | Study nurses were not blinded to the DVD group allocation since, however, the primary endpoint of positive response to invitation occurred prior to screening visits. SWAT participants were blinded. |
| Blinding of outcome assessment ok? | Low risk | Objective outcome (proportion recruited to host trial), therefore not an issue |
| Incomplete outcome data handled ok? | Unclear risk | Contradicting evidence. Whereas the CONSORT diagram suggests that in the DVD invitation group, 11 potential participants were lost to follow-up and 498 were overall analysed, in the analysis itself 501 participants were analysed. The missing data were handled by removing completely data from patients lost to follow-up; this may introduce attrition bias. |
| Free of selective reporting? | Low risk | There is no evidence of reporting bias in the study. |
| Was the study free of other bias? | Low risk | No other source of bias has been found in the study. |
| Overall bias? | Unclear risk | Unclear risk of bias as described in individual items above |

Saif 2020

|  |  |  |
| --- | --- | --- |
| ***Study characteristics*** | | |
| Methods | 2-arm individually-randomised study within a trial | |
| Data | Setting: Online, USA. Participants were 351 individuals aged 65 to 85 with a self-reported diagnosis of Alzheimer's disease | |
| Comparisons | Investigating web-based course.  Intervention: Six-lesson interactive web-based course on Alzheimer's disease accessible via computer, tablet, and personal mobile devices.    Comparator: Time-neutral control: One time-matched interactive webinar lesson on Alzheimer's disease statistics and public policy, and time-matched video lessons | |
| Outcomes | Proportion recruited to host trial | |
| ***Risk of bias*** | | |
| **Bias** | **Authors' judgement** | **Support for judgement** |
| Random sequence generation ok? | Low risk | “Participants were randomized (1:1) using a random number generator” |
| Allocation concealment? | Low risk | Unlikely that allocation could have been foreseen |
| Blinding of participants and personnel ok? | Unclear risk | Not stated if participants were blinded to the SWAT |
| Blinding of outcome assessment ok? | Unclear risk | Unclear - implied all online |
| Incomplete outcome data handled ok? | Low risk | No incomplete outcome data reported |
| Free of selective reporting? | Low risk | Primary outcome reported |
| Was the study free of other bias? | Low risk | No other sources of bias identified |
| Overall bias? | Unclear risk | Unclear RoB as not stated if participants were blinded to the SWAT and if outcome assessment was blinded |

Sheridan 2020 (START PIS ISDR)

|  |  |  |
| --- | --- | --- |
| ***Study characteristics*** | | |
| Methods | 2-arm cluster-randomised study within a trial | |
| Data | Setting: Hospital, UK. Participants were 815 individuals aged 16 years or older who were diabetic and scheduled to attend retinal screening. | |
| Comparisons | Investigating optimised, user-tested PIS.  Intervention: Optimised study invitation letter and 12-page PIS developed through two rounds of user testing.    Comparator: Standard study invitation letter and 5-page PIS alongside their annual screening clinic appointment. | |
| Outcomes | Proportion recruited to host trial | |
| ***Risk of bias*** | | |
| **Bias** | **Authors' judgement** | **Support for judgement** |
| Random sequence generation ok? | Low risk | Cluster randomised allocation to receive the standard or optimised PIS by week of mail-out (1:1), by random number generator, determined by date of clinic appointment. |
| Allocation concealment? | Low risk | Concealment of allocation was achieved because the appointment schedule was set before SWAT allocations were randomised. |
| Blinding of participants and personnel ok? | High risk | Recruiting researchers were not masked as they saw the ISDR booklet the patients brought with them; patients were not masked but were nevertheless unaware that a SWAT was ongoing. |
| Blinding of outcome assessment ok? | High risk | Recruiting researchers were not masked as they saw the ISDR booklet the patients brought with them. |
| Incomplete outcome data handled ok? | Low risk | Doesn’t look like there were any missing data? |
| Free of selective reporting? | Low risk | Expected outcomes reported. |
| Was the study free of other bias? | Low risk | Not incomplete outcome data, but worthy of note: 620 patients attended an appointment when no researcher was present and therefore were not asked to participate in ISDR. Sensitivity analysis including those patients did not substantially alter results. |
| Overall bias? | High risk | Overall high risk of bias due to answering ‘No’ for blinding of participants/personnel and outcome assessment as per the comments |

Simes 1986

|  |  |  |
| --- | --- | --- |
| ***Study characteristics*** | | |
| Methods | Randomised controlled trial | |
| Data | Setting: secondary care, Australia. 57 patients attending an oncology unit | |
| Comparisons | Investigated the effect of different consent methods  Individual approach to consent – patients given information about aims, expected results, potential toxicities of treatment; details of treatment left to discretion of consultant; patients given opportunity to ask questions, verbal consent obtained. This was compared to total disclosure approach – participants were fully informed about all trial aspects by consultant, with opportunity to ask questions and a consent form outlining the information; this was kept overnight, and written consent was obtained the following day. | |
| Outcomes | Proportion recruited to trial | |
| ***Risk of bias*** | | |
| **Bias** | **Authors' judgement** | **Support for judgement** |
| Random sequence generation ok? | Low risk | Sealed envelopes using balanced randomisation |
| Allocation concealment? | Unclear risk | Unclear if envelopes were sequentially numbered |
| Blinding of participants and personnel ok? | Unclear risk | Participants were probably blinded. Clinicians were probably not blinded. It is not clear if it is the same clinicians provided information in to both groups. |
| Blinding of outcome assessment ok? | Low risk | Objective outcome |
| Incomplete outcome data handled ok? | Low risk | Adequate |
| Free of selective reporting? | Low risk | Recruitment outcome presented, which is all the review needs |
| Was the study free of other bias? | Low risk | No other biases apparent |
| Overall bias? | Unclear risk | Unclear risk of bias |

Skinner 2019

|  |  |  |
| --- | --- | --- |
| ***Study characteristics*** | | |
| Methods | 2-arm individually-randomised study within a trial | |
| Data | Setting: Hospital, USA. Participants were 63 individuals diagnosed with malignancy, aged 18 years or older, with no previous history of clinical trial participation | |
| Comparisons | Investigating in-office video viewing.  Intervention: In-office viewing of video depicting oncology patient advocates and oncologists discussing clinical trials. Participants were also given an educational booklet and a DVD copy of the video.   Comparator: Clinical trial educational booklet and a DVD copy of the video on cancer clinical trials to take home. | |
| Outcomes | Proportion recruited to host trial | |
| ***Risk of bias*** | | |
| **Bias** | **Authors' judgement** | **Support for judgement** |
| Random sequence generation ok? | Low risk | Participants were randomized to the intervention (in-office video viewing group) or control, (DVD to take home, usual care group) using the web-based program Research Randomizer (4.0). |
| Allocation concealment? | Unclear risk | No mention of this in the report. |
| Blinding of participants and personnel ok? | High risk | From SWAT registration (https://trialbulletin.com/lib/entry/ct-02600533) :  Masking: None (Open Label) |
| Blinding of outcome assessment ok? | Low risk | Patients were likely blind to outcome assessment. Enrollment in clinical trials was assessed 1-year post follow-up visit by review of VICC clinical trial participation electronic record. |
| Incomplete outcome data handled ok? | Low risk | Flow chart outlining flow of participants and drop outs. |
| Free of selective reporting? | Low risk | All outcomes are reported. |
| Was the study free of other bias? | Low risk | No additional comment |
| Overall bias? | High risk | Lack of blinding and clear allocation concealment. |

Sundaresan 2017

|  |  |  |
| --- | --- | --- |
| ***Study characteristics*** | | |
| Methods | 2-arm individually-randomised study within a trial | |
| Data | Setting: Hospital, Australia and New Zealand. Participants were 129 men aged 18 and over, with a diagnosis of prostate cancer and cancer cells that had not been removed or have spread beyond the prostate at radical prostatectomy | |
| Comparisons | Investigating decision aid.  Intervention: Decision Aid including evidence-based information on pros and cons of management options, general information about RCTs, and value-clarification exercises.   Comparator: Blank notebook of identical weight and shape to the Decision Aid. | |
| Outcomes | Proportion recruited to host trial | |
| ***Risk of bias*** | | |
| **Bias** | **Authors' judgement** | **Support for judgement** |
| Random sequence generation ok? | Low risk | “Randomisation was performed at the central research office of the Psycho Oncology Co-operative Group (PoCoG), at the University of Sydney. Random block sizes of 2, 4 and 6 were employed with stratification by recruiting site. Each recruiting site was provided with a box of pre-randomised, consecutively ordered and numbered study packages.” |
| Allocation concealment? | Low risk | “The blank notebook was the same size and shape as the Decision Aid so that all packages were of identical weight  and shape to maintain allocation concealment” |
| Blinding of participants and personnel ok? | Low risk | State that “ the current Decision Aid (DA) study – a phase III, double blind, randomised controlled study”, however write that “After a brief introduction to the Decision Aid study, recruiting clinicians gave interested men a pre-randomised, sealed, study package and advised them that they would be contacted by the  Decision Aid study team” |
| Blinding of outcome assessment ok? | Low risk | “ the current Decision Aid (DA) study – a phase III, double blind, randomised controlled study” RAVES enrollment was an objective outcome |
| Incomplete outcome data handled ok? | Low risk | No concerns about incomplete outcome data. |
| Free of selective reporting? | Low risk | No selective reporting. |
| Was the study free of other bias? | Low risk | No other source of bias was identified. |
| Overall bias? | Low risk | No concerns about sources of bias. |

Tilley 2012

|  |  |  |
| --- | --- | --- |
| ***Study characteristics*** | | |
| Methods | Cluster-randomised controlled trial | |
| Data | Setting: primary care, USA. Participants were neurologists, primary care docs and internists within 30 miles of trial site. Intention was that this would increase proportion of non-white, non-Hispanic participants into the trial. Participants being enrolled had Parkinson's. 616 participants in analysis | |
| Comparisons | Investigated effect of a recruitment coordinator  Intervention: recruitment coordinator plus package of training, materials and events, some carrying CME points.  Comparator: whatever recruitment procedures sites wanted to use | |
| Outcomes | Proportion recruited to trial | |
| ***Risk of bias*** | | |
| **Bias** | **Authors' judgement** | **Support for judgement** |
| Random sequence generation ok? | Low risk | Adequate |
| Allocation concealment? | Unclear risk | No details given |
| Blinding of participants and personnel ok? | Low risk | Possible that intervention sites mentioned what they were doing to control sites but controls did not have the coordinator and funding for events so unlikely to really influence outcome, which was anyway objective (recruitment) |
| Blinding of outcome assessment ok? | Low risk | Adequate |
| Incomplete outcome data handled ok? | Low risk | Adequate |
| Free of selective reporting? | Unclear risk | Recruitment reported and this is only outcome needed for review |
| Was the study free of other bias? | High risk | Stopped early because of a formal stopping rule |
| Overall bias? | High risk | High risk of bias |

Tilley 2021

|  |  |  |
| --- | --- | --- |
| ***Study characteristics*** | | |
| Methods | 2-arm cluster-randomised study within a trial | |
| Data | Setting: Mixed (university and specialist care), USA. Participants were 2,107 individuals eligible for one of four RCTs across 50 sites. Sites were all located in an area where at least 20% of the individuals in the eligile age groups are from minority backgrounds. | |
| Comparisons | Investigating trust-based educational program.  Intervention: Trust-based educational program promoting relationships between physician-investigators and minority-serving physicians and their minority patients .   Comparator: Recruitment as usual. | |
| Outcomes | Proportion recruited to host trial | |
| ***Risk of bias*** | | |
| **Bias** | **Authors' judgement** | **Support for judgement** |
| Random sequence generation ok? | Low risk | “Sites, the units of analyses, were matched within parent trial, where possible, on total minorities living within a 30-mile radius of the site, past minority recruitment or projected minority enrollment at the site, and geographic region. Within each pair, sites were randomly assigned to intervention or control using simple randomization. Simple randomization for each pair was based on a single sequence of random assignments, in this case a random numbers table.” |
| Allocation concealment? | Low risk | Allocation unlikely to have been foreseen. |
| Blinding of participants and personnel ok? | Unclear risk | “Also, to participate in RECRUIT, each site Principal Investigator and Coordinator consented to be randomized to intervention or control without knowing the specifics of the recruitment intervention (since the RECRUIT trial was un-blinded).”  Unblinding of personnel may have potentially introduced some bias, even though the personnel weren’t aware of the specific intervention. |
| Blinding of outcome assessment ok? | Low risk | Outcome of interest for this review (enrollment rate) was objective |
| Incomplete outcome data handled ok? | Low risk | “There were no missing values for the primary outcome (proportion of minorities enrolled) or for counts of participants enrolled or minorities enrolled.” |
| Free of selective reporting? | Low risk | No selective reporting. |
| Was the study free of other bias? | Low risk | No other sources of bias were identified |
| Overall bias? | Unclear risk | Unclear risk of bias, as some potential concerns around blinding of personnel an outcome assessment. |

Trevena 2006

|  |  |  |
| --- | --- | --- |
| ***Study characteristics*** | | |
| Methods | Randomised controlled trial | |
| Data | Setting: primary care, Australia. 152 participants aged 50 to 74 eligible for a colorectal cancer screening trial | |
| Comparisons | Investigated the effect of different trial information methods  Opt-in recruitment; letter from doctor advising that the practice is taking part in screening trial; would only be contacted if contact details returned. This was compared to opt-out recruitment; letter from doctor advising that the practice is taking part in screening trial; would be contacted unless the practice was advised to withhold contact details  The distribution of participants between intervention and comparison groups is uneven: 60 versus 92, respectively. This was due to a change in legislation in Australia, which meant that the trialists could no longer continue with the opt-out procedure and had to change to opt-in to keep their ethical approval. | |
| Outcomes | Proportion recruited to trial | |
| ***Risk of bias*** | | |
| **Bias** | **Authors' judgement** | **Support for judgement** |
| Random sequence generation ok? | Low risk | Computer-generated randomisation |
| Allocation concealment? | Unclear risk | Unclear if randomisation list was open |
| Blinding of participants and personnel ok? | Low risk | Participants not told about different recruitment methods. Not clear if clinicians were blinded but they were not involved in recruitment, which was done by letter and then contact with research team. |
| Blinding of outcome assessment ok? | Low risk | See above |
| Incomplete outcome data handled ok? | Low risk | Adequate |
| Free of selective reporting? | Low risk | Recruitment outcome presented, which is all the review needs |
| Was the study free of other bias? | Low risk | No other biases apparent |
| Overall bias? | Unclear risk | Unclear risk of bias |

Treweek 2012

|  |  |  |
| --- | --- | --- |
| ***Study characteristics*** | | |
| Methods | Randomised controlled trial | |
| Data | Setting: primary care, UK. Participants were 1760 GPs | |
| Comparisons | Investigated use of different modes of invitation to take part in trial  Intervention: email invitation (email plus link to info sheet - text the same as with intervention)  Comparator: postal invitation (letter plus 2-page information sheet) | |
| Outcomes | Proportion recruited to trial | |
| ***Risk of bias*** | | |
| **Bias** | **Authors' judgement** | **Support for judgement** |
| Random sequence generation ok? | Low risk | Centrally generated by statistician using computer |
| Allocation concealment? | Low risk | 3rd party used to send out invitations |
| Blinding of participants and personnel ok? | Low risk | Research team blind. Participants did not know study was ongoing so also blind |
| Blinding of outcome assessment ok? | Low risk | Adequate |
| Incomplete outcome data handled ok? | Low risk | Adequate |
| Free of selective reporting? | Low risk | Recruitment reported and this is only outcome needed for review |
| Was the study free of other bias? | Low risk | No other biases apparent |
| Overall bias? | Low risk | Low risk of bias |

Veerus 2016

|  |  |  |
| --- | --- | --- |
| ***Study characteristics*** | | |
| Methods | 2-arm individually-randomised study within a trial | |
| Data | Setting: 3 clinical centres, Estonia. Participants were 4171 postmenopausal women aged 50-64 years. | |
| Comparisons | Intervention: Non-blind subtrial, where participants were informed of whether they were receiving the intervention (hormonal therapy) or an inactive control before confirming willingness and signing informed consent forms    Comparator: Blind subtrial, where participants were not informed of whether they were receiving the intervention (hormonal therapy) or an inactive control before confirming willingness and signing informed consent forms. | |
| Outcomes | Proportion recruited to host trial | |
| ***Risk of bias*** | | |
| **Bias** | **Authors' judgement** | **Support for judgement** |
| Random sequence generation ok? | Low risk | “Randomisation was performed from 1999 to 2001 in seven batches, using a computer-based stratiﬁed block randomisation program (block size 16).” |
| Allocation concealment? | Low risk | Intentional due to study design. Decided to reclassify as “Yes” as was intentional due to the study design, and to be consistent with previously-included studies assessing open- vs blinded trial design. |
| Blinding of participants and personnel ok? | Low risk | Intentional due to study design |
| Blinding of outcome assessment ok? | Low risk | Proportion recruited is an objective outcome. Willingness, eligibility, and attendance assessed by directly observed patient turnout. |
| Incomplete outcome data handled ok? | Unclear risk | Handling of incomplete outcome data not discussed. |
| Free of selective reporting? | Low risk | Stated outcomes all presented. |
| Was the study free of other bias? | Low risk | No other source of bias was identified. |
| Overall bias? | Unclear risk | Unclear risk of bias as handling of incomplete outcome data not discussed. |

Wadland 1990

|  |  |  |
| --- | --- | --- |
| ***Study characteristics*** | | |
| Methods | Randomised controlled trial | |
| Data | Setting: primary care, USA. Participants were 104 smokers > 18 years old | |
| Comparisons | Intervention: consent form read out by researcher  Comparator: consent form read by patient | |
| Outcomes | Proportion recruited to trial | |
| Notes | Only site 2 in the study ran a randomised evaluation so only its data are included | |
| ***Risk of bias*** | | |
| **Bias** | **Authors' judgement** | **Support for judgement** |
| Random sequence generation ok? | Unclear risk | Randomisation mentioned but no more details |
| Allocation concealment? | Unclear risk | See above |
| Blinding of participants and personnel ok? | Unclear risk | Both actively involved but not clear if the participants were told about how consent might be varied |
| Blinding of outcome assessment ok? | Low risk | Objective outcome |
| Incomplete outcome data handled ok? | Low risk | Adequate |
| Free of selective reporting? | Low risk | Recruitment outcome presented, which is all the review needs |
| Was the study free of other bias? | Low risk | No other biases apparent |
| Overall bias? | Unclear risk | Unclear risk of bias |

White 2023

|  |  |  |
| --- | --- | --- |
| ***Study characteristics*** | | |
| Methods | 2x2 factorial individually-randomised study within a trial | |
| Data | Setting: General Practice, UK. Participants were 6,946 individuals aged 18-69 years with persistent physical (medically unexplained) symptoms. | |
| Comparisons | Investigating brief PIL and trial-branded pen.  Intervention: Standard invitation + brief PIL. The brief PIL provided a succinct, easy-to-read summary of the MSS3 trial on a folded, high-quality A4 leaflet, designed to help potential participants assess initial interest before accessing the standard, detailed PIL.   Intervention 2: Standard invitation + trial branded pen.   Intervention 3: Standard invitation + brief PIL + trial branded pen.   Comparator: Standard invitation only | |
| Outcomes | Proportion recruited to host trial, cost-effectiveness. | |
| ***Risk of bias*** | | |
| **Bias** | **Authors' judgement** | **Support for judgement** |
| Random sequence generation ok? | Low risk | Factorial individual-level 2x2 randomisation, using randomly-permuted blocks of length four or eight, equal allocation ratio and stratification by GP centre. |
| Allocation concealment? | Low risk | The participants did not know the alternative allocations. |
| Blinding of participants and personnel ok? | Low risk | Whereas it was not possible to entirely blind practice staff to the interventions as it will have been clear that some packs contained pens, patients did not know that they were part of a trial testing recruitment interventions hence were blind to the SWAT hypothesis, CTRU staff undertaking MSS3 trial recruitment were blind to the SWAT group to which patients were allocated. |
| Blinding of outcome assessment ok? | Low risk | The participants were unaware of receiving a specific recruitment strategy, for the purpose of improving patient recruitment in randomised trials. |
| Incomplete outcome data handled ok? | Unclear risk | The review authors mentioned that five missing data were removed from the analysis. However, such an approach may introduce attrition bias. |
| Free of selective reporting? | Low risk | There is no evidence of reporting bias in the study |
| Was the study free of other bias? | Low risk | No other source of bias has been found in the study. |
| Overall bias? | Unclear risk | Unclear risk of bias as some missing data was removed from the analyses. |

Whiteside 2019

|  |  |  |
| --- | --- | --- |
| ***Study characteristics*** | | |
| Methods | 2-arm individually-randomised study within a trial | |
| Data | Setting: General Practice, UK. Participants were 1862 community-dwelling older adults (65+), who were willing to receive an occupational therapist home visit who either have experienced a fall in the last 12 months or feel worried about falling in the future. | |
| Comparisons | Investigating branded pen with invitation pack.  Intervention: trials unit branded pen sent with their invitation pack.   Comparator: Invitation pack without branded pen: Included an invitation letter, a participant information sheet, consent form, screening form, and a pre-paid return envelope | |
| Outcomes | Proportion recruited to host trial, costs, proportion retained at 3 months. | |
| ***Risk of bias*** | | |
| **Bias** | **Authors' judgement** | **Support for judgement** |
| Random sequence generation ok? | Low risk | Participants were allocated to the intervention (pen) or control (no pen) using block randomisation stratified by GP practice, with the sequence generated by a statistician using Stata v13. |
| Allocation concealment? | Low risk | Unique participant ID numbers were randomised by a statistician not involved in producing the invitation packs, ensuring concealment until the packs were received by participants. |
| Blinding of participants and personnel ok? | Low risk | While not explicitly informed of the SWAT, participants were effectively blinded to the hypothesis as they only received one version of the invitation pack and were unaware of the alternative arm. |
| Blinding of outcome assessment ok? | Low risk | Not clearly mentioned. However, the participants should be unaware of receiving a specific recruitment strategy, for the purpose of improving patient recruitment in randomised trials. |
| Incomplete outcome data handled ok? | Low risk | Analysis followed intention-to-treat (ITT) principles; all participants were included in the final analysis except for a balanced number of packs not sent out prior to intervention delivery. |
| Free of selective reporting? | Low risk | There is no evidence of reporting bias in the study. |
| Was the study free of other bias? | Low risk | No other source of bias has been found in the study. |
| Overall bias? | Low risk | Low risk of bias as described in individual domains above |

Witham 2018

|  |  |  |
| --- | --- | --- |
| ***Study characteristics*** | | |
| Methods | 2-arm individually-randomised study within a trial | |
| Data | Setting: General practice, Scotland, UK. Participants were 4,145 older adults aged 65 and over, with a history of falls. | |
| Comparisons | Investigating information sheet with different wording.  Intervention: New information sheet minimizing the use of the word ‘falls’ and instead emphasising the maintenance of balance, health and wellbeing.   Comparator: Standard information sheet. | |
| Outcomes | Proportion recruited to host trial | |
| ***Risk of bias*** | | |
| **Bias** | **Authors' judgement** | **Support for judgement** |
| Random sequence generation ok? | Low risk | 1:1 randomisation was performed via a computer- generated list, with each study code being assigned to either the new sheet or the standard sheet and their respective cover letters. Randomisation was stratified by primary care practice. |
| Allocation concealment? | Low risk | Randomisation and preparation of study packs was performed by staff not other- wise engaged in the study; sealed packs were then delivered to Scottish Primary Care Research Network who added address labels and posted them to potential participants. |
| Blinding of participants and personnel ok? | Low risk | Scottish Primary Care Research Network were not aware of which pack contained which information sheet; nor were the study team. |
| Blinding of outcome assessment ok? | High risk | Intervention allocation was masked from the study team for the primary outcome, but after first contact with potential participants, it was not possible to mask the study team from intervention allocation; the researcher taking con- sent had the potential to become aware of the allocation during the consent procedure. |
| Incomplete outcome data handled ok? | Low risk | No incomplete data reported by the looks of it? |
| Free of selective reporting? | Low risk | No selective reporting. |
| Was the study free of other bias? | Low risk | No other sources of bias were identified. |
| Overall bias? | High risk | Overall marked high risk of bias due to ‘Blinding of outcome assessment (detection bias) ok?’ being marked as ‘no’ |

Wong 2013

|  |  |  |
| --- | --- | --- |
| ***Study characteristics*** | | |
| Methods | Randomised controlled trial | |
| Data | Setting: primary care, Canada. Participants were 952 people aged 50-70 years who had not responded to initial invitation by 4 weeks. People were being recruited to a colorectal cancer screening trial not had recent colorectal cancer screening | |
| Comparisons | Investigated use of telephone reminders to non-responders  Intervention: up to 3 telephone reminders to those not responding to initial posted invitation  Comparison: no telephone reminders (but did get a 2nd invitation) | |
| Outcomes | Proportion recruited to trial | |
| ***Risk of bias*** | | |
| **Bias** | **Authors' judgement** | **Support for judgement** |
| Random sequence generation ok? | Low risk | Adequate |
| Allocation concealment? | Low risk | Adequate |
| Blinding of participants and personnel ok? | Low risk | Participants blinded, study nurse making calls clearly not but outcome objective |
| Blinding of outcome assessment ok? | Low risk | Recruitment objective (this was study's secondary outcome, primary was attendance at eligibility screening) |
| Incomplete outcome data handled ok? | Low risk | Adequate |
| Free of selective reporting? | Low risk | Recruitment reported and this is only outcome needed for review |
| Was the study free of other bias? | Low risk | No other biases apparent |
| Overall bias? | Low risk | Low risk of bias |

## Footnotes

**CBT**: cognitive behavioural therapy; **CME**: continuing medical education; **CVD**: cardiovascular disease; **DSM-IV**: *Diagnostic and Statistical Manual of Mental Disorders*, Fourth Edition; **GP**: general practitioner; **HRT**: hormone replacement therapy; **NICU**: neonatal intensive care unit; **NSAIDs**: non-steroidal anti-inflammatory drugs; **PIL**: participant information leaflet; **PL/SQL**: procedural language extension to Structured Query Language; **RCT**: randomised controlled trial; **SMS**: short message service; **SOP**: standard operating protocol.

## References to studies

### Abd-Elsayed 2012 {published data only}

- Abd-Elsayed AA, Sessler DI, Mendoza-Cuartas M, Dalton JE, Said T, Meinert J, et al. A randomized controlled study to assess patients' understanding of and consenting for clinical trials using two different consent form presentations. Minerva Anestesiologica 2012;78:564-73.

### Agni 2022 {published data only}

- Agni NR, Fairhurst C, McDaid C, Reed MR, Torgerson DJ. EnTraP: a factorial randomised controlled trial embedded within world hip trauma evaluation eight COPAL investigating the effect of an enhanced trainee principal investigator package and digital nudge on recruitment rates. Research Methods in Medicine & Health Sciences 2022;3(2):33-41. [DOI: 10.1177/26320843211061297]

### Arundel 2017 {published data only}

- Arundel C, Jefferson L, Bailey M, Cockayne S, Hicks K, Loughrey L, et al. A randomized, embedded trial of pre-notification of trial participation did not increase recruitment rates to a falls prevention trial. Journal of Evaluation in Clinical Practice 2017;23(1):73-8. [DOI: 10.1111/jep.12576]

### Avenell 2004 {published data only}

- Avenell A, Grant AM, McGee M, McPherson G, Campbell MK, McGee MA; the RECORD Trial Management Group. The effects of an open design on trial participant recruitment, compliance and retention - a randomized controlled trial comparison with a blinded, placebo-controlled design. Clinical Trials (London, England) 2004;1:490-8.

### Bergenmar 2014 {published data only}

- Bergenmar M, Johansson H, Wilking N, Hatschek T, Brandberg Y. Audio-recorded information to patients considering participation in cancer clinical trials - a randomized study. Acta Oncologica 2014;53:1197-204. [DOI: 10.3109/0284186X.2014.921726]

### Bobb 2016 {published data only}

- Bobb MR, Van Heukelom PG, Faine BA, Ahmed A, Messerly JT, Bell G, et al. Telemedicine provides noninferior research informed consent for remote study enrollment: a randomized controlled trial. Academic Emergency Medicine 2016;23(7):759-65. [DOI: 10.1111/acem.12966]

### Bracken 2019 {published data only}

- Bracken K, Keech A, Hague W, Kirby A, Robledo KP, Allan C, et al. Telephone call reminders did not increase screening uptake more than SMS reminders: a recruitment study within a trial. Journal of Clinical Epidemiology 2019;1(112):45-52. [DOI: 10.1016/j.jclinepi.2019.04.009]

### Brierley 2012 {published data only}

- Brierley G, Richardson R, Torgerson DJ. Using short information leaflets as recruitment tools did not improve recruitment: a randomized controlled trial. Journal of Clinical Epidemiology 2012;65:147-54. [DOI: 10.1016/j.jclinepi.2011.06.005]

### Brown 2015 {published data only}

- Brown SD, Partee PN, Feng J, Quesenberry CP, Hedderson MM, Ehrlich SF, et al. Outreach to diversify clinical trial participation: a randomized recruitment study. Clinical Trials (London, England) 2015;12(3):205-11. [DOI: 10.1177/1740774514568125]

### Chen 2011 {published and unpublished data}

- Chen F, Rahimi K, Haynes R, Naessens K, Taylor-Clarke M, Murray C, et al. Investigating strategies to improve attendance at screening visits in a randomized trial. Trials 2011;12(Suppl 1):A111. [DOI: 10.1186/1745-6215-12-S1-A111]

### Chow 2020 {published data only}

- Chow EJ, Baldwin LM, Hagen AM, Hudson MM, Gibson TM, Kochar K, et al. Communicating health information and improving coordination with primary care (CHIIP): rationale and design of a randomized cardiovascular health promotion trial for adult survivors of childhood cancer. Contemporary Clinical Trials 2020;1(89):105915. [DOI: 10.1016/j.cct.2019.105915]

### Cockayne 2017 (START PIL REFORM) {published data only}

- Cockayne S, Fairhurst C, Adamson J, Hewitt C, Hull R, Hicks K, et al. An optimised patient information sheet did not significantly increase recruitment or retention in a falls prevention study: an embedded randomised recruitment trial. Trials 2017;18:144. [DOI: 10.1186/s13063-017-1797-7]

### Cooper 1997 {published data only}

- Cooper KG, Grant AM, Garratt AM. The impact of using a partially randomised patient preference design when evaluating alternative managements for heavy menstrual bleeding. Journal of Obstetrics and Gynaecology (Lahore) 1997;104:1367-73.

### Coyne 2003 {published data only}

- Coyne CA, Xu R, Raich P, Plomer K, Dignan M, Wenzel LB, et al. Randomized, controlled trial of an easy-to-read informed consent statement for clinical trial participation: a study of the Eastern Cooperative Oncology Group. Journal of Clinical Oncology 2003;21(5):836-42.

### Crane 2016 {published data only}

- Crane MM, LaRose JG, Espeland MA, Wing RR, Tate DF. Recruitment of young adults for weight gain prevention: randomized comparison of direct mail strategies. Trials 2016;17:1-9. [DOI: 10.1186/s13063-016-1411-4]

### Dear 2011 {published data only}

- Dear RF, Barratt AL, Askie LM, Butow PN, McGeechan K, Crossing S, et al. Impact of a cancer clinical trials web site on discussions about trial participation: a cluster randomized trial. Annals of Oncology 2012;23:1912-8. [DOI: 10.1093/annonc/mdr585]

### Du 2008 {published data only}

- Du W, Mood D, Gadgeel S, Simon MS. An educational video to increase clinical trials enrolment among lung cancer patients. Journal of Thoracic Oncology 2008;3(1):23-9.

### Du 2009 {published data only}

- Du W, Mood M, Gadgeel S, Simon MS. An educational video to increase clinical trials enrollment among breast cancer patients. Breast Cancer Research and Treatment 2009;117:339-47.

### Dwyer 2022 {published data only}

- Dwyer CP, Joyce RA, Rogers F, Hynes SM. Comparing the effects of a patient-designed-and-informed participant information leaflet in comparison with a standard, researcher-designed information leaflet on recruitment, retention and understanding: a study-within-a-trial. Contemporary Clinical Trials Communications 2022;28:100936. [DOI: 10.1016/j.conctc.2022.100936]

### Ethier 2017 {published data only}

- Ethier JF, Curcin V, McGilchrist MM, Keung SN, Zhao L, Andreasson A, et al. Esource for clinical trials: implementation and evaluation of a standards-based approach in a real world trial. International Journal of Medical Informatics 2017;1(106):17-24. [DOI: 10.1016/j.ijmedinf.2017.06.006]

### Fairhurst 2022 {published data only}

- Fairhurst C, Roche J, Bissell L, Hewitt C, Hugill-Jones J, Howsam J, et al. A 2x2 randomised factorial SWAT of the use of a pen and small, financial incentive to improve recruitment in a randomised controlled trial of yoga for older adults with multimorbidity. F1000Research 2022;10:326. [DOI: 10.12688/f1000research.52164.2]

### Felicitas-Perkins 2017 {published data only}

- Felicitas-Perkins JQ, Palalay MP, Cuaresma C, Ho RC, Chen Jr MS, Dang J, et al. A pilot study to determine the effect of an educational DVD in Philippine languages on cancer clinical trial participation among Filipinos in Hawai ‘i. Hawai'i Journal of Medicine & Public Health 2017;76(7):171. [PMID: 28721310]

### Ford 2004 {published data only}

- Ford ME, Havstad SL, Davis SD. A randomized trial of recruitment methods for older African American men in the Prostate, Lung, Colorectal and Ovarian (PLCO) cancer screening trial. Clinical Trials (London, England) 2004;1:343-51.

### Foss 2016 {published and unpublished data}

- Foss KT, Kjærgaard J, Stensballe LG, Greisen G. Recruiting to clinical trials on the telephone - a randomized controlled trial. Trials 2016;17:552. [DOI: 10.1186/s13063-016-1680-y]

### Fowell 2006 {published data only}

- Fowell A, Johnstone R, Finlay IG, Russell D, Russell IT. Design of trials with dying patients: a feasibility study of cluster randomisation versus randomised consent. Palliative Medicine 2006;20:799-804.

### Fracasso 2013 {published data only}

- Fracasso PM, Goodner SA, Creekmore AN, Morgan HP, Foster DM, Hardmon AA, et al. Coaching intervention as a strategy for minority recruitment to cancer clinical trials. Journal of Oncology Practice 2013;9:294-301.

### Free 2010 {published and unpublished data}

- Free C, Hoile E, Robertson S, Knight R. Three controlled trials of interventions to increase recruitment to a randomized controlled trial of mobile phone based smoking cessation support. Clinical Trials (London, England) 2010;7:265-73.

### Free 2011 {published data only}

- Free CJ, Hoile E, Knight R, Robertson S, Devries KM. Do messages of scarcity increase trial recruitment? Contemporary Clinical Trials 2011;32:36-9. [DOI: 10.1016/j.cct.2010.09.002]

### Garvelink 2015 {published data only}

- Garvelink MM, Freitas A, Menear M, Brière N, Stacey D, Légaré F. In for a penny, in for a pound: the effect of pre-engaging healthcare organizations on their subsequent participation in trials. BMC Research Notes 2015;8:1-5. [DOI: 10.1186/s13104-015-1743-2]

### Giguere 2015 {published data only}

- Giguere AM, Labrecque M, Borduas F, Rouleau M. Effectiveness of monetary incentives to recruit family physicians as study subjects: a randomized controlled trial. BMC Research Notes 2015;8:1-4. [DOI: 10.1186/s13104-014-0969-8]

### Halpern 2021a ($200) {published data only}

- Halpern SD, Chowdhury M, Bayes B, Cooney E, Hitsman BL, Schnoll RA, et al. Effectiveness and ethics of incentives for research participation: 2 randomized clinical trials. JAMA Internal Medicine 2021;181(11):1479-88. [DOI: 10.1001/jamainternmed.2021.5450]

### Halpern 2021a ($500) {published data only}

- Halpern SD, Chowdhury M, Bayes B, Cooney E, Hitsman BL, Schnoll RA, et al. Effectiveness and ethics of incentives for research participation: 2 randomized clinical trials. AMA Internal Medicine 2021;181(11):1479-88. [DOI: 10.1001/jamainternmed.2021.5450]

### Halpern 2021b ($100) {published data only}

- Halpern SD, Chowdhury M, Bayes B, Cooney E, Hitsman BL, Schnoll RA, et al. Effectiveness and ethics of incentives for research participation: 2 randomized clinical trials. JAMA Internal Medicine 2021;181(11):1479-88. [DOI: 10.1001/jamainternmed.2021.5450]

### Halpern 2021b ($300) {published data only}

- Halpern SD, Chowdhury M, Bayes B, Cooney E, Hitsman BL, Schnoll RA, et al. Effectiveness and ethics of incentives for research participation: 2 randomized clinical trials. JAMA Internal Medicine 2021;181(11):1479-88. [DOI: 10.1001/jamainternmed.2021.5450]

### Hemminki 2004 {published data only}

- Hemminki E, Hovi SL, Veerus P, Sevon T, Tuimala R, Rahu M, et al. Blinding decreased recruitment in a prevention trial of postmenopausal hormone therapy. Journal of Clinical Epidemiology 2004;57:1237-43.

### Hughes-Morley 2016 {published data only}

- Hughes-Morley A, Hann M, Fraser C, Meade O, Lovell K, Young B, et al. The impact of advertising patient and public involvement on trial recruitment: embedded cluster randomised recruitment trial. Trials 2016;17:1-3. [DOI: 10.1186/s13063-016-1718-1]

### Hutchison 2007 {published data only}

- Hutchison C, Cowan C, McMahon T, Paul J. A randomised controlled study of an audiovisual patient information intervention on informed consent and recruitment to cancer clinical trials. British Journal of Cancer 2007;97:705-11.

### Isaksson 2025 {published data only}

- Isaksson E, Näsman P, Wester P, Laska AC, Lundström E. Enhancing Recruitment Using Teleconference and Commitment Contract (ERUTECC): a stepped wedge cluster randomised trial within the EFFECTS trial. Upsala Journal of Medical Sciences 2025;130:e12897. [DOI: 10.48101/ujms.v130.12897]

### Ives 2001 {published data only}

- Ives NJ, Troop M, Waters A, Davies S, Higgs C, Easterbrook PJ. Does an HIV clinical trial information booklet improve patient knowledge and understanding of HIV clinical trials? HIV Medicine 2001;2:241-9.

### Jefferson 2018 {published data only}

- Jefferson L, Fairhurst C, Brealey S, Coleman E, Cook L, Hewitt C, et al. Remote or on-site visits were feasible for the initial setup meetings with hospitals in a multicenter surgical trial: an embedded randomized trial. Journal of Clinical Epidemiology 2018;100:13-21. [DOI: 10.1016/j.jclinepi.2018.04.011]

### Jennings 2015a {published data only}

- Jennings CG, MacDonald TM, Wei L, Brown MJ, McConnachie L, Mackenzie IS. Does offering an incentive payment improve recruitment to clinical trials and increase the proportion of socially deprived and elderly participants? Trials 2015;16:80. [DOI: 10.1186/s13063-015-0582-8]

### Jennings 2015b {published data only}

- Jennings CG, MacDonald TM, Wei L, Brown MJ, McConnachie L, Mackenzie IS. Does offering an incentive payment improve recruitment to clinical trials and increase the proportion of socially deprived and elderly participants? Trials 2015;16:80. [DOI: 10.1186/s13063-015-0582-8]

### Jennings 2015c {published data only}

- Jennings CG, MacDonald TM, Wei L, Brown MJ, McConnachie L, Mackenzie IS. Does offering an incentive payment improve recruitment to clinical trials and increase the proportion of socially deprived and elderly participants? Trials 2015;16:80. [DOI: 10.1186/s13063-015-0582-8]

### Jennings 2015d {published data only}

- Jennings CG, MacDonald TM, Wei L, Brown MJ, McConnachie L, Mackenzie IS. Does offering an incentive payment improve recruitment to clinical trials and increase the proportion of socially deprived and elderly participants? Trials 2015;16:80. [DOI: 10.1186/s13063-015-0582-8]

### Jennings 2015e {published data only}

- Jennings CG, MacDonald TM, Wei L, Brown MJ, McConnachie L, Mackenzie IS. Does offering an incentive payment improve recruitment to clinical trials and increase the proportion of socially deprived and elderly participants? Trials 2015;16:80. [DOI: 10.1186/s13063-015-0582-8]

### Jolly 2019 (START MMI PSM-COPD) {published data only}

- Jolly K, Sidhu M, Bower P, Madurasinghe V; MRC START Group; PSM COPD Group, et al. Improving recruitment to a study of telehealth management for COPD: a cluster randomised controlled ‘study within a trial’ (SWAT) of a multimedia information resource. Trials 2019;20:453. [DOI: 10.1186/s13063-019-3496-z]

### Kamen 2018 {published data only}

- Kamen CS, Quinn GP, Asare M, Heckler CE, Guido JJ, Giguere JK, et al. Multimediapsychoeducation for patients with cancer who are eligible for clinical trials: a randomized clinical trial. Cancer 2018;124(23):4504-11. [DOI: 10.1002/cncr.31771]

### Kendrick 2001 {published data only}

- Kendrick D, Watson M, Dewey M, Woods AJ. Does sending a home safety questionnaire increase recruitment to an injury prevention trial? A randomised controlled trial. Journal of Epidemiology and Community Health 2001;55:845-6.

### Kimmick 2005 {published data only}

- Kimmick GG, Peterson BL, Kornblith AB, Mandelblatt J, Johnson JL, Wheeler J, et al. Improving accrual of older persons to cancer treatment trials: a randomized trial comparing an educational intervention with standard information: CALGB 360001. Journal of Clinical Oncology 2005;23(10):2201-7.

### Knapp 2020 (START PIS CASPER) {published data only}

- Knapp P, Gilbody S, Holt J, Keding A, Mitchell N, Raynor DK, et al. Optimised patient information materials and recruitment to a study of behavioural activation in older adults: an embedded study within a trial. F1000Research 2020;9:417. [DOI: 10.12688/f1000research.24051.1]

### Knapp 2023a (BALANCE) {published data only}

- Knapp P, Martin-Kerry J, Sheridan R, Coleman E, Roche J, Moe-Byrne T, et al. The effectiveness and acceptability of multimedia information when recruiting children and young people to trials: the TRECA meta-analysis of SWATs. Health and Social Care Delivery Research 2023;11(24):1-112. [DOI: 10.3310/HTPM3841]

### Knapp 2023b (BAMP) {published data only}

- Knapp P, Martin-Kerry J, Sheridan R, Coleman E, Roche J, Moe-Byrne T, et al. The effectiveness and acceptability of multimedia information when recruiting children and young people to trials: the TRECA meta-analysis of SWATs. Health and Social Care Delivery Research 2023;11(24):1-112. [DOI: 10.3310/HTPM3841]

### Knapp 2023c (CHAMP-UK) {published data only}

- Knapp P, Martin-Kerry J, Sheridan R, Coleman E, Roche J, Moe-Byrne T, et al. The effectiveness and acceptability of multimedia information when recruiting children and young people to trials: the TRECA meta-analysis of SWATs. Health and Social Care Delivery Research 2023;11(24):1-112. [DOI: 10.3310/HTPM3841]

### Knapp 2023d (FORCE) {published data only}

- Knapp P, Martin-Kerry J, Sheridan R, Coleman E, Roche J, Moe-Byrne T, et al. The effectiveness and acceptability of multimedia information when recruiting children and young people to trials: the TRECA meta-analysis of SWATs. Health and Social Care Delivery Research 2023;11(24):1-112. [DOI: 10.3310/HTPM3841]

### Knapp 2023e (Thermic-3) {published data only}

- Knapp P, Martin-Kerry J, Sheridan R, Coleman E, Roche J, Moe-Byrne T, et al. The effectiveness and acceptability of multimedia information when recruiting children and young people to trials: the TRECA meta-analysis of SWATs. Health and Social Care Delivery Research 2023;11(24):1-112. [DOI: 10.3310/HTPM3841]

### Knapp 2023f (UKALL) {published data only}

- Knapp P, Martin-Kerry J, Sheridan R, Coleman E, Roche J, Moe-Byrne T, et al. The effectiveness and acceptability of multimedia information when recruiting children and young people to trials: the TRECA meta-analysis of SWATs. Health and Social Care Delivery Research 2023;11(24):1-112. [DOI: 10.3310/HTPM3841]

### Larkey 2002 {published data only}

- Larkey LK, Staten LK, Ritenbaugh C, Hall RA, Buller DB, Bassford T, et al. Recruitment of Hispanic women to the Women's Health Initiative: the case of *Embajadoras* in Arizona. Controlled Clinical Trials 2002;23:289-98.

### Lee 2017 {published data only}

- Lee H, Hubscher M, Moseley GL, Kamper SJ, Traeger AC, Skinner IW, et al. An embedded randomised controlled trial of a teaser campaign to optimise recruitment in primary care. Clinical Trials (London, England) 2017;14:162-9. [DOI: 10.1177/1740774516683921]

### Liénard 2006 {published data only}

- Liénard J-L, Quinax E, Fabre-Guillevin E, Piedbois P, Jouhaud A, Decoster G, et al. Impact of on-site initiation visits on patient recruitment and data quality in a randomized trial of adjuvant chemotherapy for breast cancer. Clinical Trials (London, England) 2006;3:486-92.

### Lipstein 2021 {published data only}

- Lipstein EA, Breslin M, Dodds CM, Kappelman MD, Ollberding NJ, Margolis P, et al. Integrating shared decision making into trial consent: a nested, cluster-randomized trial. Patient Education and Counseling 2021;104(7):1575-82. [DOI: 10.1016/j.pec.2020.12.018]

### Litchfield 2005 {published data only}

- Litchfield J, Freeman J, Schou H, Elsley M, Fuller R, Chubb B. Is the future for clinical trials internet-based? A cluster randomized clinical trial. Clinical Trials (London, England) 2005;2:72-9.

### Madurasinghe 2021 (START PIS HeLP Diabetes) {published data only}

- Madurasinghe VW, Bower P, Eldridge S, Collier D, Graffy J, Treweek S, et al. Can we achieve better recruitment by providing better information? Meta-analysis of ‘studies within a trial’ (SWATs) of optimised participant information sheets. BMC Medicine 2021;19:1-8. [DOI: 10.1186/s12916-021-02086-2]

### Madurasinghe 2023a (START MMI GHT2000a) {published data only}

- Madurasinghe VW, Knapp P, Eldridge S, Collier D, Treweek S, Rick J, et al. Can we achieve better trial recruitment by presenting patient information through multimedia? Meta-analysis of ‘studies within a trial’ (SWATs). BMC Medicine 2023;21(1):425. [DOI: 10.1186/s12916-023-03081-5]

### Madurasinghe 2023b (START MMI GHT2000b) {published data only}

- Madurasinghe VW, Knapp P, Eldridge S, Collier D, Treweek S, Rick J, et al. Can we achieve better trial recruitment by presenting patient information through multimedia? Meta-analysis of ‘studies within a trial’ (SWATs). BMC Medicine 2023;21(1):425. [DOI: 10.1186/s12916-023-03081-5]

### Madurasinghe 2023c (START MMI HI-Light) {published data only}

- Madurasinghe VW, Knapp P, Eldridge S, Collier D, Treweek S, Rick J, et al. Can we achieve better trial recruitment by presenting patient information through multimedia? Meta-analysis of ‘studies within a trial’(SWATs). BMC Medicine 2023;21(1):425. [DOI: 10.1186/s12916-023-03081-5]

### Madurasinghe 2023d (START MMI seAFOod) {published data only}

- Madurasinghe VW, Knapp P, Eldridge S, Collier D, Treweek S, Rick J, et al. Can we achieve better trial recruitment by presenting patient information through multimedia? Meta-analysis of ‘studies within a trial’(SWATs). BMC Medicine 2023;21(1):425. [DOI: 10.1186/s12916-023-03081-5]

### Man 2015 (START PIS Healthlines Depression) {published data only}

- Man MS, on behalf of the Healthlines Study Group; Rick J, Bower P, on behalf of the MRC-START Group. Improving recruitment to a study of telehealth management for long-term conditions in primary care: two embedded, randomised controlled trials of optimised patient information materials. Trials 2015;16:309. [DOI: 10.1186/s13063-015-0820-0]

### Mattock 2020 {published data only}

- Mattock HC, Ryan R, O’Farrelly C, Babalis D, Ramchandani PG. Does a video clip enhance recruitment into a parenting trial? Learnings from a study within a trial. Trials 2020;21:1-2. [DOI: 10.1186/s13063-020-04779-0]

### Maxwell 2017 {published data only}

- Maxwell AE, Parker RA, Drever J, Rudd A, Dennis MS, Weir CJ, et al. Promoting Recruitment using Information Management Efficiently (PRIME): a stepped-wedge, cluster randomised trial of a complex recruitment intervention embedded within the REstart or Stop Antithrombotics Randomised Trial. Trials 2017;18:1-2. [DOI: 10.1186/s13063-017-2355-z]

### McCaffery 2019 {published data only}

- McCaffery J, Mitchell A, Fairhurst C, Cockayne S, Rodgers S, Relton C, et al; OTIS Study Team. Does handwriting the name of a potential trial participant on an invitation letter improve recruitment rates? A randomised controlled study within a trial. F1000Research 2019;8:659. [DOI: 10.12688/f1000research.18939.1]

### Miller 2021a {published data only}

- Miller HN, Plante TB, Gleason KT, Charleston J, Mitchell CM, Miller III ER, et al. A/B design testing of a clinical trial recruitment website: a pilot study to enhance the enrollment of older adults. Contemporary Clinical Trials 2021;111:106598. [DOI: 10.1016/j.cct.2021.106598]

### Miller 2021b {published data only}

- Miller HN, Plante TB, Gleason KT, Charleston J, Mitchell CM, Miller III ER, et al. A/B design testing of a clinical trial recruitment website: a pilot study to enhance the enrollment of older adults. Contemporary Clinical Trials 2021;111:106598. [DOI: 10.1016/j.cct.2021.106598]

### Monaghan 2007 {published data only}

- Monaghan H, Richens A, Colman S, Currie R, Girgis S, Jayne K, et al. A randomised trial of the effects of an additional communication strategy on recruitment into a large-scale, multi-centre trial. Contemporary Clinical Trials 2007;28:1-5.

### Nystuen 2004 {published data only}

- Nystuen P, Hagen KB. Telephone reminders are effective in recruiting nonresponding patients to randomized controlled trials. Journal of Clinical Epidemiology 2004;57:773-6.

### Paris 2015 {published data only}

- Paris A, Deygas B, Cornu C, Thalamas C, Maison P, Duale C, et al. Improved informed consent documents for biomedical research do not increase patients’ understanding but reduce enrolment: a study in real settings. British Journal of Clinical Pharmacology 2015;80(5):1010-20. [DOI: 10.1111/bcp.12716]

### Parker 2018 (START PIS ECLS) {published data only}

- Parker A, Knapp P, Treweek S, Madhurasinghe V, Littleford R, Gallant S, et al. The effect of optimised patient information materials on recruitment in a lung cancer screening trial: an embedded randomised recruitment trial. Trials 2018;19:1-8. [DOI: 10.1186/s13063-018-2896-9]

### Paul 2011 {published data only}

- Paul J, Iveson T, Midgely R, Harkin A, Masterton M, Alexander L, et al. Choice of randomisation time-point in non-inferiority studies of reduced treatment duration: experience from the SCOT study. Trials 2011;12:A30.

### Paul 2014 {published data only}

- Paul C, Courtney R, Sanson-Fisher R, Carey M, Hill D, Simmons J, et al. A randomized controlled trial of the effectiveness of a pre-recruitment primer letter to increase participation in a study of colorectal screening and surveillance. BMC Medical Research Methodology 2014;14:44. [DOI: 10.1186/1471-2288-14-44]

### Rogers 2019 {published data only}

- Rogers A, Flynn RW, Mackenzie IS, MacDonald TM. Does the provision of a DVD-based audio-visual presentation improve recruitment in a clinical trial? A randomised trial of DVD trial invitations. BMC Medical Research Methodology 2019;19:1-6. [DOI: 10.1186/s12874-019-0663-6]

### Saif 2020 {published data only}

- Saif N, Berkowitz C, Tripathi S, Scheyer O, Caesar E, Hristov H, et al. Effectiveness of online education for recruitment to an Alzheimer's disease prevention clinical trial. Alzheimer's & Dementia: Translational Research & Clinical Interventions 2020;6(1):e12006. [DOI: 10.1002/trc2.12006]

### Sheridan 2020 (START PIS ISDR) {published data only}

- Sheridan R, Knapp P, Bower P, Madurasinghe V, Broadbent D, Awoyale L, et al; ISDR Trial Group. Patient recruitment to a diabetic retinopathy screening trial through optimised patient information materials: an embedded study within a trial (SWAT). F1000Research 2020;9:779. [DOI: 10.12688/f1000research.24938.1]

### Simes 1986 {published data only}

- Simes RJ, Tattersall MH, Coates AS, Raghavan D, Solomon HJ. Randomised comparison of procedures for obtaining informed consent in clinical trials of treatment for cancer. BMJ 1986;293:1065-8.

### Skinner 2019 {published data only}

- Skinner JS, Fair AM, Holman AS, Boyer AP, Wilkins CH. The impact of an educational video on clinical trial enrollment and knowledge in ethnic minorities: a randomized control trial. Frontiers in Public Health 2019;7:104. [DOI: 10.3389/fpubh.2019.00104]

### Sundaresan 2017 {published data only}

- Sundaresan P, Ager B, Turner S, Costa D, Kneebone A, Pearse M, et al. A randomised controlled trial evaluating the utility of a patient Decision Aid to improve clinical trial (RAVES 08.03) related decision-making. Radiotherapy and Oncology 2017;125(1):124-9. [DOI: 10.1016/j.radonc.2017.08.013]

### Tilley 2012 {published data only}

- Tilley BC, Mainous AG, Elm JJ, Pickelsimer E, Soderstrom LH, Ford ME, et al. A randomized recruitment intervention trial in Parkinson's disease to increase participant diversity: early stopping for lack of efficacy. Clinical Trials (London, England) 2012;9:188-97.

### Tilley 2021 {published data only}

- Tilley BC, Mainous AG 3rd, Amorrortu RP, McKee MD, Smith DW, Li R, et al. Using increased trust in medical researchers to increase minority recruitment: the RECRUIT cluster randomized clinical trial. Contemporary Clinical Trials 2021;109:106519. [DOI: 10.1016/j.cct.2021.106519]

### Trevena 2006 {published data only}

- Trevena L, Irwig L, Barratt A. Impact of privacy legislation on the number and characteristics of people who are recruited for research: a randomised controlled trial. Journal of Medical Ethics 2006;32:473-7.

### Treweek 2012 {published data only}

- Treweek S, Barnett K, Maclennan G, Bonetti D, Eccles MP, Francis JJ, et al. E-mail invitations to general practitioners were as effective as postal invitations and were more efficient. Journal of Clinical Epidemiology 2012;65:793-7. [DOI: 10.1016/j.jclinepi.2011.11.010]

### Veerus 2016 {published data only}

- Veerus P, Fischer K, Hemminki E, Hovi SL, Hakama M. Effect of characteristics of women on attendance in blind and non-blind randomised trials: analysis of recruitment data from the EPHT Trial. BMJ Open 2016;6(10):e011099. [DOI: 10.1136/bmjopen-2016-011099]

### Wadland 1990 {published data only}

- Wadland WC, Hughes JR, Secker-Walker RH, Bronson DL, Fenwick J. Recruitment in a primary care trial on smoking cessation. Family Medicine 1990;22:201-4.

### White 2023 {published data only}

- White D, Sutton L, Mooney C, Dawson J, Burton C; PROMETHEUS Group. SWAT 137: a 2x2 randomised factorial SWAT of the use of a pen and brief information leaflet to improve recruitment in a randomised controlled trial of a community-based clinic for patients with persistent physical symptoms. F1000Research 2023;12:1136. [DOI: 10.12688/f1000research.130154.1]

### Whiteside 2019 {published data only}

- Whiteside K, Flett L, Mitchell A, Fairhurst C, Cockayne S, Rodgers S, et al; OTIS Study Group. Using pens as an incentive for trial recruitment of older adults: an embedded randomised controlled trial. F1000Research 2019;8:315. [DOI: 10.12688/f1000research.18300.1]

### Witham 2018 {published data only}

- Witham MD, Band MM, Price RJ, Fulton RL, Clarke CL, Donnan PT, et al. Effect of two different participant information sheets on recruitment to a falls trial: an embedded randomised recruitment trial. Clinical Trials (London, England) 2018;15(6):551-6. [DOI: 10.1177/1740774518803558]

### Wong 2013 {published data only}

- Wong AD, Kirby J, Guyatt GH, Moayyedi P, Vora P, You JJ. Randomized controlled trial comparing telephone and mail follow-up for recruitment of participants into a clinical trial of colorectal cancer screening. Trials 2013;14:40. [DOI: 10.1186/1745-6215-14-40]
