# Supplementary material for: Strategies to improve recruitment to randomised trials
Source: Cochrane Database Syst Rev. 2026 Sep 15;2026(9):MR000013. doi: 10.1002/14651858.MR000013.pub7 (PMC13576036; doi:10.1002/14651858.MR000013.pub7)
Supplement: Supplementary file 3 [file MR000013-SUP-03-characteristicsOfExcludedStudies.html]

Characteristics of excluded studies


# Supplementary material 3 to: Strategies to improve recruitment to randomised trials

Parker A, Mongelli G, Piccolo-Lawrance C, Coleman E, Gkekas A, Wang HI, Dawson S, Green H, Way R, Rai A, Bruhn H, Sullivan F, Boxall C, Chan M, Manson P, Hewitt C, Fairhurst C, Clarke M, Treweek S
  
https://doi.org/10.1002/14651858.MR000013.pub7

The material in this section has been supplied by the author(s) for publication under a Licence for Publication and the author(s) are solely responsible for the material. Cochrane has reviewed this material, but Cochrane has not copyedited, formatted or proofread. Cochrane accordingly gives no representations or warranties of any kind in relation to, and accepts no liability for any reliance on or use of, such material.

Back to top

# Characteristics of excluded studies

## Table of contents

- Studies ordered by Study ID
  - Abhyankar 2010
  - Addissie 2016
  - Agni 2019
  - Annett 2017
  - Arana-Chicas 2022
  - Aysola 2018
  - Bachour 2017
  - Bardach 2021
  - Bauer 2018
  - Benoit-Piau 2020
  - Bentley 2004
  - Bickmore 2016
  - Bishop 2019
  - Blumenberg 2019
  - Bradford-Rogers 2022
  - Cornelius 2018
  - Cottler 2017
  - Crosbie 2022
  - Cunningham-Erves 2022
  - D'Angio 2017
  - Darden 2022
  - Diguiseppi 2006
  - Donohue 2016
  - Donovan 2010
  - Duane 2020
  - Ellis 2002
  - Faro 2021
  - Fleissig 2001
  - Freer 2009
  - Frew 2015
  - Furberg 2018
  - Fureman 1997
  - Gioia 2016
  - Godinho 2019
  - Graham 2007
  - Halpern 2004
  - Haynes 2019
  - Heijmans 2015
  - Hoffmeister 2017
  - Horowitz 2019
  - Huesch 2018
  - Jacobsen 2012
  - Jeste 2009
  - Juraschek 2018
  - Karunaratne 2010
  - Kass 2015
  - Kerr 2004
  - Koonrungsesomboon 2016
  - Krischer 2017
  - Krishnamurti 2016
  - Krutsinger 2019
  - Krutsinger 2020
  - Law 2022
  - Leavitt 2017
  - Lindley 2022
  - Llewellyn‐Thomas 1995a
  - Llewellyn‐Thomas 1995b
  - Lois 2023
  - MacQueen 2014
  - Man 2015b
  - Mandelblatt 2005
  - Martin-Kerry 2017
  - Massett 2017
  - Mastellos 2015
  - McCullough 2017
  - Miller 1999
  - Mitchell 2019
  - Mudano 2013
  - Murphy 2023
  - Myles 1999
  - Neighbors 2018
  - Neil 2021
  - Neil 2022
  - Nickell 2019
  - Nollett 2016
  - O'Hare 2018
  - O'Sullivan 2022
  - Ortiz 2019
  - Palmeirim 2020
  - Palmer 2018
  - Parker 2017a
  - Parker 2017b
  - Pawlak 2015
  - Peel 2019
  - Peng 2019
  - Perrone 1995
  - Peters 2022
  - Pighills 2009
  - Plante 2020
  - Realpe 2016
  - Rimel 2015
  - Robinson 2017
  - Shepherd 2022
  - Simel 1991
  - Simmons 2022
  - So 2018
  - Stephens 2022
  - Sygna 2015
  - Tattersall 2017
  - Tehranisa 2014
  - Thilsing 2021
  - Tilley 2017
  - Treschan 2003
  - Uveges 2018
  - Valentine 2020
  - Villanti 2015
  - Wallace 2021
  - Warner 2021
  - Warnock 2022
  - Weinfurt 2008a
  - Weinfurt 2008b
  - Wells 2013
  - Welton 1999
  - Weston 1997
  - Winhusen 2020
  - Won 2022
  - Woodford 2020
  - Yeary 2018
- Footnotes
- References to studies

## Studies ordered by Study ID

| Study | Reason for exclusion |
| --- | --- |
| Abhyankar 2010 | Hypothetical SWAT |
| Addissie 2016 | Not recruiting to a randomised trial |
| Agni 2019 | Protocol |
| Annett 2017 | Not reporting recruitment results |
| Arana-Chicas 2022 | No randomisation or Quasi randomised SWAT/trial |
| Aysola 2018 | Not recruiting to a randomised trial |
| Bachour 2017 | No randomisation |
| Bardach 2021 | No randomisation |
| Bauer 2018 | Not recruiting to a randomised trial |
| Benoit-Piau 2020 | No randomisation or Quasi randomised SWAT/trial |
| Bentley 2004 | Hypothetical SWAT |
| Bickmore 2016 | Not recruiting to a randomised trial |
| Bishop 2019 | Hypothetical trial/SWAT |
| Blumenberg 2019 | Not recruiting to a randomised trial |
| Bradford-Rogers 2022 | No randomisation or Quasi randomised SWAT/trial |
| Cornelius 2018 | Not recruiting to a randomised trial |
| Cottler 2017 | Not recruiting to a randomised trial |
| Crosbie 2022 | No randomisation or Quasi randomised SWAT/trial |
| Cunningham-Erves 2022 | No randomisation or Quasi randomised SWAT/trial |
| D'Angio 2017 | Not reporting recruitment results |
| Darden 2022 | No randomisation or Quasi randomised SWAT/trial |
| Diguiseppi 2006 | Examining hypothetical recruitment |
| Donohue 2016 | Not recruiting to a randomised trial |
| Donovan 2010 | Allocation not randomised |
| Duane 2020 | Protocol |
| Ellis 2002 | Hypothetical SWAT |
| Faro 2021 | No randomisation or Quasi randomised SWAT/trial |
| Fleissig 2001 | Quasi-randomised |
| Freer 2009 | Hypothetical SWAT |
| Frew 2015 | Not recruiting to a randomised trial |
| Furberg 2018 | Protocol |
| Fureman 1997 | Hypothetical SWAT |
| Gioia 2016 | No randomisation |
| Godinho 2019 | No randomisation or Quasi randomised SWAT/trial |
| Graham 2007 | Hypothetical SWAT |
| Halpern 2004 | Hypothetical SWAT |
| Haynes 2019 | Not reporting recruitment results |
| Heijmans 2015 | Not recruiting to a randomised trial |
| Hoffmeister 2017 | Not recruiting to a randomised trial |
| Horowitz 2019 | No randomisation or Quasi randomised SWAT/trial |
| Huesch 2018 | No randomisation or Quasi randomised SWAT/trial |
| Jacobsen 2012 | Examining hypothetical recruitment |
| Jeste 2009 | Examining hypothetical recruitment |
| Juraschek 2018 | Not reporting recruitment results |
| Karunaratne 2010 | Hypothetical SWAT |
| Kass 2015 | Not reporting recruitment results |
| Kerr 2004 | Hypothetical SWAT |
| Koonrungsesomboon 2016 | No randomisation or Quasi randomised SWAT/trial |
| Krischer 2017 | No randomisation or Quasi randomised SWAT/trial |
| Krishnamurti 2016 | Not recruiting to a randomised trial |
| Krutsinger 2019 | Not recruiting to a randomised trial |
| Krutsinger 2020 | Hypothetical trial/SWAT |
| Law 2022 | No randomisation or Quasi randomised SWAT/trial |
| Leavitt 2017 | No randomisation or Quasi randomised SWAT/trial |
| Lindley 2022 | No randomisation or Quasi randomised SWAT/trial |
| Llewellyn‐Thomas 1995a | Hypothetical SWAT |
| Llewellyn‐Thomas 1995b | Hypothetical SWAT |
| Lois 2023 | No randomisation or Quasi randomised SWAT/trial |
| MacQueen 2014 | Examining hypothetical recruitment |
| Man 2015b | Examining hypothetical recruitment |
| Mandelblatt 2005 | Examining hypothetical recruitment |
| Martin-Kerry 2017 | Protocol |
| Massett 2017 | Hypothetical trial/SWAT |
| Mastellos 2015 | Protocol |
| McCullough 2017 | No randomisation or Quasi randomised SWAT/trial |
| Miller 1999 | Was a quasi-randomised evaluation |
| Mitchell 2019 | No randomisation or Quasi randomised SWAT/trial |
| Mudano 2013 | Examining hypothetical recruitment |
| Murphy 2023 | No randomisation or Quasi randomised SWAT/trial |
| Myles 1999 | Examining hypothetical recruitment |
| Neighbors 2018 | Not recruiting to a randomised trial |
| Neil 2021 | Host trial not actively recruiting, therefore not assessing trial recruitment |
| Neil 2022 | Host trial not actively recruiting, therefore not assessing trial recruitment |
| Nickell 2019 | Not reporting recruitment results |
| Nollett 2016 | Protocol |
| O'Hare 2018 | No randomisation or Quasi randomised SWAT/trial |
| O'Sullivan 2022 | Not reporting recruitment results |
| Ortiz 2019 | Not reporting recruitment results |
| Palmeirim 2020 | No randomisation or Quasi randomised SWAT/trial |
| Palmer 2018 | Hypothetical trial/SWAT |
| Parker 2017a | Assessing incentives for clinitians to recruit to RCTs, which is captured by a separate Cochrane review |
| Parker 2017b | Not reporting recruitment results |
| Pawlak 2015 | Not recruiting to a randomised trial |
| Peel 2019 | No randomisation or Quasi randomised SWAT/trial |
| Peng 2019 | Not reporting recruitment results |
| Perrone 1995 | Hypothetical SWAT |
| Peters 2022 | No randomisation or Quasi randomised SWAT/trial |
| Pighills 2009 | Quasi-randomised |
| Plante 2020 | No randomisation or Quasi randomised SWAT/trial |
| Realpe 2016 | No randomisation or Quasi randomised SWAT/trial |
| Rimel 2015 | No randomisation or Quasi randomised SWAT/trial |
| Robinson 2017 | No randomisation or Quasi randomised SWAT/trial |
| Shepherd 2022 | Protocol |
| Simel 1991 | Hypothetical SWAT |
| Simmons 2022 | No randomisation or Quasi randomised SWAT/trial |
| So 2018 | Not recruiting to a randomised trial |
| Stephens 2022 | No randomisation or Quasi randomised SWAT/trial |
| Sygna 2015 | No randomisation or Quasi randomised SWAT/trial |
| Tattersall 2017 | Not reporting recruitment results |
| Tehranisa 2014 | Examining hypothetical recruitment |
| Thilsing 2021 | Not recruiting to a randomised trial |
| Tilley 2017 | Not reporting recruitment results |
| Treschan 2003 | Hypothetical SWAT |
| Uveges 2018 | Protocol |
| Valentine 2020 | Not recruiting to a randomised trial |
| Villanti 2015 | Not recruiting to a randomised trial |
| Wallace 2021 | No randomisation or Quasi randomised SWAT/trial |
| Warner 2021 | No randomisation or Quasi randomised SWAT/trial |
| Warnock 2022 | Host trial not actively recruiting, therefore not assessing trial recruitment |
| Weinfurt 2008a | Examining hypothetical recruitment |
| Weinfurt 2008b | Examining hypothetical recruitment |
| Wells 2013 | Examining hypothetical recruitment |
| Welton 1999 | Examining hypothetical recruitment |
| Weston 1997 | Examining hypothetical recruitment |
| Winhusen 2020 | Not recruiting to a randomised trial |
| Won 2022 | Not reporting recruitment results |
| Woodford 2020 | Protocol |
| Yeary 2018 | Not recruiting to a randomised trial |

## Footnotes

Most studies that we considered in detail but excluded arose from records that we had retrieved because the database reference gave no abstract and it was not possible to exclude them on the basis of the title. We excluded most of the records falling into this category as soon as we checked the full text, with the most common reason being that the study did not evaluate a recruitment intervention.

The two exceptions are Aaronson 1996 and Kiernan 2000, which we excluded at the data extraction stage for the reasons given in the table.

## References to studies

### Abhyankar 2010 {published data only}

- Abhyankar P, Bekker HL, Summers BA, Velikova G. Why values elicitation techniques enable people to make informed decisions about cancer trial participation. Health Expectations 2010;14(Suppl 1):20-32. 2010;14(Suppl 1):20-32. [DOI: 10.1111/j.1369-7625.2010.00615.x]

### Addissie 2016 {published data only}

- Addissie A, Abay S, Feleke Y, Newport M, Farsides B, Davey G. Cluster randomized trial assessing the effects of rapid ethical assessment on informed consent comprehension in a low-resource setting. BMC Medical Ethics 2016;17(1):40. [DOI: 10.1186/s12910-016-0127-z]

### Agni 2019 {published data only}

- Agni N, Fairhurst C, McDaid C, Reed M, Torgerson D. Protocol for a factorial randomised controlled trial, embedded within WHiTE 8 COPAL, of an Enhanced Trainee Principal Investigator Package and Additional Digital Nudge to increase recruitment rates. F1000Research 2019;8:1153. [DOI: 10.12688/f1000research.19743.1]

### Annett 2017 {published data only}

- Annett RD, Brody JL, Scherer DG, Turner CW, Dalen J, Raissy H. A randomized study of a method for optimizing adolescent assent to biomedical research. AJOB Empirical Bioethics 2017;8(3):189-97. [DOI: 10.1080/23294515.2016.1251507]

### Arana-Chicas 2022 {published data only}

- Arana-Chicas E, Cartujano-Barrera F, Rieth KK, Richter KK, Ellerbeck EF, Cox LS, et al. Effectiveness of recruitment strategies of Latino smokers: secondary analysis of a mobile health smoking cessation randomized clinical trial. Journal of Medical Internet Research 2022;24(6):e34863. [DOI: 10.2196/34863]

### Aysola 2018 {published data only}

- Aysola J, Tahirovic E, Troxel AB, Asch DA, Gangemi K, Hodlofski AT, et al. A randomized controlled trial of opt-in versus opt-out enrollment into a diabetes behavioral intervention. American Journal of Health Promotion 2018;32(3):745-52. [DOI: 10.1177/0890117116671673]

### Bachour 2017 {published data only}

- Bachour CC, Bachmann GA, Foster DC, Wan JY, Rawlinson LA, Brown CS; Gabapentin Study Group (GABAgroup). Recruitment methods in a clinical trial of provoked vulvodynia: predictors of enrollment. Clinical Trials (London, England) 2017;14(1):103-8. [DOI: 10.1177/1740774516663461]

### Bardach 2021 {published data only}

- Bardach NS, Lam R, Jasik CB. Assessment of automated clinical trial recruitment and enrolment using patient-facing technology. BMJ Health & Care Informatics 2021;28(1):e100076. [DOI: 10.1136/bmjhci-2019-100076]

### Bauer 2018 {published data only}

- Bauer A, Riemann JF, Seufferlein T, Reinshagen M, Hollerbach S, Haug U, et al. Invitation to screening colonoscopy in the population at familial risk for colorectal cancer: a cluster-randomized study aimed at increasing participation rates. Deutsches Ärzteblatt International 2018;115(43):715. [DOI: 10.3238/arztebl.2018.0715]

### Benoit-Piau 2020 {published data only}

- Benoit-Piau J, Dumoulin C, Carroll MS, Mayrand MH, Bergeron S, Khalifé S, et al; cost: e-recruitment is a promising method in gynecological trials. e-recruitment is a promising method in gynecological trials. Journal of Sexual Medicine 2020;17(7):1304-11. [DOI: 10.1016/j.jsxm.2020.04.005]

### Bentley 2004 {published data only}

- Bentley JP, Thacker PG. The influences of risk and monetary payment on the research participation decision making process. Journal of Medical Ethics 2004;30(3):293‐8. [DOI: 10.1136/jme.2002.001594]

### Bickmore 2016 {published data only}

- Bickmore TW, Utami D, Matsuyama R, Paasche-Orlow MK. Improving access to online health information with conversational agents: a randomized controlled experiment. Journal of Medical Internet Research 2016;18(1):e1. [DOI: 10.2196/jmir.5239]

### Bishop 2019 {published data only}

- Bishop FL, Greville-Harris M, Bostock J, Din A, Graham CA, Lewith G, et al. Informing adults with back pain about placebo effects: randomized controlled evaluation of a new website with potential to improve informed consent in clinical research. Journal of Medical Internet Research 2019;21(1):10.2196/jmir.9955. [DOI: 10.2196/jmir.9955]

### Blumenberg 2019 {published data only}

- Blumenberg C, Menezes AM, Gonçalves H, Assunção MC, Wehrmeister FC, Barros AJ. How different online recruitment methods impact on recruitment rates for the web-based coortesnaweb project: a randomised trial. BMC Medical Research Methodology 2019;19(1):127. [DOI: 10.1186/s12874-019-0767-z]

### Bradford-Rogers 2022 {published data only}

- Bradford-Rogers J, Lopez-Matos J, Cain D, Lopez D, Starks TJ. Comparing the efficiency of online and field-based outreach for the recruitment of Black and Latino sexual minority men into an HIV prevention implementation trial. Prevention Science 2022;23(6):900-6. [DOI: 10.1007/s11121-022-01367-3]

### Cornelius 2018 {published data only}

- Cornelius VR, McDermott L, Forster AS, Ashworth M, Wright AJ, Gulliford MC. Automated recruitment and randomisation for an efficient randomised controlled trial in primary care. Trials 2018;19(1):341. [DOI: 10.1186/s13063-018-2723-3]

### Cottler 2017 {published data only}

- Cottler LB, Striley CW, Elliott AL, Zulich AE, Kwiatkowski E, Nelson DR. Pragmatic trial of a Study Navigator Model (NAU) vs. Ambassador Model (N+) to increase enrollment to health research among community members who use illicit drugs. Drug and Alcohol Dependence 2017;175:146-50. [DOI: 10.1016/j.drugalcdep.2016.12.031]

### Crosbie 2022 {published data only}

- Crosbie PA, Gabe R, Simmonds I, Hancock N, Alexandris P, Kennedy M, et al. Participation in community-based lung cancer screening: the Yorkshire Lung Screening Trial. European Respiratory Journal 2022;60(5):2200483. [DOI: 10.1183/13993003.00483-2022]

### Cunningham-Erves 2022 {published data only}

- Cunningham-Erves J, Kusnoor SV, Villalta-Gil V, Stallings SC, Ichimura JS, Israel TL, et al. Development and pilot implementation of guidelines for culturally tailored research recruitment materials for African Americans and Latinos. BMC Medical Research Methodology 2022;22(1):248. [DOI: 10.1186/s12874-022-01724-4]

### D'Angio 2017 {published data only}

- D’Angio CT, Wang H, Hunn JE, Pryhuber GS, Chess PR, Lakshminrusimha S. Permission form synopses to improve parents’ understanding of research: a randomized trial. Journal of Perinatology 2017;37(6):735-9. [DOI: 10.1038/jp.2017.26]

### Darden 2022 {published data only}

- Darden PM, Davis AM, Lee JY, Bimali M, Simon AE, Atz AM, et al. Active vs traditional methods of recruiting children for a clinical trial in rural primary care clinics: a cluster-randomized clinical trial. JAMA Network Open 2022;5(11):e2244040-e2244040. [DOI: 10.1001/jamanetworkopen.2022.44040]

### Diguiseppi 2006 {published data only}

- Diguiseppi C, Goss C, Xu S, Magid D, Graham A. Telephone screening for hazardous drinking among injured patients seen in acute care clinics: feasibility study. Alcohol & Alcoholism 2006;41(4):438-45.

### Donohue 2016 {published data only}

- Donohue B, Dowd A, Philips C, Plant CP, Loughran T, Gavrilova Y. Controlled evaluation of a method to assist recruitment of participants into treatment outcome research and engage student-athletes into substance abuse intervention. Journal of Clinical Sport Psychology 2016;10(4):272-88. [DOI: 10.1123/jcsp.2015-0022]

### Donovan 2010 {published data only}

- Donovan J, Lane A, Mills N, Neal D, Hamdy F; the ProtecT Study Group. Development and application of a complex intervention to improve recruitment to randomised controlled trials. Clinical Trials (London, England) 2010;7:469.

### Duane 2020 {published data only}

- Duane S, Vellinga A, Smith V, Tierney M, Beecher C, Burke M, et al. The effectiveness of digital multimedia presentation of trial information on recruitment and retention of patients: protocol for a study within a trial (SWAT). HRB Open Research 2020;3:10. [DOI: 10.12688/hrbopenres.12994.1]

### Ellis 2002 {published data only}

- Ellis PM, Butow PN, Tattersall MH. Informing breast cancer patients about clinical trials: a randomized clinical trial of an educational booklet. Annals of Oncology 2002;13(9):1414-23. [DOI: 10.1093/annonc/mdf255]

### Faro 2021 {published data only}

- Faro JM, Nagawa CS, Orvek EA, Smith BM, Blok AC, Houston TK, et al. Comparing recruitment strategies for a digital smoking cessation intervention: technology-assisted peer recruitment, social media, ResearchMatch, and smokefree. gov. Contemporary Clinical Trials 2021;103:106314. [DOI: 10.1016/j.cct.2021.106314]

### Fleissig 2001 {published data only}

- Fleissig A, Jenkins V, Fallowfield L. Results of an intervention study to improve communication about randomised clinical trials of cancer therapy. Euorpean Journal of Cancer 2001;37(3):322-31. [DOI: 10.1016/S0959-8049(00)00415-9]

### Freer 2009 {published data only}

- Freer Y, McIntosh N, Teunisse S, Kanwaljeet JS, Boyle EM. More information, less understanding: a randomized study on consent issues in neonatal research. Pediatrics 2009;123(5):1301-5. [DOI: 10.1542/peds.2007-3860]

### Frew 2015 {published data only}

- Frew PM, Omer SB, Parker K, Bolton M, Schamel J, Shapiro E, et al. Delivering a “dose of hope”: a faith-based program to increase older African Americans’ participation in clinical trials. JMIR Research Protocols 2015;4(2):e4072. [DOI: 10.2196/resprot.4072]

### Furberg 2018 {published data only}

- Furberg RD, Raspa M, Wheeler AC, McCormack LA, Bailey DB. A digital health app to assess decisional capacity to provide informed consent: protocol for a randomized controlled trial. JMIR Research Protocols 2018;7(11):e10360. [DOI: 10.2196/10360]

### Fureman 1997 {published data only}

- Fureman I, Meyers K, McLellan AT, Metzger D, Woody G. Evaluation of a video‐supplement to informed consent: injection drug users and preventive HIV vaccine efficacy trials. AIDS Education and Prevention 1997;9(4):330-41. [DOI: 10.1521/aeap.1997.9.4.330]

### Gioia 2016 {published data only}

- Gioia CJ, Sobell LC, Sobell MB, Agrawal S. Craigslist versus print newspaper advertising for recruiting research participants for alcohol studies: cost and participant characteristics. Addictive Behaviors 2016;54:24-32. [DOI: 10.1016/j.addbeh.2015.11.008]

### Godinho 2019 {published data only}

- Godinho A, Schell C, Cunningham JA. How one small text change in a study document can impact recruitment rates and follow-up completions. Internet Interventions 2019;18:100284. [DOI: 10.1016/j.invent.2019.100284]

### Graham 2007 {published data only}

- Graham A, Goss C, Xu S, Magid D, Diguiseppi C. Effect of using different modes to administer the AUDIT‐C on identification of hazardous drinking and acquiescence to trial participation among injured patients. Alcohol & Alcoholism 2007;42(5):423‐9. [DOI: 10.1093/alcalc/agl123]

### Halpern 2004 {published data only}

- Halpern SD, Karlawish JH, Casarett, D, Berlin JA, Asch DA. Empirical assessment of whether moderate payments are undue or unjust inducements for participation in clinical trials. Annals of Internal Medicine 2004;164(7):801-3. [DOI: 10.1001/archinte.164.7.801]

### Haynes 2019 {published data only}

- Haynes R, Chen F, Wincott E, Dayanandan R, Lay MJ, Parish S, et al. Investigating modifications to participant information materials to improve recruitment into a large randomized trial. Trials 2019;20(1):681. [DOI: 10.1186/s13063-019-3779-4]

### Heijmans 2015 {published data only}

- Heijmans N, van Lieshout J, Wensing M. Improving participation rates by providing choice of participation mode: two randomized controlled trials. BMC Medical Research Methodology 2015;15(1):29. [DOI: 10.1186/s12874-015-0021-2]

### Hoffmeister 2017 {published data only}

- Hoffmeister M, Holleczek B, Zwink N, Stock C, Stegmaier C, Brenner H. Screening for bowel cancer: increasing participation via personal invitation: a randomized intervention study. Deutsches Ärzteblatt International 2017;114(6):87. [DOI: 10.3238/arztebl.2017.0087]

### Horowitz 2019 {published data only}

- Horowitz CR, Sabin T, Ramos M, Richardson LD, Hauser D, Robinson M, et al. Successful recruitment and retention of diverse participants in a genomics clinical trial: a good invitation to a great party. Genetics in Medicine 2019;21(10):2364-70. [DOI: 10.1038/s41436-019-0498-x]

### Huesch 2018 {published data only}

- Huesch MD, Mukherjee D, Saunders EF. E-recruitment into a bipolar disorder trial using Facebook tailored advertising. Clinical Trials (London, England) 2018;15(5):522-3. [DOI: 10.1177/1740774518784018]

### Jacobsen 2012 {published data only}

- Jacobsen PB, Wells KJ, Meade CD, Quinn GP, Lee JH, Fulp WJ, et al. Effects of a brief multimedia psychoeducational intervention on the attitudes and interest of patients with cancer regarding clinical trial participation: a multicenter randomized controlled trial. Journal of Clinical Oncology 2012;30:2516-21. [DOI: 10.1200/JCO.2011.39.5186]

### Jeste 2009 {published data only}

- Jeste DV, Palmer BW, Golshan S, Eyler LT, Dunn LB, Meeks T, et al. Multimedia consent for research in people with schizophrenia and normal subjects: a randomized controlled trial. Schizophrenia Bulletin 2009;35:719-29.

### Juraschek 2018 {published data only}

- Juraschek SP, Plante TB, Charleston J, Miller ER, Yeh HC, Appel LJ, et al. Use of online recruitment strategies in a randomized trial of cancer survivors. Clinical Trials (London, England) 2018;15(2):130-8. [DOI: 10.1177/1740774517745829]

### Karunaratne 2010 {published data only}

- Karunaratne A, Koremann SG, Thomas SL, Myles PS, Komeraroff PA. Improving communication when seeking informed consent: a randomised controlled study of a computer‐based method for providing information to prospective clinical trial participants. Medical Journal of Australia 2010;192:388‐92. [DOI: 10.5694/j.1326-5377.2010.tb03561.x]

### Kass 2015 {published data only}

- Kass NE, Taylor HA, Ali J, Hallez K, Chaisson L. A pilot study of simple interventions to improve informed consent in clinical research: feasibility, approach, and results. Clinical Trials (London, England) 2015;12(1):54-66. [DOI: 10.1177/1740774514560831]

### Kerr 2004 {published data only}

- Kerr CE, Robinson EJ, Lilford RL, Edwards SL, Braunholtz DA, Stevens AJ. The impact of describing clinical trial treatments as new or standard. Patient Education and Counseling 2004;53(1):107‐13. [DOI: 10.1016/S0738-3991(03)00124-1]

### Koonrungsesomboon 2016 {published data only}

- Koonrungsesomboon N, Laothavorn J, Chokevivat V, Hirayama K, Karbwang J. SIDCER informed consent form: principles and a developmental guideline. Indian Journal of Medical Ethics 2016;1(2):83-6.

### Krischer 2017 {published data only}

- Krischer J, Cronholm PF, Burroughs C, McAlear CA, Borchin R, Easley E, et al. Experience with direct-to-patient recruitment for enrollment into a clinical trial in a rare disease: a web-based study. Journal of Medical Internet Research 2017;19(2):e50. [DOI: 10.2196/jmir.6798]

### Krishnamurti 2016 {published data only}

- Krishnamurti T, Argo N. A patient-centered approach to informed consent: results from a survey and randomized trial. Medical Decision Making 2016;36(6):726-40. [DOI: 10.1177/0272989X16636844]

### Krutsinger 2019 {published data only}

- Krutsinger DC, Yadav KN, Cooney E, Brooks S, Halpern SD, Courtright KR. A pilot randomized trial of five financial incentive strategies to increase study enrollment and retention rates. Contemporary Clinical Trials Communications 2019;15:100390. [DOI: 10.1016/j.conctc.2019.100390]

### Krutsinger 2020 {published data only}

- Krutsinger DC, O’Leary KL, Ellenberg SS, Cotner CE, Halpern SD, Courtright KR. A randomized controlled trial of behavioral nudges to improve enrollment in critical care trials. Annals of the American Thoracic Society 2020;17(9):1117-25. [DOI: 10.1513/AnnalsATS.202003-194OC]

### Law 2022 {published data only}

- Law ZK, Appleton JP, Scutt P, Roberts I, Al-Shahi Salman R, England TJ, et al. Brief consent methods enable rapid enrollment in acute stroke trial: results from the TICH-2 randomized controlled trial. Stroke 2022;53(4):1141-8. [DOI: 10.1161/STROKEAHA.121.035191]

### Leavitt 2017 {published data only}

- Leavitt L, Abroms L, Johnson P, Schindler-Ruwisch J, Bushar J, Singh I, et al. Recruiting pregnant smokers from Text4baby for a randomized controlled trial of Quit4baby. Translational Behavioral Medicine 2017;7(2):157-65. [DOI: 10.1007/s13142-016-0450-4]

### Lindley 2022 {published data only}

- Lindley RI, Kane I, Cohen G, Sandercock PA. Factors influencing the use of different methods of consent in a randomized acute stroke trial: the Third International Stroke Trial (IST-3). International Journal of Stroke 2022;17(5):553-8. [DOI: 10.1177/17474930211037123]

### Llewellyn‐Thomas 1995a {published data only}

- Llewellyn‐Thomas HA, McGreal MJ, Thiel EC. Presenting clinical trial information: a comparison of methods. Patient Education and Counseling 1995;25(2):97-107. [DOI: 10.1016/0738-3991(94)00705-Q]

### Llewellyn‐Thomas 1995b {published data only}

- Llewellyn‐Thomas HA, Thiel EC, Sem FW, Woermke DE. Presenting clinical trial information: a comparison of methods. Patient Education and Counseling 1995;25(2):97‐107. [DOI: 10.1016/0738-3991(94)00705-Q]

### Lois 2023 {published data only}

- Lois A, Kohler JE, Monsell SE, Pullar KM, Victory J, Odom SR, et al. A Video-Based Consent Tool: development and Effect of Risk–Benefit Framing on Intention to Randomize. Journal of Surgical Research 2023;283:357-67. [DOI: 10.1016/j.jss.2022.10.089]

### MacQueen 2014 {published data only}

- MacQueen KM, Chen M, Ramirez C, Nnko SE, Earp KM. Comparison of closed-ended, open-ended, and perceived informed consent comprehension measures for a mock HIV prevention trial among women in Tanzania. PLOS One 2013;9:e105720. [DOI: 10.1371/journal.pone.0105720]

### Man 2015b {published data only}

- Man MS, on behalf of the Healthlines Study Group, Rick J, Bower P, on behalf of the MRC-START Group. Improving recruitment to a study of telehealth management for long-term conditions in primary care: two embedded, randomised controlled trials of optimised patient information materials. Trials 2015;16:309. [DOI: 10.1186/s13063-015-0820-0]

### Mandelblatt 2005 {published data only}

- Mandelblatt J, Kaufmann E, Sheppard VB, Pomeroy J, Kavanaugh J, Canar J, et al. Breast cancer prevention in community clinics: will low-income Latina patients participate in clinical trials? Preventive Medicine 2005;40:611-8.

### Martin-Kerry 2017 {published data only}

- Martin-Kerry J, Bower P, Young B, Graffy J, Sheridan R, Watt I, et al. Developing and evaluating multimedia information resources to improve engagement of children, adolescents, and their parents with trials (TRECA study): study protocol for a series of linked randomised controlled trials. Trials 2017;18(1):265. [DOI: 10.1186/s13063-017-1962-z]

### Massett 2017 {published data only}

- Massett HA, Hiser M, Atkinson NL, Brittle C, Bailey R, Adler J, et al. A randomized controlled study comparing the national cancer institute’s original and revised consent form templates. IRB: Ethics & Human Research 2017;39(5):1-7.

### Mastellos 2015 {published data only}

- Mastellos N, Andreasson A, Huckvale K, Larsen M, Curcin V, Car J, et al. A cluster randomised controlled trial evaluating the effectiveness of eHealth-supported patient recruitment in primary care research: the TRANSFoRm study protocol. Implementation Science 2015;10(1):15. [DOI: 10.1186/s13012-015-0207-3]

### McCullough 2017 {published data only}

- McCullough MB, Janicke D, Odar Stough C, Robson S, Bolling C, Zion C, et al. Barriers to recruitment in pediatric obesity trials: comparing opt-in and opt-out recruitment approaches. Journal of Pediatric Psychology 2017;42(2):174-85. [DOI: 10.1093/jpepsy/jsw054]

### Miller 1999 {published data only}

- Miller NL, Markowitz JC, Kocsis JH, Leon AC, Brisco ST, Garno JL. Cost effectiveness of screening for clinical trials by research assistants versus senior investigators. Journal of Psychiatric Research 1999;33:81-5.

### Mitchell 2019 {published data only}

- Mitchell JW, Lammert S, Danh T, Horvath KJ. Optimizing enrollment methods: lessons learned from an mHealth, pilot HIV prevention randomized controlled trial with sexual minority men. International Journal of STD & AIDS 2019;03(7):703-6. [DOI: 10.1177/0956462419831854]

### Mudano 2013 {published data only}

- Mudano AS, Gary LC, Oliveira A, Wright MM, Curtis J, Delzell E, et al. Using tablet computers compared to interactive voice response to improve subject recruitment in osteoporosis pragmatic clinical trials: feasibility, satisfaction, and sample size. Patient Preference and Adherence 2013;7:517-23. [DOI: 10.2147/PPA.S44551]

### Murphy 2023 {published data only}

- Murphy AL, Turner JP, Rajda M, Allen KG, Pinter K, Gardner DM. Effectiveness and cost analysis of methods used to recruit older adult sedative users to a deprescribing randomized controlled trial during the COVID-19 pandemic. Exploratory Research in Clinical and Social Pharmacy 2023;9:100214. [DOI: 10.1016/j.rcsop.2022.100214]

### Myles 1999 {published data only}

- Myles PS, Fletcher HE, Cairo S, Madder H, McRae R, Cooper J, et al. Randomized trial of informed consent and recruitment for clinical trials in the immediate preoperative period. Anesthesiology 1999;91:969-78.

### Neighbors 2018 {published data only}

- Neighbors C, Rodriguez L, Garey L, Tomkins M. Testing a motivational model of delivery modality and incentives on participation in a brief alcohol intervention. Addictive behaviours 2018;84:131-8. [DOI: 10.1016/j.addbeh.2018.03.030]

### Neil 2021 {published data only}

- Neil JM, Chang Y, Goshe B, Rigotti N, Gonzalez I, Hawari S, et al. A web-based intervention to increase smokers’ intentions to participate in a cessation study offered at the point of lung screening: factorial randomized trial. JMIR Formative Research 2021;5(6):e28952. [DOI: 10.2196/28952]

### Neil 2022 {published data only}

- Neil JM, Senecal C, Ballini L, Chang Y, Goshe B, Flores E, et al. A multimethod evaluation of tobacco treatment trial recruitment messages for current smokers recently diagnosed with cancer: pilot factorial randomized controlled trial. JMIR Cancer 2022;8(3):e37526. [DOI: 10.2196/37526]

### Nickell 2019 {published data only}

- Nickell A, Stewart SL, Burke NJ, Guerra C, Cohen E, Lawlor C, et al. Engaging limited English proficient and ethnically diverse low-income women in health research: a randomized trial of a patient navigator intervention. Patient Education and Counseling 2019;102(7):1313-23. [DOI: 10.1016/j.pec.2019.02.013]

### Nollett 2016 {published data only}

- Nollett C, Kelson M, Hood K. Site visits to initiate recruitment in a clinical trial: does it matter who conducts the visit? Protocol for implementation in trials. Journal of Evidence-Based Medicine 2016;9(4):225-7. [DOI: 10.1111/jebm.12212]

### O'Hare 2018 {published data only}

- O'Hare F, Spark S, Flanagan Z, Heritier S, Curtis A, Zoungas S; STAREE Investigator Group. Impact of informed consent content and length on recruitment of older adults into a community based primary prevention trial. Contemporary Clinical Trials Communications 2018;11:89-94. [DOI: 10.1016/j.conctc.2018.05.003]

### O'Sullivan 2022 {published data only}

- O’Sullivan L, Savinelli S, O’Hare S, Holden S, McHugh C, Mallon P, et al. An enhanced participant information leaflet and multimedia intervention to improve the quality of informed consent to a randomised clinical trial enrolling people living with HIV and obesity: a protocol for a Study Within A Trial (SWAT). Trials 2022;23(1):50. [DOI: 10.1186/s13063-021-05979-y]

### Ortiz 2019 {published data only}

- Ortiz AP, Machin M, Soto-Salgado M, Centeno-Girona H, Rivera-Collazo D, González D, et al. Effect of an educational video to increase calls and screening into an anal cancer clinical trial among HIV+ Hispanics in PR: results from a randomized controlled behavioral trial. AIDS and Behavior 2019;23(5):1135-46. [DOI: 10.1007/s10461-018-2330-z]

### Palmeirim 2020 {published data only}

- Palmeirim MS, Ross A, Obrist B, Mohammed UA, Ame SM, Ali SM, et al. Informed consent procedure in a double blind randomized anthelminthic trial on Pemba Island, Tanzania: do pamphlet and information session increase caregivers knowledge? BMC Medical Ethics 2020;21(1):1. [DOI: 10.1186/s12910-019-0441-3]

### Palmer 2018 {published data only}

- Palmer BW, Harmell AL, Dunn LB, Kim SY, Pinto LL, Golshan S, et al. Multimedia aided consent for Alzheimer’s disease research. Clinical Gerontologist 2018;41(1):20-32. [DOI: 10.1080/07317115.2017.1373177]

### Parker 2017a {published data only}

- Parker C, Snyder R, Jefford M, Dilts D, Wolfe R, Millar J. A randomized controlled trial of an additional funding intervention to improve clinical trial enrollment. Journal of the National Comprehensive Cancer Network 2017;15(9):1104-10. [DOI: 10.6004/jnccn.2017.0150]

### Parker 2017b {published data only}

- Parker RA, Weir CJ, Maxwell AE, Al-Shahi Salman R. Promoting Recruitment using Information Management Efficiently (PRIME): statistical analysis plan for a stepped wedge cluster randomised trial within the REstart or STop Antithrombotics Randomised Trial (RESTART). Trials 2017;18(1):94. [DOI: 10.1186/s13063-017-1840-8]

### Pawlak 2015 {published data only}

- Pawlak CE, Fields Jr HW, Beck FM, Firestone AR. Orthodontic informed consent considering information load and serial position effect. American Journal of Orthodontics and Dentofacial Orthopedics 2015;147(3):363-72. [DOI: 10.1016/j.ajodo.2014.11.021]

### Peel 2019 {published data only}

- Peel R, Ren S, Hure A, Evans TJ, D'Este CA, Abhayaratna WP, et al. Evaluating recruitment strategies for AUSPICE, a large Australian community‐based randomised controlled trial. Medical Journal of Australia 2019;210(9):409-15. [DOI: 10.5694/mja2.50117]

### Peng 2019 {published data only}

- Peng W, Morgan SE, Mao B, McFarlane SJ, Occa A, Grinfeder G, et al. Ready to make a decision: a model of informational aids to improve informed participation in clinical trial research. Journal of Health Communication 2019;24(12):865-77. [DOI: 10.1080/10810730.2019.1680773]

### Perrone 1995 {published data only}

- \*Perrone F, De Placido S, Giusti C, Gallo C. Looking for consent in RCTs: a randomised trial with surrogate patients. Epidemiologia e Prevenzione 1995;19:282‐90.

### Peters 2022 {published data only}

- Peters AE, Ogunniyi MO, Hegde SM, Bianco C, Ghafghazi S, Hernandez AF, et al. A multicenter program for electronic health record screening for patients with heart failure with preserved ejection fraction: lessons from the DELIVER-EHR initiative. Contemporary Clinical Trials 2022;121:106924. [DOI: 10.1016/j.cct.2022.106924]

### Pighills 2009 {published data only}

- Pighills A, Torgerson DJ, Sheldon T. Publicity does not increase recruitment to falls prevention trials: the results of two quasi‐randomized trials. Journal of Clinical Epidemiology 2009;62:1332‐5. [DOI: 10.1016/j.jclinepi.2009.01.017]

### Plante 2020 {published data only}

- Plante TB, Gleason KT, Miller HN, Charleston J, McArthur K, Himmelfarb CD, et al. Recruitment of trial participants through electronic medical record patient portal messaging: a pilot study. Clinical Trials (London, England) 2020;17(1):30-8. [DOI: 10.1177/174077451987365]

### Realpe 2016 {published data only}

- Realpe A, Adams A, Wall P, Griffin D, Donovan JL. A new simple six-step model to promote recruitment to RCTs was developed and successfully implemented. Journal of Clinical Epidemiology 2016;76:166-74. [DOI: 10.1016/j.jclinepi.2016.02.002]

### Rimel 2015 {published data only}

- Rimel BJ, Lester J, Sabacan L, Park D, Bresee C, Dang C, et al. A novel clinical trial recruitment strategy for women's cancer. Gynecologic Oncology 2015;138(2):445-8. [DOI: 10.1016/j.ygyno.2015.05.008]

### Robinson 2017 {published data only}

- Robinson BN, Newman AF, Tefera E, Herbolsheimer P, Nunes R, Gallagher C, et al. Video intervention increases participation of black breast cancer patients in therapeutic trials. NPJ Breast Cancer 2017;3(1):36. [DOI: 10.1038/s41523-017-0039-1]

### Shepherd 2022 {published data only}

- Shepherd V, Wood F, Gillies K, Martin A, O’Connell A, Hood K. Feasibility, effectiveness and costs of a decision support intervention for consultees and legal representatives of adults lacking capacity to consent (CONSULT): protocol for a randomised Study Within a Trial. Trials 2022;23(1):957. [DOI: 10.1186/s13063-022-06887-5]

### Simel 1991 {published data only}

- \*Simel DL, Feussner JR. A randomized controlled trial comparing quantitative informed consent formats. Journal of Clinical Epidemiology 1991;44(8):771‐7. [DOI: 10.1016/0895-4356(91)90129-W]

### Simmons 2022 {published data only}

- Simmons LA, Phipps JE, Whipps M, Smith P, Carbajal KA, Overstreet C, et al. From hybrid to fully remote clinical trial amidst the COVID-19 pandemic: strategies to promote recruitment, retention, and engagement in a randomized mHealth trial. Digital Health 2022;8:1-8. [DOI: 10.1177/20552076221129065]

### So 2018 {published data only}

- So R, Shinohara K, Aoki T, Tsujimoto Y, Suganuma AM, Furukawa TA. Effect of recruitment methods on response rate in a web-based study for primary care physicians: factorial randomized controlled trial. Journal of Medical Internet Research 2018;20(2):28. [DOI: 10.2196/jmir.8561]

### Stephens 2022 {published data only}

- Stephens SW, Farley P, Collins SP, Wong MD, Panas AB, Dennis BM, et al. Multicenter social media community consultation for an exception from informed consent trial of the XStat device (PhoXStat trial). Journal of Trauma and Acute Care Surgery 2022;92(2):442-6. [DOI: 10.1097/TA.0000000000003425]

### Sygna 2015 {published data only}

- Sygna K, Johansen S, Ruland CM. Recruitment challenges in clinical research including cancer patients and caregivers. Trials 2015;16(1):428. [DOI: 10.1186/s13063-015-0948-y]

### Tattersall 2017 {published data only}

- Tattersall MH, Jefford M, Martin A, Olver I, Thompson JF, Brown RF, et al. Parallel multicentre randomised trial of a clinical trial question prompt list in patients considering participation in phase 3 cancer treatment trials. BMJ Open 2017;7(3):e012666. [DOI: 10.1136/bmjopen-2016-012666]

### Tehranisa 2014 {published data only}

- Tehranisa JS, Meurer WJ. Can response-adaptive randomization increase participation in acute stroke trials? Stroke 2014;45:2131-3. [DOI: 10.1161/STROKEAHA.114.005418]

### Thilsing 2021 {published data only}

- Thilsing T, Larsen LB, Sonderlund AL, Andreassen SS, Christensen JR, Svensson NH, et al; Sondergaard. Effects of a co-design–based invitation strategy on participation in a preventive health check program: randomized controlled trial. JMIR Public Health and Surveillance 2021;7(3):e25617. [DOI: 10.2196/25617]

### Tilley 2017 {published data only}

- Tilley BC, Mainous III AG, Smith DW, McKee MD, Amorrortu RP, Alvidrez J, et al. Design of a cluster-randomized minority recruitment trial: RECRUIT. Clinical Trials (London, England) 2017;14(3):286-98. [DOI: 10.1177/1740774517690146]

### Treschan 2003 {published data only}

- Treschan TA, Scheck T, Kober A, Fleischmann E, Birkenberg B, Petschnigg B, et al. The influence of protocol pain and risk on patients' willingness to consent for clinical studies: a randomized trial. Economics, Education, and Health Systems Research 2003;96:498‐506. [DOI: 10.1213/00000539-200302000-00037]

### Uveges 2018 {published data only}

- Uveges MK, Lansey DG, Mbah O, Gray T, Sherden L, Wenzel J. Patient navigation and clinical trial participation: a randomized controlled trial design. Contemporary Clinical Trials Communications 2018;12:98-102. [DOI: 10.1016/j.conctc.2018.09.003]

### Valentine 2020 {published data only}

- Valentine S, Majer J, Grant N, Ugoni A, Taylor DM. The effect of the consent process on patient satisfaction with pain management: a randomized controlled trial. Annals of Emergency Medicine 2021;77(1):82-90. [DOI: 10.1016/j.annemergmed.2020.03.029]

### Villanti 2015 {published data only}

- Villanti AC, Jacobs MA, Zawistowski G, Brookover J, Stanton CA, Graham AL. Impact of baseline assessment modality on enrollment and retention in a Facebook smoking cessation study. Journal of Medical Internet Research 2015;17(7):179. [DOI: 10.2196/jmir.4341]

### Wallace 2021 {published data only}

- Wallace MK, Still CH, Jeanblanc AB, Musil CM. Successful and cost-effective Facebook recruitment: is it possible? Results from a longitudinal randomized clinical trial in grandmother caregivers. International Journal of Aging and Human Development 2021;93(4):1031-50. [DOI: 10.1177/0091415020987667]

### Warner 2021 {published data only}

- Warner G, Osman F, McDiarmid S, Sarkadi A. Giving schools a nudge: can behavioural insights improve recruitment of schools to randomised controlled trials? BMC Research Notes 2021;14(1):99. [DOI: 10.1186/s13104-021-05509-8]

### Warnock 2022 {published data only}

- Warnock AC, Kaur H, Buckman JR, Hoenemeyer T, Demark-Wahnefried W. A comparison of two mail-based strategies to recruit older cancer survivors into a randomized controlled trial of a lifestyle intervention. Journal of Cancer Survivorship 2022;16(5):998-1003. [PMID: 10.1007/s11764-021-01091-x]

### Weinfurt 2008a {published data only}

- Weinfurt KP, Hall MA, Friedman JY, Hardy NC, Fortune-Greeley AK, Lawlor JS, et al. Effects of disclosing financial interests on participation in medical research: a randomized vignette trial. Journal of General Internal Medicine 2008;23:860-6.

### Weinfurt 2008b {published data only}

- Weinfurt KP, Hall MA, Friedman JY, Hardy NC, Fortune-Greeley AK, Lawlor JS, et al. Effects of disclosing financial interests on participation in medical research: a randomized vignette trial. American Heart Journal 2008;156:689-97.

### Wells 2013 {published data only}

- Wells KJ, McIntyre J, Gonzalez LE, Lee JH, Fisher KJ, Jacobsen PB. Feasibility trial of a Spanish-language multimedia educational intervention. Clinical Trials (London, England) 2013;10:767-74.

### Welton 1999 {published data only}

- Welton AJ, Vickers MR, Cooper JA, Meade TW, Marteau TM. Is recruitment more difficult with a placebo arm in randomised controlled trials? A quasi randomised, interview based study. BMJ 1991;318:1114-7.

### Weston 1997 {published data only}

- Weston J, Hannah M, Downes J. Evaluating the benefits of a patient information video during the informed consent process. Patient Education and Counseling 1997;30:239-45.

### Winhusen 2020 {published data only}

- Winhusen T, Wilder C, Kropp F, Theobald J, Lyons MS, Lewis D. A brief telephone-delivered peer intervention to encourage enrollment in medication for opioid use disorder in individuals surviving an opioid overdose: results from a randomized pilot trial. Drug and Alcohol Dependence 2020;216:108270. [DOI: 10.1016/j.drugalcdep.2020.108270]

### Won 2022 {published data only}

- Won J, Bang H, Lee H. Effect of partial vs full disclosure of potential assignment to placebo on participant blinding, perceptions of group assignment, and trial outcomes: a randomized clinical trial. JAMA Network Open 2022;5(3):e224050. [DOI: 10.1001/jamanetworkopen.2022.4050]

### Woodford 2020 {published data only}

- Woodford J, Norbäck K, Hagström J, Grönqvist H, Parker A, Arundel C, et al. Study within a trial (SWAT) protocol. Investigating the effect of personalised versus non-personalised study invitations on recruitment: an embedded randomised controlled recruitment trial. Contemporary Clinical Trials Communications 2020;18:100572. [DOI: 10.1016/j.conctc.2020.100572]

### Yeary 2018 {published data only}

- Yeary KH, Moore P, Turner J, Dawson L, Heo S, Greene P. Feasibility test of a community-relevant intervention designed to promote African American participation in translational, breast cancer disparities research: know about Health Options for Women (Know HOW). Journal of Cancer Education 2018;33(1):29-36. [DOI: 10.1007/s13187-016-1032-3]
