# Supplementary material for: Strategies to improve recruitment to randomised trials
Source: Cochrane Database Syst Rev. 2026 Sep 15;2026(9):MR000013. doi: 10.1002/14651858.MR000013.pub7 (PMC13576036; doi:10.1002/14651858.MR000013.pub7)
Supplement: Supplementary file 4 [file MR000013-SUP-04-analyses.html]

Analyses


# Supplementary material 4 to: Strategies to improve recruitment to randomised trials

Parker A, Mongelli G, Piccolo-Lawrance C, Coleman E, Gkekas A, Wang HI, Dawson S, Green H, Way R, Rai A, Bruhn H, Sullivan F, Boxall C, Chan M, Manson P, Hewitt C, Fairhurst C, Clarke M, Treweek S
  
https://doi.org/10.1002/14651858.MR000013.pub7

The material in this section has been supplied by the author(s) for publication under a Licence for Publication and the author(s) are solely responsible for the material. Cochrane has reviewed this material, but Cochrane has not copyedited, formatted or proofread. Cochrane accordingly gives no representations or warranties of any kind in relation to, and accepts no liability for any reliance on or use of, such material.

Back to top

# Analyses

## Analysis group 1: A–Open-label trial vs blinded trial (GRADE: high)

| Analysis or subgroup title | No. of studies | No. of participants | Statistical method | Effect size |
| --- | --- | --- | --- | --- |
| 1.1 Participants recruited | 3 | 9004 | Risk Difference (IV, Random, 95% CI) | 0.10 [0.08, 0.12] |

## Analysis group 2: A–Patient preference design vs conventional RCT (GRADE: low)

| Analysis or subgroup title | No. of studies | No. of participants | Statistical method | Effect size |
| --- | --- | --- | --- | --- |
| 2.1 Participants recruited | 1 | 273 | Risk Difference (M-H, Fixed, 95% CI) | −0.04 [−0.15, 0.07] |

## Analysis group 3: A–Electronic data capture vs paper-based data capture (GRADE: low)

| Analysis or subgroup title | No. of studies | No. of participants | Statistical method | Effect size |
| --- | --- | --- | --- | --- |
| 3.1 Participants recruited | 1 | 80 | Risk Difference (M-H, Fixed, 95% CI) | −0.13 [−0.24, −0.03] |

## Analysis group 6: B–Pre-engagement with potential trial sites during proposal development vs no pre-engagement (GRADE: low)

| Analysis or subgroup title | No. of studies | No. of participants | Statistical method | Effect size |
| --- | --- | --- | --- | --- |
| 6.1 Participants Recruited | 1 | 33 | Risk Difference (M-H, Fixed, 95% CI) | 0.50 [0.18, 0.81] |

## Analysis group 7: C–Telephone reminder vs no telephone reminder (GRADE: high)

| Analysis or subgroup title | No. of studies | No. of participants | Statistical method | Effect size |
| --- | --- | --- | --- | --- |
| 7.1 Participants recruited | 2 | 1450 | Risk Difference (IV, Random, 95% CI) | 0.06 [0.03, 0.09] |

## Analysis group 8: C–SMS reminder mentioning scarcity vs SMS reminder with no mention (GRADE: moderate)

| Analysis or subgroup title | No. of studies | No. of participants | Statistical method | Effect size |
| --- | --- | --- | --- | --- |
| 8.1 Participants recruited | 1 | 1862 | Risk Difference (M-H, Fixed, 95% CI) | 0.03 [0.01, 0.06] |

## Analysis group 9: C–SMS messages containing quotes from existing participants vs no messages (GRADE: moderate)

| Analysis or subgroup title | No. of studies | No. of participants | Statistical method | Effect size |
| --- | --- | --- | --- | --- |
| 9.1 Participants recruited | 1 | 811 | Risk Difference (M-H, Fixed, 95% CI) | 0.04 [0.02, 0.06] |

## Analysis group 10: C–Email invitation vs postal invitation (GRADE: moderate)

| Analysis or subgroup title | No. of studies | No. of participants | Statistical method | Effect size |
| --- | --- | --- | --- | --- |
| 10.1 Participants recruited | 1 | 1760 | Risk Difference (M-H, Fixed, 95% CI) | 0.01 [−0.03, 0.04] |

## Analysis group 11: C–Telephone reminders vs SMS reminders (GRADE: moderate)

| Analysis or subgroup title | No. of studies | No. of participants | Statistical method | Effect size |
| --- | --- | --- | --- | --- |
| 11.1 Participants recruited | 1 | 709 | Risk Difference (M-H, Fixed, 95% CI) | 0.05 [−0.01, 0.11] |

## Analysis group 12: D–Opt-out consent vs opt-in consent (GRADE: low)

| Analysis or subgroup title | No. of studies | No. of participants | Statistical method | Effect size |
| --- | --- | --- | --- | --- |
| 12.1 Participants recruited | 1 | 152 | Risk Difference (M-H, Fixed, 95% CI) | 0.19 [0.03, 0.35] |

## Analysis group 13: D–Researcher reading out consent vs participant reading consent (GRADE: very low)

| Analysis or subgroup title | No. of studies | No. of participants | Statistical method | Effect size |
| --- | --- | --- | --- | --- |
| 13.1 Participants recruited | 1 | 104 | Risk Difference (M-H, Fixed, 95% CI) | 0.06 [−0.13, 0.25] |

## Analysis group 15: D–Information printed on heavyweight paper and blue folio vs standard (high risk of bias)

| Analysis or subgroup title | No. of studies | No. of participants | Statistical method | Effect size |
| --- | --- | --- | --- | --- |
| 15.1 Participants recruited | 1 | 499 | Risk Difference (M-H, Fixed, 95% CI) | −0.09 [−0.17, −0.01] |

## Analysis group 16: D–Enhanced pre-consent discussion with aid of poster vs. standard consent (high risk of bias)

| Analysis or subgroup title | No. of studies | No. of participants | Statistical method | Effect size |
| --- | --- | --- | --- | --- |
| 16.1 Participants recruited | 1 | 769 | Risk Difference (M-H, Fixed, 95% CI) | −0.01 [−0.08, 0.06] |

## Analysis group 17: E–Brief participant information leaflet (PIL) vs full PIL (GRADE: moderate)

| Analysis or subgroup title | No. of studies | No. of participants | Statistical method | Effect size |
| --- | --- | --- | --- | --- |
| 17.1 Participants recruited | 2 | 4633 | Risk Difference (IV, Random, 95% CI) | 0.00 [−0.01, 0.02] |

## Analysis group 18: E–Study-related questionnaire + trial invitation vs trial invitation (GRADE: moderate)

| Analysis or subgroup title | No. of studies | No. of participants | Statistical method | Effect size |
| --- | --- | --- | --- | --- |
| 18.1 Participants recruited | 1 | 2393 | Risk Difference (M-H, Fixed, 95% CI) | 0.05 [0.02, 0.08] |

## Analysis group 19: E–Optimised PIL (developed with feedback from users) vs usual PIL (GRADE: moderate)

| Analysis or subgroup title | No. of studies | No. of participants | Statistical method | Effect size |
| --- | --- | --- | --- | --- |
| 19.1 Participants recruited | 2 | 16,763 | Risk Difference (IV, Random, 95% CI) | 0.00 [−0.00, 0.01] |

## Analysis group 20: E–Enhanced recruitment package + recruitment at churches vs standard recruitment package (GRADE: low)

| Analysis or subgroup title | No. of studies | No. of participants | Statistical method | Effect size |
| --- | --- | --- | --- | --- |
| 20.1 Participants recruited | 1 | 6246 | Risk Difference (M-H, Fixed, 95% CI) | 0.01 [0.00, 0.02] |

## Analysis group 21: E–Enhanced recruitment package vs standard recruitment package (GRADE: low)

| Analysis or subgroup title | No. of studies | No. of participants | Statistical method | Effect size |
| --- | --- | --- | --- | --- |
| 21.1 Participants recruited | 1 | 6376 | Risk Difference (M-H, Fixed, 95% CI) | −0.00 [−0.01, 0.00] |

## Analysis group 22: E–Enhanced recruitment package + baseline data over telephone vs standard recruitment package (GRADE: low)

| Analysis or subgroup title | No. of studies | No. of participants | Statistical method | Effect size |
| --- | --- | --- | --- | --- |
| 22.1 Participants recruited | 1 | 6372 | Risk Difference (M-H, Fixed, 95% CI) | −0.00 [−0.01, 0.01] |

## Analysis group 23: E–Audio record of information given about trial vs no audio record (GRADE: very low)

| Analysis or subgroup title | No. of studies | No. of participants | Statistical method | Effect size |
| --- | --- | --- | --- | --- |
| 23.1 Participants recruited | 1 | 130 | Risk Difference (M-H, Fixed, 95% CI) | −0.03 [−0.19, 0.13] |

## Analysis group 24: E–Clinical trial booklet + standard information vs standard information (GRADE: very low)

| Analysis or subgroup title | No. of studies | No. of participants | Statistical method | Effect size |
| --- | --- | --- | --- | --- |
| 24.1 Participants recruited | 1 | 31 | Risk Difference (M-H, Fixed, 95% CI) | 0.20 [−0.05, 0.46] |

## Analysis group 25: E–Total information disclosure vs standard disclosure (GRADE: very low)

| Analysis or subgroup title | No. of studies | No. of participants | Statistical method | Effect size |
| --- | --- | --- | --- | --- |
| 25.1 Participants recruited | 1 | 57 | Risk Difference (M-H, Fixed, 95% CI) | 0.11 [−0.06, 0.28] |

## Analysis group 26: E–Access to cancer trials website vs no access (high risk of bias)

| Analysis or subgroup title | No. of studies | No. of participants | Statistical method | Effect size |
| --- | --- | --- | --- | --- |
| 26.1 Participants recruited | 1 | 340 | Risk Difference (M-H, Fixed, 95% CI) | −0.01 [−0.07, 0.06] |

## Analysis group 27: E–Optimised PIL (bespoke & user-tested) vs usual PIL (GRADE: high)

| Analysis or subgroup title | No. of studies | No. of participants | Statistical method | Effect size |
| --- | --- | --- | --- | --- |
| 27.1 Participants recruited | 6 | 27,805 | Risk Difference (IV, Random, 95% CI) | 0.00 [−0.00, 0.01] |

## Analysis group 28: E–Recruitment primer letter/leaflet vs no letter/leaflet (GRADE: high)

| Analysis or subgroup title | No. of studies | No. of participants | Statistical method | Effect size |
| --- | --- | --- | --- | --- |
| 28.1 Participants recruited | 2 | 5376 | Risk Difference (IV, Random, 95% CI) | 0.01 [−0.01, 0.02] |

## Analysis group 29: E–Information provided remotely vs information provided face-to-face (GRADE: moderate)

| Analysis or subgroup title | No. of studies | No. of participants | Statistical method | Effect size |
| --- | --- | --- | --- | --- |
| 29.1 Participants recruited | 2 | 249 | Risk Difference (IV, Random, 95% CI) | −0.09 [−0.18, 0.01] |

## Analysis group 30: E–Providing information by video vs standard information (GRADE: very low)

| Analysis or subgroup title | No. of studies | No. of participants | Statistical method | Effect size |
| --- | --- | --- | --- | --- |
| 30.1 Participants recruited | 5 | 1634 | Risk Difference (IV, Random, 95% CI) | −0.04 [−0.23, 0.15] |

## Analysis group 31: E–Invitation letter emphasising ethnicity-specific disease risk vs standard letter (GRADE: low)

| Analysis or subgroup title | No. of studies | No. of participants | Statistical method | Effect size |
| --- | --- | --- | --- | --- |
| 31.1 Participants recruited | 1 | 445 | Risk Difference (M-H, Fixed, 95% CI) | −0.03 [−0.12, 0.06] |

## Analysis group 32: E–Handwritten personalised invitation letter vs standard letter (GRADE: low)

| Analysis or subgroup title | No. of studies | No. of participants | Statistical method | Effect size |
| --- | --- | --- | --- | --- |
| 32.1 Participants recruited | 1 | 317 | Risk Difference (M-H, Fixed, 95% CI) | −0.04 [−0.08, 0.00] |
| 32.2 Participants retained | 1 | 317 | Risk Difference (M-H, Fixed, 95% CI) | −0.04 [−0.08, 0.00] |

## Analysis group 33: E–Different website wording, including infographic, about the risk of underlying condition (GRADE: Very low)

| Analysis or subgroup title | No. of studies | No. of participants | Statistical method | Effect size |
| --- | --- | --- | --- | --- |
| 33.1 Participants recruited | 2 | 2605 | Risk Difference (IV, Random, 95% CI) | −0.00 [−0.01, 0.01] |

## Analysis group 34: E– Shorter recruitment package vs. Standard recruitment package (GRADE: very low)

| Analysis or subgroup title | No. of studies | No. of participants | Statistical method | Effect size |
| --- | --- | --- | --- | --- |
| 34.1 Participants recruited | 1 | 170 | Risk Difference (M-H, Fixed, 95% CI) | 0.02 [−0.13, 0.17] |

## Analysis group 35: E– Shorter recruitment pack with simplified consent vs. Standard recruitment package (GRADE: very low)

| Analysis or subgroup title | No. of studies | No. of participants | Statistical method | Effect size |
| --- | --- | --- | --- | --- |
| 35.1 Participants recruited | 1 | 175 | Risk Difference (M-H, Fixed, 95% CI) | 0.02 [−0.13, 0.17] |

## Analysis group 36: E–Leaflet advertising PPI involvement in trial + standard trial invitation vs standard trial invitation only (GRADE: moderate)

| Analysis or subgroup title | No. of studies | No. of participants | Statistical method | Effect size |
| --- | --- | --- | --- | --- |
| 36.1 Participants recruited | 1 | 8182 | Risk Difference (M-H, Fixed, 95% CI) | −0.01 [−0.02, −0.00] |

## Analysis group 37: E–Gender-targeted recruitment postcard vs generic recruitment postcard (GRADE: low)

| Analysis or subgroup title | No. of studies | No. of participants | Statistical method | Effect size |
| --- | --- | --- | --- | --- |
| 37.1 Participants recruited | 1 | 30,000 | Risk Difference (M-H, Fixed, 95% CI) | 0.00 [−0.00, 0.00] |

## Analysis group 38: E–Researcher optimised PIL vs standard PIL (GRADE: low)

| Analysis or subgroup title | No. of studies | No. of participants | Statistical method | Effect size |
| --- | --- | --- | --- | --- |
| 38.1 Participants recruited | 1 | 480 | Risk Difference (M-H, Fixed, 95% CI) | −0.09 [−0.17, −0.00] |

## Analysis group 39: E–In-clinic information video viewing vs information DVD to take home (high risk of bias)

| Analysis or subgroup title | No. of studies | No. of participants | Statistical method | Effect size |
| --- | --- | --- | --- | --- |
| 39.1 Participants recruited | 1 | 79 | Risk Difference (M-H, Fixed, 95% CI) | 0.03 [−0.08, 0.14] |

## Analysis group 40: E–Brief PIL + standard PIL vs standard PIL (GRADE: low)

| Analysis or subgroup title | No. of studies | No. of participants | Statistical method | Effect size |
| --- | --- | --- | --- | --- |
| 40.1 Participants recruited | 1 | 6946 | Risk Difference (M-H, Fixed, 95% CI) | 0.00 [−0.01, 0.01] |

## Analysis group 41: E–Decision aid about RCTs vs blank notebook (GRADE: low)

| Analysis or subgroup title | No. of studies | No. of participants | Statistical method | Effect size |
| --- | --- | --- | --- | --- |
| 41.1 Participant recruited | 1 | 129 | Risk Difference (M-H, Fixed, 95% CI) | 0.12 [−0.01, 0.24] |

## Analysis group 42: E–Interactive online digital eductional tool vs time-matched webiner (GRADE: low)

| Analysis or subgroup title | No. of studies | No. of participants | Statistical method | Effect size |
| --- | --- | --- | --- | --- |
| 42.1 Participants recruited | 1 | 351 | Risk Difference (M-H, Fixed, 95% CI) | 0.01 [−0.01, 0.03] |

## Analysis group 43: E–PPI designed and informed PIL vs standard researcher designed PIL (GRADE: very low)

| Analysis or subgroup title | No. of studies | No. of participants | Statistical method | Effect size |
| --- | --- | --- | --- | --- |
| 43.1 Participants recruited | 1 | 207 | Risk Difference (M-H, Fixed, 95% CI) | −0.02 [−0.15, 0.12] |
| 43.2 Participants retained | 1 | 207 | Risk Difference (M-H, Fixed, 95% CI) | 0.04 [−0.09, 0.18] |

## Analysis group 44: E–Culturally-tailored educational DVD about clinical trials + usual education vs usual education by healthcare staff only (high risk of bias)

| Analysis or subgroup title | No. of studies | No. of participants | Statistical method | Effect size |
| --- | --- | --- | --- | --- |
| 44.1 Participants recruited | 1 | 37 | Risk Difference (M-H, Fixed, 95% CI) | 0.03 [−0.19, 0.25] |

## Analysis group 45: E–Multimedia education about clinical trials (DVD + booklet) vs standard educational booklet only (GRADE: low)

| Analysis or subgroup title | No. of studies | No. of participants | Statistical method | Effect size |
| --- | --- | --- | --- | --- |
| 45.1 Participants recruited | 1 | 418 | Risk Difference (M-H, Fixed, 95% CI) | 0.04 [−0.05, 0.14] |

## Analysis group 46: E–PIL minimising the use of phrases with negative connotations vs standard PIL (high risk of bias)

| Analysis or subgroup title | No. of studies | No. of participants | Statistical method | Effect size |
| --- | --- | --- | --- | --- |
| 46.1 Participants recruited | 1 | 4145 | Risk Difference (M-H, Fixed, 95% CI) | −0.01 [−0.02, 0.00] |

## Analysis group 47: E–Multimedia information (viewed in person) + paper PIL vs paper PIL only (GRADE: very low)

| Analysis or subgroup title | No. of studies | No. of participants | Statistical method | Effect size |
| --- | --- | --- | --- | --- |
| 47.1 Participant recruitment | 3 | 23 | Risk Difference (IV, Random, 95% CI) | 0.17 [−0.89, 1.23] |
| 47.2 Participants retained | 3 | 23 | Risk Difference (IV, Random, 95% CI) | 0.28 [−0.79, 1.36] |

## Analysis group 48: E–Multimedia information (viewed in person) PIL vs paper PIL (GRADE: moderate)

| Analysis or subgroup title | No. of studies | No. of participants | Statistical method | Effect size |
| --- | --- | --- | --- | --- |
| 48.1 Participants recruited | 3 | 1428 | Risk Difference (IV, Random, 95% CI) | 0.04 [−0.01, 0.08] |
| 48.2 Participants retained | 3 | 1428 | Risk Difference (IV, Random, 95% CI) | 0.22 [−0.51, 0.96] |

## Analysis group 49: E–Multimedia information (provided as link/QR code) + paper PIL vs paper PIL (GRADE: high)

| Analysis or subgroup title | No. of studies | No. of participants | Statistical method | Effect size |
| --- | --- | --- | --- | --- |
| 49.1 Participants recruited | 7 | 11,612 | Risk Difference (IV, Random, 95% CI) | −0.00 [−0.01, 0.01] |
| 49.2 Participants retained | 5 | 7403 | Risk Difference (IV, Random, 95% CI) | 0.00 [−0.02, 0.03] |

## Analysis group 50: E–Multimedia information (provided as link/QR code) vs paper PIL (GRADE: moderate)

| Analysis or subgroup title | No. of studies | No. of participants | Statistical method | Effect size |
| --- | --- | --- | --- | --- |
| 50.1 Participants recruited | 2 | 229 | Risk Difference (IV, Random, 95% CI) | 0.13 [0.01, 0.26] |
| 50.2 Participants retained | 2 | 229 | Risk Difference (IV, Random, 95% CI) | 0.13 [−0.00, 0.26] |

## Analysis group 54: F–Teaser campaign using postcards vs no teaser (GRADE: moderate)

| Analysis or subgroup title | No. of studies | No. of participants | Statistical method | Effect size |
| --- | --- | --- | --- | --- |
| 54.1 Primary care centre recruited | 1 | 670 | Risk Difference (M-H, Fixed, 95% CI) | 0.00 [−0.04, 0.05] |

## Analysis group 55: F–Educational program to tailor recruitment strategies for minority recruitment vs usual recruitment (GRADE: low)

| Analysis or subgroup title | No. of studies | No. of participants | Statistical method | Effect size |
| --- | --- | --- | --- | --- |
| 55.1 Participants recruited | 1 | 2107 | Risk Difference (M-H, Fixed, 95% CI) | −0.00 [−0.04, 0.03] |

## Analysis group 56: F–Remote vs face-to-face site initiation visits (GRADE: moderate)

| Analysis or subgroup title | No. of studies | No. of participants | Statistical method | Effect size |
| --- | --- | --- | --- | --- |
| 56.1 Participants recruited | 1 | 688 | Risk Difference (M-H, Fixed, 95% CI) | 0.02 [−0.05, 0.10] |
| 56.2 Participants retained | 1 | 688 | Risk Difference (M-H, Fixed, 95% CI) | 0.01 [−0.06, 0.09] |

## Analysis group 63: G–Monetary incentive vs no incentive (GRADE: low)

| Analysis or subgroup title | No. of studies | No. of participants | Statistical method | Effect size |
| --- | --- | --- | --- | --- |
| 63.1 Participants recruited | 12 | 5082 | Risk Difference (IV, Random, 95% CI) | 0.05 [0.01, 0.09] |
| 63.1.1 Up to £5 | 2 | 1323 | Risk Difference (IV, Random, 95% CI) | 0.03 [−0.02, 0.07] |
| 63.1.2 Around £100 | 6 | 1335 | Risk Difference (IV, Random, 95% CI) | 0.03 [−0.02, 0.08] |
| 63.1.3 Over £100 | 4 | 2424 | Risk Difference (IV, Random, 95% CI) | 0.10 [−0.02, 0.21] |
| 63.2 Participants recruited (by allocation concealment) | 12 | 5082 | Risk Difference (IV, Random, 95% CI) | 0.05 [0.01, 0.09] |
| 63.2.1 Adequate allocation concealment | 8 | 3786 | Risk Difference (IV, Random, 95% CI) | 0.03 [0.00, 0.05] |
| 63.2.2 Inadequate or unclear allocation concealment | 4 | 1296 | Risk Difference (IV, Random, 95% CI) | 0.10 [−0.02, 0.22] |

## Analysis group 64: G–Monetary incentive: higher value vs lower value (GRADE: very low)

| Analysis or subgroup title | No. of studies | No. of participants | Statistical method | Effect size |
| --- | --- | --- | --- | --- |
| 64.1 Participants recruited | 2 | 864 | Risk Difference (IV, Random, 95% CI) | 0.03 [−0.13, 0.19] |

## Analysis group 65: G–Non-monetary incentive (branded pen vs no pen) (GRADE: moderate)

| Analysis or subgroup title | No. of studies | No. of participants | Statistical method | Effect size |
| --- | --- | --- | --- | --- |
| 65.1 Participants recruited | 3 | 9640 | Risk Difference (IV, Random, 95% CI) | −0.00 [−0.01, 0.01] |
| 65.2 Participants retained | 1 | 1862 | Risk Difference (M-H, Fixed, 95% CI) | 0.00 [−0.02, 0.02] |

# Figures and tables

Analysis 1.1: Participants recruited


Study or Subgroup
Avenell 2004
Hemminki 2004
Veerus 2016

Total (Wald
a
)
Total events:
Test for overall effect: Z = 10.07 (P < 0.00001)

Heterogeneity: Tau² (REML
b
) = 0.00; Chi² = 0.10, df = 2 (P = 0.95); I² = 0%

Open
Events
134
1027
1001
2162
Total
180
2159
2087

4426

Blinded
Events
233
796
777
1806
Total
358
2136
2084

4578
Weight
6.3%
47.5%
46.1%

100.0%

Risk Difference
IV, Random, 95% CI
0.09 [0.01 , 0.17]
0.10 [0.07 , 0.13]
0.11 [0.08 , 0.14]

0.10 [0.08 , 0.12]

Risk Difference
IV, Random, 95% CI


-1

-0.5

0

0.5

1


Favours blinded

Favours open-label


Risk of Bias
A

+

+

+
B

+

+

+
C

+

+

+
D

+

+

+
E

+

+

?
F

+

+

+
G

+

+

+
H

+

+

?

Footnotes

a
CI calculated by Wald-type method.

b
Tau² calculated by Restricted Maximum-Likelihood method.
Risk of bias legend

(A) Random sequence generation ok?

(B) Allocation concealment?

(C) Blinding of participants and personnel ok?

(D) Blinding of outcome assessment ok?

(E) Incomplete outcome data handled ok?

(F) Free of selective reporting?

(G) Was the study free of other bias?

(H) Overall bias?


Analysis 2.1: Participants recruited


Study or Subgroup
Cooper 1997

Total
Total events:
Test for overall effect: Z = 0.64 (P = 0.52)

Heterogeneity: Not applicable

Patient preference design
Events
90
90
Total
135

135

Conventional design
Events
97
97
Total
138

138
Weight
100.0%

100.0%

Risk Difference
M-H, Fixed, 95% CI
-0.04 [-0.15 , 0.07]

-0.04 [-0.15 , 0.07]

Risk Difference
M-H, Fixed, 95% CI


-2

-1

0

1

2


Favours conventional

Favours preference


Risk of Bias
A

+
B

+
C

+
D

+
E

+
F

+
G

+
H

+

Risk of bias legend

(A) Random sequence generation ok?

(B) Allocation concealment?

(C) Blinding of participants and personnel ok?

(D) Blinding of outcome assessment ok?

(E) Incomplete outcome data handled ok?

(F) Free of selective reporting?

(G) Was the study free of other bias?

(H) Overall bias?


Analysis 3.1: Participants recruited


Study or Subgroup
Litchfield 2005

Total
Total events:
Test for overall effect: Z = 2.51 (P = 0.01)

Heterogeneity: Not applicable

Electronic data capture
Events
45
45
Total
52

52

Paper data capture
Events
28
28
Total
28

28
Weight
100.0%

100.0%

Risk Difference
M-H, Fixed, 95% CI
-0.13 [-0.24 , -0.03]

-0.13 [-0.24 , -0.03]

Risk Difference
M-H, Fixed, 95% CI


-4

-2

0

2

4


Favours paper

Favours electronic


Risk of Bias
A

+
B

+
C

?
D

+
E

+
F

+
G

+
H

?

Risk of bias legend

(A) Random sequence generation ok?

(B) Allocation concealment?

(C) Blinding of participants and personnel ok?

(D) Blinding of outcome assessment ok?

(E) Incomplete outcome data handled ok?

(F) Free of selective reporting?

(G) Was the study free of other bias?

(H) Overall bias?


Analysis 6.1: Participants Recruited


Study or Subgroup
Garvelink 2015

Total
Total events:
Test for overall effect: Z = 3.12 (P = 0.002)

Heterogeneity: Not applicable

Pre-engagment
Events
16
16
Total
23

23

No pre-engagement
Events
2
2
Total
10

10
Weight
100.0%

100.0%

Risk Difference
M-H, Fixed, 95% CI
0.50 [0.18 , 0.81]

0.50 [0.18 , 0.81]

Risk Difference
M-H, Fixed, 95% CI


-10

-5

0

5

10


Favours no pre-engagment

Favours pre-engagment


Risk of Bias
A

+
B

+
C

?
D

+
E

+
F

+
G

+
H

?

Risk of bias legend

(A) Random sequence generation ok?

(B) Allocation concealment?

(C) Blinding of participants and personnel ok?

(D) Blinding of outcome assessment ok?

(E) Incomplete outcome data handled ok?

(F) Free of selective reporting?

(G) Was the study free of other bias?

(H) Overall bias?


Analysis 7.1: Participants recruited


Study or Subgroup
Nystuen 2004
Wong 2013

Total (Wald
a
)
Total events:
Test for overall effect: Z = 3.91 (P < 0.0001)

Heterogeneity: Tau² (REML
b
) = 0.00; Chi² = 0.75, df = 1 (P = 0.39); I² = 0%

Telephone reminder
Events
31
59
90
Total
256
480

736

No reminder
Events
11
35
46
Total
242
472

714
Weight
38.3%
61.7%

100.0%

Risk Difference
IV, Random, 95% CI
0.08 [0.03 , 0.12]
0.05 [0.01 , 0.09]

0.06 [0.03 , 0.09]

Risk Difference
IV, Random, 95% CI


-1

-0.5

0

0.5

1


Favours no reminder

Favours reminder


Risk of Bias
A

+

+
B

+

+
C

+

+
D

+

+
E

+

+
F

+

+
G

+

+
H

+

+

Footnotes

a
CI calculated by Wald-type method.

b
Tau² calculated by Restricted Maximum-Likelihood method.
Risk of bias legend

(A) Random sequence generation ok?

(B) Allocation concealment?

(C) Blinding of participants and personnel ok?

(D) Blinding of outcome assessment ok?

(E) Incomplete outcome data handled ok?

(F) Free of selective reporting?

(G) Was the study free of other bias?

(H) Overall bias?


Analysis 8.1: Participants recruited


Study or Subgroup
Free 2011

Total
Total events:
Test for overall effect: Z = 2.41 (P = 0.02)

Heterogeneity: Not applicable

SMS with scarcity
Events
90
90
Total
895

895

SMS without scarcity
Events
67
67
Total
967

967
Weight
100.0%

100.0%

Risk Difference
M-H, Fixed, 95% CI
0.03 [0.01 , 0.06]

0.03 [0.01 , 0.06]

Risk Difference
M-H, Fixed, 95% CI


-1

-0.5

0

0.5

1


Favours SMS no scarcity

Favours SMS + scarcity


Risk of Bias
A

+
B

+
C

+
D

+
E

+
F

+
G

+
H

+

Risk of bias legend

(A) Random sequence generation ok?

(B) Allocation concealment?

(C) Blinding of participants and personnel ok?

(D) Blinding of outcome assessment ok?

(E) Incomplete outcome data handled ok?

(F) Free of selective reporting?

(G) Was the study free of other bias?

(H) Overall bias?


Analysis 9.1: Participants recruited


Study or Subgroup
Free 2010

Total
Total events:
Test for overall effect: Z = 4.10 (P < 0.0001)

Heterogeneity: Not applicable

SMS
Events
17
17
Total
405

405

No SMS
Events
0
0
Total
406

406
Weight
100.0%

100.0%

Risk Difference
M-H, Fixed, 95% CI
0.04 [0.02 , 0.06]

0.04 [0.02 , 0.06]

Risk Difference
M-H, Fixed, 95% CI


-1

-0.5

0

0.5

1


Favours no SMS

Favours SMS


Risk of Bias
A

+
B

+
C

+
D

+
E

+
F

+
G

+
H

+

Risk of bias legend

(A) Random sequence generation ok?

(B) Allocation concealment?

(C) Blinding of participants and personnel ok?

(D) Blinding of outcome assessment ok?

(E) Incomplete outcome data handled ok?

(F) Free of selective reporting?

(G) Was the study free of other bias?

(H) Overall bias?


Analysis 10.1: Participants recruited


Study or Subgroup
Treweek 2012

Total
Total events:
Test for overall effect: Z = 0.40 (P = 0.69)

Heterogeneity: Not applicable

Email
Events
138
138
Total
880

880

Postal
Events
132
132
Total
880

880
Weight
100.0%

100.0%

Risk Difference
M-H, Fixed, 95% CI
0.01 [-0.03 , 0.04]

0.01 [-0.03 , 0.04]

Risk Difference
M-H, Fixed, 95% CI


-1

-0.5

0

0.5

1


Favours postal

Favours email


Risk of Bias
A

+
B

+
C

+
D

+
E

+
F

+
G

+
H

+

Risk of bias legend

(A) Random sequence generation ok?

(B) Allocation concealment?

(C) Blinding of participants and personnel ok?

(D) Blinding of outcome assessment ok?

(E) Incomplete outcome data handled ok?

(F) Free of selective reporting?

(G) Was the study free of other bias?

(H) Overall bias?


Analysis 11.1: Participants recruited


Study or Subgroup
Bracken 2019

Total
Total events:
Test for overall effect: Z = 1.67 (P = 0.09)

Heterogeneity: Not applicable

Telephone
Events
80
80
Total
355

355

SMS
Events
62
62
Total
354

354
Weight
100.0%

100.0%

Risk Difference
M-H, Fixed, 95% CI
0.05 [-0.01 , 0.11]

0.05 [-0.01 , 0.11]

Risk Difference
M-H, Fixed, 95% CI


-1

-0.5

0

0.5

1


Favours SMS

Favours Telephone


Risk of Bias
A

+
B

+
C

+
D

+
E

+
F

+
G

+
H

+

Risk of bias legend

(A) Random sequence generation ok?

(B) Allocation concealment?

(C) Blinding of participants and personnel ok?

(D) Blinding of outcome assessment ok?

(E) Incomplete outcome data handled ok?

(F) Free of selective reporting?

(G) Was the study free of other bias?

(H) Overall bias?


Analysis 12.1: Participants recruited


Study or Subgroup
Trevena 2006

Total
Total events:
Test for overall effect: Z = 2.35 (P = 0.02)

Heterogeneity: Not applicable

Opt-out
Events
40
40
Total
60

60

Opt-in
Events
44
44
Total
92

92
Weight
100.0%

100.0%

Risk Difference
M-H, Fixed, 95% CI
0.19 [0.03 , 0.35]

0.19 [0.03 , 0.35]

Risk Difference
M-H, Fixed, 95% CI


-4

-2

0

2

4


Favours opt-in

Favours opt-out


Risk of Bias
A

+
B

?
C

+
D

+
E

+
F

+
G

+
H

?

Risk of bias legend

(A) Random sequence generation ok?

(B) Allocation concealment?

(C) Blinding of participants and personnel ok?

(D) Blinding of outcome assessment ok?

(E) Incomplete outcome data handled ok?

(F) Free of selective reporting?

(G) Was the study free of other bias?

(H) Overall bias?


Analysis 13.1: Participants recruited


Study or Subgroup
Wadland 1990

Total
Total events:
Test for overall effect: Z = 0.59 (P = 0.56)

Heterogeneity: Not applicable

Researcher reads
Events
27
27
Total
51

51

Participant reads
Events
25
25
Total
53

53
Weight
100.0%

100.0%

Risk Difference
M-H, Fixed, 95% CI
0.06 [-0.13 , 0.25]

0.06 [-0.13 , 0.25]

Risk Difference
M-H, Fixed, 95% CI


-4

-2

0

2

4


Favours participant

Favours researcher


Risk of Bias
A

?
B

?
C

?
D

+
E

+
F

+
G

+
H

?

Risk of bias legend

(A) Random sequence generation ok?

(B) Allocation concealment?

(C) Blinding of participants and personnel ok?

(D) Blinding of outcome assessment ok?

(E) Incomplete outcome data handled ok?

(F) Free of selective reporting?

(G) Was the study free of other bias?

(H) Overall bias?


Analysis 15.1: Participants recruited


Study or Subgroup
Abd-Elsayed 2012

Total
Total events:
Test for overall effect: Z = 2.26 (P = 0.02)

Heterogeneity: Not applicable

Heavyweight cream paper
Events
164
164
Total
248

248

Standard
Events
189
189
Total
251

251
Weight
100.0%

100.0%

Risk Difference
M-H, Fixed, 95% CI
-0.09 [-0.17 , -0.01]

-0.09 [-0.17 , -0.01]

Risk Difference
M-H, Fixed, 95% CI


-1

-0.5

0

0.5

1


Favours standard

Favours heavyweight paper


Risk of Bias
A

+
B

+
C

?
D

+
E

+
F

+
G

−
H

−

Risk of bias legend

(A) Random sequence generation ok?

(B) Allocation concealment?

(C) Blinding of participants and personnel ok?

(D) Blinding of outcome assessment ok?

(E) Incomplete outcome data handled ok?

(F) Free of selective reporting?

(G) Was the study free of other bias?

(H) Overall bias?


Analysis 16.1: Participants recruited


Study or Subgroup
Lipstein 2021

Total
Total events:
Test for overall effect: Z = 0.27 (P = 0.79)

Heterogeneity: Not applicable

Enhanced pre-consent
Events
134
134
Total
394

394

Standard consent
Events
131
131
Total
375

375
Weight
100.0%

100.0%

Risk Difference
M-H, Fixed, 95% CI
-0.01 [-0.08 , 0.06]

-0.01 [-0.08 , 0.06]

Risk Difference
M-H, Fixed, 95% CI


-1

-0.5

0

0.5

1


Favours standard 

Favours enhanced pre-consent


Risk of Bias
A

+
B

+
C

+
D

+
E

+
F

?
G

−
H

−

Risk of bias legend

(A) Random sequence generation ok?

(B) Allocation concealment?

(C) Blinding of participants and personnel ok?

(D) Blinding of outcome assessment ok?

(E) Incomplete outcome data handled ok?

(F) Free of selective reporting?

(G) Was the study free of other bias?

(H) Overall bias?


Analysis 17.1: Participants recruited


Study or Subgroup
Brierley 2012
Chen 2011

Total (Wald
a
)
Total events:
Test for overall effect: Z = 0.23 (P = 0.82)

Heterogeneity: Tau² (REML
b
) = 0.00; Chi² = 0.15, df = 1 (P = 0.69); I² = 0%

Brief PIL
Events
63
720
783
Total
1165
1181

2346

Full PIL
Events
59
690
749
Total
1165
1122

2287
Weight
82.9%
17.1%

100.0%

Risk Difference
IV, Random, 95% CI
0.00 [-0.01 , 0.02]
-0.01 [-0.05 , 0.03]

0.00 [-0.01 , 0.02]

Risk Difference
IV, Random, 95% CI


-1

-0.5

0

0.5

1


Favours full PIL

Favours brief PIL


Risk of Bias
A

+

+
B

+

+
C

+

+
D

+

+
E

+

+
F

+

+
G

+

+
H

+

+

Footnotes

a
CI calculated by Wald-type method.

b
Tau² calculated by Restricted Maximum-Likelihood method.
Risk of bias legend

(A) Random sequence generation ok?

(B) Allocation concealment?

(C) Blinding of participants and personnel ok?

(D) Blinding of outcome assessment ok?

(E) Incomplete outcome data handled ok?

(F) Free of selective reporting?

(G) Was the study free of other bias?

(H) Overall bias?


Analysis 18.1: Participants recruited


Study or Subgroup
Kendrick 2001

Total
Total events:
Test for overall effect: Z = 3.27 (P = 0.001)

Heterogeneity: Not applicable

Study questionnaire
Events
217
217
Total
1203

1203

No study questionnaire
Events
157
157
Total
1190

1190
Weight
100.0%

100.0%

Risk Difference
M-H, Fixed, 95% CI
0.05 [0.02 , 0.08]

0.05 [0.02 , 0.08]

Risk Difference
M-H, Fixed, 95% CI


-1

-0.5

0

0.5

1


Favours no questionnaire

Favours questionnaire


Risk of Bias
A

+
B

+
C

+
D

+
E

+
F

+
G

+
H

+

Risk of bias legend

(A) Random sequence generation ok?

(B) Allocation concealment?

(C) Blinding of participants and personnel ok?

(D) Blinding of outcome assessment ok?

(E) Incomplete outcome data handled ok?

(F) Free of selective reporting?

(G) Was the study free of other bias?

(H) Overall bias?


Analysis 19.1: Participants recruited


Study or Subgroup
Chen 2011
Cockayne 2017 (START PIL REFORM)

Total (Wald
a
)
Total events:
Test for overall effect: Z = 1.26 (P = 0.21)

Heterogeneity: Tau² (REML
b
) = 0.00; Chi² = 0.16, df = 1 (P = 0.69); I² = 0%

PIL plus feedback
Events
373
68
441
Total
6104
2301

8405

Usual PIL
Events
339
62
401
Total
6060
2298

8358
Weight
56.9%
43.1%

100.0%

Risk Difference
IV, Random, 95% CI
0.01 [-0.00 , 0.01]
0.00 [-0.01 , 0.01]

0.00 [-0.00 , 0.01]

Risk Difference
IV, Random, 95% CI


-0.5

-0.25

0

0.25

0.5


Favours usual

Favours template


Risk of Bias
A

+

+
B

+

+
C

+

+
D

+

+
E

+

+
F

+

+
G

+

+
H

+

+

Footnotes

a
CI calculated by Wald-type method.

b
Tau² calculated by Restricted Maximum-Likelihood method.
Risk of bias legend

(A) Random sequence generation ok?

(B) Allocation concealment?

(C) Blinding of participants and personnel ok?

(D) Blinding of outcome assessment ok?

(E) Incomplete outcome data handled ok?

(F) Free of selective reporting?

(G) Was the study free of other bias?

(H) Overall bias?


Analysis 20.1: Participants recruited


Study or Subgroup
Ford 2004

Total
Total events:
Test for overall effect: Z = 2.28 (P = 0.02)

Heterogeneity: Not applicable

Enhanced+churches
Events
116
116
Total
2949

2949

Standard
Events
95
95
Total
3297

3297
Weight
100.0%

100.0%

Risk Difference
M-H, Fixed, 95% CI
0.01 [0.00 , 0.02]

0.01 [0.00 , 0.02]

Risk Difference
M-H, Fixed, 95% CI


-0.5

-0.25

0

0.25

0.5


Favours standard

Favours enhanced+churches


Risk of Bias
A

?
B

?
C

?
D

+
E

+
F

+
G

+
H

?

Risk of bias legend

(A) Random sequence generation ok?

(B) Allocation concealment?

(C) Blinding of participants and personnel ok?

(D) Blinding of outcome assessment ok?

(E) Incomplete outcome data handled ok?

(F) Free of selective reporting?

(G) Was the study free of other bias?

(H) Overall bias?


Analysis 21.1: Participants recruited


Study or Subgroup
Ford 2004

Total
Total events:
Test for overall effect: Z = 0.86 (P = 0.39)

Heterogeneity: Not applicable

Enhanced
Events
78
78
Total
3079

3079

Standard
Events
95
95
Total
3297

3297
Weight
100.0%

100.0%

Risk Difference
M-H, Fixed, 95% CI
-0.00 [-0.01 , 0.00]

-0.00 [-0.01 , 0.00]

Risk Difference
M-H, Fixed, 95% CI


-0.5

-0.25

0

0.25

0.5


Favours standard

Favours enhanced


Risk of Bias
A

?
B

?
C

?
D

+
E

+
F

+
G

+
H

?

Risk of bias legend

(A) Random sequence generation ok?

(B) Allocation concealment?

(C) Blinding of participants and personnel ok?

(D) Blinding of outcome assessment ok?

(E) Incomplete outcome data handled ok?

(F) Free of selective reporting?

(G) Was the study free of other bias?

(H) Overall bias?


Analysis 22.1: Participants recruited


Study or Subgroup
Ford 2004

Total
Total events:
Test for overall effect: Z = 0.12 (P = 0.90)

Heterogeneity: Not applicable

Enhanced+phone
Events
87
87
Total
3075

3075

Standard
Events
95
95
Total
3297

3297
Weight
100.0%

100.0%

Risk Difference
M-H, Fixed, 95% CI
-0.00 [-0.01 , 0.01]

-0.00 [-0.01 , 0.01]

Risk Difference
M-H, Fixed, 95% CI


-0.5

-0.25

0

0.25

0.5


Favours standard

Favours enhanced+phone


Risk of Bias
A

?
B

?
C

?
D

+
E

+
F

+
G

+
H

?

Risk of bias legend

(A) Random sequence generation ok?

(B) Allocation concealment?

(C) Blinding of participants and personnel ok?

(D) Blinding of outcome assessment ok?

(E) Incomplete outcome data handled ok?

(F) Free of selective reporting?

(G) Was the study free of other bias?

(H) Overall bias?


Analysis 23.1: Participants recruited


Study or Subgroup
Bergenmar 2014

Total
Total events:
Test for overall effect: Z = 0.35 (P = 0.73)

Heterogeneity: Not applicable

Audio recording
Events
46
46
Total
67

67

No audio recording
Events
45
45
Total
63

63
Weight
100.0%

100.0%

Risk Difference
M-H, Fixed, 95% CI
-0.03 [-0.19 , 0.13]

-0.03 [-0.19 , 0.13]

Risk Difference
M-H, Fixed, 95% CI


-4

-2

0

2

4


Favours no audio

Favours audio


Risk of Bias
A

?
B

?
C

+
D

+
E

+
F

+
G

+
H

?

Risk of bias legend

(A) Random sequence generation ok?

(B) Allocation concealment?

(C) Blinding of participants and personnel ok?

(D) Blinding of outcome assessment ok?

(E) Incomplete outcome data handled ok?

(F) Free of selective reporting?

(G) Was the study free of other bias?

(H) Overall bias?


Analysis 24.1: Participants recruited


Study or Subgroup
Ives 2001

Total
Total events:
Test for overall effect: Z = 1.58 (P = 0.11)

Heterogeneity: Not applicable

Booklet
Events
15
15
Total
16

16

Standard
Events
11
11
Total
15

15
Weight
100.0%

100.0%

Risk Difference
M-H, Fixed, 95% CI
0.20 [-0.05 , 0.46]

0.20 [-0.05 , 0.46]

Risk Difference
M-H, Fixed, 95% CI


-4

-2

0

2

4


Favours standard

Favours booklet


Risk of Bias
A

+
B

+
C

+
D

+
E

?
F

+
G

+
H

?

Risk of bias legend

(A) Random sequence generation ok?

(B) Allocation concealment?

(C) Blinding of participants and personnel ok?

(D) Blinding of outcome assessment ok?

(E) Incomplete outcome data handled ok?

(F) Free of selective reporting?

(G) Was the study free of other bias?

(H) Overall bias?


Analysis 25.1: Participants recruited


Study or Subgroup
Simes 1986

Total
Total events:
Test for overall effect: Z = 1.27 (P = 0.20)

Heterogeneity: Not applicable

Total disclosure
Events
27
27
Total
29

29

Standard
Events
23
23
Total
28

28
Weight
100.0%

100.0%

Risk Difference
M-H, Fixed, 95% CI
0.11 [-0.06 , 0.28]

0.11 [-0.06 , 0.28]

Risk Difference
M-H, Fixed, 95% CI


-4

-2

0

2

4


Favours standard

Favours total disclosure


Risk of Bias
A

+
B

?
C

?
D

+
E

+
F

+
G

+
H

?

Risk of bias legend

(A) Random sequence generation ok?

(B) Allocation concealment?

(C) Blinding of participants and personnel ok?

(D) Blinding of outcome assessment ok?

(E) Incomplete outcome data handled ok?

(F) Free of selective reporting?

(G) Was the study free of other bias?

(H) Overall bias?


Analysis 26.1: Participants recruited


Study or Subgroup
Dear 2011

Total
Total events:
Test for overall effect: Z = 0.22 (P = 0.83)

Heterogeneity: Not applicable

Website
Events
14
14
Total
146

146

No access
Events
20
20
Total
194

194
Weight
100.0%

100.0%

Risk Difference
M-H, Fixed, 95% CI
-0.01 [-0.07 , 0.06]

-0.01 [-0.07 , 0.06]

Risk Difference
M-H, Fixed, 95% CI


-1

-0.5

0

0.5

1


Favours no access

Favours website


Risk of Bias
A

+
B

+
C

+
D

+
E

−
F

+
G

+
H

−

Risk of bias legend

(A) Random sequence generation ok?

(B) Allocation concealment?

(C) Blinding of participants and personnel ok?

(D) Blinding of outcome assessment ok?

(E) Incomplete outcome data handled ok?

(F) Free of selective reporting?

(G) Was the study free of other bias?

(H) Overall bias?


Analysis 27.1: Participants recruited


Study or Subgroup
Cockayne 2017 (START PIL REFORM)
Knapp 2020 (START PIS CASPER)
Madurasinghe 2021 (START PIS HeLP Diabetes)
Man 2015 (START PIS Healthlines Depression)
Parker 2018 (START PIS ECLS)
Sheridan 2020 (START PIS ISDR)

Total (Wald
a
)
Total events:
Test for overall effect: Z = 0.61 (P = 0.54)

Heterogeneity: Tau² (REML
b
) = 0.00; Chi² = 3.99, df = 5 (P = 0.55); I² = 0%

Experimental
Events
63
116
183
43
180
422
1007
Total
2301
5765
2510
682
1136
1666

14060

Usual PIL
Events
62
113
166
27
176
393
937
Total
2298
5766
2370
682
1126
1503

13745
Weight
19.3%
65.7%
8.2%
3.1%
1.9%
1.8%

100.0%

Risk Difference
IV, Random, 95% CI
0.00 [-0.01 , 0.01]
0.00 [-0.00 , 0.01]
0.00 [-0.01 , 0.02]
0.02 [0.00 , 0.05]
0.00 [-0.03 , 0.03]
-0.01 [-0.04 , 0.02]

0.00 [-0.00 , 0.01]

Risk Difference
IV, Random, 95% CI


-0.5

-0.25

0

0.25

0.5


Favours usual

Favours bespoke


Risk of Bias
A

+

+

+

+

+

+
B

+

+

+

+

+

+
C

+

+

+

+

?

−
D

+

+

+

+

?

−
E

+

+

+

+

+

+
F

+

+

+

+

+

+
G

+

+

+

+

+

+
H

+

+

+

+

?

−

Footnotes

a
CI calculated by Wald-type method.

b
Tau² calculated by Restricted Maximum-Likelihood method.
Risk of bias legend

(A) Random sequence generation ok?

(B) Allocation concealment?

(C) Blinding of participants and personnel ok?

(D) Blinding of outcome assessment ok?

(E) Incomplete outcome data handled ok?

(F) Free of selective reporting?

(G) Was the study free of other bias?

(H) Overall bias?


Analysis 28.1: Participants recruited


Study or Subgroup
Arundel 2017
Paul 2014

Total (Wald
a
)
Total events:
Test for overall effect: Z = 0.97 (P = 0.33)

Heterogeneity: Tau² (REML
b
) = 0.00; Chi² = 0.10, df = 1 (P = 0.75); I² = 0%

Primer letter
Events
73
207
280
Total
1436
519

1955

No letter
Events
126
218
344
Total
2878
543

3421
Weight
94.9%
5.1%

100.0%

Risk Difference
IV, Random, 95% CI
0.01 [-0.01 , 0.02]
-0.00 [-0.06 , 0.06]

0.01 [-0.01 , 0.02]

Risk Difference
IV, Random, 95% CI


-0.5

-0.25

0

0.25

0.5


Favours no letter/leaflet

Favours primer letter/leaflet


Risk of Bias
A

+

+
B

+

+
C

+

+
D

+

+
E

+

+
F

+

+
G

+

+
H

+

+

Footnotes

a
CI calculated by Wald-type method.

b
Tau² calculated by Restricted Maximum-Likelihood method.
Risk of bias legend

(A) Random sequence generation ok?

(B) Allocation concealment?

(C) Blinding of participants and personnel ok?

(D) Blinding of outcome assessment ok?

(E) Incomplete outcome data handled ok?

(F) Free of selective reporting?

(G) Was the study free of other bias?

(H) Overall bias?


Analysis 29.1: Participants recruited


Study or Subgroup
Bobb 2016
Foss 2016

Total (Wald
a
)
Total events:
Test for overall effect: Z = 1.79 (P = 0.07)

Heterogeneity: Tau² (REML
b
) = 0.00; Chi² = 0.30, df = 1 (P = 0.58); I² = 0%

Information by telephone
Events
36
50
86
Total
64
59

123

Information face-to-face
Events
46
54
100
Total
67
59

126
Weight
33.2%
66.8%

100.0%

Risk Difference
IV, Random, 95% CI
-0.12 [-0.29 , 0.04]
-0.07 [-0.18 , 0.05]

-0.09 [-0.18 , 0.01]

Risk Difference
IV, Random, 95% CI


-1

-0.5

0

0.5

1


Favours face-to-face

Favours telephone


Risk of Bias
A

+

+
B

+

+
C

+

+
D

+

+
E

+

+
F

+

+
G

+

+
H

+

+

Footnotes

a
CI calculated by Wald-type method.

b
Tau² calculated by Restricted Maximum-Likelihood method.
Risk of bias legend

(A) Random sequence generation ok?

(B) Allocation concealment?

(C) Blinding of participants and personnel ok?

(D) Blinding of outcome assessment ok?

(E) Incomplete outcome data handled ok?

(F) Free of selective reporting?

(G) Was the study free of other bias?

(H) Overall bias?


Analysis 30.1: Participants recruited


Study or Subgroup
Du 2008
Du 2009
Hutchison 2007
Mattock 2020
Rogers 2019

Total (HKSJ
a
)
Total events:
Test for overall effect: T = 0.57, df = 4 (P = 0.60)

Heterogeneity: Tau² (REML
b
) = 0.02; Chi² = 17.79, df = 4 (P = 0.001); I² = 88%

Video information
Events
16
10
62
10
114
212
Total
63
98
86
56
501

804

Usual information
Events
10
6
66
26
129
237
Total
63
98
87
51
531

830
Weight
18.6%
22.1%
19.2%
16.9%
23.1%

100.0%

Risk Difference
IV, Random, 95% CI
0.10 [-0.05 , 0.24]
0.04 [-0.04 , 0.12]
-0.04 [-0.17 , 0.09]
-0.33 [-0.50 , -0.16]
-0.02 [-0.07 , 0.04]

-0.04 [-0.23 , 0.15]

Risk Difference
IV, Random, 95% CI


-1

-0.5

0

0.5

1


Favours usual information

Favours video information


Risk of Bias
A

?

?

+

−

+
B

?

?

+

−

?
C

+

?

+

?

+
D

+

+

+

+

+
E

+

+

+

+

?
F

+

+

+

+

+
G

+

+

+

−

+
H

?

?

+

−

?

Footnotes

a
CI calculated by Hartung-Knapp-Sidik-Jonkman (HKSJ) method.

b
Tau² calculated by Restricted Maximum-Likelihood method.
Risk of bias legend

(A) Random sequence generation ok?

(B) Allocation concealment?

(C) Blinding of participants and personnel ok?

(D) Blinding of outcome assessment ok?

(E) Incomplete outcome data handled ok?

(F) Free of selective reporting?

(G) Was the study free of other bias?

(H) Overall bias?


Analysis 31.1: Participants recruited


Study or Subgroup
Brown 2015

Total
Total events:
Test for overall effect: Z = 0.70 (P = 0.48)

Heterogeneity: Not applicable

E-S risk letter
Events
65
65
Total
216

216

Standard letter
Events
76
76
Total
229

229
Weight
100.0%

100.0%

Risk Difference
M-H, Fixed, 95% CI
-0.03 [-0.12 , 0.06]

-0.03 [-0.12 , 0.06]

Risk Difference
M-H, Fixed, 95% CI


-1

-0.5

0

0.5

1


Favours standard letter

Favours E-S risk letter


Risk of Bias
A

+
B

+
C

+
D

+
E

+
F

+
G

+
H

+

Risk of bias legend

(A) Random sequence generation ok?

(B) Allocation concealment?

(C) Blinding of participants and personnel ok?

(D) Blinding of outcome assessment ok?

(E) Incomplete outcome data handled ok?

(F) Free of selective reporting?

(G) Was the study free of other bias?

(H) Overall bias?


Analysis 32.1: Participants recruited


Study or Subgroup
McCaffery 2019

Total
Total events:
Test for overall effect: Z = 1.78 (P = 0.07)

Heterogeneity: Not applicable

HW personalised letter
Events
3
3
Total
159

159

Standard letter
Events
9
9
Total
158

158
Weight
100.0%

100.0%

Risk Difference
M-H, Fixed, 95% CI
-0.04 [-0.08 , 0.00]

-0.04 [-0.08 , 0.00]

Risk Difference
M-H, Fixed, 95% CI


-1

-0.5

0

0.5

1


Favours standard letter

Favours HW personalised letter


Risk of Bias
A

+
B

+
C

?
D

?
E

+
F

+
G

+
H

?

Risk of bias legend

(A) Random sequence generation ok?

(B) Allocation concealment?

(C) Blinding of participants and personnel ok?

(D) Blinding of outcome assessment ok?

(E) Incomplete outcome data handled ok?

(F) Free of selective reporting?

(G) Was the study free of other bias?

(H) Overall bias?


Analysis 32.2: Participants retained


Study or Subgroup
McCaffery 2019

Total
Total events:
Test for overall effect: Z = 1.95 (P = 0.05)

Heterogeneity: Not applicable

HW personalised letter
Events
2
2
Total
159

159

Standard letter
Events
8
8
Total
158

158
Weight
100.0%

100.0%

Risk Difference
M-H, Fixed, 95% CI
-0.04 [-0.08 , 0.00]

-0.04 [-0.08 , 0.00]

Risk Difference
M-H, Fixed, 95% CI


-0.5

-0.25

0

0.25

0.5


Favours standard letter

Favours HW personalised letter


Risk of Bias
A

+
B

+
C

?
D

?
E

+
F

+
G

+
H

?

Risk of bias legend

(A) Random sequence generation ok?

(B) Allocation concealment?

(C) Blinding of participants and personnel ok?

(D) Blinding of outcome assessment ok?

(E) Incomplete outcome data handled ok?

(F) Free of selective reporting?

(G) Was the study free of other bias?

(H) Overall bias?


Analysis 33.1: Participants recruited


Study or Subgroup
Miller 2021a
Miller 2021b

Total (Wald
a
)
Total events:
Test for overall effect: Z = 0.02 (P = 0.98)

Heterogeneity: Tau² (REML
b
) = 0.00; Chi² = 0.03, df = 1 (P = 0.85); I² = 0%

New wording
Events
17
18
35
Total
920
871

1791

Standard wording
Events
8
8
16
Total
407
407

814
Weight
51.3%
48.7%

100.0%

Risk Difference
IV, Random, 95% CI
-0.00 [-0.02 , 0.01]
0.00 [-0.02 , 0.02]

-0.00 [-0.01 , 0.01]

Risk Difference
IV, Random, 95% CI


-0.5

-0.25

0

0.25

0.5


Favours standard wording

Favours new wording


Risk of Bias
A

+

+
B

+

+
C

+

+
D

+

+
E

+

+
F

+

+
G

−

−
H

−

−

Footnotes

a
CI calculated by Wald-type method.

b
Tau² calculated by Restricted Maximum-Likelihood method.
Risk of bias legend

(A) Random sequence generation ok?

(B) Allocation concealment?

(C) Blinding of participants and personnel ok?

(D) Blinding of outcome assessment ok?

(E) Incomplete outcome data handled ok?

(F) Free of selective reporting?

(G) Was the study free of other bias?

(H) Overall bias?


Analysis 34.1: Participants recruited


Study or Subgroup
Chow 2020

Total
Total events:
Test for overall effect: Z = 0.29 (P = 0.78)

Heterogeneity: Not applicable

Shorter recruitment pack with standrd consent
Events
36
36
Total
84

84

Standard recruitment pack
Events
35
35
Total
86

86
Weight
100.0%

100.0%

Risk Difference
M-H, Fixed, 95% CI
0.02 [-0.13 , 0.17]

0.02 [-0.13 , 0.17]

Risk Difference
M-H, Fixed, 95% CI


-1

-0.5

0

0.5

1


Favours standard pack

Favours shorter, standard consent 


Risk of Bias
A

?
B

?
C

?
D

+
E

+
F

+
G

+
H

?

Risk of bias legend

(A) Random sequence generation ok?

(B) Allocation concealment?

(C) Blinding of participants and personnel ok?

(D) Blinding of outcome assessment ok?

(E) Incomplete outcome data handled ok?

(F) Free of selective reporting?

(G) Was the study free of other bias?

(H) Overall bias?


Analysis 35.1: Participants recruited


Study or Subgroup
Chow 2020

Total
Total events:
Test for overall effect: Z = 0.27 (P = 0.79)

Heterogeneity: Not applicable

Shorter recuirment pack + simplified consent
Events
38
38
Total
89

89

Standard recruitment pack
Events
35
35
Total
86

86
Weight
100.0%

100.0%

Risk Difference
M-H, Fixed, 95% CI
0.02 [-0.13 , 0.17]

0.02 [-0.13 , 0.17]

Risk Difference
M-H, Fixed, 95% CI


-1

-0.5

0

0.5

1


Favours standard pack

Favours shorter pack, simplifed consent


Risk of Bias
A

?
B

?
C

?
D

+
E

+
F

+
G

+
H

?

Risk of bias legend

(A) Random sequence generation ok?

(B) Allocation concealment?

(C) Blinding of participants and personnel ok?

(D) Blinding of outcome assessment ok?

(E) Incomplete outcome data handled ok?

(F) Free of selective reporting?

(G) Was the study free of other bias?

(H) Overall bias?


Analysis 36.1: Participants recruited


Study or Subgroup
Hughes-Morley 2016

Total
Total events:
Test for overall effect: Z = 2.54 (P = 0.01)

Heterogeneity: Not applicable

Leaflet advertising PPI involvement
Events
216
216
Total
5382

5382

Standard trial invtitation
Events
148
148
Total
2800

2800
Weight
100.0%

100.0%

Risk Difference
M-H, Fixed, 95% CI
-0.01 [-0.02 , -0.00]

-0.01 [-0.02 , -0.00]

Risk Difference
M-H, Fixed, 95% CI


-0.5

-0.25

0

0.25

0.5


Favours standard invitation

Favours PPI advertise leaflet


Risk of Bias
A

+
B

+
C

+
D

+
E

+
F

+
G

+
H

+

Risk of bias legend

(A) Random sequence generation ok?

(B) Allocation concealment?

(C) Blinding of participants and personnel ok?

(D) Blinding of outcome assessment ok?

(E) Incomplete outcome data handled ok?

(F) Free of selective reporting?

(G) Was the study free of other bias?

(H) Overall bias?


Analysis 37.1: Participants recruited


Study or Subgroup
Crane 2016

Total
Total events:
Test for overall effect: Z = 0.00 (P = 1.00)

Heterogeneity: Not applicable

Gender-targeted postcard
Events
15
15
Total
15000

15000

Generic postcard
Events
15
15
Total
15000

15000
Weight
100.0%

100.0%

Risk Difference
M-H, Fixed, 95% CI
0.00 [-0.00 , 0.00]

0.00 [-0.00 , 0.00]

Risk Difference
M-H, Fixed, 95% CI


-0.5

-0.25

0

0.25

0.5


Favours generic postcard

Favours gender-targeted postcard


Risk of Bias
A

+
B

?
C

?
D

?
E

?
F

+
G

?
H

?

Risk of bias legend

(A) Random sequence generation ok?

(B) Allocation concealment?

(C) Blinding of participants and personnel ok?

(D) Blinding of outcome assessment ok?

(E) Incomplete outcome data handled ok?

(F) Free of selective reporting?

(G) Was the study free of other bias?

(H) Overall bias?


Analysis 38.1: Participants recruited


Study or Subgroup
Paris 2015

Total
Total events:
Test for overall effect: Z = 2.04 (P = 0.04)

Heterogeneity: Not applicable

Researcher optimised PIL
Events
154
154
Total
239

239

Standard PIL
Events
176
176
Total
241

241
Weight
100.0%

100.0%

Risk Difference
M-H, Fixed, 95% CI
-0.09 [-0.17 , -0.00]

-0.09 [-0.17 , -0.00]

Risk Difference
M-H, Fixed, 95% CI


-1

-0.5

0

0.5

1


Favours standard PIL

Favours researcher PIL


Risk of Bias
A

+
B

+
C

+
D

+
E

+
F

+
G

+
H

+

Risk of bias legend

(A) Random sequence generation ok?

(B) Allocation concealment?

(C) Blinding of participants and personnel ok?

(D) Blinding of outcome assessment ok?

(E) Incomplete outcome data handled ok?

(F) Free of selective reporting?

(G) Was the study free of other bias?

(H) Overall bias?


Analysis 39.1: Participants recruited


Study or Subgroup
Skinner 2019

Total
Total events:
Test for overall effect: Z = 0.60 (P = 0.55)

Heterogeneity: Not applicable

In-clinic video
Events
3
3
Total
37

37

Take home DVD
Events
2
2
Total
42

42
Weight
100.0%

100.0%

Risk Difference
M-H, Fixed, 95% CI
0.03 [-0.08 , 0.14]

0.03 [-0.08 , 0.14]

Risk Difference
M-H, Fixed, 95% CI


-1

-0.5

0

0.5

1


Favours DVD

Favours in-clinic


Risk of Bias
A

+
B

?
C

−
D

+
E

+
F

+
G

+
H

−

Risk of bias legend

(A) Random sequence generation ok?

(B) Allocation concealment?

(C) Blinding of participants and personnel ok?

(D) Blinding of outcome assessment ok?

(E) Incomplete outcome data handled ok?

(F) Free of selective reporting?

(G) Was the study free of other bias?

(H) Overall bias?


Analysis 40.1: Participants recruited


Study or Subgroup
White 2023

Total
Total events:
Test for overall effect: Z = 0.84 (P = 0.40)

Heterogeneity: Not applicable

Brief PIS
Events
166
166
Total
3467

3467

Standard PIS
Events
152
152
Total
3479

3479
Weight
100.0%

100.0%

Risk Difference
M-H, Fixed, 95% CI
0.00 [-0.01 , 0.01]

0.00 [-0.01 , 0.01]

Risk Difference
M-H, Fixed, 95% CI


-0.5

-0.25

0

0.25

0.5


Favours standard PIS

Favours brief PIS


Risk of Bias
A

+
B

+
C

+
D

+
E

?
F

+
G

+
H

?

Risk of bias legend

(A) Random sequence generation ok?

(B) Allocation concealment?

(C) Blinding of participants and personnel ok?

(D) Blinding of outcome assessment ok?

(E) Incomplete outcome data handled ok?

(F) Free of selective reporting?

(G) Was the study free of other bias?

(H) Overall bias?


Analysis 41.1: Participant recruited


Study or Subgroup
Sundaresan 2017

Total
Total events:
Test for overall effect: Z = 1.86 (P = 0.06)

Heterogeneity: Not applicable

Decision Aid
Events
13
13
Total
63

63

Blank notebook
Events
6
6
Total
66

66
Weight
100.0%

100.0%

Risk Difference
M-H, Fixed, 95% CI
0.12 [-0.01 , 0.24]

0.12 [-0.01 , 0.24]

Risk Difference
M-H, Fixed, 95% CI


-1

-0.5

0

0.5

1


Favours blank notebook

Favours decision aid


Risk of Bias
A

+
B

+
C

+
D

+
E

+
F

+
G

+
H

+

Risk of bias legend

(A) Random sequence generation ok?

(B) Allocation concealment?

(C) Blinding of participants and personnel ok?

(D) Blinding of outcome assessment ok?

(E) Incomplete outcome data handled ok?

(F) Free of selective reporting?

(G) Was the study free of other bias?

(H) Overall bias?


Analysis 42.1: Participants recruited


Study or Subgroup
Saif 2020

Total
Total events:
Test for overall effect: Z = 1.16 (P = 0.25)

Heterogeneity: Not applicable

Interactive online tool
Events
2
2
Total
180

180

Time-matched webinar
Events
0
0
Total
171

171
Weight
100.0%

100.0%

Risk Difference
M-H, Fixed, 95% CI
0.01 [-0.01 , 0.03]

0.01 [-0.01 , 0.03]

Risk Difference
M-H, Fixed, 95% CI


-0.5

-0.25

0

0.25

0.5


Favours webinar

Favours online tool


Risk of Bias
A

+
B

+
C

?
D

?
E

+
F

+
G

+
H

?

Risk of bias legend

(A) Random sequence generation ok?

(B) Allocation concealment?

(C) Blinding of participants and personnel ok?

(D) Blinding of outcome assessment ok?

(E) Incomplete outcome data handled ok?

(F) Free of selective reporting?

(G) Was the study free of other bias?

(H) Overall bias?


Analysis 43.1: Participants recruited


Study or Subgroup
Dwyer 2022

Total
Total events:
Test for overall effect: Z = 0.23 (P = 0.82)

Heterogeneity: Not applicable

PPI designed and informed PIL
Events
62
62
Total
104

104

Standard researcher designe PIL
Events
63
63
Total
103

103
Weight
100.0%

100.0%

Risk Difference
M-H, Fixed, 95% CI
-0.02 [-0.15 , 0.12]

-0.02 [-0.15 , 0.12]

Risk Difference
M-H, Fixed, 95% CI


-1

-0.5

0

0.5

1


Favours standard PIL

Favours PPI PIL


Risk of Bias
A

+
B

+
C

+
D

+
E

?
F

+
G

+
H

?

Risk of bias legend

(A) Random sequence generation ok?

(B) Allocation concealment?

(C) Blinding of participants and personnel ok?

(D) Blinding of outcome assessment ok?

(E) Incomplete outcome data handled ok?

(F) Free of selective reporting?

(G) Was the study free of other bias?

(H) Overall bias?


Analysis 43.2: Participants retained


Study or Subgroup
Dwyer 2022

Total
Total events:
Test for overall effect: Z = 0.67 (P = 0.50)

Heterogeneity: Not applicable

PPI designed and informed PIL
Events
40
40
Total
104

104

Standard researcher designe PIL
Events
35
35
Total
103

103
Weight
100.0%

100.0%

Risk Difference
M-H, Fixed, 95% CI
0.04 [-0.09 , 0.18]

0.04 [-0.09 , 0.18]

Risk Difference
M-H, Fixed, 95% CI


-1

-0.5

0

0.5

1


Favours standard PIL

Favours PPI PIL


Risk of Bias
A

+
B

+
C

+
D

+
E

?
F

+
G

+
H

?

Risk of bias legend

(A) Random sequence generation ok?

(B) Allocation concealment?

(C) Blinding of participants and personnel ok?

(D) Blinding of outcome assessment ok?

(E) Incomplete outcome data handled ok?

(F) Free of selective reporting?

(G) Was the study free of other bias?

(H) Overall bias?


Analysis 44.1: Participants recruited


Study or Subgroup
Felicitas-Perkins 2017

Total
Total events:
Test for overall effect: Z = 0.29 (P = 0.77)

Heterogeneity: Not applicable

Culturally-tailored DVD on trials
Events
3
3
Total
20

20

Standard education
Events
2
2
Total
17

17
Weight
100.0%

100.0%

Risk Difference
M-H, Fixed, 95% CI
0.03 [-0.19 , 0.25]

0.03 [-0.19 , 0.25]

Risk Difference
M-H, Fixed, 95% CI


-1

-0.5

0

0.5

1


Favours standard education

Favours DVD


Risk of Bias
A

?
B

?
C

−
D

+
E

+
F

+
G

?
H

−

Risk of bias legend

(A) Random sequence generation ok?

(B) Allocation concealment?

(C) Blinding of participants and personnel ok?

(D) Blinding of outcome assessment ok?

(E) Incomplete outcome data handled ok?

(F) Free of selective reporting?

(G) Was the study free of other bias?

(H) Overall bias?


Analysis 45.1: Participants recruited


Study or Subgroup
Kamen 2018

Total
Total events:
Test for overall effect: Z = 0.92 (P = 0.36)

Heterogeneity: Not applicable

Multimedia education on trials
Events
117
117
Total
199

199

Standard educational booklet
Events
119
119
Total
219

219
Weight
100.0%

100.0%

Risk Difference
M-H, Fixed, 95% CI
0.04 [-0.05 , 0.14]

0.04 [-0.05 , 0.14]

Risk Difference
M-H, Fixed, 95% CI


-1

-0.5

0

0.5

1


Favours booklet

Favours multimedia


Risk of Bias
A

+
B

+
C

+
D

+
E

+
F

+
G

+
H

+

Risk of bias legend

(A) Random sequence generation ok?

(B) Allocation concealment?

(C) Blinding of participants and personnel ok?

(D) Blinding of outcome assessment ok?

(E) Incomplete outcome data handled ok?

(F) Free of selective reporting?

(G) Was the study free of other bias?

(H) Overall bias?


Analysis 46.1: Participants recruited


Study or Subgroup
Witham 2018

Total
Total events:
Test for overall effect: Z = 1.29 (P = 0.20)

Heterogeneity: Not applicable

PIL with minimal negative phrasing
Events
43
43
Total
2148

2148

Standard PIL
Events
52
52
Total
1997

1997
Weight
100.0%

100.0%

Risk Difference
M-H, Fixed, 95% CI
-0.01 [-0.02 , 0.00]

-0.01 [-0.02 , 0.00]

Risk Difference
M-H, Fixed, 95% CI


-0.5

-0.25

0

0.25

0.5


Favours standard PIL

Favours PIL minimising negative phrases


Risk of Bias
A

+
B

+
C

+
D

−
E

+
F

+
G

+
H

−

Risk of bias legend

(A) Random sequence generation ok?

(B) Allocation concealment?

(C) Blinding of participants and personnel ok?

(D) Blinding of outcome assessment ok?

(E) Incomplete outcome data handled ok?

(F) Free of selective reporting?

(G) Was the study free of other bias?

(H) Overall bias?


Analysis 47.1: Participant recruitment


Study or Subgroup
Knapp 2023a (BALANCE)
Knapp 2023b (BAMP)
Knapp 2023f (UKALL)

Total (HKSJ
a
)
Total events:
Test for overall effect: T = 0.69, df = 2 (P = 0.56)

Heterogeneity: Tau² (REML
b
) = 0.12; Chi² = 5.71, df = 2 (P = 0.06); I² = 64%

MMI PIL (in person) + paper PIL
Events
8
4
1
13
Total
9
4
1

14

Paper PIL
Events
4
1
2
7
Total
4
3
2

9
Weight
42.5%
32.7%
24.8%

100.0%

Risk Difference
IV, Random, 95% CI
-0.11 [-0.45 , 0.23]
0.67 [0.12 , 1.21]
0.00 [-0.73 , 0.73]

0.17 [-0.89 , 1.23]

Risk Difference
IV, Random, 95% CI


-2

-1

0

1

2


Favours paper PIL

Favours MMI + paper PIL


Risk of Bias
A

+

+

+
B

+

+

+
C

+

+

+
D

+

+

+
E

+

+

+
F

+

+

+
G

+

+

+
H

+

+

+

Footnotes

a
CI calculated by Hartung-Knapp-Sidik-Jonkman (HKSJ) method.

b
Tau² calculated by Restricted Maximum-Likelihood method.
Risk of bias legend

(A) Random sequence generation ok?

(B) Allocation concealment?

(C) Blinding of participants and personnel ok?

(D) Blinding of outcome assessment ok?

(E) Incomplete outcome data handled ok?

(F) Free of selective reporting?

(G) Was the study free of other bias?

(H) Overall bias?


Analysis 47.2: Participants retained


Study or Subgroup
Knapp 2023a (BALANCE)
Knapp 2023b (BAMP)
Knapp 2023f (UKALL)

Total (HKSJ
a
)
Total events:
Test for overall effect: T = 1.14, df = 2 (P = 0.37)

Heterogeneity: Tau² (REML
b
) = 0.11; Chi² = 4.68, df = 2 (P = 0.10); I² = 57%

MMI PIL (in person) + paper PIL
Events
7
3
1
11
Total
9
4
1

14

Paper PIL
Events
3
0
2
5
Total
4
3
2

9
Weight
37.2%
36.5%
26.3%

100.0%

Risk Difference
IV, Random, 95% CI
0.03 [-0.48 , 0.53]
0.75 [0.23 , 1.27]
0.00 [-0.73 , 0.73]

0.28 [-0.79 , 1.36]

Risk Difference
IV, Random, 95% CI


-2

-1

0

1

2


Favours paper PIL

Favours MMI + paper PIL


Risk of Bias
A

+

+

+
B

+

+

+
C

+

+

+
D

+

+

+
E

+

+

+
F

+

+

+
G

+

+

+
H

+

+

+

Footnotes

a
CI calculated by Hartung-Knapp-Sidik-Jonkman (HKSJ) method.

b
Tau² calculated by Restricted Maximum-Likelihood method.
Risk of bias legend

(A) Random sequence generation ok?

(B) Allocation concealment?

(C) Blinding of participants and personnel ok?

(D) Blinding of outcome assessment ok?

(E) Incomplete outcome data handled ok?

(F) Free of selective reporting?

(G) Was the study free of other bias?

(H) Overall bias?


Analysis 48.1: Participants recruited


Study or Subgroup
Knapp 2023a (BALANCE)
Knapp 2023b (BAMP)
Knapp 2023d (FORCE)

Total (Wald
a
)
Total events:
Test for overall effect: Z = 1.52 (P = 0.13)

Heterogeneity: Tau² (REML
b
) = 0.00; Chi² = 4.69, df = 2 (P = 0.10); I² = 0%

MMI (in person) PIL
Events
8
3
475
486
Total
8
3
681

692

Paper PIL
Events
4
1
484
489
Total
4
3
729

736
Weight
2.5%
0.7%
96.8%

100.0%

Risk Difference
IV, Random, 95% CI
0.00 [-0.30 , 0.30]
0.67 [0.09 , 1.24]
0.03 [-0.02 , 0.08]

0.04 [-0.01 , 0.08]

Risk Difference
IV, Random, 95% CI


-1

-0.5

0

0.5

1


Favours paper PIL

Favours MMI PIL


Risk of Bias
A

+

+

+
B

+

+

+
C

+

+

+
D

+

+

+
E

+

+

+
F

+

+

+
G

+

+

+
H

+

+

+

Footnotes

a
CI calculated by Wald-type method.

b
Tau² calculated by Restricted Maximum-Likelihood method.
Risk of bias legend

(A) Random sequence generation ok?

(B) Allocation concealment?

(C) Blinding of participants and personnel ok?

(D) Blinding of outcome assessment ok?

(E) Incomplete outcome data handled ok?

(F) Free of selective reporting?

(G) Was the study free of other bias?

(H) Overall bias?


Analysis 48.2: Participants retained


Study or Subgroup
Knapp 2023a (BALANCE)
Knapp 2023b (BAMP)
Knapp 2023d (FORCE)

Total (HKSJ
a
)
Total events:
Test for overall effect: T = 1.31, df = 2 (P = 0.32)

Heterogeneity: Tau² (REML
b
) = 0.06; Chi² = 5.53, df = 2 (P = 0.06); I² = 64%

MMI (in person) PIL
Events
8
2
473
483
Total
8
3
681

692

Paper PIL
Events
3
0
481
484
Total
4
3
729

736
Weight
28.0%
20.5%
51.5%

100.0%

Risk Difference
IV, Random, 95% CI
0.25 [-0.18 , 0.68]
0.67 [0.09 , 1.24]
0.03 [-0.01 , 0.08]

0.22 [-0.51 , 0.96]

Risk Difference
IV, Random, 95% CI


-2

-1

0

1

2


Favours paper PIL

Favours MMI PIL


Risk of Bias
A

+

+

+
B

+

+

+
C

+

+

+
D

+

+

+
E

+

+

+
F

+

+

+
G

+

+

+
H

+

+

+

Footnotes

a
CI calculated by Hartung-Knapp-Sidik-Jonkman (HKSJ) method.

b
Tau² calculated by Restricted Maximum-Likelihood method.
Risk of bias legend

(A) Random sequence generation ok?

(B) Allocation concealment?

(C) Blinding of participants and personnel ok?

(D) Blinding of outcome assessment ok?

(E) Incomplete outcome data handled ok?

(F) Free of selective reporting?

(G) Was the study free of other bias?

(H) Overall bias?


Analysis 49.1: Participants recruited


Study or Subgroup
Jolly 2019 (START MMI PSM-COPD)
Knapp 2023c (CHAMP-UK)
Knapp 2023e (Thermic-3)
Madurasinghe 2023a (START MMI GHT2000a)
Madurasinghe 2023b (START MMI GHT2000b)
Madurasinghe 2023c (START MMI HI-Light)
Madurasinghe 2023d (START MMI seAFOod)

Total (Wald
a
)
Total events:
Test for overall effect: Z = 0.83 (P = 0.41)

Heterogeneity: Tau² (REML
b
) = 0.00; Chi² = 5.97, df = 6 (P = 0.43); I² = 0%

MMI (QR code/link) PIL + paper PIL
Events
185
30
22
57
35
54
68
451
Total
1934
68
51
1048
1055
1094
333

5583

Paper PIL
Events
247
28
24
64
41
51
61
516
Total
2280
65
47
1049
1057
1136
395

6029
Weight
22.9%
0.3%
0.2%
19.2%
30.3%
24.7%
2.4%

100.0%

Risk Difference
IV, Random, 95% CI
-0.01 [-0.03 , 0.01]
0.01 [-0.16 , 0.18]
-0.08 [-0.28 , 0.12]
-0.01 [-0.03 , 0.01]
-0.01 [-0.02 , 0.01]
0.00 [-0.01 , 0.02]
0.05 [-0.01 , 0.11]

-0.00 [-0.01 , 0.01]

Risk Difference
IV, Random, 95% CI


-0.5

-0.25

0

0.25

0.5


Favours paper PIL

Favours MMI PIL + paper PIL


Risk of Bias
A

+

+

+

+

+

+

+
B

+

+

+

+

+

+

+
C

+

+

+

+

+

+

+
D

+

+

+

+

+

+

+
E

+

+

+

+

+

+

+
F

+

+

+

+

+

+

+
G

+

+

+

+

+

+

+
H

+

+

+

+

+

+

+

Footnotes

a
CI calculated by Wald-type method.

b
Tau² calculated by Restricted Maximum-Likelihood method.
Risk of bias legend

(A) Random sequence generation ok?

(B) Allocation concealment?

(C) Blinding of participants and personnel ok?

(D) Blinding of outcome assessment ok?

(E) Incomplete outcome data handled ok?

(F) Free of selective reporting?

(G) Was the study free of other bias?

(H) Overall bias?


Analysis 49.2: Participants retained


Study or Subgroup
Jolly 2019 (START MMI PSM-COPD)
Knapp 2023c (CHAMP-UK)
Knapp 2023e (Thermic-3)
Madurasinghe 2023c (START MMI HI-Light)
Madurasinghe 2023d (START MMI seAFOod)

Total (Wald
a
)
Total events:
Test for overall effect: Z = 0.28 (P = 0.78)

Heterogeneity: Tau² (REML
b
) = 0.00; Chi² = 7.04, df = 4 (P = 0.13); I² = 59%

MMI (QR code/link) PIL + paper PIL
Events
159
29
21
40
57
306
Total
1934
68
51
1094
333

3480

Paper PIL
Events
223
26
21
35
49
354
Total
2280
65
47
1136
395

3923
Weight
39.4%
2.1%
1.6%
41.3%
15.6%

100.0%

Risk Difference
IV, Random, 95% CI
-0.02 [-0.03 , 0.00]
0.03 [-0.14 , 0.19]
-0.04 [-0.23 , 0.16]
0.01 [-0.01 , 0.02]
0.05 [-0.00 , 0.10]

0.00 [-0.02 , 0.03]

Risk Difference
IV, Random, 95% CI


-1

-0.5

0

0.5

1


Favours paper PIL

Favours MMI PIL + paper PIL


Risk of Bias
A

+

+

+

+

+
B

+

+

+

+

+
C

+

+

+

+

+
D

+

+

+

+

+
E

+

+

+

+

+
F

+

+

+

+

+
G

+

+

+

+

+
H

+

+

+

+

+

Footnotes

a
CI calculated by Wald-type method.

b
Tau² calculated by Restricted Maximum-Likelihood method.
Risk of bias legend

(A) Random sequence generation ok?

(B) Allocation concealment?

(C) Blinding of participants and personnel ok?

(D) Blinding of outcome assessment ok?

(E) Incomplete outcome data handled ok?

(F) Free of selective reporting?

(G) Was the study free of other bias?

(H) Overall bias?


Analysis 50.1: Participants recruited


Study or Subgroup
Knapp 2023c (CHAMP-UK)
Knapp 2023e (Thermic-3)

Total (Wald
a
)
Total events:
Test for overall effect: Z = 2.06 (P = 0.04)

Heterogeneity: Tau² (REML
b
) = 0.00; Chi² = 0.01, df = 1 (P = 0.91); I² = 0%

MMI PIL (QR code/link)
Events
38
32
70
Total
68
49

117

Paper PIL
Events
28
24
52
Total
65
47

112
Weight
57.3%
42.7%

100.0%

Risk Difference
IV, Random, 95% CI
0.13 [-0.04 , 0.30]
0.14 [-0.05 , 0.34]

0.13 [0.01 , 0.26]

Risk Difference
IV, Random, 95% CI


-1

-0.5

0

0.5

1


Favours paper PIL

Favours MMI PIL


Risk of Bias
A

+

+
B

+

+
C

+

+
D

+

+
E

+

+
F

+

+
G

+

+
H

+

+

Footnotes

a
CI calculated by Wald-type method.

b
Tau² calculated by Restricted Maximum-Likelihood method.
Risk of bias legend

(A) Random sequence generation ok?

(B) Allocation concealment?

(C) Blinding of participants and personnel ok?

(D) Blinding of outcome assessment ok?

(E) Incomplete outcome data handled ok?

(F) Free of selective reporting?

(G) Was the study free of other bias?

(H) Overall bias?


Analysis 50.2: Participants retained


Study or Subgroup
Knapp 2023c (CHAMP-UK)
Knapp 2023e (Thermic-3)

Total (Wald
a
)
Total events:
Test for overall effect: Z = 1.95 (P = 0.05)

Heterogeneity: Tau² (REML
b
) = 0.00; Chi² = 0.25, df = 1 (P = 0.62); I² = 0%

MMI PIL (QR code/link)
Events
34
30
64
Total
68
49

117

Paper PIL
Events
26
21
47
Total
65
47

112
Weight
57.8%
42.2%

100.0%

Risk Difference
IV, Random, 95% CI
0.10 [-0.07 , 0.27]
0.17 [-0.03 , 0.36]

0.13 [-0.00 , 0.26]

Risk Difference
IV, Random, 95% CI


-1

-0.5

0

0.5

1


Favours paper PIL

Favours MMI PIL


Risk of Bias
A

+

+
B

+

+
C

+

+
D

+

+
E

+

+
F

+

+
G

+

+
H

+

+

Footnotes

a
CI calculated by Wald-type method.

b
Tau² calculated by Restricted Maximum-Likelihood method.
Risk of bias legend

(A) Random sequence generation ok?

(B) Allocation concealment?

(C) Blinding of participants and personnel ok?

(D) Blinding of outcome assessment ok?

(E) Incomplete outcome data handled ok?

(F) Free of selective reporting?

(G) Was the study free of other bias?

(H) Overall bias?


Analysis 54.1: Primary care centre recruited


Study or Subgroup
Lee 2017

Total
Total events:
Test for overall effect: Z = 0.02 (P = 0.98)

Heterogeneity: Not applicable

Teaser campaign
Events
32
32
Total
329

329

No teaser
Events
33
33
Total
341

341
Weight
100.0%

100.0%

Risk Difference
M-H, Fixed, 95% CI
0.00 [-0.04 , 0.05]

0.00 [-0.04 , 0.05]

Risk Difference
M-H, Fixed, 95% CI


-1

-0.5

0

0.5

1


Favours no teaser

Favours teaser


Risk of Bias
A

+
B

+
C

+
D

+
E

+
F

+
G

+
H

+

Risk of bias legend

(A) Random sequence generation ok?

(B) Allocation concealment?

(C) Blinding of participants and personnel ok?

(D) Blinding of outcome assessment ok?

(E) Incomplete outcome data handled ok?

(F) Free of selective reporting?

(G) Was the study free of other bias?

(H) Overall bias?


Analysis 55.1: Participants recruited


Study or Subgroup
Tilley 2021

Total
Total events:
Test for overall effect: Z = 0.13 (P = 0.90)

Heterogeneity: Not applicable

Educational programme
Events
225
225
Total
1231

1231

Usual recruitment
Events
162
162
Total
876

876
Weight
100.0%

100.0%

Risk Difference
M-H, Fixed, 95% CI
-0.00 [-0.04 , 0.03]

-0.00 [-0.04 , 0.03]

Risk Difference
M-H, Fixed, 95% CI


-1

-0.5

0

0.5

1


Favours usual recruitment

Favours educational program


Risk of Bias
A

+
B

+
C

?
D

+
E

+
F

+
G

+
H

?

Risk of bias legend

(A) Random sequence generation ok?

(B) Allocation concealment?

(C) Blinding of participants and personnel ok?

(D) Blinding of outcome assessment ok?

(E) Incomplete outcome data handled ok?

(F) Free of selective reporting?

(G) Was the study free of other bias?

(H) Overall bias?


Analysis 56.1: Participants recruited


Study or Subgroup
Jefferson 2018

Total
Total events:
Test for overall effect: Z = 0.65 (P = 0.52)

Heterogeneity: Not applicable

Remote SIV
Events
185
185
Total
329

329

Face-to-face SIV
Events
193
193
Total
359

359
Weight
100.0%

100.0%

Risk Difference
M-H, Fixed, 95% CI
0.02 [-0.05 , 0.10]

0.02 [-0.05 , 0.10]

Risk Difference
M-H, Fixed, 95% CI


-1

-0.5

0

0.5

1


Favours face-to-face

Favours remote


Risk of Bias
A

+
B

+
C

+
D

+
E

+
F

+
G

+
H

+

Risk of bias legend

(A) Random sequence generation ok?

(B) Allocation concealment?

(C) Blinding of participants and personnel ok?

(D) Blinding of outcome assessment ok?

(E) Incomplete outcome data handled ok?

(F) Free of selective reporting?

(G) Was the study free of other bias?

(H) Overall bias?


Analysis 56.2: Participants retained


Study or Subgroup
Jefferson 2018

Total
Total events:
Test for overall effect: Z = 0.35 (P = 0.73)

Heterogeneity: Not applicable

Remote SIV
Events
151
151
Total
329

329

Face-to-face SIV
Events
160
160
Total
359

359
Weight
100.0%

100.0%

Risk Difference
M-H, Fixed, 95% CI
0.01 [-0.06 , 0.09]

0.01 [-0.06 , 0.09]

Risk Difference
M-H, Fixed, 95% CI


-1

-0.5

0

0.5

1


Favours face-to-face

Favours remote 


Risk of Bias
A

+
B

+
C

+
D

+
E

+
F

+
G

+
H

+

Risk of bias legend

(A) Random sequence generation ok?

(B) Allocation concealment?

(C) Blinding of participants and personnel ok?

(D) Blinding of outcome assessment ok?

(E) Incomplete outcome data handled ok?

(F) Free of selective reporting?

(G) Was the study free of other bias?

(H) Overall bias?


Analysis 63.1: Participants recruited


Study or Subgroup

63.1.1 Up to £5
Fairhurst 2022
Free 2010

Subtotal (Wald
a
)
Total events:
Test for overall effect: Z = 1.18 (P = 0.24)

Heterogeneity: Tau² (REML
b
) = 0.00; Chi² = 5.29, df = 1 (P = 0.02); I² = 81%

63.1.2 Around £100
Halpern 2021b ($100)
Jennings 2015a
Jennings 2015b
Jennings 2015c
Jennings 2015d
Jennings 2015e

Subtotal (Wald
a
)
Total events:
Test for overall effect: Z = 1.32 (P = 0.19)

Heterogeneity: Tau² (REML
b
) = 0.00; Chi² = 10.94, df = 5 (P = 0.05); I² = 55%

63.1.3 Over £100
Giguere 2015
Halpern 2021a ($200)
Halpern 2021a ($500)
Halpern 2021b ($300)

Subtotal (Wald
a
)
Total events:
Test for overall effect: Z = 1.66 (P = 0.10)

Heterogeneity: Tau² (REML
b
) = 0.01; Chi² = 23.12, df = 3 (P < 0.0001); I² = 89%

Total (Wald
a
)
Total events:
Test for overall effect: Z = 2.43 (P = 0.02)
Test for subgroup differences: Chi² = 1.30, df = 2 (P = 0.52), I² = 0%

Heterogeneity: Tau² (REML
b
) = 0.00; Chi² = 40.45, df = 11 (P < 0.0001); I² = 89%

Payment
Events
14
13
27
102
26
58
2
3
5
196
25
78
104
92
299
522
Total
416
246

662
212
84
158
46
101
92

693
724
217
221
214

1376

2731

No payment
Events
12
1
13
49
24
40
3
6
0
122
4
23
24
49
100
235
Total
416
245

661
108
97
174
47
109
107

642
724
108
108
108

1048

2351
Weight
11.3%
11.1%

22.4%
6.1%
5.4%
7.1%
7.5%
9.8%
10.1%

46.0%
11.6%
7.0%
6.8%
6.2%

31.6%

100.0%

Risk Difference
IV, Random, 95% CI
0.00 [-0.02 , 0.03]
0.05 [0.02 , 0.08]

0.03 [-0.02 , 0.07]
0.03 [-0.09 , 0.14]
0.06 [-0.07 , 0.19]
0.14 [0.04 , 0.23]
-0.02 [-0.11 , 0.07]
-0.03 [-0.08 , 0.03]
0.05 [0.00 , 0.10]

0.03 [-0.02 , 0.08]
0.03 [0.01 , 0.04]
0.15 [0.05 , 0.25]
0.25 [0.15 , 0.35]
-0.02 [-0.14 , 0.09]

0.10 [-0.02 , 0.21]

0.05 [0.01 , 0.09]

Risk Difference
IV, Random, 95% CI


-0.5

-0.25

0

0.25

0.5


Favours no payment

Favours payment


Risk of Bias
A

+

+

?

+

+

+

+

+

+

?

?

?
B

+

+

?

+

+

+

+

+

+

?

?

?
C

−

+

−

+

+

+

+

+

+

−

−

−
D

+

+

+

+

+

+

+

+

+

+

+

+
E

+

+

+

+

+

+

+

+

+

+

+

+
F

+

+

+

+

+

+

+

+

+

+

+

+
G

+

+

+

+

+

+

+

+

+

+

+

+
H

−

+

−

+

+

+

+

+

+

−

−

−

Footnotes

a
CI calculated by Wald-type method.

b
Tau² calculated by Restricted Maximum-Likelihood method.
Risk of bias legend

(A) Random sequence generation ok?

(B) Allocation concealment?

(C) Blinding of participants and personnel ok?

(D) Blinding of outcome assessment ok?

(E) Incomplete outcome data handled ok?

(F) Free of selective reporting?

(G) Was the study free of other bias?

(H) Overall bias?


Analysis 63.2: Participants recruited (by allocation concealment)


Study or Subgroup

63.2.1 Adequate allocation concealment
Fairhurst 2022
Free 2010
Giguere 2015
Jennings 2015a
Jennings 2015b
Jennings 2015c
Jennings 2015d
Jennings 2015e

Subtotal (Wald
a
)
Total events:
Test for overall effect: Z = 2.37 (P = 0.02)

Heterogeneity: Tau² (REML
b
) = 0.00; Chi² = 16.56, df = 7 (P = 0.02); I² = 62%

63.2.2 Inadequate or unclear allocation concealment
Halpern 2021a ($200)
Halpern 2021a ($500)
Halpern 2021b ($100)
Halpern 2021b ($300)

Subtotal (Wald
a
)
Total events:
Test for overall effect: Z = 1.67 (P = 0.09)

Heterogeneity: Tau² (REML
b
) = 0.01; Chi² = 14.69, df = 3 (P = 0.002); I² = 80%

Total (Wald
a
)
Total events:
Test for overall effect: Z = 2.43 (P = 0.02)
Test for subgroup differences: Chi² = 1.41, df = 1 (P = 0.24), I² = 29.0%

Heterogeneity: Tau² (REML
b
) = 0.00; Chi² = 40.45, df = 11 (P < 0.0001); I² = 89%

Payment
Events
14
13
25
26
58
2
3
5
146
78
104
102
92
376
522
Total
416
246
724
84
158
46
101
92

1867
217
221
212
214

864

2731

No payment
Events
12
1
4
24
40
3
6
0
90
23
24
49
49
145
235
Total
416
245
724
97
174
47
109
107

1919
108
108
108
108

432

2351
Weight
11.3%
11.1%
11.6%
5.4%
7.1%
7.5%
9.8%
10.1%

73.9%
7.0%
6.8%
6.1%
6.2%

26.1%

100.0%

Risk Difference
IV, Random, 95% CI
0.00 [-0.02 , 0.03]
0.05 [0.02 , 0.08]
0.03 [0.01 , 0.04]
0.06 [-0.07 , 0.19]
0.14 [0.04 , 0.23]
-0.02 [-0.11 , 0.07]
-0.03 [-0.08 , 0.03]
0.05 [0.00 , 0.10]

0.03 [0.00 , 0.05]
0.15 [0.05 , 0.25]
0.25 [0.15 , 0.35]
0.03 [-0.09 , 0.14]
-0.02 [-0.14 , 0.09]

0.10 [-0.02 , 0.22]

0.05 [0.01 , 0.09]

Risk Difference
IV, Random, 95% CI


-1

-0.5

0

0.5

1


Favours no payment

Favours payment


Risk of Bias
A

+

+

+

+

+

+

+

+

?

?

?

?
B

+

+

+

+

+

+

+

+

?

?

?

?
C

−

+

+

+

+

+

+

+

−

−

−

−
D

+

+

+

+

+

+

+

+

+

+

+

+
E

+

+

+

+

+

+

+

+

+

+

+

+
F

+

+

+

+

+

+

+

+

+

+

+

+
G

+

+

+

+

+

+

+

+

+

+

+

+
H

−

+

+

+

+

+

+

+

−

−

−

−

Footnotes

a
CI calculated by Wald-type method.

b
Tau² calculated by Restricted Maximum-Likelihood method.
Risk of bias legend

(A) Random sequence generation ok?

(B) Allocation concealment?

(C) Blinding of participants and personnel ok?

(D) Blinding of outcome assessment ok?

(E) Incomplete outcome data handled ok?

(F) Free of selective reporting?

(G) Was the study free of other bias?

(H) Overall bias?


Analysis 64.1: Participants recruited


Study or Subgroup
Halpern 2021a ($200)
Halpern 2021b ($100)

Total (Wald
a
)
Total events:
Test for overall effect: Z = 0.37 (P = 0.71)

Heterogeneity: Tau² (REML
b
) = 0.01; Chi² = 5.84, df = 1 (P = 0.02); I² = 83%

Higher value
Events
104
92
196
Total
221
214

435

Lower value
Events
78
102
180
Total
217
212

429
Weight
50.3%
49.7%

100.0%

Risk Difference
IV, Random, 95% CI
0.11 [0.02 , 0.20]
-0.05 [-0.15 , 0.04]

0.03 [-0.13 , 0.19]

Risk Difference
IV, Random, 95% CI


-0.5

-0.25

0

0.25

0.5


Favours lower value

Favours higher value


Risk of Bias
A

?

?
B

?

?
C

−

−
D

+

+
E

+

+
F

+

+
G

+

+
H

−

−

Footnotes

a
CI calculated by Wald-type method.

b
Tau² calculated by Restricted Maximum-Likelihood method.
Risk of bias legend

(A) Random sequence generation ok?

(B) Allocation concealment?

(C) Blinding of participants and personnel ok?

(D) Blinding of outcome assessment ok?

(E) Incomplete outcome data handled ok?

(F) Free of selective reporting?

(G) Was the study free of other bias?

(H) Overall bias?


Analysis 65.1: Participants recruited


Study or Subgroup
Fairhurst 2022
White 2023
Whiteside 2019

Total (Wald
a
)
Total events:
Test for overall effect: Z = 0.56 (P = 0.57)

Heterogeneity: Tau² (REML
b
) = 0.00; Chi² = 2.17, df = 2 (P = 0.34); I² = 19%

Pen
Events
15
145
28
188
Total
416
3464
620

4500

No pen
Events
11
173
54
238
Total
416
3482
1242

5140
Weight
16.2%
61.8%
21.9%

100.0%

Risk Difference
IV, Random, 95% CI
0.01 [-0.01 , 0.03]
-0.01 [-0.02 , 0.00]
0.00 [-0.02 , 0.02]

-0.00 [-0.01 , 0.01]

Risk Difference
IV, Random, 95% CI


-0.5

-0.25

0

0.25

0.5


Favours no pen

Favours pen


Risk of Bias
A

+

+

+
B

+

+

+
C

−

+

+
D

+

+

+
E

+

?

+
F

+

+

+
G

+

+

+
H

−

?

+

Footnotes

a
CI calculated by Wald-type method.

b
Tau² calculated by Restricted Maximum-Likelihood method.
Risk of bias legend

(A) Random sequence generation ok?

(B) Allocation concealment?

(C) Blinding of participants and personnel ok?

(D) Blinding of outcome assessment ok?

(E) Incomplete outcome data handled ok?

(F) Free of selective reporting?

(G) Was the study free of other bias?

(H) Overall bias?


Analysis 65.2: Participants retained


Study or Subgroup
Whiteside 2019

Total
Total events:
Test for overall effect: Z = 0.41 (P = 0.68)

Heterogeneity: Not applicable

Pen
Events
27
27
Total
620

620

No pen
Events
49
49
Total
1242

1242
Weight
100.0%

100.0%

Risk Difference
M-H, Fixed, 95% CI
0.00 [-0.02 , 0.02]

0.00 [-0.02 , 0.02]

Risk Difference
M-H, Fixed, 95% CI


-0.5

-0.25

0

0.25

0.5


Favours no pen

Favours pen


Risk of Bias
A

+
B

+
C

+
D

+
E

+
F

+
G

+
H

+

Risk of bias legend

(A) Random sequence generation ok?

(B) Allocation concealment?

(C) Blinding of participants and personnel ok?

(D) Blinding of outcome assessment ok?

(E) Incomplete outcome data handled ok?

(F) Free of selective reporting?

(G) Was the study free of other bias?

(H) Overall bias?
