# Supplementary material for: Strategies to improve recruitment to randomised trials
Source: Cochrane Database Syst Rev. 2026 Sep 15;2026(9):MR000013. doi: 10.1002/14651858.MR000013.pub7 (PMC13576036; doi:10.1002/14651858.MR000013.pub7)
Supplement: Supplementary file 5 [file MR000013-SUP-05-dataPackage.zip › MR000013-data-package-info.html]

Data package 

# Supplementary material 5 to: Strategies to improve recruitment to randomised trials

Parker A, Mongelli G, Piccolo-Lawrance C, Coleman E, Gkekas A, Wang HI, Dawson S, Green H, Way R, Rai A, Bruhn H, Sullivan F, Boxall C, Chan M, Manson P, Hewitt C, Fairhurst C, Clarke M, Treweek S  
https://doi.org/10.1002/14651858.MR000013.pub7

The material in this section has been supplied by the author(s) for publication under a Licence for Publication and the author(s) are solely responsible for the material. Cochrane has reviewed this material, but Cochrane has not copyedited, formatted or proofread. Cochrane accordingly gives no representations or warranties of any kind in relation to, and accepts no liability for any reliance on or use of, such material.

Back to top

# Data package

This data package conforms to version 2.5 of the Cochrane review data package specification.

Depending on the nature of the review, this data package may contain one or more of the following elements:

- Study data:
  - Study information (information on which studies exist and their study characteristics, separate files for included, excluded, awaiting and ongoing studies)
  - Study arms (arms reported for the included studies in the review)
  - Study references (separate files for included, excluded, awaiting and ongoing studies)
  - Study results data (outcome data reported for the included studies in the review)
  - Risk of bias (judgements and support for judgements for the included studies in the review)
  - Study data JSON (includes all of the above; separate files for included, excluded, awaiting and ongoing studies)
- Analysis data:
  - Overall estimates and settings (analysis level)
  - Subgroup estimates (mid-level estimates for each subgroup)
  - Individual data rows (all the rows in all the analyses)
  - Analysis data JSON (includes all of the above)
- Summary of findings JSON (only available for dynamic tables created in RevMan)
- Other references:
  - Additional references
  - References to other versions of the review

For more information on how to interpret and use the contents of this data package, please refer to the user guide.
