# Supplementary material for: Strategies to improve recruitment to randomised trials
Source: Cochrane Database Syst Rev. 2026 Sep 15;2026(9):MR000013. doi: 10.1002/14651858.MR000013.pub7 (PMC13576036; doi:10.1002/14651858.MR000013.pub7)
Supplement: Supplementary file 6 [file MR000013-SUP-06-other.html]

Protocol


# Supplementary material 6 to: Strategies to improve recruitment to randomised trials

Parker A, Mongelli G, Piccolo-Lawrance C, Coleman E, Gkekas A, Wang HI, Dawson S, Green H, Way R, Rai A, Bruhn H, Sullivan F, Boxall C, Chan M, Manson P, Hewitt C, Fairhurst C, Clarke M, Treweek S
  
https://doi.org/10.1002/14651858.MR000013.pub7

The material in this section has been supplied by the author(s) for publication under a Licence for Publication and the author(s) are solely responsible for the material. Cochrane has reviewed this material, but Cochrane has not copyedited, formatted or proofread. Cochrane accordingly gives no representations or warranties of any kind in relation to, and accepts no liability for any reliance on or use of, such material.

Back to top

# Protocol

## Cover sheet

## Title

Strategies to improve recruitment to randomised trials

### Reviewers

Parker A, Mongelli G, Piccolo-Lawrance C, Coleman I, Gkekas N, Wang HI, Dawson S, Green H, Way R, Rai A, Bruhn H, Sullivan F, Boxall C, Chan M, Manson P, Hewitt C, Fairhurst C, Clarke M, Treweek S

### Contact reviewer

Adwoa Parker

York Trials Unit

Department of Health Sciences

University of York

Heslington

YO10 5DD

UK

Telephone: +44 1904 321671

E-mail: adwoa.parker@york.ac.uk

Secondary contact person's name:

Prof Shaun Treweek  
Health Services Research Unit  
University of Aberdeen  
3rd Floor, Health Sciences Building  
Foresterhill  
Aberdeen  
AB25 2ZD, UK  
  
Telephone: +44 1224 438145  
Mobile:+44 777 901 6955  
E‐mail: streweek@mac.com

### Contribution of reviewers

All authors contributed to the writing of the protocol.

### Internal sources of support

None

### External sources of support

This project is funded by the UK National Institute for Health and Care Research (NIHR) Advanced Fellowship (reference: NIHR302256). The views expressed are those of the authors and not necessarily those of the NIHR or the Department of Health and Social Care.

### Background

Essentially all trials need to recruit participants but this is often a challenge. Poor recruitment can lead to an underpowered study, which may report clinically relevant effects to be statistically non‐significant. A non‐significant finding increases the risk that an effective intervention will be abandoned before its true value is established, or that there will be a delay in demonstrating this value while more trials or meta‐analyses are done. Underpowered trials also raise an ethical problem: trialists have exposed participants to an intervention with uncertain benefit but may still be unable to determine whether the intervention does more good than harm on completion of the trial. Poor recruitment can also lead to the trial being extended, increasing costs.

Although investigations of recruitment differ in their estimates of the proportion of studies that achieve their recruitment targets, it is likely that less than 50% meet their target (Charlson 1984; Foy 2003; Haidich 2001; McDonald 2006; Sully 2013). For example, McDonald and colleagues found that only 38 (31%) of 114 trials achieved their original recruitment target and 65 (53%) were extended (McDonald 2006). A more recent replication of this work found that the number of trials meeting recruitment targets had increased to around 63% (245/388), but 32% (79/245) of these trials required an extension to their recruitment period to meet the target, meaning approximately 43% of trials recruit to target without an extension (Jacques 2022). An additional 22% (86/388) of trials recruited to within 80% of their final recruitment target with 36% (31/86) of these trials requiring an extension to their recruitment period (Jacques 2022). The costs of poor recruitment can be huge (Kitterman 2011).

Trialists use many interventions to improve recruitment (see for example Caldwell 2010, Watson 2006 and Prescott 1999) but it is generally difficult to predict the effect of these interventions.

This review updates the Treweek 2018, Treweek 2013, and Treweek 2010 reviews.

### Objectives

The primary objective is to quantify the effects of strategies to improve recruitment of participants to randomised controlled trials.

Secondary objectives are to:

1. Assess the cost-effectiveness of recruitment strategies, where there is available evidence. This objective is new for this review update.
2. Assess the impact of the recruitment strategy on retention rates. This objective is new for this review update.
3. Determine the effectiveness of recruitment strategies across different patient populations, including for sex, age, ethnicity, geographic location, and deprivation. This objective is new for this review update.

## Criteria for considering studies for this review

### Types of studies

Randomised trials of interventions to improve recruitment to randomised trials.

We will exclude quasi-randomised trials testing recruitment strategies, as well as strategies tested in quasi-randomised host trials, as these types of studies are at high risk of bias and have low GRADE certainty for evidence.

### Types of participants

Randomised trials of recruitment strategies set in the context of trials but not limited to health care; interventions that work in other fields (e.g., education, housing) could be applicable to healthcare settings. Only strategies within real settings are eligible for this version of the review.

**Note:** We have excluded hypothetical trials since these are all considered to be at high risk of bias because the recruitment decision is not a real one; many also have other methodological problems. There are three reasons for deciding to exclude them:

1. The relevance of the results of hypothetical trials will always be in doubt because of uncertainty as to how people would have reacted had the decision to take part in a trial been a real one not a hypothetical one.
2. It clearly is possible to study recruitment interventions in real trials, avoiding the above problem.
3. Now that the number of evaluations in real trials has increased, we do not think the trade‐off between value‐added and work involved to include hypothetical trials comes down in favour of including hypothetical trials in future versions of this review.

We will exclude research into ways to improve questionnaire response and research looking at incentives and disincentives for clinicians to recruit patients to trials as these issues are addressed by complementary Cochrane Methodology Reviews (Edwards 2009; Rendell 2007). Studies of retention strategies will also be excluded as a Cochrane Methodology Review on strategies to reduce attrition from trials already exists (Gillies 2022; Brueton 2013).

### Types of interventions

Any intervention that aimed to improve recruitment of participants to a randomised trial. The interventions being studied could be directed at potential participants (e.g., patients being randomised to a trial), collaborators (e.g., clinicians recruiting patients for a trial), or others (e.g., research ethics committees). Examples of such interventions are letters introducing the trial being signed by influential people, alternative methods of providing information about the trial to potential participants, additional training for collaborators, monetary incentives for participants, telephone follow‐up of expressions of interest and modifications to the design of the trial (e.g., using a preference design).

### Types of outcome measures

#### *Primary*

Proportion of eligible individuals or centres recruited.

#### *Secondary*

- Cost-effectiveness: Incremental cost-effectiveness ratio (ICER), expressed as the incremental cost per additional participant recruited. This outcome is new for this review.
- Impact of the recruitment strategy on retention rates (with incremental cost per additional participant retained if available). This outcome is new for this review.

Additionally, we will explore the effectiveness of the strategies for recruiting diverse patient populations, such as sex, age, ethnicity, geographic location, and deprivation.

### Search strategy for identification of studies

We will search the following electronic databases without language restriction for eligible studies:

- The Cochrane Methodology Review Group Specialised Register (CMR)
- MEDLINE and MEDLINE In Process (OVID)
- EMBASE (OVID)
- Science Citation Index & Social Science Citation Index (ISI)
- ERIC (EBSCO)
- CINHAL Plus (EBSCO)
- ASSIA
- PyscINFO (EBSCO)

The search results will be downloaded to Endnote reference management software, de‐duplicated and uploaded to covidence (covidence 2023) to enable screening and data extraction.

The following MEDLINE search strategy will be adjusted according to the above listed databases.

Search Strategy:

‐‐‐‐‐‐‐‐‐‐‐‐‐‐‐‐‐‐‐‐‐‐‐‐‐‐‐‐‐‐‐‐‐‐‐‐‐‐‐‐‐‐‐‐‐‐‐‐‐‐‐‐‐‐‐‐‐‐‐‐‐‐‐‐‐‐‐‐‐‐‐‐‐‐‐‐‐‐‐‐

1 Patient Selection/ 69271

2 ((participat$ or recruit$ or enrol$) adj4 trial?).tw. 35516

3 1 or 2 102118

4 Informed Consent/ 38582

5 informed consent.tw. 43089

6 4 or 5 70143

7 exp Clinical Trials as Topic/ 380558

8 Research Subjects/ 6673

9 (trial? or study or studies or research).tw. 13061511

10 7 or 8 or 9 13167732

11 3 or (6 and 10) 139927

12 (research support nih extramural or research support nih intramural or research support non us govt or research support us govt non phs or research support us govt phs).pt. 10133718

13 recruitment.ab. /freq=2 32756

14 participation.ab. /freq=2 38366

15 12 or 13 or 14 10167972

16 11 and 15 45241

17 randomized controlled trial.pt. 587215

18 controlled clinical trial.pt. 95195

19 random$.ab. 1351586

20 ((nest\* or embed\*) adj4 (RCT or trial? or stud\*)).tw. 20524

21 (substud\* or sub-stud\*).tw. 8692

22 ("stud\* within a trial" or "trial? within a trial").tw. 91

23 ("stud\* in a trial" or "trial? in a trial").tw. 41

24 ("stud\* within a study" or "trial? within a study").tw. 3

25 ("stud\* in a study" or "trial? in a study").tw. 51

26 20 or 21 or 22 or 23 or 24 or 25 29183

27 16 and (17 or 18 or 19 or 26) 17540

28 exp animals/ not humans/ 5095551

29 27 not 28 17508

30 29 not (comment or editorial).pt. 17468

31 limit 30 to yr="2015 -Current" 7559

\*\*\*\*\*\*\*\*\*\*\*\*\*\*\*\*\*\*\*\*\*\*\*\*\*\*\*

## Methods of the review

### Identifying trials

Two authors will independently screen the titles and abstracts of all records retrieved from the searches of the electronic bibliographic databases. If necessary, any disagreements will be resolved through the involvement of a third author. The full text will be obtained for studies that appear to meet the inclusion criteria. All potentially eligible studies will be independently assessed by two authors to determine if they meet the inclusion criteria. Any disagreements will be resolved through discussion or the involvement of a third author.

### Assessment of methodological quality

We will use the Cochrane Risk of Bias tool (Cochrane Risk of Bias tool) to assess risk of bias. We will use GRADE (Guyatt 2008) on all studies where relevant data are available. Where we do a meta‐analysis, the details of the GRADE assessment will be given in the relevant Summary of Findings table. Where we use GRADE on a single study, we will use the following rules for assigning a GRADE rating of High, Moderate, Low or Very low:

- All studies start at High
- Study limitations: downgrade all high RoB studies by two levels; downgrade all uncertain RoB studies by one level.
- Inconsistency: assume no serious inconsistency.
- Indirectness: downgrade all hypothetical studies by two levels.
- Imprecision: downgrade all single studies by one level because of the sparseness of data; downgrade by a further one level if the confidence interval is wide and crosses the line where risk difference = 0.
- Reporting bias: assume no serious reporting bias.

Data on methodological quality will be presented in an additional table for all included studies.

Although we will not exclude studies because of a high of risk of bias, the low confidence we have in the data they present means that these studies will not be mentioned in the text of the Results or Discussion, except where it has been possible to include them in a meta‐analysis and the data can be interpreted together with data from other studies.

High risk of bias studies will appear in Data and analyses but we suggest that readers use these data only to make decisions as to whether they would like to evaluate the intervention themselves in a more rigorous way. We do not believe they should be used to make judgements about effect.

### Data extraction

Two review authors will independently carry out data extraction of each included article (using a proforma specifically designed for the purpose). Differences in data extraction will be resolved by discussion. We will extract data on the method evaluated; country in which the study was carried out; nature of the population; nature of the study setting; nature of the study to be recruited into; randomisation method; numbers and proportions of participants in the intervention and comparator groups of the study comparing recruitment strategies; data related to the inclusion of diverse participants such as sex, age, ethnicity, geographic location, and deprivation; and data on any costs presented.

### Data analysis

Trials will be grouped according to the type of intervention based on the categorisation used in the Online Resource for Recruitment research in Clinical triAls (ORRCA) project. We split one ORRCA category (Recruitment Information Needs) into two so as to separate out interventions aimed at the consent process from those aimed at more general participant information. Our seven categories are therefore:

1. **Design (Category A).** This includes changes to the general design of the trial specifically done to increase recruitment.
2. **Pre‐trial planning (Category B).** This includes work done before the trial starts (possibly in a separate study) to explicitly make it more likely that recruitment will be successful.
3. **Trial conduct changes (Category C).** This includes initiatives implemented once the trial has started such as better ways of identifying participants, changes to how data are collected, changes to the type of data collected, tailor recruitment to different types of participant.
4. **Modifications to the consent process (Category D).** This includes changes to the staff member helping with consent, when consent is taken, what sort of consent information is presented and how it is presented.
5. **Modification to the information given to potential participants about the trial (Category E).** This includes who provides it, when, where what sort of information is presented, how the information is presented.
6. **Interventions aimed at the recruiter or recruitment site (Category F).** This includes anything that is aimed at the recruiter or recruitment site staff rather than the person being recruited such as changes to training.
7. **Incentives (Category G).** Monetary and other incentives for participants (but not staff, which is covered by a separate review).

We will present results as risk difference (RD) with the associated 95% confidence intervals (CIs) where sufficient data are available. We will only include cluster‐randomised trials in the meta‐analysis if sufficient data were reported to allow inclusion of analyses that adjusted for clustering; an odds ratio (OR) will be used as the summary effect in the meta‐analysis result if risk difference or risk ratio clustering adjusted analyses were not possible with available data. Where two or more studies could be included in meta‐analyses we will use a random effect approach using a restricted maximum likelihood (REML) method to produce a pooled estimate, due to the likely presence of substantial heterogeneity. The CI for the summary effect will be calculated using the Wald-type method. However, when the between-study estimate is greater than zero and there are more than two studies in the comparison, then the Hartung and Knapp and by Sidik and Jonkman (HKSJ) method will be used.

Cost-effectiveness outcomes will be reported in terms of the incremental cost-effectiveness ratio (ICER), i.e., the incremental cost per additional patient randomised or per additional patient consented. Where it is not possible to report cost-effectiveness, we will report any costs of recruitment strategies available.

Publication bias will be investigated for the primary outcomes using a funnel plot where 10 or more studies are available.

## Potential conflict of interest

None known.

## Additional references

None. All are listed in main review reference list.

### Contributions to the protocol

Updated December 2022 by Adwoa Parker, Gloria Mongelli, Elizabeth Coleman, Shoba Dawson, Heidi Gardner, Arti Rai, Athanasios Gkekas, Hanne Bruhne, Frank Sullivan, Paul Manson, Catherine Jackson, Cherish Boxall, Shaun Treweek

Contributing authors (May 2017): Treweek S, Pitkethly M, Cook J, Mitchell E, Sullivan F,Fraser C, Jackson C, Johansen M, Taskila T, Wilson S, Jones R, Lockhart P, Gardner H.

## Glossary of selected terms

See the GET IT Glossary (http://getitglossary.org) for plain language definitions of a wide range of terms relevant to fair tests of treatments.
