# Supplementary material for: Strategies to improve recruitment to randomised trials
Source: Cochrane Database Syst Rev. 2026 Sep 15;2026(9):MR000013. doi: 10.1002/14651858.MR000013.pub7 (PMC13576036; doi:10.1002/14651858.MR000013.pub7)
Supplement: Supplementary file 7 [file MR000013-SUP-07-other.html]

Reference list of reviews and other publications searched


# Supplementary material 7 to: Strategies to improve recruitment to randomised trials

Parker A, Mongelli G, Piccolo-Lawrance C, Coleman E, Gkekas A, Wang HI, Dawson S, Green H, Way R, Rai A, Bruhn H, Sullivan F, Boxall C, Chan M, Manson P, Hewitt C, Fairhurst C, Clarke M, Treweek S
  
https://doi.org/10.1002/14651858.MR000013.pub7

The material in this section has been supplied by the author(s) for publication under a Licence for Publication and the author(s) are solely responsible for the material. Cochrane has reviewed this material, but Cochrane has not copyedited, formatted or proofread. Cochrane accordingly gives no representations or warranties of any kind in relation to, and accepts no liability for any reliance on or use of, such material.

Back to top

# Reference list of reviews and other publications searched

Abdelazeem B, Abbas KS, Amin MA, El-Shahat NA, Malik B, Kalantary A, Eltobgy M. The effectiveness of incentives for research participation: A systematic review and meta-analysis of randomized controlled trials. PLoS One. 2022 Apr 22;17(4):e0267534. doi: 10.1371/journal.pone.0267534. PMID: 35452488; PMCID: PMC9032371.

Boland J, Currow DC, Wilcock A, Tieman J, Hussain JA, Pitsillides C, Abernethy AP, Johnson MJ. A systematic review of strategies used to increase recruitment of people with cancer or organ failure into clinical trials: implications for palliative care research. J Pain Symptom Manage. 2015 Apr;49(4):762-772.e5. doi: 10.1016/j.jpainsymman.2014.09.018. Epub 2014 Dec 27. PMID: 25546286.

Crocker JC, Ricci-Cabello I, Parker A, Hirst JA, Chant A, Petit-Zeman S, Evans D, Rees S. Impact of patient and public involvement on enrolment and retention in clinical trials: systematic review and meta-analysis. BMJ. 2018 Nov 28;363:k4738. doi: 10.1136/bmj.k4738. PMID: 30487232; PMCID: PMC6259046.

Davis R, Bekker P. Recruitment of Older Adults With Dementia for Research: An Integrative Review. Res Gerontol Nurs. 2022 Sep-Oct;15(5):255-264. doi: 10.3928/19404921-20220830-04. Epub 2022 Sep 1. PMID: 36113010; PMCID: PMC10950034.

Delaney H, Devane D, Hunter A, Hennessy M, Parker A, Murphy L, Cronin P, Smith V. Limited evidence exists on the effectiveness of education and training interventions on trial recruitment; a systematic review. J Clin Epidemiol. 2019 Sep;113:75-82. doi: 10.1016/j.jclinepi.2019.05.013. Epub 2019 May 22. PMID: 31128220.

Preston NJ, Farquhar MC, Walshe CE, Stevinson C, Ewing G, Calman LA, Burden S, Brown Wilson C, Hopkinson JB, Todd C. Strategies designed to help healthcare professionals to recruit participants to research studies. Cochrane Database Syst Rev. 2016 Feb 29;2(2):MR000036. doi: 10.1002/14651858.MR000036.pub2. PMID: 35658160; PMCID: PMC8190980.

Feldman WB, Kim AS, Josephson SA, Lowenstein DH, Chiong W. Effect of waivers of consent on recruitment in acute stroke trials: A systematic review. Neurology. 2016 Apr 19;86(16):1543-51. doi: 10.1212/WNL.0000000000002587. Epub 2016 Mar 23. PMID: 27009262; PMCID: PMC4836887.

Frampton, G.K., Shepherd, J., Pickett, K. et al. Digital tools for the recruitment and retention of participants in randomised controlled trials: a systematic map. Trials 21, 478 (2020). https://doi.org/10.1186/s13063-020-04358-3

Gillies K, Cotton SC, Brehaut JC, Politi MC, Skea Z. Decision aids for people considering taking part in clinical trials. Cochrane Database Syst Rev. 2015 Nov 27;2015(11):CD009736. doi: 10.1002/14651858.CD009736.pub2. PMID: 26613337; PMCID: PMC8725643.

Hughson JA, Woodward-Kron R, Parker A, Hajek J, Bresin A, Knoch U, Phan T, Story D. A review of approaches to improve participation of culturally and linguistically diverse populations in clinical trials. Trials. 2016 May 26;17(1):263. doi: 10.1186/s13063-016-1384-3. PMID: 27229153; PMCID: PMC4880985.

Lacey RJ, Wilkie R, Wynne-Jones G, Jordan JL, Wersocki E, McBeth J. Evidence for strategies that improve recruitment and retention of adults aged 65 years and over in randomised trials and observational studies: a systematic review. Age Ageing. 2017 Nov 1;46(6):895-903. doi: 10.1093/ageing/afx057. PMID: 28481964.

Madurasinghe, V.W., Bower, P., Eldridge, S. et al. Can we achieve better recruitment by providing better information? Meta-analysis of ‘studies within a trial’ (SWATs) of optimised participant information sheets. BMC Med 19, 218 (2021). https://doi.org/10.1186/s12916-021-02086-2

Masood Y, Bower P, Waheed MW, Brown G, Waheed W. Synthesis of researcher reported strategies to recruit adults of ethnic minorities to clinical trials in the United Kingdom: A systematic review. Contemp Clin Trials. 2019 Mar;78:1-10. doi: 10.1016/j.cct.2019.01.004. Epub 2019 Jan 8. PMID: 30634035.

McGill, K., Sackley, C.M., Godwin, J. et al. A systematic review of the efficiency of recruitment to stroke rehabilitation randomised controlled trials. Trials 21, 68 (2020). https://doi.org/10.1186/s13063-019-3991-2

McNamara R, Davy K, Niranjan V, O'Regan A. Recruitment and characteristics of participants in trials of physical activity for adults aged 45 years and above in general practice: a systematic review. Fam Pract. 2023 Mar 28;40(2):387-397. doi: 10.1093/fampra/cmac128. PMID: 36472583; PMCID: PMC10047612.

Niznick N, Lun R, Dewar B, et al. Advance consent for participation in randomised controlled trials for emergency conditions: a scoping reviewBMJ Open 2023;13:e066742. doi: 10.1136/bmjopen-2022-066742

Nolan TS, Bell AM, Chan YN, Leak Bryant A, Bissram JS, Hirschey R. Use of Video Education Interventions to Increase Racial and Ethnic Diversity in Cancer Clinical Trials: A Systematic Review. Worldviews Evid Based Nurs. 2021 Oct;18(5):302-309. doi: 10.1111/wvn.12539. Epub 2021 Sep 24. PMID: 34561957; PMCID: PMC8483572.

Nouvini R, Parker PA, Malling CD, Godwin K, Costas-Muñiz R. Interventions to increase racial and ethnic minority accrual into cancer clinical trials: A systematic review. Cancer. 2022 Nov 1;128(21):3860-3869. doi: 10.1002/cncr.34454. Epub 2022 Sep 15. PMID: 36107740; PMCID: PMC10456972.

Ojha RP, Jackson BE, Lu Y, Burton M, Blair SE, MacDonald BR, Chu TC, Teigen KJ, Acosta M. Participation and retention can be high in randomized controlled trials targeting underserved populations: a systematic review and meta-analysis. J Clin Epidemiol. 2018 Jun;98:154-157. doi: 10.1016/j.jclinepi.2017.11.014. Epub 2017 Nov 26. PMID: 29183689.

Parkinson, B., Meacock, R., Sutton, M. et al. Designing and using incentives to support recruitment and retention in clinical trials: a scoping review and a checklist for design. Trials 20, 624 (2019). https://doi.org/10.1186/s13063-019-3710-z

Raper SE, Clapp JT, Fleisher LA. Improving Surgical Informed Consent: Unanswered Questions. Ann Surg Open. 2021 Jan 7;2(1):e030. doi: 10.1097/AS9.0000000000000030. PMID: 37638239; PMCID: PMC10455139.

Rogers A, De Paoli G, Subbarayan S, Copland R, Harwood K, Coyle J, Mitchell L, MacDonald TM, Mackenzie IS; Trials@Home Consortium. A systematic review of methods used to conduct decentralised clinical trials. Br J Clin Pharmacol. 2022 Jun;88(6):2843-2862. doi: 10.1111/bcp.15205. Epub 2022 Jan 27. PMID: 34961991; PMCID: PMC9306873.

Sedrak MS, Freedman RA, Cohen HJ, Muss HB, Jatoi A, Klepin HD, Wildes TM, Le-Rademacher JG, Kimmick GG, Tew WP, George K, Padam S, Liu J, Wong AR, Lynch A, Djulbegovic B, Mohile SG, Dale W; Cancer and Aging Research Group (CARG). Older adult participation in cancer clinical trials: A systematic review of barriers and interventions. CA Cancer J Clin. 2021 Jan;71(1):78-92. doi: 10.3322/caac.21638. Epub 2020 Oct 1. PMID: 33002206; PMCID: PMC7854940.

Townsend D, Mills N, Savović J, Donovan JL. A systematic review of training programmes for recruiters to randomised controlled trials. Trials. 2015 Sep 28;16:432. doi: 10.1186/s13063-015-0908-6. PMID: 26416143; PMCID: PMC4587840.

Unger JM, Vaidya R, Hershman DL, Minasian LM, Fleury ME. Systematic Review and Meta-Analysis of the Magnitude of Structural, Clinical, and Physician and Patient Barriers to Cancer Clinical Trial Participation. J Natl Cancer Inst. 2019 Mar 1;111(3):245-255. doi: 10.1093/jnci/djy221. PMID: 30856272; PMCID: PMC6410951.

Vuong I, Wright J, Nolan MB, Eggen A, Bailey E, Strickland R, Traynor A, Downs T. Overcoming Barriers: Evidence-Based Strategies to Increase Enrollment of Underrepresented Populations in Cancer Therapeutic Clinical Trials-a Narrative Review. J Cancer Educ. 2020 Oct;35(5):841-849. doi: 10.1007/s13187-019-01650-y. PMID: 31713103.

Wong CA, Song WB, Jiao M, O'Brien E, Ubel P, Wang G, Scales CD. Strategies for research participant engagement: A synthetic review and conceptual framework. Clin Trials. 2021 Aug;18(4):457-465. doi: 10.1177/17407745211011068. Epub 2021 May 20. PMID: 34011179.
