# Supplementary material for: Strategies to improve recruitment to randomised trials
Source: Cochrane Database Syst Rev. 2026 Sep 15;2026(9):MR000013. doi: 10.1002/14651858.MR000013.pub7 (PMC13576036; doi:10.1002/14651858.MR000013.pub7)
Supplement: Supplementary file 8 [file MR000013-SUP-08-other.html]

Full list of strategies


# Supplementary material 8 to: Strategies to improve recruitment to randomised trials

Parker A, Mongelli G, Piccolo-Lawrance C, Coleman E, Gkekas A, Wang HI, Dawson S, Green H, Way R, Rai A, Bruhn H, Sullivan F, Boxall C, Chan M, Manson P, Hewitt C, Fairhurst C, Clarke M, Treweek S
  
https://doi.org/10.1002/14651858.MR000013.pub7

The material in this section has been supplied by the author(s) for publication under a Licence for Publication and the author(s) are solely responsible for the material. Cochrane has reviewed this material, but Cochrane has not copyedited, formatted or proofread. Cochrane accordingly gives no representations or warranties of any kind in relation to, and accepts no liability for any reliance on or use of, such material.

Back to top

# Full list of strategies

### Category A: Design

1. Open-label RCT versus blinded RCT (GRADE: high; Analysis 1.1)
2. Patient preference design versus conventional RCT design (GRADE: low; Analysis 2.1)
3. Electronic data capture versus paper-based data capture (GRADE: low; Analysis 3.1)
4. Cluster randomisation versus Zelen design (risk of bias: low; Fowell 2006)
5. Two-stage randomisation to choose duration of treatment versus single randomisation (low risk of bias; Paul 2011)

### Category B: Pre‐trial planning

1. Pre-engagement with potential trial sites during proposal development vs no pre-engagement (GRADE: low; Analysis 6.1)

### Category C: Trial conduct changes

1. Telephone reminder versus no telephone reminder (GRADE: high; Analysis 7.1)
2. SMS reminder mentioning scarcity vs SMS reminder with no mention (GRADE: moderate; Analysis 8.1)
3. SMS messages containing quotes from existing participants vs no messages (GRADE: moderate; Analysis 9.1)
4. Email invitation versus postal invitation (GRADE: moderate; Analysis 10.1)
5. Telephone reminders vs SMS reminders (GRADE: moderate; Analysis 11.1)

### Category D: Modification to the consent process

1. Opt-out consent versus opt-in consent (GRADE: low; Analysis 12.1)
2. Researcher reading out consent versus participant reading consent (GRADE: very low; Analysis 13.1)
3. Easy to read consent versus standard consent (unclear risk of bias; Coyne 2003)
4. Information printed on heavyweight paper and blue folio versus standard (high risk of bias; Abd-Elsayed 2012)
5. Enhanced pre-consent discussion with aid of poster versus standard consent (high risk of bias; Lipstein 2021)

### Category E: Modification to the information given to potential participants about the trial

1. Brief participant information leaflet (PIL) vs full PIL (GRADE: moderate; Analysis 17.1)
2. Study-related questionnaire + trial invitation versus trial invitation (GRADE: moderate; Analysis 18.1)
3. PIL developed with feedback from users vs usual PIL (GRADE: moderate; Analysis 19.1)
4. Enhanced recruitment package + recruitment at churches versus standard recruitment package (GRADE: low; Analysis 20.1)
5. Enhanced recruitment package versus standard recruitment package (GRADE: low; Analysis 21.1)
6. Enhanced recruitment package + baseline data over telephone versus standard recruitment package (GRADE: low; Analysis 22.1)
7. Audio record of information given about trial vs no audio record (GRADE: very low; Analysis 23.1)
8. Clinical trial booklet + standard information versus standard information (GRADE: very low; Analysis 24.1)
9. Total information disclosure versus standard disclosure (GRADE: very low; Analysis 25.1)
10. Access to cancer trials website vs no access (high risk of bias; Analysis 26.1)
11. Bespoke user-tested PIL vs usual PIL (GRADE: high; Analysis 27.1)
12. Recruitment primer letter vs no letter (GRADE: low; Analysis 28.1)
13. Information provided over telephone vs information provided face-to-face (GRADE: moderate; Analysis 29.1)
14. Providing information by video versus standard information (GRADE: very low; Analysis 30.1)
15. Invitation letter emphasising ethnicity-specific disease risk vs standard letter (GRADE: low; Analysis 31.1)
16. Handwritten personalised invitation letter vs standard letter (GRADE: low; Analysis 32.1; Analysis 32.2)
17. Different website wording, including infographic, about the risk of underlying condition (GRADE: Very low; Analysis 33.1)
18. Shorter recruitment package vs. Standard recruitment package (GRADE: very low; Analysis 34.1)
19. Shorter recruitment pack with simplified consent vs. Standard recruitment package (GRADE: very low; Analysis 35.1)
20. Leaflet advertising PPI involvement in trial + standard trial invitation vs standard trial invitiation only (GRADE: moderate; Analysis 36.1)
21. Gender-targeted recruitment postcard vs generic recruitment postcard (GRADE: low; Analysis 37.1)
22. Researcher optimised PIL vs standard PIL (GRADE: low; Analysis 38.1)
23. In-clinic information video viewing vs information DVD to take home (high risk of bias; Analysis 39.1)
24. Brief PIL + standard PIL vs standard PIL (GRADE: low; Analysis 40.1)
25. Decision aid about RCTs vs blank notebook (GRADE: low; Analysis 41.1)
26. Interactive online digital eductional tool vs time-matched webiner (GRADE: low; Analysis 42.1)
27. PPI designed and informed PIL vs standard researcher designed PIL (GRADE: very lowl; Analysis 43.1; Analysis 43.2)
28. Culturally-tailored educational DVD about clinical trials + usual education vs usual education by healthcare staff only (high risk of bias; Analysis 44.1)
29. Multimedia education about clinical trials (DVD + booklet) vs standard educational booklet only (GRADE: low; Analysis 45.1)
30. PIL minimising the use of phrases with negative connotations vs standard PIL (high risk of bias; Analysis 46.1)
31. Multimedia information (viewed in person) + paper PIL vs paper PIL only (GRADE: very low; Analysis 47.1; Analysis 47.2)
32. Multimedia information (viewed in person) PIL vs paper PIL (GRADE: moderate; Analysis 48.1; Analysis 48.2)
33. Multimedia information (provided as link/QR code) + paper PIL vs paper PIL (GRADE: high; Analysis 49.1; Analysis 49.2)
34. Multimedia information (provided as link/QR code) vs paper PIL (GRADE: moderate;Analysis 50.1; Analysis 50.2)
35. Educational material to provide additional information about a trial versus standard information alone (unclear risk of bias; Kimmick 2005)
36. Trained recruiters from a similar ethnic background to study population already taking part in a trial as lay advocates (unclear risk of bias; Larkey 2002)
37. Coach to support recruitment of minority participants versus no coach (high risk of bias; Fracasso 2013)

### Category F: Interventions aimed at the recruiter or recruitment site

1. Teaser campaign using postcards vs no teaser (GRADE: moderate; Analysis 54.1)
2. Educational program to tailor recruitment strategies for minority recruitment vs usual recruitment (GRADE: low; Analysis 55.1)
3. Remote vs face-to-face site initiation visits (GRADE: moderate; Analysis 56.1; Analysis 56.2)
4. Recruitment coordinator plus training vs usual recruitment (high risk of bias; Maxwell 2017)
5. Using teleconference and commitment contracts with site teams to enhance recruitment (Unclear risk of bias, Isaksson 2025)
6. Onsite initiation visit versus no initiation visit (unclear risk of bias; Liénard 2006)
7. Additional communication from central trial coordinator to sites versus standard communication (low risk of bias; Monaghan 2007)
8. Recruiter training with ongoing support and/or theory-informed personalised nudge to the recruiter after each randomisation may increase recruitment (low risk of bias; Agni 2022)
9. Automatically flagging eligible patients to clinicians via electronic health records vs. manually identifying patients via electronic health records (high risk of bias; Ethier 2017)

### Category G: Incentives

1. Monetary incentive vs no incentive (GRADE: low; Analysis 63.1)
2. Higher versus lower monetary incentive (GRADE: very low; Analysis 64.1)
3. Non-monetary incentive (branded pen vs no pen) (GRADE: moderate; Analysis 65.1)
