# Supplementary material for: Strategies to improve recruitment to randomised trials
Source: Cochrane Database Syst Rev. 2026 Sep 15;2026(9):MR000013. doi: 10.1002/14651858.MR000013.pub7 (PMC13576036; doi:10.1002/14651858.MR000013.pub7)
Supplement: Supplementary file 9 [file MR000013-SUP-09-other.html]

Participant numbers per study


# Supplementary material 9 to: Strategies to improve recruitment to randomised trials

Parker A, Mongelli G, Piccolo-Lawrance C, Coleman E, Gkekas A, Wang HI, Dawson S, Green H, Way R, Rai A, Bruhn H, Sullivan F, Boxall C, Chan M, Manson P, Hewitt C, Fairhurst C, Clarke M, Treweek S
  
https://doi.org/10.1002/14651858.MR000013.pub7

The material in this section has been supplied by the author(s) for publication under a Licence for Publication and the author(s) are solely responsible for the material. Cochrane has reviewed this material, but Cochrane has not copyedited, formatted or proofread. Cochrane accordingly gives no representations or warranties of any kind in relation to, and accepts no liability for any reliance on or use of, such material.

Back to top

# Participant numbers per study

| **Category A - Design** | | | | | |
| --- | --- | --- | --- | --- | --- |
| **Studies presented in the results** | | | **High** risk of bias not presented in the results | | |
| *Study* | *N participants* | *N clusters* | *Study* | *N participants* | *N clusters* |
| Avenell 2004  Cooper 1997  Fowell 2006  Hemminki 2004  Litchfield 2005  Paul 2011  Veerus 2016 | 538 273 53 4,295 80 398  4171 | - - - - 28 - - | None | 0 | — |
| **Total** | 9,808 | 28 | **Total** | 0 | — |
| **Category B - pre-trial planning** | | | | | |
| **Studies presented in the results** | | | **High** risk of bias not presented in the results | | |
| *Study* | *N participants* | *N clusters* | *Study* | *N participants* | *N clusters* |
| Garvelink 2015 | - | 33 | None | 0 | — |
| **Total** | - | 33 | **Total** | 0 | — |
| **Category C - Trial conduct changes** | | | | | |
| **Studies presented in the results** | | | **High** risk of bias not presented in the results | | |
| *Study* | *N participants* | *N clusters* | *Study* | *N participants* | *N clusters* |
| Bracken 2019  Free 2010^a^  Free 2011  Nystuen 2004  Treweek 2012  Wong 2013 | 709  811 1,862 498 1,760 952 | — | None | 0 | — |
| **Total** | 6,592 |  | **Total** | 0 | — |
| **Category D - Modification to the consent process** | | | | | |
| **Studies presented in the results** | | | **High** risk of bias not presented in the results | | |
| *Study* | *N participants* | *N clusters* | *Study* | *N participants* | *N clusters* |
| Coyne 2003  Trevena 2006  Wadland 1990 | 226 152 104 | — | Abd-Elsayed 2012  Lipstein 2021 | 499  265 | -  43 |
| **Total** | 482 | — | **Total** | 764 | 43 |
| **Category E - Modification to the information given to potential participants about the trial** | | | | | |
| **Studies presented in the results** |  |  | **High** risk of bias not presented in the results | | |
| *Study* | *N participants* | *N clusters* | *Study* | *N participants* | *N clusters* |
| Arundel 2017  Bergenmar 2014  Bobb 2016 Brierley 2012  Brown 2015  Chen 2011  Chow 2020 Cockayne 2017 (START PIL REFORM)  Crane 2016 Du 2008  Du 2009  Dwyer 2022 Ford 2004  Foss 2016  Fracasso 2013  Hughes-Morley 2016 Hutchison 2007  Ives 2001  Jolly 2019 (START MMI PSM-COPD)  Kamen 2018 Kendrick 2001  Kimmick 2005  Knapp 2020 (START PIS CASPER)^a^  Knapp 2023e (Thermic-3)^e^  Knapp 2023a (BALANCE)^e^  Knapp 2023b (BAMP)^e^  Knapp 2023c (CHAMP-UK)^e^  Knapp 2023d (FORCE)^e^  Knapp 2023f (UKALL)^e^  Larkey 2002  Man 2015 (START PIS Healthlines Depression)  Madurasinghe 2021 (START PIS HeLP Diabetes)  Madurasinghe 2023a (START MMI GHT2000a)^b^  Madurasinghe 2023b (START MMI GHT2000b)^b^  Madurasinghe 2023c (START MMI HI-Light)^b^  Madurasinghe 2023d (START MMI seAFOod)^b^  Mattock 2020  McCaffery 2019  Miller 2021a  Paris 2015  Parker 2018 (START PIS ECLS)^a^  Paul 2014  Rogers 2019  Saif 2020  Sheridan 2020 (START PIS ISDR)^a^  Simes 1986  Sundaresan 2017  White 2023^c^ | 4,314  130  131  2330  445  14,467  259  6,900  30,000 126 196  207  12,400 118  60  8,182 173 50  4,214  418  2393 126  11,531  153  1,792  1,792  1,792  1,409  1,792 15  1364  4,880  11,381  -  -  -  107  317  2,605  1,062  480  2,262  1062  1,050  351  57  815  6,946 148 | — | Dear 2011  Felicitas-Perkins 2017  Skinner 2019  Witham 2018 | 340 37  63  4,145 | — |
| **Total** | 142,802 | — | **Total** | 4,585 | — |
| **Category F - Interventions aimed at the recruiter or recruitment site** | | | | | |
| **Studies presented in the results** | | | **High** risk of bias not presented in the results | | |
| *Study* | *N participants* | *N clusters* | *Study* | *N participants* | *N clusters* |
| Agni 2022  Isaksson 2025  Jefferson 2018  Lee 2017  Liénard 2006  Maxwell 2017  Monaghan 2007  Tilley 2021 | 1,215  -  480  670  573  -  -  2,107 | 20  20  37  -  -  72  167  - | Ethier 2017 Tilley 2012 | 600  606 | -  32 |
| **Total** | 5,045 | 316 | **Total** | 1,206 | 32 |
| **Category G - Incentives** | | | | | |
| **Studies presented in the results** | | | **High** risk of bias not presented in the results | | |
| *Study* | *N participants* | *N clusters* | *Study* | *N participants* | *N clusters* |
| Fairhurst 2022  Free 2010^a^  Giguere 2015  Halpern 2021a ($200)  Halpern 2021b ($100)  Jennings 2015a ^d^  Jennings 2015b ^d^  Jennings 2015c ^d^  Jennings 2015d ^d^  Jennings 2015e ^d^  White 2023^c^  Whiteside 2019 | 818  491  1,314  654  642  181  332  93  210  199  6,946  1,862 | — | None | 0 | — |
| **Total** | 13,742 | — | **Total** | 0 | — |
| **Overall totals** | | | | | |
| **Studies presented in the results** | | | **High** risk of bias not presented in the results | | |
| *N studies* | *N participants* | *N clusters* | *N studies* | *N participants* | *N clusters* |
| 83 | 176,459 | 377 | 8 | 6,555 | 75 |
| **All** risk of bias | | | | | |
| *N studies* | | | | *N participants* | *N clusters* |
| 91 | | | | 183,014 | 752 |

^a^Contained two interventions (see Category G).  
^b^Both included in same article.  
^c^Included two interventions (see Category G).  
^d^All included in same article.)  
^e^All included in same article.
