# Supplementary material for: Strategies to improve recruitment to randomised trials
Source: Cochrane Database Syst Rev. 2026 Sep 15;2026(9):MR000013. doi: 10.1002/14651858.MR000013.pub7 (PMC13576036; doi:10.1002/14651858.MR000013.pub7)
Supplement: Supplementary file 10 [file MR000013-SUP-10-other.html]

Data and analyses


# Supplementary material 10 to: Strategies to improve recruitment to randomised trials

Parker A, Mongelli G, Piccolo-Lawrance C, Coleman E, Gkekas A, Wang HI, Dawson S, Green H, Way R, Rai A, Bruhn H, Sullivan F, Boxall C, Chan M, Manson P, Hewitt C, Fairhurst C, Clarke M, Treweek S
  
https://doi.org/10.1002/14651858.MR000013.pub7

The material in this section has been supplied by the author(s) for publication under a Licence for Publication and the author(s) are solely responsible for the material. Cochrane has reviewed this material, but Cochrane has not copyedited, formatted or proofread. Cochrane accordingly gives no representations or warranties of any kind in relation to, and accepts no liability for any reliance on or use of, such material.

Back to top

# Data and analyses

## Category A: Design

**Note**: EDI data reporting was very limited for studies in this category; interpret results with caution regarding generalisability to under-served groups.

### Comparison 1A–Open-label trial vs blinded trial (GRADE: high)

#### Analysis 1.1 Participants recruited

|  |  |  |  |  |
| --- | --- | --- | --- | --- |
| **Outcome or subgroup title** | **No. of studies** | **No. of participants** | **Statistical method** | **Effect size** |
| 1 Participants recruited | 3 | 9004 | Risk difference (IV, random, 95% CI) | 0.10 [0.08,0.12] |

### Comparison 2A–Patient preference design vs conventional RCT (GRADE: low)

#### Analysis 2.1 Participants recruited

|  |  |  |  |  |
| --- | --- | --- | --- | --- |
| **Outcome or subgroup title** | **No. of studies** | **No. of participants** | **Statistical method** | **Effect size** |
| 1 Participants recruited | 1 | 273 | Risk difference  M-H, Fixed, 95% CI | -0.04 [-0.15 , 0.07] |

### Comparison 3A–Electronic data capture vs paper-based data capture (GRADE: low)

#### Analysis 3.1 Participants recruited

|  |  |  |  |  |
| --- | --- | --- | --- | --- |
| **Outcome or subgroup title** | **No. of studies** | **No. of participants** | **Statistical method** | **Effect size** |
| 1 Participants recruited | 1 | 80 | Risk difference  M-H, Fixed, 95% CI | -0.13 [-0.24 , -0.03] |

### Comparison 4A–Cluster‐randomised design compared to Zelen design (GRADE: unable to complete, no forest plot available)

#### Comparison 4.1 Participants recruited

|  |  |  |  |  |
| --- | --- | --- | --- | --- |
| **Outcome or subgroup title** | **No. of studies** | **No. of participants** | **Statistical method** | **Effect size** |
| 1 Participants recruited | 1 | 53 | N/A | N/A |

We were unable to generate a forest plot for this comparison as the data required to do so were not reported in the study (Fowell 2006). The study had only two sites (clusters) with few participants: 6 out of 24 potential participants were recruited in the cluster arm, compared to 0 out of 29 in the Zelen arm; RoB: low. We were also unable to complete a GRADE assessment for this comparison due to lack of data.

### Comparison 5A–Two‐stage randomisation to choose duration of treatment (GRADE: unable to complete, no forest plot available)

#### Comparison 5.1: Participants recruited

|  |  |  |  |  |
| --- | --- | --- | --- | --- |
| **Outcome or subgroup title** | **No. of studies** | **No. of participants** | **Statistical method** | **Effect size** |
| 1 Participants recruited | 1 | 215 | N/A | N/A |

We were unable to generate a forest plot for this comparison as the data required to do so were not reported in the study (Paul, 2006). Data on numbers recruited were not available for one arm but up‐front randomisation to 3 or 6 months treatment gave a recruitment rate of 5.21 per year per centre compared to 4.09 for delayed randomisation to decide whether a second 3 month treatment was given. We were also unable to complete a GRADE assessment for this comparison due to lack of data.

## Category B: Pre‐trial planning

**Note:** EDI data were not reported for studies in this category; interpret results with caution regarding generalisability to under-served groups.

### Comparison 6B–Pre-engagement with potential trial sites during proposal development vs no pre-engagement (GRADE: low)

#### Analysis 6.1: Participants recruited

|  |  |  |  |  |
| --- | --- | --- | --- | --- |
| **Outcome or subgroup title** | **No. of studies** | **No. of participants** | **Statistical method** | **Effect size** |
| 1 Participants recruited | 1 | 33 | Risk difference  M-H, Fixed, 95% CI | 0.50 [0.18 , 0.81] |

## Category C: Trial conduct changes

**Note**: EDI data reporting was very limited for studies in this category; interpret results with caution regarding generalisability to under-served groups.

### Comparison 7C–Telephone reminder vs no telephone reminder (GRADE: high)

#### Analysis 7.1: Participants recruited

|  |  |  |  |  |
| --- | --- | --- | --- | --- |
| **Outcome or subgroup title** | **No. of studies** | **No. of participants** | **Statistical method** | **Effect size** |
| 1 Participants recruited | 2 | 1450 | Risk difference  IV, Random, 95% CI | 0.06 [0.03 , 0.09] |

### Comparison 8C–SMS reminder mentioning scarcity vs SMS reminder with no mention (GRADE: moderate)

#### Analysis 8.1: Participants recruited

|  |  |  |  |  |
| --- | --- | --- | --- | --- |
| **Outcome or subgroup title** | **No. of studies** | **No. of participants** | **Statistical method** | **Effect size** |
| 1 Participants recruited | 1 | 1862 | Risk difference  M-H, Fixed, 95% CI | 0.03 [0.01 , 0.06] |

### Comparison 9C–SMS messages containing quotes from existing participants vs no messages (GRADE: moderate)

#### Analysis 9.1: Participants recruited

|  |  |  |  |  |
| --- | --- | --- | --- | --- |
| **Outcome or subgroup title** | **No. of studies** | **No. of participants** | **Statistical method** | **Effect size** |
| 1 Participants recruited | 1 | 811 | Risk difference  M-H, Fixed, 95% CI | 0.04 [0.02 , 0.06] |

### Comparison 10C–Email invitation vs postal invitation (GRADE: moderate)

#### Analysis 10.1: Participants recruited

|  |  |  |  |  |
| --- | --- | --- | --- | --- |
| **Outcome or subgroup title** | **No. of studies** | **No. of participants** | **Statistical method** | **Effect size** |
| 1 Participants recruited | 1 | 1760 | Risk difference  M-H, Fixed, 95% CI | 0.01 [-0.03 , 0.04] |

### Comparison 11C–Telephone reminders vs SMS reminders (GRADE: moderate)

#### Analysis 11.1: Participants recruited

|  |  |  |  |  |
| --- | --- | --- | --- | --- |
| **Outcome or subgroup title** | **No. of studies** | **No. of participants** | **Statistical method** | **Effect size** |
| 1 Participants recruited | 1 | 709 | Risk difference  M-H, Fixed, 95% CI | 0.05 [-0.01 , 0.11] |

## Category D: Modification to the consent process

**Note**: EDI data reporting was very limited for studies in this category; interpret results with caution regarding generalisability to under-served groups.

### Comparison 12D–Opt-out consent vs opt-in consent (GRADE: low)

#### Analysis 12.1: Participants recruited

|  |  |  |  |  |
| --- | --- | --- | --- | --- |
| **Outcome or subgroup title** | **No. of studies** | **No. of participants** | **Statistical method** | **Effect size** |
| 1 Participants recruited | 1 | 152 | Risk difference  M-H, Fixed, 95% CI | 0.19 [0.03 , 0.35] |

### Comparison 13D–Researcher reading out consent vs participant reading consent (GRADE: very low)

#### Analysis 13.1: Participants recruited

|  |  |  |  |  |
| --- | --- | --- | --- | --- |
| **Outcome or subgroup title** | **No. of studies** | **No. of participants** | **Statistical method** | **Effect size** |
| 1 Participants recruited | 1 | 104 | Risk difference  M-H, Fixed, 95% CI | 0.06 [-0.13 , 0.25] |

### Comparison 14D-Easy to read consent form (GRADE: unable to complete, no forest plot available)

#### Comparison 14.1: Participants recruited

|  |  |  |  |  |
| --- | --- | --- | --- | --- |
| **Outcome or subgroup title** | **No. of studies** | **No. of participants** | **Statistical method** | **Effect size** |
| 1 Participants recruited | 1 | 226 | N/A | N/A |

We were unable to generate a forest plot for this comparison as the data required to do so were not reported in the study (Coyne 2003). The authors of this cluster trial did not present centre‐level recruitment data, nor provide an intracluster correlation coefficient. We were also unable to complete a GRADE assessment for this comparison due to lack of data.

### Comparison 15D–Information printed on heavyweight paper and blue folio vs standard (high risk of bias)

**Note:** This is a single study, with high risk of bias. We do not present the results in the main manuscript.

#### Analysis 15.1: Participants recruited

|  |  |  |  |  |
| --- | --- | --- | --- | --- |
| **Outcome or subgroup title** | **No. of studies** | **No. of participants** | **Statistical method** | **Effect size** |
| 1 Participants recruited | 1 | 499 | Risk difference  M-H, Fixed, 95% CI | -0.09 [-0.17, -0.01] |

### Comparison 16D–Enhanced pre-consent discussion with aid of poster vs. standard consent (high risk of bias)

**Note:** This is a single study, with high risk of bias. We do not present the results in the main manuscript.

#### Analysis 16.1: participants recruited

|  |  |  |  |  |
| --- | --- | --- | --- | --- |
| **Outcome or subgroup title** | **No. of studies** | **No. of participants** | **Statistical method** | **Effect size** |
| 1 Participants recruited | 1 | 769 | Risk difference  M-H, Fixed, 95% CI | -0.01 [-0.08 , 0.06] |

## Category E: Modification to the information given to potential participants about the trial

**Note**: EDI data reporting was very limited for studies in this category; interpret results with caution regarding generalisability to under-served groups.

### Comparison 17E–Brief participant information leaflet (PIL) vs full PIL (GRADE: moderate)

#### Analysis 17.1: participants recruited

|  |  |  |  |  |
| --- | --- | --- | --- | --- |
| **Outcome or subgroup title** | **No. of studies** | **No. of participants** | **Statistical method** | **Effect size** |
| 1 Participants recruited | 2 | 4633 | Risk difference  IV, Random, 95% CI | 0.00 [-0.01 , 0.02] |

### Comparison 18E–Study-related questionnaire + trial invitation vs trial invitation (GRADE: moderate)

#### Analysis 18.1 Participants recruited

|  |  |  |  |  |
| --- | --- | --- | --- | --- |
| **Outcome or subgroup title** | **No. of studies** | **No. of participants** | **Statistical method** | **Effect size** |
| 1 Participants recruited | 1 | 2393 | Risk difference  M-H, Fixed, 95% CI | 0.05 [0.02 , 0.08] |

### Comparison 19E–PIL developed with feedback from users vs usual PIL (GRADE: moderate)

#### Analysis 19.1 Participants recruited

|  |  |  |  |  |
| --- | --- | --- | --- | --- |
| **Outcome or subgroup title** | **No. of studies** | **No. of participants** | **Statistical method** | **Effect size** |
| 1 Participants recruited | 2 | 16763 | Risk difference  IV, Random, 95% CI | 0.00 [-0.00 , 0.01] |

### Comparison 20E–Enhanced recruitment package + recruitment at churches vs standard recruitment package (GRADE: low)

#### Analysis 20.1: Participants recruited

|  |  |  |  |  |
| --- | --- | --- | --- | --- |
| **Outcome or subgroup title** | **No. of studies** | **No. of participants** | **Statistical method** | **Effect size** |
| 1 Participants recruited | 1 | 6246 | Risk difference  M-H, Fixed, 95% CI | 0.01 [0.00 , 0.02] |

### Comparison 21E–Enhanced recruitment package vs standard recruitment package (GRADE: low)

#### Analysis 21.1: Participants recruited

|  |  |  |  |  |
| --- | --- | --- | --- | --- |
| **Outcome or subgroup title** | **No. of studies** | **No. of participants** | **Statistical method** | **Effect size** |
| 1 Participants recruited | 1 | 6376 | Risk difference  M-H, Fixed, 95% CI | -0.00 [-0.01 , 0.00] |

### Comparison 22E–Enhanced recruitment package + baseline data over telephone vs standard recruitment package (GRADE: low)

#### Analysis 22.1 Participants recruited

|  |  |  |  |  |
| --- | --- | --- | --- | --- |
| **Outcome or subgroup title** | **No. of studies** | **No. of participants** | **Statistical method** | **Effect size** |
| 1 Participants recruited | 1 | 6372 | Risk difference  M-H, Fixed, 95% CI | -0.00 [-0.01 , 0.01] |

### Comparison 23E–Audio record of information given about trial vs no audio record (GRADE: very low)

#### Analysis 23.1 Participants recruited

|  |  |  |  |  |
| --- | --- | --- | --- | --- |
| **Outcome or subgroup title** | **No. of studies** | **No. of participants** | **Statistical method** | **Effect size** |
| 1 Participants recruited | 1 | 130 | Risk difference  M-H, Fixed, 95% CI | -0.03 [-0.19 , 0.13] |

### Comparison 24E–Clinical trial booklet + standard information vs standard information (GRADE: very low)

#### Analysis 24.1: Participants recruited

|  |  |  |  |  |
| --- | --- | --- | --- | --- |
| **Outcome or subgroup title** | **No. of studies** | **No. of participants** | **Statistical method** | **Effect size** |
| 1 Participants recruited | 1 | 31 | Risk difference  M-H, Fixed, 95% CI | 0.20 [-0.05 , 0.46] |

### Comparison 25E–Total information disclosure vs standard disclosure (GRADE: very low)

#### Analysis 25.1 Participants recruited

|  |  |  |  |  |
| --- | --- | --- | --- | --- |
| **Outcome or subgroup title** | **No. of studies** | **No. of participants** | **Statistical method** | **Effect size** |
| 1 Participants recruited | 1 | 57 | Risk difference  M-H, Fixed, 95% CI | 0.11 [-0.06 , 0.28] |

### Comparison 26E–Access to cancer trials website vs no access (high risk of bias)

**Note:** This is a single study, with high risk of bias. We do not present the results in the main manuscript.

#### Analysis 26.1 Participants recruited

|  |  |  |  |  |
| --- | --- | --- | --- | --- |
| **Outcome or subgroup title** | **No. of studies** | **No. of participants** | **Statistical method** | **Effect size** |
| 1 Participants recruited | 1 | 340 | Risk difference  M-H, Fixed, 95% CI | -0.01 [-0.07 , 0.06] |

### Comparison 27E–Bespoke, user-tested PIL vs usual PIL (GRADE: high)

#### Analysis 27.1 Participants recruited

|  |  |  |  |  |
| --- | --- | --- | --- | --- |
| **Outcome or subgroup title** | **No. of studies** | **No. of participants** | **Statistical method** | **Effect size** |
| 1 Participants recruited | 6 | 27805 | Risk difference  IV, Random, 95% CI | 0.00 [-0.00 , 0.01] |

### Comparison 28E–Recruitment primer letter/leaflet vs no letter/leaflet (GRADE: High)

#### Analysis 28.1 Participants recruited

|  |  |  |  |  |
| --- | --- | --- | --- | --- |
| **Outcome or subgroup title** | **No. of studies** | **No. of participants** | **Statistical method** | **Effect size** |
| 1 Participants recruited | 2 | 5376 | Risk difference  IV, Random, 95% CI | 0.01 [-0.01 , 0.02] |

### Comparison 29E–Information provided remotely vs information provided face-to-face (GRADE: Moderate)

#### Analysis 29.1 Participants recruited

|  |  |  |  |  |
| --- | --- | --- | --- | --- |
| **Outcome or subgroup title** | **No. of studies** | **No. of participants** | **Statistical method** | **Effect size** |
| 1 Participants recruited | 2 | 249 | Risk difference  IV, Random, 95% CI | 0.01 [-0.01 , 0.02] |

### Comparison 30E–Providing information by video vs standard information (GRADE: very low)

#### Analysis 30.1 Participants recruited

|  |  |  |  |  |
| --- | --- | --- | --- | --- |
| **Outcome or subgroup title** | **No. of studies** | **No. of participants** | **Statistical method** | **Effect size** |
| 1 Participants recruited | 5 | 1634 | Risk difference  IV, Random, 95% CI | -0.04 [-0.23 , 0.15] |

### Comparison 31E–Invitation letter emphasising ethnicity-specific disease risk vs standard letter (GRADE: low)

#### Analysis 31.1 Participants recruited

|  |  |  |  |  |
| --- | --- | --- | --- | --- |
| **Outcome or subgroup title** | **No. of studies** | **No. of participants** | **Statistical method** | **Effect size** |
| 1 Participants recruited | 1 | 445 | Risk difference  M-H, Fixed, 95% CI | -0.03 [-0.12 , 0.06] |

### Comparison 32E–Handwritten personalised invitation letter vs standard letter (GRADE: low)

#### Analysis 32.1 Participants recruited

|  |  |  |  |  |
| --- | --- | --- | --- | --- |
| **Outcome or subgroup title** | **No. of studies** | **No. of participants** | **Statistical method** | **Effect size** |
| 1 Participants recruited | 1 | 317 | Risk difference  M-H, Fixed, 95% CI | -0.04 [-0.08 , 0.00] |

### Comparison 33E–Different website wording, including infographic, about the risk of underlying condition (GRADE: Very low)

#### Analysis 33.1 Participants recruited

|  |  |  |  |  |
| --- | --- | --- | --- | --- |
| **Outcome or subgroup title** | **No. of studies** | **No. of participants** | **Statistical method** | **Effect size** |
| 1 Participants recruited | 2 | 2605 | Risk difference  IV, Random, 95% CI | -0.04 [-0.23 , 0.15] |

### Comparison 34E– Shorter recruitment package vs. Standard recruitment package (GRADE: very low)

#### Analysis 35.1 Participants recruited

|  |  |  |  |  |
| --- | --- | --- | --- | --- |
| **Outcome or subgroup title** | **No. of studies** | **No. of participants** | **Statistical method** | **Effect size** |
| 1 Participants recruited | 1 | 170 | Risk difference  M-H, Fixed, 95% CI | 0.02 [-0.13 , 0.17] |

### Comparison 35E– Shorter recruitment pack with simplified consent vs. Standard recruitment package (GRADE: very low)

#### Analysis 35.1 Participants recruited

|  |  |  |  |  |
| --- | --- | --- | --- | --- |
| **Outcome or subgroup title** | **No. of studies** | **No. of participants** | **Statistical method** | **Effect size** |
| 1 Participants recruited | 1 | 175 | Risk difference  M-H, Fixed, 95% CI | 0.02 [-0.13 , 0.17] |

### Comparison 36E–Leaflet advertising PPI involvement in trial + standard trial invitation vs standard trial invitiation only (GRADE: moderate)

#### Analysis 36.1 Participants recruited

|  |  |  |  |  |
| --- | --- | --- | --- | --- |
| **Outcome or subgroup title** | **No. of studies** | **No. of participants** | **Statistical method** | **Effect size** |
| 1 Participants recruited | 1 | 8182 | Risk difference  M-H, Fixed, 95% CI | -0.01 [-0.02 , -0.00] |

### Comparison 37E–Gender-targeted recruitment postcard vs generic recruitment postcard (GRADE: low)

#### Analysis 37.1 Participants recruited

|  |  |  |  |  |
| --- | --- | --- | --- | --- |
| **Outcome or subgroup title** | **No. of studies** | **No. of participants** | **Statistical method** | **Effect size** |
| 1 Participants recruited | 1 | 30000 | Risk difference  M-H, Fixed, 95% CI | 0.00 [-0.00 , 0.00] |

### Comparison 38E–Researcher optimised PIL vs standard PIL (GRADE: low)

#### Analysis 38.1 Participants recruited

|  |  |  |  |  |
| --- | --- | --- | --- | --- |
| **Outcome or subgroup title** | **No. of studies** | **No. of participants** | **Statistical method** | **Effect size** |
| 1 Participants recruited | 1 | 480 | Risk difference  M-H, Fixed, 95% CI | -0.09 [-0.17 , -0.00] |

### Comparison 39E–In-clinic information video viewing vs information DVD to take home (high risk of bias)

**Note:** This is a single study, with high risk of bias. We do not present the results in the main manuscript.

#### Analysis 39.1 Participants recruited

|  |  |  |  |  |
| --- | --- | --- | --- | --- |
| **Outcome or subgroup title** | **No. of studies** | **No. of participants** | **Statistical method** | **Effect size** |
| 1 Participants recruited | 1 | 79 | Risk difference  M-H, Fixed, 95% CI | 0.03 [-0.08 , 0.14] |

### Comparison 40E–Brief PIL + standard PIL vs standard PIL (GRADE: low)

#### Analysis 40.1 Participants recruited

|  |  |  |  |  |
| --- | --- | --- | --- | --- |
| **Outcome or subgroup title** | **No. of studies** | **No. of participants** | **Statistical method** | **Effect size** |
| 1 Participants recruited | 1 | 6946 | Risk difference  M-H, Fixed, 95% CI | 0.00 [-0.01 , 0.01] |

### Comparison 41E–Decision aid about RCTs vs blank notebook (GRADE: low)

#### Analysis 41.1 Participant recruited

|  |  |  |  |  |
| --- | --- | --- | --- | --- |
| **Outcome or subgroup title** | **No. of studies** | **No. of participants** | **Statistical method** | **Effect size** |
| 1 Participants recruited | 1 | 129 | Risk difference  M-H, Fixed, 95% CI | 0.12 [-0.01 , 0.24] |

### Comparison 42E–Interactive online digital eductional tool vs time-matched webiner (GRADE: low)

#### Analysis 42.1 Participants recruited

|  |  |  |  |  |
| --- | --- | --- | --- | --- |
| **Outcome or subgroup title** | **No. of studies** | **No. of participants** | **Statistical method** | **Effect size** |
| 1 Participants recruited | 1 | 351 | Risk difference  M-H, Fixed, 95% CI | 0.01 [-0.01 , 0.03] |

### Comparison 43E–PPI designed and informed PIL vs standard researcher designed PIL (GRADE: very low)

#### Analysis 43.1 Participants recruited

|  |  |  |  |  |
| --- | --- | --- | --- | --- |
| **Outcome or subgroup title** | **No. of studies** | **No. of participants** | **Statistical method** | **Effect size** |
| 1 Participants recruited | 1 | 207 | Risk difference  M-H, Fixed, 95% CI | -0.02 [-0.15 , 0.12] |

#### Analysis 43.2 Participants retained

|  |  |  |  |  |
| --- | --- | --- | --- | --- |
| **Outcome or subgroup title** | **No. of studies** | **No. of participants** | **Statistical method** | **Effect size** |
| 1 Participants retained | 1 | 207 | Risk difference  M-H, Fixed, 95% CI | 0.04 [-0.09 , 0.18] |

### Comparison 44E–Culturally-tailored educational DVD about clinical trials + usual education vs usual education by healthcare staff only (high risk of bias)

**Note:** This is a single study, with high risk of bias. We do not present the results in the main manuscript.

#### Analysis 44.1 Participants recruited

|  |  |  |  |  |
| --- | --- | --- | --- | --- |
| **Outcome or subgroup title** | **No. of studies** | **No. of participants** | **Statistical method** | **Effect size** |
| 1 Participants recruited | 1 | 37 | Risk difference  M-H, Fixed, 95% CI | 0.03 [-0.19 , 0.25] |

### Comparison 45E–Multimedia education about clinical trials (DVD + booklet) vs standard educational booklet only (GRADE: low)

#### Analysis 45.1 Participants recruited

|  |  |  |  |  |
| --- | --- | --- | --- | --- |
| **Outcome or subgroup title** | **No. of studies** | **No. of participants** | **Statistical method** | **Effect size** |
| 1 Participants recruited | 1 | 418 | Risk difference  M-H, Fixed, 95% CI | 0.04 [-0.05 , 0.14] |

### Comparison 46E–PIL minimising the use of phrases with negative connotations vs standard PIL (high risk of bias)

**Note:** This is a single study, with high risk of bias. We do not present the results in the main manuscript.

#### Analysis 46.1 Participants recruited

|  |  |  |  |  |
| --- | --- | --- | --- | --- |
| **Outcome or subgroup title** | **No. of studies** | **No. of participants** | **Statistical method** | **Effect size** |
| 1 Participants recruited | 1 | 4145 | Risk difference  M-H, Fixed, 95% CI | -0.01 [-0.02 , 0.00] |

### Comparison 47E–Multimedia information (viewed in person) + paper PIL vs paper PIL only (GRADE: very low)

#### Analysis 47.1 Participant recruitment

|  |  |  |  |  |
| --- | --- | --- | --- | --- |
| **Outcome or subgroup title** | **No. of studies** | **No. of participants** | **Statistical method** | **Effect size** |
| 1 Participants recruited | 3 | 23 | Risk difference  IV, Random, 95% CI | 0.17 [-0.89 , 1.23] |

#### Analysis 47.2 Participants retained

|  |  |  |  |  |
| --- | --- | --- | --- | --- |
| **Outcome or subgroup title** | **No. of studies** | **No. of participants** | **Statistical method** | **Effect size** |
| 1 Participants retained | 3 | 26 | Risk difference  IV, Random, 95% CI | 0.35 [-0.59 , 1.30] |

### Comparison 48E–Multimedia information (viewed in person) PIL vs paper PIL (GRADE: Moderate)

#### Analysis 48.1 Participants recruited

|  |  |  |  |  |
| --- | --- | --- | --- | --- |
| **Outcome or subgroup title** | **No. of studies** | **No. of participants** | **Statistical method** | **Effect size** |
| 1 Participants recruited | 3 | 1428 | Risk difference  IV, Random, 95% CI | 0.04 [-0.01 , 0.08] |

#### Analysis 48.2 Participants retained

|  |  |  |  |  |
| --- | --- | --- | --- | --- |
| **Outcome or subgroup title** | **No. of studies** | **No. of participants** | **Statistical method** | **Effect size** |
| 1 Participants retained | 3 | 26 | Risk difference  IV, Random, 95% CI | 0.28 [-0.51 , 1.06] |

### Comparison 49E–Multimedia information (provided as link/QR code) + paper PIL vs paper PIL (GRADE: high)

#### Analysis 49.1 Participants recruited

|  |  |  |  |  |
| --- | --- | --- | --- | --- |
| **Outcome or subgroup title** | **No. of studies** | **No. of participants** | **Statistical method** | **Effect size** |
| 1 Participants recruited | 7 | 11612 | Risk difference  IV, Random, 95% CI | -0.00 [-0.01 , 0.01] |

#### Analysis 49.2 Participants retained

|  |  |  |  |  |
| --- | --- | --- | --- | --- |
| **Outcome or subgroup title** | **No. of studies** | **No. of participants** | **Statistical method** | **Effect size** |
| 1 Participants retained | 5 | 7413 | Risk difference  IV, Random, 95% CI | 0.00 [-0.02 , 0.03] |

### Comparison 50E–Multimedia information (provided as link/QR code) vs paper PIL (GRADE: moderate)

#### Analysis 50.1 Participants recruited

|  |  |  |  |  |
| --- | --- | --- | --- | --- |
| **Outcome or subgroup title** | **No. of studies** | **No. of participants** | **Statistical method** | **Effect size** |
| 1 Participants recruited | 2 | 229 | Risk difference  IV, Random, 95% CI | 0.13 [0.01 , 0.26] |

#### Analysis 50.2 Participants retained

|  |  |  |  |  |
| --- | --- | --- | --- | --- |
| **Outcome or subgroup title** | **No. of studies** | **No. of participants** | **Statistical method** | **Effect size** |
| 2 Participants retained | 2 | 237 | Risk difference  IV, Random, 95% CI | 0.13 [0.01 , 0.26] |

### Comparison 51E–Educational material to provide additional information about a trial. (GRADE: unable to complete, no forest plot available)

|  |  |  |  |  |
| --- | --- | --- | --- | --- |
| **Outcome or subgroup title** | **No. of studies** | **No. of participants** | **Statistical method** | **Effect size** |
| 1 Participants recruited | 1 | 126 centres | N/A | N/A |

We were unable to generate a forest plot for this comparison as the data required to do so were not reported in the study (Kimmick 2005). The authors of this cluster trial did not present centre‐level recruitment data, or provide an intracluster correlation coefficient. We were also unable to complete a GRADE assessment for this comparison due to lack of data.

### Comparison 52E-Trained recruiters from a similar ethnic background to study population already taking part in a trial as lay advocates (high risk of bias, no forest plot available)

|  |  |  |  |  |
| --- | --- | --- | --- | --- |
| **Outcome or subgroup title** | **No. of studies** | **No. of participants** | **Statistical method** | **Effect size** |
| 1 Participants recruited | 1 | 68 | N/A | N/A |

We were unable to generate a forest plot for this comparison as the data required to do so were not reported in the study (Larkey 2002). The authors of this cluster trial did not report an analysis that corrected for the clustering or provide an intracluster correlation coefficient. We were also unable to complete a GRADE assessment for this comparison due to lack of data.

### Comparison 53E–Using a coach to promote trust among minority patients (high risk of bias, no forest plot available)

|  |  |  |  |  |
| --- | --- | --- | --- | --- |
| **Outcome or subgroup title** | **No. of studies** | **No. of participants** | **Statistical method** | **Effect size** |
| 1 Participants recruited | 1 | 75 | N/A | N/A |

We were unable to generate a forest plot for this comparison as the data required to do so were not reported in the study (Facasso 2013). The authors did not report the numbers of participants who were randomised to receive coaching versus usual care, so we were unable to determine the proportion of participants who went on to enrol in a trial in each study arm. We were also unable to complete a GRADE assessment for this comparison due to lack of data.

## F: Interventions aimed at the recruiter or recruitment site

**Note:** EDI data were not reported for studies in this category; interpret results with caution regarding generalisability to under-served groups.

### Comparison 54F–Teaser campaign using postcards vs no teaser (GRADE: moderate)

|  |  |  |  |  |
| --- | --- | --- | --- | --- |
| **Outcome or subgroup title** | **No. of studies** | **No. of participants** | **Statistical method** | **Effect size** |
| 1 Participants recruited | 1 | 670 | Risk difference  M-H, Fixed, 95% CI | 0.00 [-0.04 , 0.05] |

### Comparison 55F–Educational program to tailor recruitment strategies for minority recruitment vs usual recruitment (GRADE: low)

#### Analysis 55.1 Participants recruited

|  |  |  |  |  |
| --- | --- | --- | --- | --- |
| **Outcome or subgroup title** | **No. of studies** | **No. of participants** | **Statistical method** | **Effect size** |
| 1 Participants recruited | 1 | 2107 | Risk difference  M-H, Fixed, 95% CI | -0.00 [-0.04 , 0.03] |

### Comparison 56F–Remote vs face-to-face site initiation visits (GRADE: moderate)

#### Analysis 56.1 Participants recruited

|  |  |  |  |  |
| --- | --- | --- | --- | --- |
| **Outcome or subgroup title** | **No. of studies** | **No. of participants** | **Statistical method** | **Effect size** |
| 1 Participants recruited | 1 | 480 | Risk difference  M-H, Fixed, 95% CI | 0.06 [-0.01 , 0.13] |

#### Analysis 56.2 Participants retained

|  |  |  |  |  |
| --- | --- | --- | --- | --- |
| **Outcome or subgroup title** | **No. of studies** | **No. of participants** | **Statistical method** | **Effect size** |
| 1 Participants recruited | 1 | 378 | Risk difference  M-H, Fixed, 95% CI | -0.01 [-0.09 , 0.06] |

### Comparison 57F–Recruitment coordinator conducting teleconferences with site teams to enhance trial recruitment using software for identifying eligible patients, with a second teleconference after 6 months (low risk of bias, GRADE- unable to complete, no forest plot available)

|  |  |  |  |  |
| --- | --- | --- | --- | --- |
| **Outcome or subgroup title** | **No. of studies** | **No. of participants** | **Statistical method** | **Effect size** |
| 1 Participants recruited | 1 | 72 hospital sites | N/A | N/A |

We were unable to generate a forest plot for this comparison as the data required to do so were not reported in the study (Maxwell 2017). A stepped wedge approach was used to evaluate this intervention - raw figures at each step were not provided, so it was not possible to calculate a risk difference. We were also unable to complete a GRADE assessment for this comparison due to lack of data.

### Comparison 58F–Using teleconference and commitment contracts with site teams to enhance recruitment (Unclear risk of bias, GRADE- unable to complete, no forest plot available)

|  |  |  |  |  |
| --- | --- | --- | --- | --- |
| **Outcome or subgroup title** | **No. of studies** | **No. of participants** | **Statistical method** | **Effect size** |
| 1 Participants recruited | 1 | 20 tertiary care centres | N/A | N/A |

We were unable to generate a forest plot for this comparison as the data required to do so were not reported in the study (Isaksson 2021). A stepped wedge approach was used to evaluate this intervention - raw figures at each step were not provided, so it was not possible to calculate a risk difference. We were also unable to complete a GRADE assessment for this comparison due to lack of data.

### Comparison 59F–Onsite initiation visits versus no initiation visit (Unclear risk of bias, GRADE- unable to complete, no forest plot available)

|  |  |  |  |  |
| --- | --- | --- | --- | --- |
| **Outcome or subgroup title** | **No. of studies** | **No. of participants** | **Statistical method** | **Effect size** |
| 1 Participants recruited | 1 |  | N/A | N/A |

We were unable to generate a forest plot for this comparison as the data required to do so were not reported in the study (Liénard 2006). The authors did not present the proportion of eligible participants recruited, only the number recruited. We were also unable to complete a GRADE assessment for this comparison due to lack of data.

### Comparison 60F–Additional communication strategies such as tailored feedback on recruitment (low risk of bias, GRADE- unable to complete, no forest plot available)

|  |  |  |  |  |
| --- | --- | --- | --- | --- |
| **Outcome or subgroup title** | **No. of studies** | **No. of participants** | **Statistical method** | **Effect size** |
| 1 Participants recruited | 1 | 167 clinical sites | N/A | N/A |

We were unable to generate a forest plot for this comparison as the data required to do so were not reported in the study (Monaghan 2007). We were also unable to complete a GRADE assessment for this comparison due to lack of data.

### Comparison 61F–Recruiter training with ongoing support and/or theory-informed personalised nudge to the recruiter after each randomisation may increase recruitment (low risk of bias, GRADE- unable to complete, no forest plot available)

|  |  |  |  |  |
| --- | --- | --- | --- | --- |
| **Outcome or subgroup title** | **No. of studies** | **No. of participants** | **Statistical method** | **Effect size** |
| 1 Participants recruited | 1 | 1215 | N/A | N/A |

We were unable to generate a forest plot for this comparison as the data required to do so were not reported in the study (Agni 2022). The authors did not collect the number of eligible participants approached in the study, as such it was not possible to calculate a risk difference for this intervention. We were also unable to complete a GRADE assessment for this comparison due to lack of data.

### Comparison 62F–Automatically flagging eligible patients to clinicians via electronic health records vs. manually identifying patients via electronic health records (high risk of bias, GRADE- unable to complete, no forest plot available)

|  |  |  |  |  |
| --- | --- | --- | --- | --- |
| **Outcome or subgroup title** | **No. of studies** | **No. of participants** | **Statistical method** | **Effect size** |
| 1 Participants recruited | 1 | 36 practices | N/A | N/A |

We were unable to generate a forest plot for this comparison as the data required to do so were not reported in the study (Ethier 2017). The authors did not collect the number of eligible participants approached in the study, as such it was not possible to calculate a risk difference for this intervention. We were also unable to complete a GRADE assessment for this comparison due to lack of data.

## Category G: Incentives

**Note**: EDI data reporting was very limited for studies in this category; interpret results with caution regarding generalisability to under-served groups.

### Comparison 63G–Monetary incentive vs no incentive (GRADE: low)

#### Analysis 63.1 Participants recruited

|  |  |  |  |  |
| --- | --- | --- | --- | --- |
| **Outcome or subgroup title** | **No. of studies** | **No. of participants** | **Statistical method** | **Effect size** |
| 1 Participants recruited | 10 | 5082 | Risk difference  IV, Random, 95% CI | 0.04 [0.02 , 0.07] |

#### Analysis 63.2 Participants recruited (by allocation concealment)

|  |  |  |  |  |
| --- | --- | --- | --- | --- |
| **Outcome or subgroup title** | **No. of studies** | **No. of participants** | **Statistical method** | **Effect size** |
| 2 Participants recruited (by allocation concealment) | 10 | 5082 | Risk difference  IV, Random, 95% CI | 0.04 [0.02 , 0.07] |

### Comparison 64G–Monetary incentive: higher value vs lower value (GRADE: very low)

#### Analysis 52.1: participants recruited

|  |  |  |  |  |
| --- | --- | --- | --- | --- |
| **Outcome or subgroup title** | **No. of studies** | **No. of participants** | **Statistical method** | **Effect size** |
| 1 Participants recruited | 2 | 864 | Risk difference  IV, Random, 95% CI | 0.03 [-0.13 , 0.19] |

### Comparison 65G–Non-monetary incentive (branded pen vs no pen) (GRADE: Very low)

#### Analysis 65.1: participants recruited

|  |  |  |  |  |
| --- | --- | --- | --- | --- |
| **Outcome or subgroup title** | **No. of studies** | **No. of participants** | **Statistical method** | **Effect size** |
| 1 Participants recruited | 3 | 9640 | Risk difference  IV, Random, 95% CI | -0.00 [-0.01 , 0.01] |
