# Supplementary material for: Strategies to improve recruitment to randomised trials
Source: Cochrane Database Syst Rev. 2026 Sep 15;2026(9):MR000013. doi: 10.1002/14651858.MR000013.pub7 (PMC13576036; doi:10.1002/14651858.MR000013.pub7)
Supplement: Supplementary file 11 [file MR000013-SUP-11-other.html]

Equity, diversity and inclusion characteristics of included participants (PRO-EDI)


# Supplementary material 11 to: Strategies to improve recruitment to randomised trials

Parker A, Mongelli G, Piccolo-Lawrance C, Coleman E, Gkekas A, Wang HI, Dawson S, Green H, Way R, Rai A, Bruhn H, Sullivan F, Boxall C, Chan M, Manson P, Hewitt C, Fairhurst C, Clarke M, Treweek S
  
https://doi.org/10.1002/14651858.MR000013.pub7

The material in this section has been supplied by the author(s) for publication under a Licence for Publication and the author(s) are solely responsible for the material. Cochrane has reviewed this material, but Cochrane has not copyedited, formatted or proofread. Cochrane accordingly gives no representations or warranties of any kind in relation to, and accepts no liability for any reliance on or use of, such material.

Back to top

# Equity, diversity and inclusion characteristics of included participants (PRO-EDI)

## A: Design

### Comparison 1. A–Open-label trial vs blinded trial (GRADE: high)

An open-label design compared to a blinded, placebo-controlled design results in a large increase in recruitment: RD = 10% (95% CI 8% to 12%); GRADE: high.

**Equity information (see** Table 2**)**

This result and its GRADE assessment apply to women (although one study did involve some men) in the UK and Estonia. The GRADE assessment when applied to groups other than those mentioned will be below high because of indirectness.

This is based on three studies: Avenell 2004 (fracture prevention); RoB: low; Hemminki 2004 (postmenopausal hormone therapy); RoB: low; Veerus 2016 (post-menopausal hormone therapy); RoB: high.

|  |  |
| --- | --- |
| **PRO EDI Participant characteristics table for open-label trial vs blinded trials** | |
| **Characteristic** | **The people who took part** |
| Age | Only one study mentioned age and in that study the mean age was around 78 years. |
| Sex | No information provided by authors. |
| Gender | Almost all participants were women. Only one study included men, representing around 20% of participants. |
| Race, ethnicity and ancestry | No information provided by authors. |
| Socio-economic status (SES) | No information provided by authors. |
| Location (country/ countries of data collection and site coordination) | All studies were in high-income countries, either Estonia (2) or the UK (1). Whether the people involved were in urban and rural environments was not reported. |

### Comparison 2. A–Patient preference design vs conventional RCT (GRADE: low)

A patient preference design increased total participation but made little to no difference to recruitment to the randomised trial: RD = -4% (reduced recruitment) (95% CI -15% to 7%); GRADE: low (-2 levels: imprecision- single study; wide CI crossing RD=0).

**Equity information (see** Table 2**)**

This result and its GRADE assessment apply to mostly higher-income women in the UK (Scotland) but to who else is unclear as more participant characteristics were not provided by the authors. The GRADE assessment when applied to groups other than those mentioned will be below low because of indirectness.

This is based on one study: Cooper 1997 (management strategies for heavy menstrual bleeding) RoB: low.

|  |  |
| --- | --- |
| **PRO EDI Participant characteristics table for patient preference design vs conventional RCT** | |
| **Characteristic** | **The people who took part** |
| Age | Intervention age < 40 n=50, 37%.  Control age < 40 n=42, 30%. |
| Sex | No information provided by authors. |
| Gender | 100% women. |
| Race, ethnicity and ancestry | No information provided by authors. |
| Socio-economic status (SES) | Living in own property  Intervention n=88, 67%.  Control n=98, 72%.   Unemployed  Intervention n=24, 18%.  Control n=31, 23%. |
| Location (country/ countries of data collection and site coordination) | The single study was done in high-income country, the UK (Scotland). Whether the people involved were in urban and rural environments was not reported. |

### Comparison 3. A–Electronic data capture vs paper-based data capture (GRADE: low)

Internet-based, electronic data collection compared to paper-based may result in a large reduction in recruitment: RD = -13% (reduced recruitment) (95% CI -24% to -3%); GRADE: low (-1 level: study limitations-unclear RoB; -1 level: imprecision-single study).

**Equity information (see** Table 2**)**

This result and its GRADE assessment apply to people living in the UK but to whom exactly is unclear as more participant characteristics were not provided by the authors. The GRADE assessment when applied to specific groups will be below low because of indirectness.

This is based on one study: Litchfield 2005 (delivery systems for insulin) RoB: unclear.

|  |  |
| --- | --- |
| **PRO EDI Participant characteristics table for electronic data capture vs paper-based capture** | |
| **Characteristic** | **The people who took part** |
| Age | No information provided by authors |
| Sex | No information provided by authors |
| Gender | No information provided by authors |
| Race, ethnicity and ancestry | No information provided by authors |
| Socio-economic status (SES) | No information provided by authors |
| Location (country/ countries of data collection and site coordination) | The single study was done in high-income country, the UK. Whether the people involved were in urban and rural environments was not reported. |

### 4 A–Cluster‐randomised design compared to Zelen design (GRADE: unable to complete, no forest plot available)

Cluster‐randomised design compared to Zelen design. The study had only two sites (clusters) with few participants: 6 out of 24 potential participants were recruited in the cluster arm, compared to 0 out of 29 in the Zelen arm; RoB: low.

**Equity information (see** Table 2**)**

This result is based on one study involving mostly older people receiving end of life care in the UK (Wales).

This is based on one study: Fowell 2006 (palliative care) RoB: low. We were unable to complete a GRADE assessment for this comparison due to lack of data.

|  |  |
| --- | --- |
| **PRO EDI Participant characteristics table for electronic data capture vs paper-based capture** | |
| **Characteristic** | **The people who took part** |
| Age | No information provided by authors |
| Sex | No information provided by authors |
| Gender | No information provided by authors |
| Race, ethnicity and ancestry | No information provided by authors |
| Socio-economic status (SES) | No information provided by authors |
| Location (country/ countries of data collection and site coordination) | The single study was done in a high-income country, the UK. |

### Comparison 5 A-Two‐stage randomisation to choose duration of treatment (GRADE: unable to complete, no forest plot available)

Two‐stage randomisation to choose duration of treatment. Data on numbers recruited not available for one arm but up‐front randomisation to 3 or 6 months treatment gave a recruitment rate of 5.21 per year per centre compared to 4.09 for delayed randomisation to decide whether a second 3 month treatment was given. We were unable to complete a GRADE assessment for this comparison due to lack of data.

**Equity information (see** Table 2**)**

This result is based on one study involving people living in the UK.

This is based on one study: Paul 2011 (adjuvant treatment for colorectal cancer) RoB: low.

|  |  |
| --- | --- |
| **PRO EDI Participant characteristics table for electronic data capture vs paper-based capture** | |
| **Characteristic** | **The people who took part** |
| Age | ‘Persons more likely to consent to the trial were under 70 years and born in Australia’. |
| Sex | No information provided by authors |
| Gender | No information provided by authors |
| Race, ethnicity and ancestry | No information provided by authors |
| Socio-economic status (SES) | No information provided by authors |
| Location (country/ countries of data collection and site coordination) | The single study was done in a high-income country, the UK. |

## B: Pre-trial planning

### Comparison 6 B–Pre-engagement with potential trial sites during proposal development vs no pre-engagement (GRADE: low)

Internet-based, electronic data collection compared to paper-based may reduce recruitment: RD = 50% (reduced recruitment) (95% CI 18% to 81%); GRADE: low (-1 level: study limitations-unclear RoB; -1 level: imprecision-single study).

**Equity information (see** Table 2**)**

This result and its GRADE assessment apply to Health and Social Services Centres Directors working in Canada but to who else is unclear as more participant characteristics were not provided by the authors. The GRADE assessment when applied to groups other than those mentioned will be below low because of indirectness.

This is based on one study: Garvelink 2015 (Shared decision making in frail elderly patients) RoB: unclear.

|  |  |
| --- | --- |
| **PRO EDI Participant characteristics table for pre-engagement with potential trial sites during proposal development vs no pre-engagement** | |
| **Characteristic** | **The people who took part** |
| Age | No information provided by authors. |
| Sex | No information provided by authors. |
| Gender | No information provided by authors. |
| Race, ethnicity and ancestry | No information provided by authors. |
| Socio-economic status (SES) | No information provided by authors. |
| Location (country/ countries of data collection and site coordination) | The single study was done in a high-income country, Canada. Whether the people involved were in urban and rural environments was not reported. |

## C: Trial conduct changes

### Comparison 7 C–Telephone reminder vs no telephone reminder (GRADE: high)

Using a telephone reminder to contact non-responders to a postal invitation increases recruitment. RD = 6% (95% CI 3% to 9%); GRADE: high.

**Equity information (see** Table 2**)**

This result and its GRADE assessment apply to middle-aged people living in Canada and Norway but to whom exactly is unclear as more participant characteristics were not provided by the authors. The GRADE assessment when applied to specific groups will be below high because of indirectness.

This is based on two studies: Nystuen 2004 (getting people to return to work); RoB: low; Wong 2013 (colorectal cancer) RoB: low.

**NOTE**: the evidence for this intervention comes entirely from trials with low (<10%) underlying recruitment. When applied to trials with higher recruitment we would downgrade the GRADE assessment because of Indirectness to moderate.

|  |  |
| --- | --- |
| **PRO EDI Participant characteristics table for telephone reminder vs no telephone reminder** | |
| **Characteristic** | **The people who took part** |
| Age | Participants had a mean age of around 56 in one study; the other study did not provide age information. |
| Sex | No information provided by authors. |
| Gender | No information provided by authors. |
| Race, ethnicity and ancestry | No information provided by authors. |
| Socio-economic status (SES) | No information provided by authors. |
| Location (country/ countries of data collection and site coordination) | Both studies were done in high-income countries, Canada and Norway. Whether the people involved were in urban and rural environments was not reported. |

### Comparison 8 C–SMS reminder mentioning scarcity vs SMS reminder with no mention (GRADE: moderate)

Mentioning scarcity of trial places in SMS messages compared to not mentioning scarcity probably increases recruitment slightly; RD = 3% (95% CI = 1% to 6%); GRADE: moderate (-1 level: imprecision-single study). This is based on one study: Free 2011 (smoking cessation) RoB: low.

**Equity information (see** Table 2**)**

This result and its GRADE assessment apply to younger people living in the UK but to whom exactly is unclear as more participant characteristics were not provided by the authors. The GRADE assessment when applied to specific groups will be below moderate because of indirectness.

|  |  |
| --- | --- |
| **PRO EDI Participant characteristics table for SMS reminder mentioning scarcity vs SMS reminder with no mention** | |
| **Characteristic** | **The people who took part** |
| Age | The mean age was 36 years. |
| Sex | No information provided by authors. |
| Gender | No information provided by authors. |
| Race, ethnicity and ancestry | No information provided by authors. |
| Socio-economic status (SES) | No information provided by authors. |
| Location (country/ countries of data collection and site coordination) | The study was done in a high-income country, the UK. Whether the people involved were in urban and rural environments was not reported. |

### Comparison 9 C–SMS messages containing quotes from existing participants vs no messages (GRADE: moderate)

Giving quotes from previous participants in SMS messages compared to giving no quotes probably increases recruitment slightly. RD = 4% (95% CI = 2% to 6%); GRADE: moderate (-1 level: imprecision-single study).

**Equity information (see** Table 2)

This result and its GRADE assessment apply to younger people living in the UK but to whom exactly is unclear as more participant characteristics were not provided by the authors. The GRADE assessment when applied to specific groups will be below moderate because of indirectness.

This is based on one study: Free 2010 (smoking cessation) RoB: low.

|  |  |
| --- | --- |
| **PRO EDI Participant characteristics table for SMS messages containing quotes from existing participants vs no messages** | |
| **Characteristic** | **The people who took part** |
| Age | Mean age was around 34 years. |
| Sex | No information provided by authors. |
| Gender | No information provided by authors. |
| Race, ethnicity and ancestry | No information provided by authors. |
| Socio-economic status (SES) | No information provided by authors. |
| Location (country/ countries of data collection and site coordination) | The study was done in a high-income country, the UK. Whether the people involved were in urban and rural environments was not reported. |

### Comparison 10 C–Email invitation vs postal invitation (GRADE: moderate)

Using email invitations probably results in little to no difference in recruitment compared to postal invitations. RD = 1% (95% CI = -3% to 4%); GRADE: moderate (-1 level: imprecision-single study).

**Equity information (see** Table 2)

This result and its GRADE assessment apply to General Practitioners living in the UK (Scotland) but to whom exactly is unclear as more participant characteristics were not provided by the authors. The GRADE assessment when applied to specific groups will be below moderate because of indirectness.

This is based on one study: Treweek 2012 (antibiotic prescribing by GPs) RoB: low.

|  |  |
| --- | --- |
| **PRO EDI Participant characteristics table for mail invitation vs postal invitation** | |
| **Characteristic** | **The people who took part** |
| Age | No information provided by authors. |
| Sex | No information provided by authors. |
| Gender | Intervention 79, 57.2% men  Control 64, 48.5% men |
| Race, ethnicity and ancestry | No information provided by authors. |
| Socio-economic status (SES) | No information provided by authors. |
| Location (country/ countries of data collection and site coordination) | The study was done in a high-income country, the UK (Scotland). Whether the people involved were in urban and rural environments was not reported. |

### Comparison 11 C–Telephone reminders vs SMS reminders (GRADE: moderate)

Using telephone reminders probably increases recruitment compared to SMS reminders. RD = 5% (95% CI = -1% to 11%); GRADE: moderate (-1 level: imprecision-single study).

**Equity information (see** Table 2)

This result and its GRADE assessment apply to middle-aged men living in Australia but to whom exactly is unclear as more participant characteristics were not provided by the authors. The GRADE assessment when applied to specific groups will be below moderate because of indirectness.

This is based on one study: Bracken 2019 (diabetes prevention) RoB: low.

|  |  |
| --- | --- |
| **PRO EDI Participant characteristics table for telephone reminders vs SMS reminders** | |
| **Characteristic** | **The people who took part** |
| Age | The mean age was around 58 years. |
| Sex | No information provided by authors. |
| Gender | 100% of participants were men. |
| Race, ethnicity and ancestry | No information provided by authors. |
| Socio-economic status (SES) | No information provided by authors. |
| Location (country/ countries of data collection and site coordination) | The study was done in a high-income country, Australia. Whether the people involved were in urban and rural environments was not reported. |

## D: Modification to the consent process

### Comparison 12 D–Opt-out consent vs opt-in consent (GRADE: low)

Opt-out consent may improve recruitment. RD = 19% (95% CI = 3% to 35%); GRADE: low (-1 level: study limitations-unclear RoB; -1 level: imprecision-single study).

**Equity information (see** Table 2)

This result and its GRADE assessment apply to mostly better-off, male and females above the age of 50 living in Australia. The GRADE assessment when applied to groups other than those mentioned will be below low because of indirectness.

This is based on one study: Trevena 2006 (colorectal cancer) RoB: unclear.

|  |  |
| --- | --- |
| **PRO EDI Participant characteristics table for opt-out consent vs opt-in consent** | |
| **Characteristic** | **The people who took part** |
| Age | Intervention Age distribution in years  50-54 – 20, 33.3%  55-64 – 26, 43.3%  65-74 – 14, 23.3%   Control Age distribution in years  50-54 – 30, 32.6%  55-64 – 40, 43.5%  65-74 – 22, 23.7% |
| Sex | Intervention 24, 40% male  Control 29, 31.5% male |
| Gender | No information provided by authors. |
| Race, ethnicity and ancestry | No information provided by authors. |
| Socio-economic status (SES) | SEIFA score below 75% quantile (lower socioeconomic status)  Intervention 8 (13.3%)  Control 12 (13.0%)   [SEIFA, Socioeconomic Index for Area (score of relative socioeconomic disadvantage). Provided by the Australian Bureau of Statistics]. |
| Location (country/ countries of data collection and site coordination) | Australia |

### Comparison 13 D–Researcher reading out consent vs participant reading consent (GRADE: very low)

The evidence is very uncertain about the effect of a researcher reading out the consent details on recruitment. RD = 6% (95% CI = -13% to 25%); GRADE: very low (-1 level: study limitations-unclear RoB; -2 levels: imprecision-single study; wide CI crossing RD= 0).

**Equity information (see** Table 2)

This result and its GRADE assessment apply to people living in the USA but to whom exactly is unclear as more participant characteristics were not provided by the authors. The GRADE assessment would be lower for other groups because of indirectness were it not already at very low.

This is based on one study: Wadland 1990 (smoking cessation) RoB: unclear.

|  |  |
| --- | --- |
| **PRO EDI Participant characteristics table for researcher reading out consent vs participant reading consent** | |
| **Characteristic** | **The people who took part** |
| Age | No information provided by authors. |
| Sex | No information provided by authors. |
| Gender | 100% of participants were men. |
| Race, ethnicity and ancestry | No information provided by authors. |
| Socio-economic status (SES) | No information provided by authors. |
| Location (country/ countries of data collection and site coordination) | The study was done in a high-income country, the USA. Whether the people involved were in urban and rural environments was not reported. |

### Comparison 14 D-Easy to read consent form (GRADE: unable to complete, no forest plot available)

The evidence is very uncertain about the effect of an easy to read consent form (RD=3; P = 0.32); GRADE: We were unable to complete a GRADE assessment for this comparison due to lack of data

**Equity information (see** Table 2)

This result and its GRADE assessment applies mostly to well-educated white women above the age of 50 years living in the USA. The GRADE assessment would be lower for other groups because of indirectness were it not already at very low.

This is based on one study: Coyne 2003 (cancer) RoB: unclear.

|  |  |
| --- | --- |
| **PRO EDI Participant characteristics table for easy to read consent vs standard consent** | |
| **Characteristic** | **The people who took part** |
| Age | Of the participants for whom demographic characteristics were collected (92%, 207/226), the mean age was 53 years. |
| Sex | No information provided by authors. |
| Gender | Of the participants for whom demographic characteristics were collected (92%, 207/226), 90% were women. |
| Race, ethnicity and ancestry | Of those for whom demographic characteristics were collected (92%, 207/226), 93% were white. |
| Socio-economic status (SES) | Education:  Of the participants for whom demographic characteristics were collected (92%, 207/226), the majority (52%) of patients had at least some college education, with more than half of these patients having earned college degrees. |
| Location (country/ countries of data collection and site coordination) | The study was done in a high-income country, the USA. Whether the people involved were in urban and rural environments was not reported. |

## Comparison 15 D–Information printed on heavyweight paper and blue folio vs standard (high risk of bias)

This is a single study, with high risk of bias. We have not presented the results.

### Comparison 16 D–Enhanced pre-consent discussion with aid of poster vs. standard consent (high risk of bias)

This is a single study, with high risk of bias. We have not presented the results.

## E: Modification to the information given to potential participants about the trial

### Comparison 17 E–Brief participant information leaflet (PIL) vs full PIL (GRADE: moderate)

Using a brief patient information leaflet (PIL) probably results in little to no difference in recruitment compared to a full PIL. RD = 0% (95% CI = -1% to 2%); GRADE: moderate (-1 level: indirectness, Chen 2011 actually measures entry to pre-randomisation phase).

**Equity information (see** Table 2)

This result and its GRADE assessment apply to people, especially women, around 40 years old living in the UK. The GRADE assessment when applied to groups other than those mentioned will be below moderate because of indirectness.

This is based on two studies: Chen 2011 RoB: low; Brierley 2012 (depression) RoB: low.

|  |  |
| --- | --- |
| **PRO EDI Participant characteristics table for brief participant information leaflet (PIL) vs full PIL** | |
| **Characteristic** | **The people who took part** |
| Age | Only one study mentioned age, and this was for those recruited to the host trial, not all those sent the PIL. Mean age was around 43 years. |
| Sex | Only one study mentioned age, and this was for those recruited to the host trial, not all those sent the PIL. About 75% of participants were female, 25% male. |
| Gender | No information provided by authors. |
| Race, ethnicity and ancestry | No information provided by authors. |
| Socio-economic status (SES) | No information provided by authors. |
| Location (country/ countries of data collection and site coordination) | Both studies were done in a high-income country, the UK. Whether the people involved were in urban and rural environments was not reported. |

### Comparison 18 E–Study-related questionnaire + trial invitation vs trial invitation (GRADE: moderate)

Enclosing a questionnaire covering issues relevant to trial with the invitation probably increases recruitment. RD = 5% (95% CI = 2% to 8%); GRADE: moderate (-1 level: imprecision-single study).

**Equity information (see** Table 2)

This result and its GRADE assessment apply to people living in the UK but to whom exactly is unclear as more participant characteristics were not provided by the authors. The GRADE assessment when applied to specific groups will be below moderate because of indirectness.

This is based on one study: Kendrick 2001 (injury prevention, recruiting family units) RoB: low.

|  |  |
| --- | --- |
| **PRO EDI Participant characteristics table for study-related questionnaire + trial invitation vs trial invitation** | |
| **Characteristic** | **The people who took part** |
| Age | No information provided by authors. |
| Sex | No information provided by authors. |
| Gender | No information provided by authors. |
| Race, ethnicity and ancestry | No information provided by authors. |
| Socio-economic status (SES) | No information provided by authors. |
| Location (country/ countries of data collection and site coordination) | The study was done in a high-income country, the UK. Whether the people involved were in urban and rural environments was not reported. |

### Comparison 19 E–PIL developed with feedback from users vs usual PIL (GRADE: moderate)

Optimising the PIL through using user feedback probably results in little to no difference in recruitment. RD = 0% (95% CI = 0% to 1%); GRADE: moderate (-1 level: indirectness, Chen 2011 actually measures entry to pre-randomisation phase).

**Equity information (see** Table 2)

This result and its GRADE assessment apply to older people living in the UK but to whom exactly is unclear as more participant characteristics were not provided by the authors. The GRADE assessment when applied to specific groups will be below moderate because of indirectness.

This is based on two studies: Chen 2011 (unclear) RoB: low; Cockayne 2017 (START PIS REFORM) (falls prevention) RoB: low.

|  |  |
| --- | --- |
| **PRO EDI Participant characteristics table for PIL developed with feedback from users vs usual PIL** | |
| **Characteristic** | **The people who took part** |
| Age | One study Cockayne 2017 (START PIS REFORM) was done in people with a mean age around 78. The second study said nothing about age. |
| Sex | One study Cockayne 2017 (START PIS REFORM) was around 45% male. The second study said nothing about sex. |
| Gender | No information provided by authors. |
| Race, ethnicity and ancestry | No information provided by authors. |
| Socio-economic status (SES) | No information provided by authors. |
| Location (country/ countries of data collection and site coordination) | Both studies were done in a high-income country, the UK. Whether the people involved were in urban and rural environments was not reported. |

### Comparison 20 E–Enhanced recruitment package + recruitment at churches vs standard recruitment package (GRADE: low)

Recruitment at a church and other enhancements may result in little to no difference in recruitment. RD = 1% (95% CI = 0% to 2%); GRADE: low (-1 level: study limitations-unclear RoB; -1 level: imprecision-single study).

**Equity information (see** Table 2)

This result and its GRADE assessment apply to African American men living in the USA but to who else is unclear as more participant characteristics were not provided by the authors. The GRADE assessment when applied to groups other than those mentioned will be below low because of indirectness.

This is based on one study: Ford 2004 (cancer) RoB: unclear.

|  |  |
| --- | --- |
| **PRO EDI Participant characteristics table for enhanced recruitment package + recruitment at churches vs standard recruitment package** | |
| **Characteristic** | **The people who took part** |
| Age | No information provided by authors. |
| Sex | No information provided by authors. |
| Gender | 100% men |
| Race, ethnicity and ancestry | 100% African American |
| Socio-economic status (SES) | No information provided by authors. |
| Location (country/ countries of data collection and site coordination) | USA. Whether the people involved were in urban and rural environments was not reported. |

### Comparison 21 E–Enhanced recruitment package vs standard recruitment package (GRADE: low)

An enhanced recruitment package including more contact may result in little to no difference in recruitment. RD = 0% (95% CI = -1% to 0%); GRADE: low (-1 level: study limitations- unclear RoB; -1 level: imprecision-single study).

**Equity information (see** Table 2)

This result and its GRADE assessment apply to African American men living in the USA but to who else is unclear as more participant characteristics were not provided by the authors. The GRADE assessment when applied to groups other than those mentioned will be below low because of indirectness.

This is based on one study: Ford 2004 (cancer) RoB: unclear.

|  |  |
| --- | --- |
| **PRO EDI Participant characteristics table for enhanced recruitment package vs standard recruitment package** | |
| **Characteristic** | **The people who took part** |
| Age | No information provided by authors. |
| Sex | No information provided by authors. |
| Gender | 100% men |
| Race, ethnicity and ancestry | 100% African American |
| Socio-economic status (SES) | No information provided by authors. |
| Location (country/ countries of data collection and site coordination) | USA. Whether the people involved were in urban and rural environments was not reported. |

### Comparison 22 E–Enhanced recruitment package + baseline data over telephone vs standard recruitment package (GRADE: low)

An enhanced recruitment package including more contact by telephone may result in little to no difference in recruitment. RD = 0% (95% CI = -1% to 1%); GRADE: low (-1 level: study limitations-unclear RoB; -1 level: imprecision-single study).

**Equity information (see** Table 2)

This result and its GRADE assessment apply to African American men living in the USA but to who else is unclear as more participant characteristics were not provided by the authors. The GRADE assessment when applied to groups other than those mentioned will be below low because of indirectness.

This is based on one study: Ford 2004 (cancer) RoB: unclear.

[This table is presented in the collection of PRO EDI participant characteristics tables, a supplementary file, not the Results section.]

|  |  |
| --- | --- |
| **PRO EDI Participant characteristics table for enhanced recruitment package + baseline data over telephone vs standard recruitment package** | |
| **Characteristic** | **The people who took part** |
| Age | No information provided by authors. |
| Sex | No information provided by authors. |
| Gender | 100% men |
| Race, ethnicity and ancestry | 100% African American |
| Socio-economic status (SES) | No information provided by authors. |
| Location (country/ countries of data collection and site coordination) | USA. Whether the people involved were in urban and rural environments was not reported. |

### Comparison 23 E–Audio record of information given about trial vs no audio record (GRADE: very low)

The evidence is very uncertain about the effect of providing an audio record of the discussion about the trial on recruitment. RD = -3% (reduced recruitment) (95% CI = -19% to 13%); GRADE: very low (-1 level: study limitations-unclear RoB; -2 levels: imprecision-single study; wide CI crossing RD=0).

**Equity information (see** Table 2)

This result and its GRADE assessment apply to middle-aged, mostly female with a broad range of educational backgrounds living in Sweden. The GRADE assessment would be lower for other groups because of indirectness were it not already at very low.

This is based on one study: Bergenmar 2014 (cancer) RoB: unclear.

|  |  |
| --- | --- |
| **PRO EDI Participant characteristics table for audio record of information given about trial vs no audio record** | |
| **Characteristic** | **The people who took part** |
| Age | Intervention mean 55 years, SD 10.3  Control mean 54 years SD 10.8   Intervention  <45 years 10, 15%  45-64 years 47, 70%  ≥65 years 10, 15%   Control  <45 years 11, 18%  45-64 years 41, 65%  ≥65 years 11, 18% |
| Sex | Intervention 13, 19% male; 54, 81% female  Control 10, 16% male; 53, 84% female   Gender/sex mix in paper (sex listed), corrected here |
| Gender | No information provided by authors. |
| Race, ethnicity and ancestry | No information provided by authors. |
| Socio-economic status (SES) | Education:  Intervention  Compulsory school (1 – 9 years) 9, 13%  Senior high school (10 – 12 years) 8, 12%  University education (13 – 16 years) 5, 8%  Higher university education ( > 16 years) 26, 39%  Data missing 19, 28%   Control  Compulsory school (1 – 9 years) 9, 14%  Senior high school (10 – 12 years) 12, 19%  University education (13 – 16 years) 9, 14%  Higher university education ( > 16 years) 22, 35%  Data missing 11, 18% |
| Location (country/ countries of data collection and site coordination) | Sweden. Whether the people involved were in urban and rural environments was not reported. |

### Comparison 24 E–Clinical trial booklet + standard information vs standard information (GRADE: very low)

The evidence is very uncertain about the effect of providing a clinical trial booklet together with standard information on recruitment. RD = 20% (95% CI = -5% to 46%); GRADE: very low (-1 level: study limitations-unclear RoB; -2 levels: imprecision-single study; wide CI crossing RD=0).

**Equity information (see** Table 2)

This result and its GRADE assessment apply to well educated males in their 30s living in an urban population in the UK. The GRADE assessment would be lower for other groups because of indirectness were it not already at very low.

This is based on one study: Ives 2001 (HIV) RoB: unclear.

|  |  |
| --- | --- |
| **PRO EDI Participant characteristics table for clinical trial booklet + standard information vs standard information** | |
| **Characteristic** | **The people who took part** |
| Age | Median age of 35 years [interquartile range (IQR) 31–42], |
| Sex | Male (94%) |
| Gender | No information provided by authors. |
| Race, ethnicity and ancestry | No information provided by authors |
| Socio-economic status (SES) | Educated up to and beyond A level: control group - 19 (73%); intervention group - 15 (65%) |
| Location (country/ countries of data collection and site coordination) | UK, urban population (London). |

### Comparison 25 E–Total information disclosure vs standard disclosure (GRADE: very low)

The evidence is very uncertain about the effect of providing total information disclosure rather than leaving it to recruiters as to what to reveal on recruitment. RD = 11% (95% CI = -6% to 28%); GRADE: very low (-1 level: study limitations-unclear RoB; -2 levels: imprecision-single study; wide CI crossing RD=0).

**Equity information (see** Table 2)

This result and its GRADE assessment apply to middle-aged, white women whose first language was English and who had high-school or lower education and are living in Australia. The GRADE assessment would be lower for other groups because of indirectness were it not already at very low.

This is based on one study: Simes 1986 (cancer) RoB: unclear.

|  |  |
| --- | --- |
| **PRO EDI Participant characteristics table for total information disclosure vs standard disclosure** | |
| **Characteristic** | **The people who took part** |
| Age | Intervention A median age 55 years, range 40-74  Intervention B median age 56 years, range 31-68 |
| Sex | No information provided by authors. |
| Gender | Intervention A 18 women (of n=29)  Intervention B 23 women (of n=28) |
| Race, ethnicity and ancestry | Intervention A 29 white patients  Intervention B 27 white patients   English as first language  Intervention A 27 (of n=29)  Intervention B 26 (of n=28) |
| Socio-economic status (SES) | Educational standard high school or below  Intervention A 21 (of n=29)  Intervention B 20 (of n=28) |
| Location (country/ countries of data collection and site coordination) | Australia. Whether the people involved were in urban and rural environments was not reported. |

### Comparison 26 E–Access to cancer trials website vs no access (high risk of bias)

This is a single study, with high risk of bias. We have not presented the results.

### Comparison 27 E–Bespoke, user-tested PIL vs usual PIL (GRADE: high)

Optimising the participant information leaflet (PIL) through a particular, bespoke process involving formal user- testing makes little to no difference to recruitment. RD = 0% (95% CI = 0% to 1%); GRADE: high.

**Equity information (see** Table 2)

This result and its GRADE is based on studies involving people living in the UK. The GRADE assessment would be lower for other groups. Only one out of the six studies reported EDI data, and participants were mostly older women.

This is based on six studies: Cockayne 2017 (START PIS REFORM) (falls prevention) RoB: low; Knapp 2020 (START PIS CASPER) (behavioural activation for depression) RoB: low; Madurasinghe 2021 (START PIS HeLP Diabetes)(type 2 diabetes self management) RoB: low; Man 2015 (extra support for depression) RoB: low; Parker 2018 (lung cancer diagnosis) RoB: unclear; Sheridan 2020 (START PIS ISDR) (diabetic retinopathy screening) RoB: high.

|  |  |
| --- | --- |
| **PRO EDI Participant characteristics table for bespoke, user-tested PIL vs usual PIL** | |
| **Characteristic** | **The people who took part** |
| Age | Only one study reported EDI data (Cockayne 2017, START PIS REFORM), and reported a mean age around 78. |
| Sex | One study Cockayne 2017 (START PIS REFORM) was around 45% male. |
| Gender | No information provided by authors. |
| Race, ethnicity and ancestry | No information provided by authors. |
| Socio-economic status (SES) | No information provided by authors. |
| Location (country/ countries of data collection and site coordination) | All studies were done in a high-income country, the UK. Whether the people involved were in urban and rural environments was not reported. |

### Comparison 28. E–Recruitment primer letter/leaflet vs no letter/leaflet (GRADE: High)

Sending a recruitment primer letter results in little to no difference in recruitment. RD = 0% (95% CI = -1% to 2%); GRADE: high.

**Equity information (see** Table 2 **)**

This result and its GRADE assessment apply to mostly older white people living in the UK and Ireland but to who else is unclear as more participant characteristics were not provided by the authors. The GRADE assessment when applied to specific groups will be below high because of indirectness.

This is based on two studies: Paul 2014 (colorectal cancer) RoB: low; Arundel 2017 (falls prevention) RoB:low.

|  |  |
| --- | --- |
| **PRO EDI Participant characteristics table for recruitment primer letter/leaflet vs no letter/leaflet** | |
| **Characteristic** | **The people who took part** |
| Age | One study (Arundel 2017) involved people with a mean age of around 77. The second study said nothing about age. |
| Sex | One study (Arundel 2017) involved people who were around 43% male and 57% female. The second study said nothing about sex. |
| Gender | No information provided by authors. |
| Race, ethnicity and ancestry | One study (Arundel 2017) involved people who were >99% white. The second study said nothing about ethnicity. |
| Socio-economic status (SES) | No information provided by authors. |
| Location (country/ countries of data collection and site coordination) | UK, Ireland and Australia. Whether the people involved were in urban and rural environments was not reported. |

### Comparison 29. E–Information provided remotely vs information provided face-to-face (GRADE: Moderate)

Providing information over the telephone rather than face-to-face probably reduces recruitment. RD = -9% (reduced recruitment) (95% CI = ‐18% to 1%); GRADE: moderate.

**Equity information (see** Table 2**)**

This result and its GRADE assessment apply to people between the ages of around 30 and 60, especially women, who have a high degree of education and are living in the USA or Denmark. The GRADE assessment when applied to groups other than those mentioned will be below high because of indirectness.

This is based on two studies: Bobb 2016 (pneumonia) RoB: low; Foss 2016 (vaccination) RoB: low.

|  |  |
| --- | --- |
| **PRO EDI Participant characteristics table for information provided remotely vs information provided face-to-face** | |
| **Characteristic** | **The people who took part** |
| Age | Most participants were between 30 and 60. |
| Sex | One study (Foss 2016) reported sex and the population was evenly split between male and female. |
| Gender | One study (Bobb 2016) reported gender and the population was 100% women. |
| Race, ethnicity and ancestry | No information provided by authors. |
| Socio-economic status (SES) | One study (Bobb 2016) provided information about education and around 55% of participants had a masters degree or higher. Around 11% had basic schooling only. The second study said nothing about socioeconomic status. |
| Location (country/ countries of data collection and site coordination) | Denmark, USA. Whether the people involved were in urban and rural environments was not reported. |

### 30 E–Providing information by video vs standard information (GRADE: very low)

The evidence is very uncertain about the effect of providing trial information by video on recruitment; RD = -4% (95% CI = ‐23% to 15%); GRADE: very low (‐1 level: study limitations–unclear & high RoB; ‐1 level: inconsistency; ‐1 level: imprecision–wide CI crossing RD=0).

**Equity information (see** Table 2)

This result and its GRADE assessment apply to older, mainly ethnic majority people living in high-income countries. The GRADE assessment would be lower for other groups because of indirectness were it not already at very low.

This is based on five studies: Du 2008 (lung cancer) RoB: unclear; Du 2009 (breast cancer) RoB: unclear; Hutchison 2007 (cancer) RoB: low; Mattock 2020 (paediatric behavioural difficulties) RoB: high; Rodgers 2019 (arthritis) RoB: high.

|  |  |
| --- | --- |
| **PRO EDI Participant characteristics table for video vs standard information** | |
| **Characteristic** | **The people who took part** |
| Age | Most of the included studies involved people who were older, with mean ages well over 50 for three studies and over 70 for one, although one study involved younger people with a mean age of 33.9. |
| Sex | Overall, the mix of male and female participants was good, ranging between 20% and 100% female depending on the study. |
| Gender | No information provided by authors. |
| Race, ethnicity and ancestry | Three studies reported ethnicity information (two reported race and one reported ethnicity). Two studies had good inclusion of African Americans (roughly 30% to 40%) in addition to white people. One study included 6% Asian, 8% Black/African/Caribbean, and 6% other ethnic groups in addition to white people.    No other ethnic groups were named. |
| Socio-economic status (SES) | People from the full range of socioeconomic status were included, with around 20% to 50% from the most disadvantaged communities. |
| Location (country/ countries of data collection and site coordination) | All studies were done in high-income countries, either the USA (2) or the UK (3). Whether the people involved were in urban and rural environments was not reported. |

### Comparison 31 E–Invitation letter emphasising ethnicity-specific disease risk vs standard letter (GRADE: low)

An invitation letter that emphasises ethnicity-specific disease risks may reduce recruitment slightly. RD = -3% (reduced recruitment) (95% CI = -12% to 6%); GRADE: low (-2 levels: imprecision-single study; wide CI crossing RD=0).

**Equity information (see** Table 2)

This result and its GRADE assessment apply to women over the age of 30 from a range of ethnic groups and who live in the USA. The GRADE assessment when applied to groups other than those mentioned will be below low because of indirectness.

This is based on one study: Brown 2015 (pregnancy) RoB: low.

|  |  |
| --- | --- |
| **PRO EDI Participant characteristics table for invitation letter emphasising ethnicity-specific disease risk vs standard letter** | |
| **Characteristic** | **The people who took part** |
| Age | Over 75% or participants were 30 or over. |
| Sex | No information provided by authors. |
| Gender | 100% women |
| Race, ethnicity and ancestry | Around 55% of participants were Asian or Pacific Islanders. Latina and white people were both around 20% and African Americans around 4% of the population. |
| Socio-economic status (SES) | The study was done in the Kaiser Permanente Northern California integrated healthcare delivery system, which is broadly representative of the region’s ethnic and socioeconomic diversity |
| Location (country/ countries of data collection and site coordination) | USA. Whether the people involved were in urban and rural environments was not reported. |

### Comparison 32 E–Handwritten personalised invitation letter vs standard letter (GRADE: low)

A handwritten, personalised invitation letter may reduce recruitment slightly compared to a standard invitation letter. RD = -4% (reduced recruitment) (95% CI = -8% to 0%); GRADE: low (-1 level: study limitations-unclear RoB; -1 level: imprecision-single study).

**Equity information (see** Table 2)

This result and its GRADE assessment apply to people living in the UK but to whom exactly is unclear as more participant characteristics were not provided by the authors. The GRADE assessment when applied to specific groups will be below low because of indirectness.

This is based on one study: McCaffery 2019 (falls reduction) RoB: unclear.

|  |  |
| --- | --- |
| **PRO EDI Participant characteristics table for handwritten personalised invitation letter vs standard letter** | |
| **Characteristic** | **The people who took part** |
| Age | No information provided by authors. |
| Sex | No information provided by authors. |
| Gender | No information provided by authors. |
| Race, ethnicity and ancestry | No information provided by authors. |
| Socio-economic status (SES) | No information provided by authors. |
| Location (country/ countries of data collection and site coordination) | UK. Whether the people involved were in urban and rural environments was not reported. |

### Comparison 33 E–Different website wording, including infographic, about the risk of underlying condition (GRADE: Very low)

Different website wording, including infographic, about the risk of the underlying condition makes little or no difference to recruitment, compared to standard wording. RD=0% (95% CI = -0.01 to 0.01); GRADE: very low (-2 levels: study limitations-both included studies are high RoB; -1 level: indirectness - ‘Visitors’ to the website may not be unique).

**Equity information (see** Table 2)

This result and its GRADE assessment are based on a study involving older people aged 70 years and over living in the USA. The GRADE assessment would be lower for other specific groups because of indirectness were it not already at very low.

These findings are based on two studies (2605 participants): Miller 2021a (falls prevention); RoB: high and Miller 2021b (falls prevention); RoB: high.

|  |  |
| --- | --- |
| **PRO EDI Participant characteristics table for standard recruitment package vs shorter recruitment pack** | |
| **Characteristic** | **The people who took part** |
| Age | Older people aged 70 years and older |
| Sex | No information provided by authors. |
| Gender | No information provided by authors. |
| Race, ethnicity and ancestry | No information provided by authors. |
| Socio-economic status (SES) | No information provided by authors. |
| Location (country/ countries of data collection and site coordination) | USA. Whether the people involved were in urban and rural environments was not reported. |

### Comparison 34 E– Shorter recruitment package vs. Standard recruitment package (GRADE: very low)

It is very uncertain whether a shorter recruitment pack with standard consent affects recruitment. RD = 2% (95% CI = -13% to 17%); GRADE: very low (-1 level: study limitations-unclear RoB; -2 levels: imprecision-single study; wide CI crossing RD=0).

**Equity information (see** Table 2)

This result and its GRADE assessment apply to people living in the USA but to whom exactly is unclear as more participant characteristics were not provided by the authors. The GRADE assessment would be lower for other groups because of indirectness were it not already at very low.

This is based on one study: Chow 2020 (cancer) RoB: unclear.

|  |  |
| --- | --- |
| **PRO EDI Participant characteristics table for standard recruitment package vs shorter recruitment pack** | |
| **Characteristic** | **The people who took part** |
| Age | No information provided by authors. |
| Sex | No information provided by authors. |
| Gender | No information provided by authors. |
| Race, ethnicity and ancestry | No information provided by authors. |
| Socio-economic status (SES) | No information provided by authors. |
| Location (country/ countries of data collection and site coordination) | USA. Whether the people involved were in urban and rural environments was not reported. |

### Comparison 35 E– Shorter recruitment pack with simplified consent vs. Standard recruitment package (GRADE: very low)

It is very uncertain whether a shorter recruitment pack with simplified consent affects recruitment compared to a standard recruitment pack. RD = 2% (95% CI = -13% to 17%); GRADE: very low (-1 level: study limitations-unclear RoB; -2 levels: imprecision-single study; wide CI crossing RD=0).

**Equity information (see** Table 2)

This result and its GRADE assessment apply to people living in the USA but to whom exactly is unclear as more participant characteristics were not provided by the authors. The GRADE assessment would be lower for other groups because of indirectness were it not already at very low.

This is based on one study: Chow 2020 (cancer) RoB: unclear.

|  |  |
| --- | --- |
| **PRO EDI Participant characteristics table for standard recruitment package vs shorter recruitment pack with simplified consent** | |
| **Characteristic** | **The people who took part** |
| Age | No information provided by authors. |
| Sex | No information provided by authors. |
| Gender | No information provided by authors. |
| Race, ethnicity and ancestry | No information provided by authors. |
| Socio-economic status (SES) | No information provided by authors. |
| Location (country/ countries of data collection and site coordination) | USA. Whether the people involved were in urban and rural environments was not reported. |

### Comparison 36 E–Leaflet advertising PPI involvement in trial + standard trial invitation vs standard trial invitiation only (GRADE: moderate)

Including a leaflet advertising PPI involvement in trial alongside the standard trial invitation probably makes little to no difference in recruitment. RD = -1% (reduced recruitment) (95% CI = -2% to 0%); GRADE: moderate (-1 level: imprecision-single study).

**Equity information (see** Table 2)

This result and its GRADE assessment apply to middle-aged, especially female, people who are more socioeconomically disadvantaged and who live in the UK. The GRADE assessment when applied to groups other than those mentioned will be below moderate because of indirectness.

This is based on one study: Hughes-Morley 2016 (mental health) RoB: low.

|  |  |
| --- | --- |
| **PRO EDI Participant characteristics table for leaflet advertising PPI involvement in trial + standard trial invitation vs standard trial invitation only** | |
| **Characteristic** | **The people who took part** |
| Age | Intervention  Mean age = 48.5 (SD = 12.8)   Comparison  Mean age = 45.5 (SD = 9.3) |
| Sex | Intervention  Male = 36.2%, female = 63.8%   Comparison  Male = 36%, female = 64% |
| Gender | No information provided by authors. |
| Race, ethnicity and ancestry | No information provided by authors. |
| Socio-economic status (SES) | Intervention  Index of multiple deprivation, median = 1.5, range 1 - 4 (where 1 is most deprived on scale of 1 - 5)   Comparison  Index of multiple deprivation, median = 2.5, range 1 - 5 |
| Location (country/ countries of data collection and site coordination) | UK. Whether the people involved were in urban and rural environments was not reported. |

### Comparison 37 E–Gender-targeted recruitment postcard vs generic recruitment postcard (GRADE: low)

Gender targeted recruitment postcards may have little or no impact on recruitment compared to generic recruitment postcards. RD = 0% (95% CI = -0% to 0%); GRADE: low (-1 level: study limitations-unclear RoB; -1 level: imprecision-single study); Analysis X.

**Equity information (see** Table 2)

This result and its GRADE assessment are based on a study involving mostly females who live in the USA. The GRADE assessment when applied to other specific groups will be below low because of indirectness.

This is based on one study: Crane 2016 (weight management) RoB: unclear.

|  |  |
| --- | --- |
| **PRO EDI Participant characteristics table for Gender-targeted recruitment postcard vs generic recruitment postcard** | |
| **Characteristic** | **The people who took part** |
| Age | No information provided by authors. |
| Sex | Of 85 respondents, 23 were male, and 62 were female. |
| Gender | No information provided by authors. |
| Race, ethnicity and ancestry | No information provided by authors. |
| Socio-economic status (SES) | No information provided by authors. |
| Location (country/ countries of data collection and site coordination) | USA |

### Comparison 38 E–Researcher optimised PIL vs standard PIL (GRADE: low)

A PIL optimised by researchers compared to a standard PIL probably reduces recruitment. RD = -9% (reduced recruitment) (95% CI = -17% to 0%); GRADE: low (-1 level: imprecision-single study; wide CI including RD=0).

**Equity information (see** Table 2)

This result and its GRADE assessment apply to middle-aged people with a broad range of socioeconomically backgrounds and who live in France. The GRADE assessment when applied to groups other than those mentioned will be below moderate because of indirectness.

This is based on one study: Paris 2015 (cardiovascular) RoB: low.

|  |  |
| --- | --- |
| **PRO EDI Participant characteristics table for researcher optimised PIL vs standard PIL** | |
| **Characteristic** | **The people who took part** |
| Age | Intervention  Mean age = 46.7 (SD = 18.2)   Comparison  Mean age = 46.8 (SD = 19.4) |
| Sex | No information provided by authors. |
| Gender | Intervention  40.6% men   Comparison  59.4% men |
| Race, ethnicity and ancestry | No information provided by authors. |
| Socio-economic status (SES) | Intervention  Farmers: 1/184 (0.5)  Tradesmen, shopkeepers and business owners: 2/184 (1.1)  Managerial and professional occupations:19/184 (10.3)  Intermediary professions: 30/184 (16.3)  Employees:54/184 (29.4)  Workers:9/184 (4.9)  Pensioners:36/184 (19.6)  Others with no employment: 33/184 (17.9)   Comparison  Farmers:2/199 (1.0)  Tradesmen, shopkeepers and business owners: 4/199 (2.0)  Managerial and professional occupations: 18/199 (9.0)  Intermediary professions:32/199 (16.1)  Employees:51/199 (25.6)  Workers:19/199 (9.6)  Pensioners:40/199 (20.1)  Others with no employment: 33/199 (16.6) |
| Location (country/ countries of data collection and site coordination) | France. Whether the people involved were in urban and rural environments was not reported. |

### Comparison 39 E–In-clinic information video viewing vs information DVD to take home (high risk of bias)

This is a single study, with high risk of bias. We have not presented the results.

### Comparison 40 E–Brief PIL + standard PIL vs standard PIL (GRADE: low)

Providing a brief PIL alongside the standard PIL may make little to no difference in recruitment. RD = 0% (95% CI = -1% to 0%); GRADE: low (-1 level: study limitations-unclear RoB; -1 level: imprecision-single study).

**Equity information (see** Table 2)

This result and its GRADE assessment is based on a study mostly involving white British females around the age of 46 with good education living in the UK. The GRADE assessment when applied to other specific groups will be below low because of indirectness.

This is based on one study: White 2023 (persistent, medically unexplained, physical symptoms) RoB: unclear.

|  |  |
| --- | --- |
| **PRO EDI Participant characteristics table for brief PIL + standard PIS vs standard PIS** | |
| **Characteristic** | **The people who took part** |
| Age | [9] Mean age was 45.9 |
| Sex | Male: 185 (17.2%)  Female: 889 (82.8%) |
| Gender | No information provided by authors. |
| Race, ethnicity and ancestry | 87.1% of participants randomised were British (assumed white). 0.6% were Irish, 5.3% Other white, 2.5% Indian, 0.9% Pakistani, 0.,3% Bangladeshi, 0.3% Other Asian, 0.3% White and Black African, 0.3% white and Asian, 0.6% Mixed/multiple, 0.3% Caribbean, 0.3% Other black, 0.6% Arab and 0.3% Other. |
| Socio-economic status (SES) | Education: overall 4.8% had no qualifications, 8.6% had a higher degree, 26.1% had a bachelor’s degree, 25.2% had an A-level or equivalent, 35.4% had GCSE or equivalent. |
| Location (country/ countries of data collection and site coordination) | UK. Mixture of urban and rural environments. |

### Comparison 41 E–Decision aid about RCTs vs blank notebook (GRADE: low)

A decision aid about RCTs may result in a large increase in recruitment. RD = 12% (95% CI = -1% to 24%); GRADE: low (-2 level: imprecision-single study; wide CI crossing RD=0).

**Equity information (see** Table 2)

This result and its GRADE assessment apply to older males with a broad range of countries of birth and education living in Australia and New Zealand. The GRADE assessment when applied to groups other than those mentioned will be below low because of indirectness

This is based on one study: Sundaresan 2017 (cancer) RoB: low.

|  |  |
| --- | --- |
| **PRO EDI Participant characteristics table for decision aid about RCTs vs blank notebook** | |
| **Characteristic** | **The people who took part** |
| Age | Mean age = 64 |
| Sex | 100% male |
| Gender | No information provided by authors. |
| Race, ethnicity and ancestry | Country of birth:  Australia- 88 (68%)  Europe- 22 (17%)  Africa- 7 (6%  Asia- 5 (4%)  New Zealand- 3 (2%)  United States of America- 2 (1.5%)  Did not specify- 2 (1.5%) |
| Socio-economic status (SES) | Highest level of education:  Intermediate (Year 10 or below)- 23 (18%)  Leaving (Year 12/HSC)- 15 (12%)  TAFE certificate/diploma-41 (32%)  University degree- 26 (2%)  Postgraduate degree- 22 (17%)  Did not specify- 2 (1.5%) |
| Location (country/ countries of data collection and site coordination) | Australia and New Zealand. Whether the people involved were in urban and rural environments was not reported. |

### Comparison 42 E–Interactive online digital eductional tool vs time-matched webiner (GRADE: low)

An interactive online digital educational tool may make little to no difference in recruitment compared to a time-matched webinar. RD = 1% (95% CI = -1% to 3%); GRADE: low (-1 level: study limitations-unclear RoB; -1 level: imprecision-single study).

**Equity information (see** Table 2)

This result and its GRADE assessment apply to older people, especially females, from a range of ethnicities (though predominantly white) with varying amounts of education living in the USA. The GRADE assessment when applied to groups other than those mentioned will be below low because of indirectness.

This is based on one study: Saif 2020 (Alzheimer's disease) RoB: uncertain.

|  |  |
| --- | --- |
| **PRO EDI Participant characteristics table for interactive online digital educational tool vs time-matched webinar** | |
| **Characteristic** | **The people who took part** |
| Age | Mean age = 68 |
| Sex | 18.23% male, 77.78% female, prefer not to say 0.28%, no response 3.7% |
| Gender | No information provided by authors. |
| Race, ethnicity and ancestry | White/Caucasian- 304 (86.61%)  Hispanic/Latino- 16 (4.56%)  Black/African American- 9 (2.56%)  Asian/Pacific Islander- 9 (2.56%)  Middle Eastern/Arab American- 5 (1.42%)  Native America/American Indian- 2 (0.57%)  Other/Mixed- 5 (1.42%)  Prefer not to say- 2 (0.57%)  No response- 12 (3.42%) |
| Socio-economic status (SES) | Education level  Elementary/primary- 1 (0.28%)  High school/ secondary- 68 (19.37%)  Associates- 46 (13.10%)  Bachelors- 87 (24.79%)  Post-graduate- 129 (36.75%)  Prefer not to say- 0 (0%)  No response- 4 (1.14%) |
| Location (country/ countries of data collection and site coordination) | USA. Whether the people involved were in urban and rural environments was not reported. |

### Comparison 43 E–PPI designed and informed PIL vs standard researcher designed PIL (GRADE: very low)

It is very uncertain whether a PPI designed-and-informed PIL affects recruitment compared to a standard researcher-designed PIL. RD = -2% (reduced recruitment) (95% CI = -15% to 12%); GRADE: very low (-1 level: study limitations-unclear RoB; -2 levels: imprecision-single study; wide CI crossing RD=0).

**Equity information (see** Table 2)  
This result and its GRADE assessment apply to people living in Ireland but to whom exactly is unclear as more participant characteristics were not provided by the authors. The GRADE assessment would be lower for other groups, were it not already at very low because of indirectness

This is based on one study: Dwyer 2022 (multiple sclerosis) RoB: uncertain.

|  |  |
| --- | --- |
| **PRO EDI Participant characteristics table for PPI-designed-and-informed PIL vs. standard researcher-designed PIL** | |
| **Characteristic** | **The people who took part** |
| Age | No information provided by authors. |
| Sex | No information provided by authors. |
| Gender | No information provided by authors. |
| Race, ethnicity and ancestry | No information provided by authors. |
| Socio-economic status (SES) | No information provided by authors. |
| Location (country/ countries of data collection and site coordination) | Ireland. Whether the people involved were in urban and rural environments was not reported. |

### 44 E–Culturally-tailored educational DVD about clinical trials + usual education vs usual education by healthcare staff only (high risk of bias)

This is a single study, with high risk of bias. We have not presented the results.

### Comparison 45 E–Multimedia education about clinical trials (DVD + booklet) vs standard educational booklet only (GRADE: low)

Providing multimedia education about clinical trials as a DVD and booklet may increase recruitment. RD = 4% (95% CI = -5% to 14%); GRADE: low (-2 levels: imprecision-single study; wide CI crossing RD=0).

**Equity information (see** Table 2)

This result and its GRADE assessment apply to older people, especially females, who are predominantly white with a range of incomes and education living in the USA. The GRADE assessment when applied to groups other than those mentioned will be below low because of indirectness.

This is based on one study: Kamen 2018 (cancer) RoB: low.

|  |  |
| --- | --- |
| **PRO EDI Participant characteristics table for multimedia education about clinical trials (DVD + booklet) vs standard educational booklet only** | |
| **Characteristic** | **The people who took part** |
| Age | Mean age = 61.5 (SD = 11.9) |
| Sex | Males = 39%, females = 61% |
| Gender | No information provided by authors. |
| Race, ethnicity and ancestry | Race provided  White: 372 (89.0%)  Black: 37 (8.9%)  Asian/Pacific Islander: 5 (1.2%)  Unknown: 4 (0.9%) |
| Socio-economic status (SES) | Yearly household income  <$20,000: 61 (14.8%)  $20,000-$39,999: 64 (15.6%)  $40,000-$59,999: 62 (15.1%)  $60,000-$100,000: 81 (19.7%)  >$100,000: 44 (10.7%)  Prefer not to answer: 99 (24.1%)   Level of education  <= High school: 151 (36.2%)  Partial college: 136 (32.7%)  >= College: 129 (31.1%) |
| Location (country/ countries of data collection and site coordination) | USA. Whether the people involved were in urban and rural environments was not reported. |

### Comparison 46 E–PIL minimising the use of phrases with negative connotations vs standard PIL (high risk of bias)

This is a single study, with high risk of bias. We have not presented the results.

### Comparison 47 E–Multimedia information (viewed in person) + paper PIL vs paper PIL only (GRADE: very low)

It is very uncertain whether providing multimedia information (viewed in person) alongside a paper PIL increases recruitment. RD = 17% (95% CI = -89% to 123%); GRADE: very low (-1 level: inconsistency; -2 levels: imprecision-extremely wide CI crossing RD=0)

**Equity information (see** Table 2)

This result and its GRADE assessment are based on studies involving a small number of male and female children aged between 6 and 13 living in the UK who are not white and live in mostly disadvantaged areas. The GRADE assessment would be lower for other groups because of indirectness were it not already at very low.

This is based on one study: Knapp 2023 (three evaluations, Knapp 2023a, Knapp 2023b and Knapp 2023f RoB: low.

|  |  |
| --- | --- |
| **PRO EDI Participant characteristics table for multimedia information {viewed in person} + paper PIS vs paper PIS only**[11] | |
| **Characteristic** | **The people who took part** |
| Age | Knapp 2023f provided no information. The two other studies involved children with median ages between 5 and 12.5. |
| Sex | Knapp 2023f provided no information. The other two studies had an even mix of males and females. |
| Gender | No information provided by authors. |
| Race, ethnicity and ancestry | Knapp 2023 b and 2023f provided no information. Limited information was provided in Knapp 2023a, and most participants were classed as non-white.   [12] |
| Socio-economic status (SES) | Knapp 2023f provided no information. The other two studies included participants from mostly more disadvantaged areas. |
| Location (country/ countries of data collection and site coordination) | UK. Whether the people involved were in urban and rural environments was not reported. |

### Comparison 48. E–Multimedia information (viewed in person) PIL vs paper PIL (GRADE: Moderate)

Providing a multimedia information PIL (viewed in person) probably increases recruitment slightly compared to a paper PIL. RD = 4% (95% CI = -1% to 8%); GRADE: moderate (-1 level: inconsistency)

**Equity information (see** Table 2)

This result and its GRADE assessment are based on studies involving male and female children aged between 6 and 13 living in the UK of mixed ethnicities living in mostly disadvantaged areas. The GRADE assessment when applied to other specific groups will be below moderate because of indirectness.

These findings are based on three studies: Knapp 2023aa, Knapp 2023bb and Knapp 2023d (BALANCE)(paediatric lazy eye) RoB: low; Knapp 2023b (BAMP)(paediatric underbite) RoB: low; Knapp 2023d (FORCE)(paediatric wrist fracture) RoB: low.

|  |  |
| --- | --- |
| **PRO EDI Participant characteristics table for multimedia information {viewed in person} PIS vs paper PIS only** | |
| **Characteristic** | **The people who took part** |
| Age | Children with median/mean ages between 5 and 12. |
| Sex | Overall, the mix of male and female participants was reasonable, ranging between 38% and 67% female depending on the study. |
| Gender | No information provided by authors. |
| Race, ethnicity and ancestry | Knapp 2023b provide no information. Limited information was provided in Knapp 2023a, and most participants were classed as non-white. Participants were predominantly (>80%) white in Knapp 2023d but around 10% were Asian/Asian British, 5% Black/African/Caribbean/Black British, Mixed/multiple ethnic groups around 1.5%. |
| Socio-economic status (SES) | All three studies included participants from mostly more disadvantaged areas. |
| Location (country/ countries of data collection and site coordination) | UK. Whether the people involved were in urban and rural environments was not reported. |

### Comparison 49 E–Multimedia information (provided as link/QR code) + paper PIL vs paper PIL (GRADE: high)

Providing multimedia information in the form of a link or QR code alongside a paper PIL makes little or no difference to recruitment. RD = 0% (95% CI = -1% to 1%); GRADE: high

**Equity information (see** Table 2)

This result and its GRADE assessment apply to people living in the UK, some of whom were male and female children between the ages of 9 months and 9 years with mixed ethnicities but reporting in most studies was very limited. The GRADE assessment when applied to other specific groups will be below high because of indirectness.

This is based on seven studies (11612 participants): Jolly 2019 (START MMI PSM-COPD) (COPD), RoB: low; Knapp 2022 (Thermic-3 Knapp 2023: paediatric congenital heart defects), RoB: low; Knapp 2023c (CHAMP-UK) ( paediatric short-sightedness), RoB: low; Madurasinghe 2023 (START MMI GHT2000a) (exercise for high blood pressure), RoB: low; Madurasinghe 2023 (START MMI GHT2000b) (exercise for high blood pressure), RoB: low; Madurasinghe 2023 (START MMI HI-Light) (vitiligo), RoB: low; Madurasinghe 2023 (START MMI seAFOod) (bowel cancer prevention), RoB: low.

|  |  |
| --- | --- |
| **PRO EDI Participant characteristics table for multimedia information {provided as link/QR code} + paper PIS vs paper PIS only** | |
| **Characteristic** | **The people who took part** |
| Age | Only two studies (Knapp 2023c and Knapp 2023e) provided information. These studies involved children with median ages between 9 months and 9 years. |
| Sex | Only two studies (Knapp 2023c and Knapp 2023e) provide information. These studies involved between around 35% and 40% females. |
| Gender | No information provided by authors. |
| Race, ethnicity and ancestry | Only Knapp 2023c provided information:   Intervention  Asian/Asian British = 1/29 (3.5%)  Black/African/Caribbean/Black British = 1/29 (3.5%)  White = 23/29 (79.3%)  Mixed/multiple ethnic groups = 0  Other ethnic group = 4/29 (13.8%)  Not stated = 0   Comparison  Asian/Asian British = 6/29 (20.7%)  Black/African/Caribbean/Black British = 2/29 (6.9%)  White = 14/29 (48.3%)  Mixed/multiple ethnic groups = 1/29 (3.5%)  Other ethnic group = 5/29 (17.2%)  Not stated = 1/29 (3.5%) |
| Socio-economic status (SES) | No information provided by authors. |
| Location (country/ countries of data collection and site coordination) | UK. Whether the people involved were in urban and rural environments was not reported. |

### Comparison 50 E–Multimedia information (provided as link/QR code) vs paper PIL (GRADE: moderate)

Providing multimedia information in the form of a link or QR code probably results in a large increase in recruitment compared to a paper PIL only. RD = 13% (95% CI = 1% to 26%); GRADE: moderate (-1 level: imprecision-wide CI).

**Equity information (see** Table 2)

This result and its GRADE assessment are based on studies involving This result and its GRADE assessment are based on studies involving children of both sexes between approximately 8 months and 9 years living in the UK with a range of ethnicities and living in mostly disadvantaged areas, although in one study (Knapp 2023cc, CHAMP-UK), parents were predominantly female, highly educated, and employed. The GRADE assessment when applied to other specific groups will be below moderate because of indirectness. .

This is based on two studies: Knapp 2023e (Thermic-3)(paediatric congenital heart defects) RoB: low; Knapp 2023c (CHAMP-UK)(paediatric short-sightedness) RoB: low.

|  |  |
| --- | --- |
| **PRO EDI Participant characteristics table for multimedia information {provided as link/QR code} vs paper PIS only** | |
| **Characteristic** | **The people who took part** |
| Age | Children with median ages between approximately 8 months and 9 years |
| Sex | Knapp 2023e (Thermic-3):  Child’s gender: 62% were male, and 38% were femaleNo information provided by authors.   Knapp 2023c (CHAMP-UK):  Child’s gender: 49.6% female, 50.4% male  Parent/carer’s gender: 92.5% female |
| Gender | No information provided by authors. |
| Race, ethnicity and ancestry | Only Knapp 2023cc provided information:   Intervention  Asian/Asian British = 4/38 (10.4%)  Black/African/Caribbean/Black British = 1/38 (2.6%)  White = 25/38 (65.8%)  Mixed/multiple ethnic groups = 6/38 (15.8%)  Other ethnic group = 0  Not stated = 2/38 (5.2%)   Comparison  Asian/Asian British = 6/29 (20.7%)  Black/African/Caribbean/Black British = 2/29 (6.9%)  White = 14/29 (48.3%)  Mixed/multiple ethnic groups = 1/29 (3.5%)  Other ethnic group = 5/29 (17.2%)  Not stated = 1/29 (3.5%) |
| Socio-economic status (SES) | Only Knapp 2023ee provided information and most participants lived in a more disadvantaged area. Parent/carer education level: 47.4% had a university degree or higher. |
| Location (country/ countries of data collection and site coordination) | UK. Whether the people involved were in urban and rural environments was not reported. |

### Comparison 51 E-Educational material to provide additional information about a trial. (GRADE: unable to complete, no forest plot available)

Although the authors of this cluster trial did not present centre‐level recruitment data, or provide an intracluster correlation coefficient, they did consider intracluster correlation in their analysis. An educational package did not significantly increase recruitment compared to standard information alone (31% of participants aged over 65 in both intervention and control groups in year 2, P = 0.83).

**Equity information (see** Table 2)

This result and its GRADE assessment are based on studies involving people living in the USA but to whom exactly is unclear as more participant characteristics were not provided by the authors.

This is based on one study (126 centres): Kimmick 2005 (cancer) RoB: unclear. We were unable to complete a GRADE assessment for this comparison due to lack of data.

|  |  |
| --- | --- |
| **PRO EDI Participant characteristics table for multimedia information {provided as link/QR code} + paper PIS vs paper PIS only** | |
| **Characteristic** | **The people who took part** |
| Age | No information provided by authors. |
| Sex | No information provided by authors. |
| Gender | No information provided by authors. |
| Race, ethnicity and ancestry | No information provided by authors. |
| Socio-economic status (SES) | No information provided by authors. |
| Location (country/ countries of data collection and site coordination) | USA. Whether the people involved were in urban and rural environments was not reported. |

### Comparison 52 E-Trained recruiters from a similar ethnic background to study population already taking part in a trial as lay advocates (high risk of bias, no forest plot available)

Trained recruiters from a similar ethnic background to study population already taking part in a trial as lay advocates. The authors of this cluster trial did not report an analysis that corrected for the clustering or provide an intracluster correlation coefficient. Data at the recruiter aggregate level were reported on whether a recruiter did or did not recruit anyone to the trial. Eight of the 28 trained Hispanic recruiters recruited one or more women to the trial whereas none of the 26 untrained Hispanic women recruited anyone for the trial. Two of the 42 untrained Anglo control group recruited two women.

**Equity information (see** Table 2)

We were unable to complete a GRADE assessment for this comparison due to lack of data. This study provides minimal information, and we have therefore not presented the characteristics of included participants.

This is based on one study (number of participants unclear): Larkey 2002 (unclear) RoB: unclear.

### Comparison 53 E-Using a coach to promote trust among minority patients (high risk of bias, no forest plot available)

Using a coach to promote trusts among minority patients did not significantly increase trial enrollment compared to usual care. The authors report the number of participants in each arm who went on to be enrolled into a trial (16 participants in the intervention group and 13 participants in the control group, P=0.35). However we were unable to determine the proportion of participants who went on to enroll in a trial in each study arm as they did not report the numbers of participants who were randomised to receive coaching versus usual care. This is based on one study (75 participants): Fracasso 2013 (cancer), RoB unclear. We were unable to complete a GRADE assessment for this comparison due to lack of data. This study provides limited information, and we have therefore not presented the characteristics of included participants.

## F: Interventions aimed at the recruiter or recruitment site

### Comparison 54 F–Teaser campaign using postcards vs no teaser (GRADE: moderate)

Using a postcard teaser campaign probably makes little to no difference in recruitment. RD = 0% (95% CI = -4% to 5%); GRADE: moderate (-1 level: imprecision-single study).

**Equity information (see** Table 2)

This result and its GRADE assessment apply to practices and clinics in the Australia but to whom exactly is unclear as more participant characteristics were not provided by the authors. The GRADE assessment when applied to specific groups will be below moderate because of indirectness.

This is based on one study: Lee 2017 (recruiting GP practices to low back pain trial) RoB: low.

|  |  |
| --- | --- |
| **PRO EDI Participant characteristics table for teaser campaign using postcards vs no teaser** | |
| **Characteristic** | **The people who took part** |
| Note: | This study recruited general practice and physiotherapy clinics, rather than patients/participants. It would have been useful to know something about the populations served by these practices and centres. |
| Age | No information provided by authors. |
| Sex | No information provided by authors. |
| Gender | No information provided by authors. |
| Race, ethnicity and ancestry | No information provided by authors. |
| Socio-economic status (SES) | No information provided by authors. |
| Location (country/ countries of data collection and site coordination) | Australia. Whether the people involved were in urban and rural environments was not reported. |

### Comparison 55 F–Educational program to tailor recruitment strategies for minority recruitment vs usual recruitment (GRADE: low)

Using an educational program to tailor recruitment strategies for minority recruitment may have little to no impact on recruitment. RD = 0% (95% CI = -4% to 3%); GRADE: low (-1 level: study limitations-unclear RoB; -1 level: imprecision-single study).

**Equity information (see** Table 2)

This result and its GRADE assessment apply to predominantly Latinx white people, especially those who speak English, and who live in the USA. The GRADE assessment when applied to groups other than those mentioned will be below low because of indirectness.

This is based on one study: Tilley 2021 (various) RoB: uncertain.

|  |  |
| --- | --- |
| **PRO EDI Participant characteristics table for educational program to tailor recruitment strategies for minority recruitment vs usual recruitment** | |
| **Characteristic** | **The people who took part** |
| Age | No information provided by authors. |
| Sex | Intervention  45% female   Comparison  37% female |
| Gender | No information provided by authors. |
| Race, ethnicity and ancestry | Intervention  American Indian/Alaskan  Native- 2 (1%)  Asian- 17 (8%)  Black- 20 (9%)  Latinx- 12 (5%)  Native Hawaiian/Pacific  Islander- 1 (0%)  More than one race- 1 (0%)   Total Latinx White- 172 (76%)   Comparison  American Indian/Alaskan  Native- 1 (1%)  Asian- 5 (3%)  Black- 6 (4%)  Latinx- 13 (8%)  Native Hawaiian/Pacific  Islander- 0 (0%)  More than one race- 1 (1%)   Total Latinx White- 136 (84%) |
| Socio-economic status (SES) | Intervention  % Non-English speaking ≤30 miles of site:  - 0%- < 10% : 7(26.9%)  - 10%- < 20%: 7(26.9%)  - 20%- < 30%: 5 (19.2%)  30% or more: 7 (26.9%)   Comparison  % Non-English speaking ≤30 miles of site:  - 0%- < 10%: 8 (33.3%)  - 10%- < 20%: 7 (29.2%)  - 20%- < 30%: 3 (12.5%)  - 30% or more: 6 (25.0%) |
| Location (country/ countries of data collection and site coordination) | USA. Whether the people involved were in urban and rural environments was not reported. |

### Comparison 56 F–Remote vs face-to-face site initiation visits (GRADE: moderate)

Using remote site initiation visits probably increases recruitment face-to-face site initiation visits. RD = 6% (95% CI = -1% to 13%); GRADE: moderate (-1 level: imprecision-single study).

**Equity information (see** Table 2)

This result and its GRADE assessment apply to the UK but to whom exactly is unclear as more participant characteristics were not provided by the authors. The GRADE assessment when applied to specific groups will be below moderate because of indirectness.

This is based on one study: Jefferson 2018 (fractures) RoB: low.

|  |  |
| --- | --- |
| **PRO EDI Participant characteristics table for remote vs face-to-face site initiation visits** | |
| **Characteristic** | **The people who took part** |
| Age | No information provided by authors. |
| Sex | No information provided by authors. |
| Gender | No information provided by authors. |
| Race, ethnicity and ancestry | No information provided by authors. |
| Socio-economic status (SES) | No information provided by authors. |
| Location (country/ countries of data collection and site coordination) | UK. Whether the people involved were in urban and rural environments was not reported. |

### Comparison 57 F-Recruitment coordinator conducting teleconferences with site teams to enhance trial recruitment using software for identifying eligible patients, with a second teleconference after 6 months (high risk of bias)

We were unable to complete a GRADE assessment for this comparison due to lack of data. This study provides minimal information, and we have therefore not presented the characteristics of included participants.

### Comparison 58 F-Using teleconference and commitment contracts with site teams to enhance recruitment (Unclear risk of bias, GRADE- unable to complete, no forest plot available)

We were also unable to complete a GRADE assessment for this comparison due to lack of data. This study provides minimal information, and we have therefore not presented the characteristics of included participants.

### Comparison 59 F-Onsite initiation visits versus no initiation visit (Unclear risk of bias, GRADE- unable to complete, no forest plot available)

We were also unable to complete a GRADE assessment for this comparison due to lack of data. This study provides minimal information, and we have therefore not presented the characteristics of included participants.

### Comparison 60 F-Additional communication strategies such as tailored feedback on recruitment (low risk of bias, GRADE- unable to complete, no forest plot available)

We were also unable to complete a GRADE assessment for this comparison due to lack of data. This study provides minimal information, and we have therefore not presented the characteristics of included participants.

### Comparison 61 F-Recruiter training with ongoing support and/or theory-informed personalised nudge to the recruiter after each randomisation may increase recruitment (low risk of bias, GRADE- unable to complete, no forest plot available)

We were also unable to complete a GRADE assessment for this comparison due to lack of data. This study provides minimal information, and we have therefore not presented the characteristics of included participants.

### Comparison 62 F-Automatically flagging eligible patients to clinicians via electronic health records vs. manually identifying patients via electronic health records (high risk of bias, GRADE- unable to complete, no forest plot available)

We were also unable to complete a GRADE assessment for this comparison due to lack of data. This study provides minimal information, and we have therefore not presented the characteristics of included participants.

## G: Incentives

### Comparison 63 G–Monetary incentive vs no incentive (GRADE: low)

Monetary incentives offered to potential participants may improve recruitment compared to no incentive. RD = 4% (95% CI = 2% to 7%); GRADE: low (-1 level: study limitations-high RoB; 1 level: inconsistency);

**Equity information (see** Table 2)

This result and its GRADE assessment are based on studies involving both sexes aged from their 30s to mid-60s, probably mostly white ethnicity (but good Black/African American involvement in two studies) and a reasonable spread of socioeconomic status living in Canada, UK and the USA. The GRADE assessment when applied to other specific groups will be below low because of indirectness.

This is based on 12 studies: Fairhurst 2022 (yoga for older adults with multimorbidity) RoB: high; Free 2010 (smoking cessation) RoB: low; Giguere 2015 (recruiting family physicians to a medical education trial) RoB: low; Jennings 2015a; Jennings 2015b; Jennings 2015c; Jennings 2015d; Jennings 2015e (primary care, older people, mainly hypertension) RoB: low; Halpern 2021a ($200); Halpern 2021a ($500); Halpern 2021b ($100); Halpern 2021b ($300) (ambulation, smoking cessation) RoB: high.

**Note:** the evidence mainly comes from high incentive amounts. When applied to settings with low incentive values the GRADE rating would be downgraded to very low.

|  |  |
| --- | --- |
| **PRO EDI Participant characteristics table for Monetary Monetary incentive vs no incentive** | |
| **Characteristic** | **The people who took part** |
| Age | One study (Giguere 2015) provided no information. Participants in Free 2010 had a mean age of 33. All other studies had mean ages between 47 and 66. |
| Sex | Free 2010 and Giguere 2015 provided no information. Participants in both Halpern studies were around 50% female, although Halpern 2021a only had 38% male, with 11% have no sex information. Jennings 2015 was 58% male. |
| Gender | No information provided by authors. |
| Race, ethnicity and ancestry | Only the two Halpern studies provided data and both studies had a good range of ethnicities represented. Halpern 2021a had 60% Black/African American, 32% White. Halpern 2021b had 35% Black/African American, 52% White and Other 6%. |
| Socio-economic status (SES) | Free 2010 and Giguere 2015 provided no information. The other studies had a good range of socioeconomic status represented, including people from the most disadvantaged groups. Jennings 2015 had 29% from the most disadvantaged areas, Halpern 2021 had 33% of participants having household incomes of under 19,000 USD per year; Halpern 2021b had 9%. |
| Location (country/ countries of data collection and site coordination) | Canada, UK and USA. Jennings 2015 included both rural and urban areas; this was unclear for other studies. |

### Comparison 64 G–Monetary incentive: higher value vs lower value (GRADE: very low)

It is very uncertain whether offering a higher monetary incentive affects recruitment compared to offering a lower monetary incentive. RD = 3% (95% CI = -13% to 19%). GRADE: very low (-2 level: study limitations-high RoB; -1 level: inconsistency; -1 level: imprecision-wide CI crossing RD= 0).

**Equity information (see** Table 2)

This result and its GRADE assessment apply to middle-aged males and females, with a range of ethnicities and socioeconomic status living in the USA. The GRADE assessment would be lower for other groups because of indirectness were it not already at very low.

This is based on two studies: Halpern 2021a (smoking cessation) RoB: high; Halpern 2021b (physical activity) RoB: high.

|  |  |
| --- | --- |
| **PRO EDI Participant characteristics table for higher monetary incentive compared to a lower monetary incentive** | |
| **Characteristic** | **The people who took part** |
| Age | The mean age of participants was around 50. |
| Sex | Around 40% of participants were male. |
| Gender | No information provided by authors |
| Race, ethnicity and ancestry | Overall, participants were relatively evenly divided between Black/African Americans (60% in one study, 35% in the other) and White people.   No other groups were named, although they accounted for around 5%. |
| Socio-economic status (SES) | Household income was reported.   People from the full range of socioeconomic status were included, with around 10% to 30% from the most disadvantaged communities. |
| Location (country/ countries of data collection and site coordination) | Both studies were done in a high-income country, the USA. Whether the people involved were in urban and rural environments was not reported. |

### Comparison 65. G–Non-monetary incentive (branded pen vs no pen) (GRADE: Very low)

Offering a non-monetary incentive in the form of a trial-branded pen alongside the trial invitation probably has little or no effect on recruitment. RD = 0% (95% CI = -1% to 1%). GRADE: moderate (-1 level: study limitations-high RoB).

**Equity information (see** Table 2)

This result and its GRADE assessment apply to the UK, probably both urban and rural settings, but to who else is unclear as more participant characteristics were not provided by the authors. The GRADE assessment when applied to groups other than those mentioned will be below moderate because of indirectness.

This is based on three studies: Whiteside 2019 (falls reduction) RoB: low; Fairhurst 2022 (improving quality of life in older people) RoB: high; White (persistent, medically unexplained, physical symptoms RoB: unclear.

|  |  |
| --- | --- |
| **PRO EDI Participant characteristics table for Non-monetary incentive (branded pen vs no pen)** | |
| **Characteristic** | **The people who took part** |
| Age | Only one study (White 2023) reported age. Mean age was 46.1, range was 18-70. |
| Sex | Only one study (White 2023) reported sex 83% of participants were female, and 17% were male. |
| Gender | No information provided by authors. |
| Race, ethnicity and ancestry | Only one study (White 2023) reported ethnicity.   Majority were British (87.1%)  Other White backgrounds: 5.3%   Asian ethnicities:  Indian: 2.5%  Pakistani: 0.9%  Bangladeshi: 0.3%  Other Asian: 0.3%  Mixed ethnicities: 1.2% (White and Asian, and Mixed/Multiple)   Black ethnicities:  Caribbean: 0.3%  Other Black: 0.3%  Arab: 0.6%   Other ethnicity: 0.3%   No participants identified as Gypsy/Irish Traveller or African |
| Socio-economic status (SES) | No information provided by authors. |
| Location (country/ countries of data collection and site coordination) | UK. Mixture of urban and rural environments. |
