# Supplementary material for: Strategies to improve recruitment to randomised trials
Source: Cochrane Database Syst Rev. 2026 Sep 15;2026(9):MR000013. doi: 10.1002/14651858.MR000013.pub7 (PMC13576036; doi:10.1002/14651858.MR000013.pub7)
Supplement: Supplementary file 12 [file MR000013-SUP-12-other.html]

Cost-effectiveness of recruitment strategies


# Supplementary material 12 to: Strategies to improve recruitment to randomised trials

Parker A, Mongelli G, Piccolo-Lawrance C, Coleman E, Gkekas A, Wang HI, Dawson S, Green H, Way R, Rai A, Bruhn H, Sullivan F, Boxall C, Chan M, Manson P, Hewitt C, Fairhurst C, Clarke M, Treweek S
  
https://doi.org/10.1002/14651858.MR000013.pub7

The material in this section has been supplied by the author(s) for publication under a Licence for Publication and the author(s) are solely responsible for the material. Cochrane has reviewed this material, but Cochrane has not copyedited, formatted or proofread. Cochrane accordingly gives no representations or warranties of any kind in relation to, and accepts no liability for any reliance on or use of, such material.

Back to top

# Cost-effectiveness of recruitment strategies

#### Trial conduct changes ‐ category C

**9. Telephone reminders versus SMS reminders.** Cost-effectiveness data were extracted from the single study included in this comparison (Bracken 2019). Telephone reminders, versus SMS reminders, have an estimated ICER of £72.72, with incremental costs of £3.64 (consisting of inflation-adjusted direct and staff time unit costs) and an effectiveness of 5%. However, the effectiveness of this strategy is not statistically significant (RD: 0.05, 95% CI: from -0.01 to 0.11), which introduces uncertainty regarding its cost-effectiveness figure.

#### Modification to the information given to potential participants about the trial ‐ category E

**11. Recruitment primer letter/leaflet versus no letter/leaflet.**  Cost-effectiveness data were extracted from one (Arundel 2017) out of the two studies included in this comparison. Adding a recruitment leaflet has an estimated ICER of £208.38, with incremental costs of £2.08 (consisting of inflation-adjusted printing, packing, supply and labelling unit costs) and a partial effectiveness of 1%. However, the partial effectiveness of this strategy is not statistically significant (RD: 0.01, 95% CI: from -0.01 to 0.02), which introduces uncertainty regarding its cost-effectiveness figure.

**13. Providing information by video vs standard information.** Cost-effectiveness data were extracted from one (Rogers 2019)out of the four studies included in this comparison. Providing information by video is dominated by standard information, as it is both more costly (with incremental costs of £41.58 consisting of inflation-adjusted video production, DVD manufacture, packaging and postage unit costs) and less effective (with a partial effectiveness of -2%). However, the partial effectiveness of this strategy is not statistically significant (RD: from -0.02, 95% CI: -0.07 to 0.04), which introduces uncertainty regarding its dominance by standard information.

**22. Providing a brief PIL alongside the standard PIS .** Cost-effectiveness data were extracted from the single study included in this comparison (White 2023). Providing a brief PIL alongside the standard PIS, versus standard PIS only, has an undefined ICER, as the difference in effectiveness (the denominator) is zero (RD: 0.00, 95% CI: from -0.01 to 0.01). This strategy presents an incremental cost of £0.57 consisting of the inflation-adjusted brief PIL unit costs. Overall, adding a brief PIL alongside the standard PIS results in a small increase in cost with no difference in recruitment.[2]

**29. Multimedia information (provided as link/QR code) + paper PIS vs paper PIS only.** Cost-effectiveness data were extracted from one (Jolly 2019 (START MMI PSM-COPD)) out of the seven studies included in this comparison. Multimedia information plus paper PIS is dominated by paper PIS only, as it is both more costly (with incremental costs of £0.78 consisting of the intervention inflation-adjusted unit cost) and less effective (with a partial effectiveness of -1%). However, the partial effectiveness of this strategy is not statistically significant (RD: -0.01, 95% CI: from -0.03 to 0.01), which introduces uncertainty regarding its dominance by paper PIS only.

#### Interventions aimed at the recruiter or recruitment site ‐ category F

**3. Remote vs face-to-face site initiation visits.** Cost-effectiveness data were extracted from the single study included in this comparison (Jefferson 2018). Remote visits dominate face-to-face site initiation visits, as they are both less costly (with inflation-adjusted unit cost savings of £42.29 taking into consideration travel costs and the average cost of setting up a site) and more effective (with an effectiveness of 6%). However, the effectiveness of this strategy is not statistically significant (RD: 0.02, 95% CI: from -0.05 to 0.10), which introduces uncertainty regarding its dominance over face-to-face visits.

#### Incentives ‐ category G

**1. Monetary incentive versus no incentive.** Cost-effectiveness data were extracted from all 12 studies included in this comparison (Fairhurst 2022; Free 2010; Giguere 2015; Halpern 2021a ($200); Halpern 2021a ($500); Halpern 2021b ($100); Halpern 2021b ($300); Jennings 2015a; Jennings 2015b; Jennings 2015c; Jennings 2015d; Jennings 2015e). Monetary incentives, versus no incentives, have an estimated ICER of £3,052.96, with incremental costs of £152.65 (consisting of the weighted-average and inflation-adjusted amount of the monetary incentive which varied from £5 to US$500 (approximately £340) across the included studies) and an effectiveness of 5%. The effectiveness of this strategy is also statistically significant (RD: 0.05, 95% CI: from 0.01 to 0.09), which raises the certainty regarding its cost-effectiveness figure. Nevertheless, due to the substantial heterogeneity in the amounts of monetary incentives offered, sensitivity analyses, by different levels of monetary incentive (low-up to £5, medium-around £100, high-above £100) were performed to assess the robustness of these findings, and the results are presented in 52a-c.

**1a. Monetary incentive (up to £5) versus no incentive.** Cost-effectiveness data were extracted from two studies (Fairhurst 2022 (£5); Free 2010 (£5)). Monetary incentives of up to £5, versus no incentives, have an estimated ICER of £229.33, with incremental costs of £6.88 (consisting of the inflation-adjusted monetary incentives of £5 evaluated from the two included studies) and an effectiveness of 3%. However, the effectiveness of this strategy is not statistically significant (RD: 0.03, 95% CI: from -0.02 to 0.07), which introduces uncertainty regarding its cost-effectiveness figure.

**1b. Monetary incentive (around £100) versus no incentive.** Cost-effectiveness data were extracted from six studies (Halpern 2021b ($100); Jennings 2015a-Jennings 2015e (£100)). Monetary incentives of around £100, versus no incentives, have an estimated ICER of £4,279.82, with incremental costs of £128.39(consisting of the weighted-average and inflation-adjusted monetary incentives of around £100 evaluated from the six included studies) and an effectiveness of 3%. However, the effectiveness of this strategy is not statistically significant (RD: 0.03, 95% CI: from -0.02 to 0.08), which introduces uncertainty regarding its cost-effectiveness figure.

**1c. Monetary incentive (above £100) versus no incentive.** Cost-effectiveness data were extracted from four studies (Giguere 2015 ($300); Halpern 2021a ($200); Halpern 2021a ($500); Halpern 2021b ($300)). Monetary incentives of above £100, versus no incentives, have an estimated ICER of £3,011.60, with incremental costs of £301.16 (consisting of the weighted-average and inflation-adjusted monetary incentives of above £100 evaluated from the four included studies) and an effectiveness of 10%. However, the effectiveness of this strategy is not statistically significant (RD: 0.10, 95% CI: from -0.02 to 0.21), which introduces uncertainty regarding its cost-effectiveness figure.

**2. Monetary incentive (change in value).** Cost-effectiveness data were extracted from all two studies included in this comparison (Halpern 2021a ($500); Halpern 2021b ($300)). Changing monetary incentives has an estimated ICER of £8,214.83, with incremental costs of £246.44 (consisting of the weighted-average and inflation-adjusted differences in monetary incentives between the intervention and the comparison groups evaluated from the two included studies) and an effectiveness of 3 %. However, the effectiveness of this strategy is not statistically significant (RD: 0.03, 95% CI: from -0.03 to 0.19), which introduces uncertainty regarding its cost-effectiveness figure.

**3. Non-monetary incentive (Trial branded pen versus no pen).** Cost-effectiveness data were extracted from all three studies included in this comparison (Fairhurst 2022; White 2023; Whiteside 2019). Trial branded pens, versus no pen, have an undefined ICER, as the difference in effectiveness (the denominator) is zero (RD: 0.00, 95% CI: from -0.01 to 0.01). This strategy presents an incremental cost of £0.45 consisting of the inflation-adjusted pen unit costs. Overall, trial branded pens result in a small increase in cost with no difference in recruitment.

**Table 7a. Cost-effectiveness of recruitment strategies**

|  |  |  |  |  |  |
| --- | --- | --- | --- | --- | --- |
| **Comparison number** | **Comparison** | **Source** | **Effectiveness**  **(Risk Difference)** | **Incremental cost**   **(£, 2023 price level)** | **ICER (Cost per additional potential participant recruited)** |
| Trial conduct changes ‐ category C-9 | Telephone reminder vs SMS reminder | Bracken 2019 | 0.05  (95% CI: from -0.01 to 0.11) | £3.64 | £72.72  Incremental effectiveness is not statistically significant. |
| Modification to the information given to potential participants about the trial ‐ category E-11 | Recruitment primer letter/leaflet vs no letter/leaflet | Arundel 2017 | 0.01  (95% CI: from -0.01 to 0.02) | £2.08 | £208.38  Effectiveness is not statistically significant. |
| Modification to the information given to potential participants about the trial ‐ category E-13 | Providing information by video vs standard information | Rogers 2019 | -0.02  (95% CI: from -0.07 to 0.04) | £41.58 | Providing information by video is dominated by standard information. Incremental effectiveness is not statistically significant. |
| Modification to the information given to potential participants about the trial ‐ category E-22 | Providing a brief PIL alongside the standard PIS | White 2023 | 0.00 (95% CI: from -0.01 to 0.01) | £0.57 | Small increase in costs with no difference in recruitment. |
| Modification to the information given to potential participants about the trial ‐ category E-29 | Multimedia information {provided as link/QR code} + paper PIS vs paper PIS only | Jolly 2019 (START MMI PSM-COPD) | -0.01  (95% CI: from -0.03 to 0.01) | £0.78 | Multimedia information is dominated by paper PIS only.  Incremental effectiveness is not statistically significant. |
| Interventions aimed aimed at the recruiter or recruitment site‐ category F-3 | Remote vs face-to-face site initiation visits | Jefferson 2018 | 0.02  (95% CI: from -0.05 to 0.10) | £42.29 | Remote visits dominate face-to-face site visits. Incremental effectiveness is not statistically significant. |
| Incentives‐ category G-1 | Monetary incentive versus no incentive | Fairhurst 2022;  Free 2010;  Giguere 2015; Halpern 2021a ($200); Halpern 2021a ($500); Halpern 2021b ($100); Halpern 2021b ($300)  Jennings 2015a; Jennings 2015b; Jennings 2015c; Jennings 2015d; Jennings 2015e | 0.05  (95% CI: from 0.01 to 0.09) | £152.65 | £3,052.96 Incremental effectiveness is statistically significant. |
| *Incentives‐ category G-1a* | *Monetary incentive (up to £5) versus no incentive* | Fairhurst 2022; Free 2010 | *0.03 (95% CI: from -0.02 to 0.07)* | *£6.88* | *£229.33*  *Incremental effectiveness is not statistically significant.* |
| *Incentives‐ category G-1b* | *Monetary incentive (around £100) versus no incentive* | Halpern 2021b ($100)  Jennings 2015a*;* Jennings 2015b*;* Jennings 2015c*;* Jennings 2015d*;* Jennings 2015e | *0.03 (95% CI: from -0.02 to 0.08)* | *£128.39* | *£4,279.82*  *Incremental effectiveness is not statistically significant.* |
| *Incentives‐ category G-1c* | *Monetary incentive (above £100) versus no incentive* | Giguere 2015; Halpern 2021a ($200); Halpern 2021a ($500); Halpern 2021b ($300) | *0.10 (95% CI: from -0.02 to 0.21)* | *£301.16* | *£3,011.60*  *Incremental effectiveness is not statistically significant.* |
| Incentives‐ category G-2 | Monetary incentive (change in value) | Halpern 2021a ($500); Halpern 2021b ($300) | 0.03 (95% CI: from -0.03 to 0.19) | £246.44 | £8,214.83  Incremental effectiveness is not statistically significant. |
| Incentives‐ category G-3 | Non-monetary incentive (Trial branded pen versus no pen) | Fairhurst 2022; White 2023; Whiteside 2019 | 0.00 (95% CI: from -0.01 to 0.01) | £0.45 | Small increase in costs with no difference in recruitment. |
