# Supplementary material for: Strategies to improve recruitment to randomised trials
Source: Cochrane Database Syst Rev. 2026 Sep 15;2026(9):MR000013. doi: 10.1002/14651858.MR000013.pub7 (PMC13576036; doi:10.1002/14651858.MR000013.pub7)
Supplement: Supplementary file 13 [file MR000013-SUP-13-other.html]

Impact of recruitment strategies on retention rates&nbsp;


# Supplementary material 13 to: Strategies to improve recruitment to randomised trials

Parker A, Mongelli G, Piccolo-Lawrance C, Coleman E, Gkekas A, Wang HI, Dawson S, Green H, Way R, Rai A, Bruhn H, Sullivan F, Boxall C, Chan M, Manson P, Hewitt C, Fairhurst C, Clarke M, Treweek S
  
https://doi.org/10.1002/14651858.MR000013.pub7

The material in this section has been supplied by the author(s) for publication under a Licence for Publication and the author(s) are solely responsible for the material. Cochrane has reviewed this material, but Cochrane has not copyedited, formatted or proofread. Cochrane accordingly gives no representations or warranties of any kind in relation to, and accepts no liability for any reliance on or use of, such material.

Back to top

# Impact of recruitment strategies on retention rates

#### Modification to the information given to potential participants about the trial ‐ category E

*32E. Handwritten personalised invitation letter vs standard letter*

1. A handwritten personalised invitation leaflet may reduce retention compared to a standard invitation leaflet. RD = -4% (reduced recruitment) (95% CI = -8% to 0%); Analysis 32.2. This is based on one study: McCaffery 2019 (falls prevention) RoB: unclear.

*43E. PPI-designed-and-informed PIL vs. standard researcher-designed PIL*

1. It is very uncertain whether a PPI-designed-and-informed PIL affects retention compared to a standard researcher-designed PIL. RD =-4% (reduced recruitment) (95% CI = -9% to 18%); Analysis 43.2. This is based on one study: Dwyer 2022 (cognitive rehabilitation intervention for multiple sclerosis) RoB: unclear.

*47E. Multimedia information (viewed in person) + paper PIL vs paper PIL only*

1. It is very uncertain whether providing multimedia information (viewed in person) alongside a paper PIS affects retention compared to a paper PIS only. RD = 28% (95% CI = -79% to 136%); Analysis 47.2. This is based on three studies: Knapp 2023a (BALANCE)(paediatric lazy eye) RoB: low; Knapp 2023b (BAMP)(paediatric underbite) RoB: low; Knapp 2023f (UKALL)(paediatric and adolescent leukemia or lymphoma) RoB: low.

*48E. Multimedia information (viewed in person) PIL vs paper PIL*

1. It is very uncertain whether providing a multimedia information PIS (viewed in person) may affect retention compared to a paper PIS only. RD = 22% (95% CI = -51% to 96%); Analysis 48.2. This is based on three studies: Knapp 2023a (BALANCE)(paediatric lazy eye) RoB: low; Knapp 2023b (BAMP)(paediatric underbite) RoB: low; Knapp 2023d (FORCE)(paediatric wrist fracture) RoB: low.

*49E. Multimedia information (provided as link/QR code) + paper PIL vs paper PIL*

1. Providing multimedia information in the form of a link or QR code alongside a paper PIS makes little or no difference to the likelihood of being retained compared to a paper PIS only. RD = 0% (95% CI = -2% to 3%); Analysis 49.2. This is based on 5 studies: Jolly 2019 (START MMI PSM-COPD)(COPD) RoB: low; Knapp 2023e (Thermic-3)(paediatric congenital heart defects) RoB: low; Knapp 2023c (CHAMP-UK)(paediatric short-sightedness) RoB: low; Madurasinghe 2023c (START MMI HI-Light)(vitiligo) RoB: low; Madurasinghe 2023d (START MMI seAFOod) (bowel cancer prevention) RoB: low.

*50E. Multimedia information (provided as link/QR code) vs paper PIL*

1. Providing multimedia information in the form of a link or QR code probably increases retention (assessed at 3 months) compared to a paper PIS only. RD = 13% (95% CI = 0 % to 26%); Analysis 50.2. This is based on two studies: Knapp 2023e (Thermic-3)(paediatric congenital heart defects) RoB: low; Knapp 2023c (CHAMP-UK)(paediatric short-sightedness) RoB: low.

#### Interventions aimed at the recruiter or recruitment site ‐ category F

*50F. Remote vs face-to-face site initiation visits*

1. Remote site initiation visits may make little or no difference to retention compared to face-to-face site initiation visits. RD = 1% (95% CI = -6% to 9%); Analysis 56.2. This is based on 1 study: Jefferson 2018 (cast versus surgery for wrist fracture) RoB: low.

#### Incentives ‐ category G

*53G. Non-monetary incentive (branded pen vs no pen)*

1. Offering a non-monetary incentive in the form of a trial-branded pen alongside the trial invitation probably has no effect on retention. RD = 0% (95% CI = -2% to 2%); Analysis 65.2. This is based on 1 study: Whiteside 2019 (falls prevention) RoB: low.
